# Factors driving and inhibiting stunting reduction acceleration programs at district level: A qualitative study in West Sumatra

**DOI:** 10.1371/journal.pone.0283739

**Published:** 2023-03-31

**Authors:** Syafrawati Syafrawati, Nur Indrawati Lipoeto, Masrul Masrul, Novianti Novianti, Gusnedi Gusnedi, Andi Susilowati, Asrawati Nurdin, Idral Purnakarya, Andrafikar Andrafikar, Hermita Bus Umar

**Affiliations:** 1 Department of Public Health, Faculty of Public Health, Andalas University, Padang, Indonesia; 2 Department of Nutrition, Faculty of Medicine, Andalas University, Padang, Indonesia; 3 Centre of Global Health and Health Technology Policy, Ministry of Health, Padang, Indonesia; 4 Department of Nutrition, Health Ministry Polytechnic of Padang, Padang, Indonesia; 5 Department of Paediatrics, Faculty of Medicine Andalas University, Padang, Indonesia; 6 Department of Nutrition, Faculty of Public Health, Andalas University, Padang, Indonesia; Universidad Nacional Autonoma de Nicaragua Leon, NICARAGUA

## Abstract

Stunting prevalence varies significantly across Indonesian provinces and districts/municipalities, including West Sumatra. This research aims to identify the driving and inhibiting factors for implementing the stunting reduction acceleration program at the district level in West Sumatra. The research was conducted in 2022 with a qualitative study design and a case study approach. Data were collected through (i) group interviews with key informants involving policymakers and program managers at the provincial, district, sub-district, and village levels and (ii) in-depth interviews with mothers of stunted children. Two districts were selected as research sites. One district represents an area that has yet to be developed (District A or failed district). The other district has seen a significant reduction in the prevalence of stunting (District B or successful district). We found several driving and inhibiting factors that affect the reduced prevalence of stunting, such as the need for the relevant agency to play a strong role as the leading and responsible sector for stunting convergence actions. Another important factor is paying close attention to improving the quality of stunting data and providing adequate funding support. High commitment from Public Health Centers to maternal health through classes for pregnant women, infants, and children under the age of five can also have an impact. Furthermore, properly performing duties by assigned actors in specific and sensitive programs and integrated monitoring and evaluation of program implementation and outcomes can influence stunting prevalence. Recommendations for accelerating stunting reduction include improving communication and coordination, establishing stunting prevalence reduction as a performance indicator among the related district government and its various agencies, and assisting the village administration with planning and budgeting to support stunting prevention.

## Introduction

The term "stunting" describes a condition in which a child’s height-for-age is more than two standard deviations below the WHO Child Growth Standards median. Stunting is caused by undernutrition, repeated infections, and insufficient psychosocial stimulation. Stunting has been increasingly recognized as a global health issue, attracting the attention of many health practitioners for various reasons. First, stunting is a serious issue affecting many children worldwide. Second, stunting can have serious long-term effects on health and self-functioning, including a decrease in intelligence, a loss of productivity, and a decrease in worker wages. Third, there is a need for globally agreed-upon definitions and standards for normal human growth. The fourth requirement is agreement on the critical window, which runs from pre-pregnancy to the first two years of life. Fifth, stunting is a multi-sectoral issue that necessitates a multi-sectoral response [1–3].

Stunted children have higher mortality and morbidity rates, as well as deficits in cognitive and motor development. Furthermore, stunting is estimated to be responsible for 17% of the mortality burden in children under five [4].

According to UNICEF/WHO and the World Bank, stunting affects approximately 151 million children worldwide or 22.2% of all children. Asia alone has approximately 83.8 million stunted children, most of whom live in South and Southeast Asia. Indonesia is a country with a high prevalence of malnutrition hence stunting [5]. According to data published by the Ministry of Health in 2021 based on the results of the SSGI (Indonesian Nutrition Survey) 2021, the prevalence of stunting in Indonesia is 24.4% [6].

According to the World Health Organization’s (WHO) "Conceptual Framework on Childhood Stunting," stunting is caused by a complex interaction of various factors such as households, environments, socioeconomics, education, food-agriculture, and culture [7]. Because of the complexities involved, it is unlikely that stunting in children can be resolved with a single nutritional intervention. Instead, multilevel, complex, and coordinated nutrition-sensitive and nutrition-specific interventions involving collaboration between health and non-health actors are required [8].

The multi-sectoral implementation of convergent interventions involving districts/municipalities and villages is the key to successful stunting prevention in various countries [9]. For example, after a long period of stagnation, Peru has reduced its national stunting prevalence due to a combination of social determinants and cross-sectoral factors [10]. One of the factors that contributed to Senegal’s success in reducing stunting was its emphasis on nutrition improvements and multi-sectoral nutrition efforts [11]. Similarly, the reduction in stunting in Ethiopia was driven by targeted nutrition interventions, focusing on agriculture, access to health care, sanitation, and education [12].

In 2018, Indonesia developed a National Strategy for Accelerating Stunting Prevention. The National Strategy is used as a guide to encourage collaboration among agencies in order to ensure the convergence of all stunting prevention programs/activities [13]. Implementing integrated stunting reduction interventions requires a multi-sectoral approach. As a result, a multi-sectoral team is required to carry out this integrated action. Health, agriculture, food security, marine and fisheries, education, industry, social aspects, religion, communication and information, public works and housing, community empowerment, women’s empowerment and child protection, population and family planning, and food and drug monitoring are all represented on the team [14].

West Sumatra Province has successfully reduced the prevalence of stunting from 31.2% to 23.3%, according to data from the Riskesdas 2018 (The Basic Health Survey 2018) and the SSGI 2021. The annual rate of decline is 2.6%. The total number of people in a population who have a disease or health condition at a given time is referred to as prevalence. The prevalence of stunting is defined as the proportion of children under the age of five in a population who are two standard deviations below the expected height for their age [3, 15].

The achievements of the districts and municipalities in West Sumatra province in reducing the prevalence of stunting vary considerably. While some districts, such as Agam, 50 Kota, and Pasaman Barat, could reduce stunting by more than 10% in three years, others, such as Solok, Pasaman, and Pesisir Selatan, only managed to reduce stunting by 0.4 to 1.7%. Stunting rates even increased in several districts/municipalities (Riskesdas 2018 & SSGI 2021).

There have been no studies conducted to determine the driving and inhibiting factors for implementing the stunting reduction program in West Sumatra. This study was conducted to fill the gap. Two districts in West Sumatra were chosen as research sites for this purpose. One district has significantly reduced stunting prevalence, while the other has failed. The experience of these two districts can help other districts, particularly in West Sumatra, anticipate factors that drive and inhibit their stunting prevention programs.

## Methods

### Study design

The research was designed to employ a qualitative approach with a case study approach. To that end, two districts were selected as research sites based on their rate of stunting reduction in the last three years (2018–2021). One of the districts, designated as "District A," represents a region with a low rate of stunting reduction, occasionally also referred to as failed district. The prevalence of stunting in District A ranges from 41.8% in 2018 to 40.1% in 2021. The decrease was only 1.7% in three years.

In contrast, the other district, District B, has succeeded in reducing its prevalence of stunting, occasionally also referred to as the successful district. This district has reduced the prevalence of stunting by 11.1%, from 35.1% in 2018 to 24% in 2021, and is confident of meeting the government’s national stunting prevalence rate target of less than 14% by 2024. This qualitative approach was chosen because it allowed a more in-depth examination of informants’ experiences with the stunting prevention program in their respective areas. Such analyses enabled the identification of driving and inhibiting factors for program implementation.

### Conceptual framework

**Fig 1:**
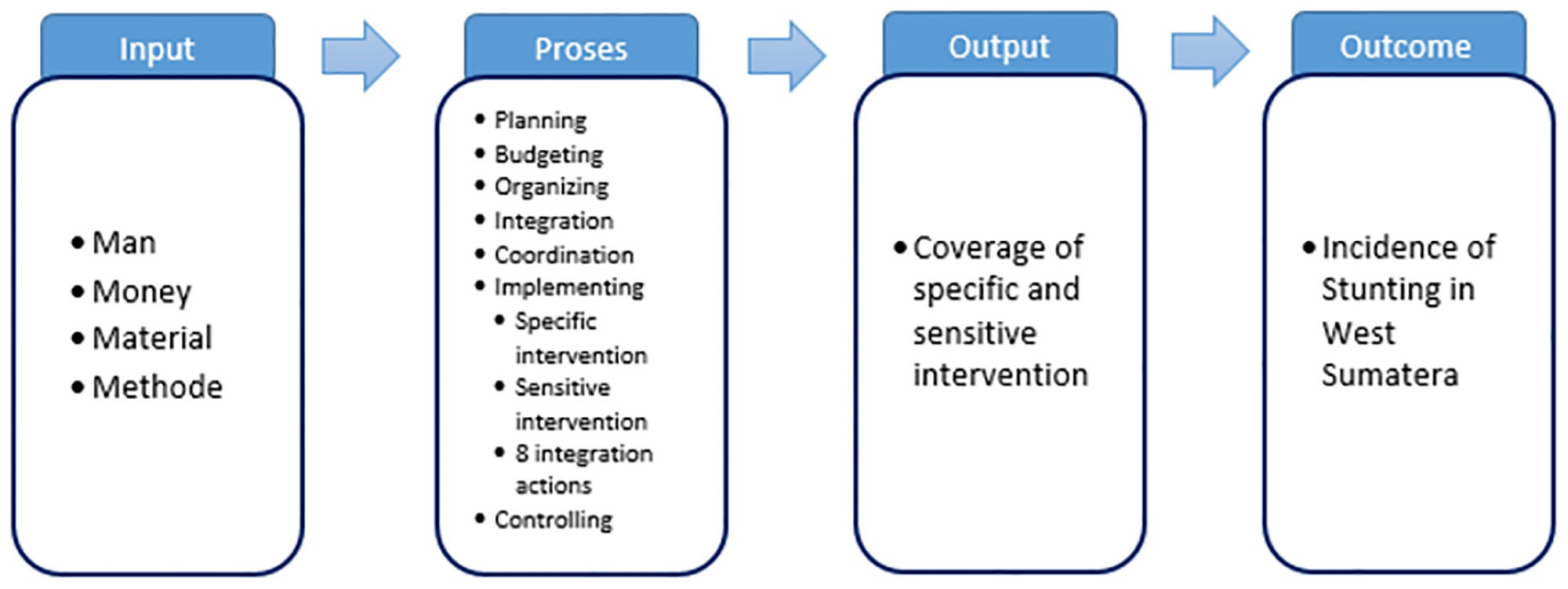
Theoretical framework for analysing driving and inhibiting factors in stunting reduction acceleration program in West Sumatra in 2021.

The IPOO model served as the theoretical framework for this study. This theoretical framework was chosen because it is a simple and widely accepted model for the performance of government organizations. The model was then used to design the study and guide the interview. As shown in Fig 1 below, the IPOO model consists of inputs, processes, outputs, and outcomes.

Inputs are the elements and factors fed into an organization’s black box. A process can be thought of as a series of logically linked transactions that convert inputs into outputs. Inputs are transformed in the black box of processes to produce outputs. Outcomes are not the direct result of the processes; rather, they are the result of the entire IPOO model and have significant and far-reaching consequences for society as a whole [16].

The availability of personnel, funds, infrastructure, Standard Operating Procedures (SOP), and supporting equipment for the program is included in the input category. We used a management approach in the process category. Management is the process of determining and achieving stated objectives by planning, organizing, executing, and controlling human beings and other resources [17]. The achievement of the stunting reduction target falls under the output category. Furthermore, the discussion groups discussed program innovation, local wisdom, and the presence or absence of potential empowerment in the region.

### Identification of informants

Semi-structured interview guides were distributed to informants beginning on March 21, 2022. The informants were chosen primarily because of their high involvement in the program implementation. They were purposively sampled, taking into account their diversity and categories.

The Development Planning, Research and Development Services, Community Empowerment Agency, Public Health Office, Public Works and Housing Office, Agriculture and Food Security Office, and Social Service were among the institutions involved in tackling stunting in West Sumatra. These organizations were chosen because they have collaborated across sectors to reduce stunting in their respective regions. The agency’s informants included the head of the respective institution and their subordinates, who were directly involved in stunting prevention programs on a practical level. As a result, one agency could be represented by more than one person. When this was the case, these informants could supplement each other’s information about their efforts to reduce stunting in their area.

**Fig 2:**
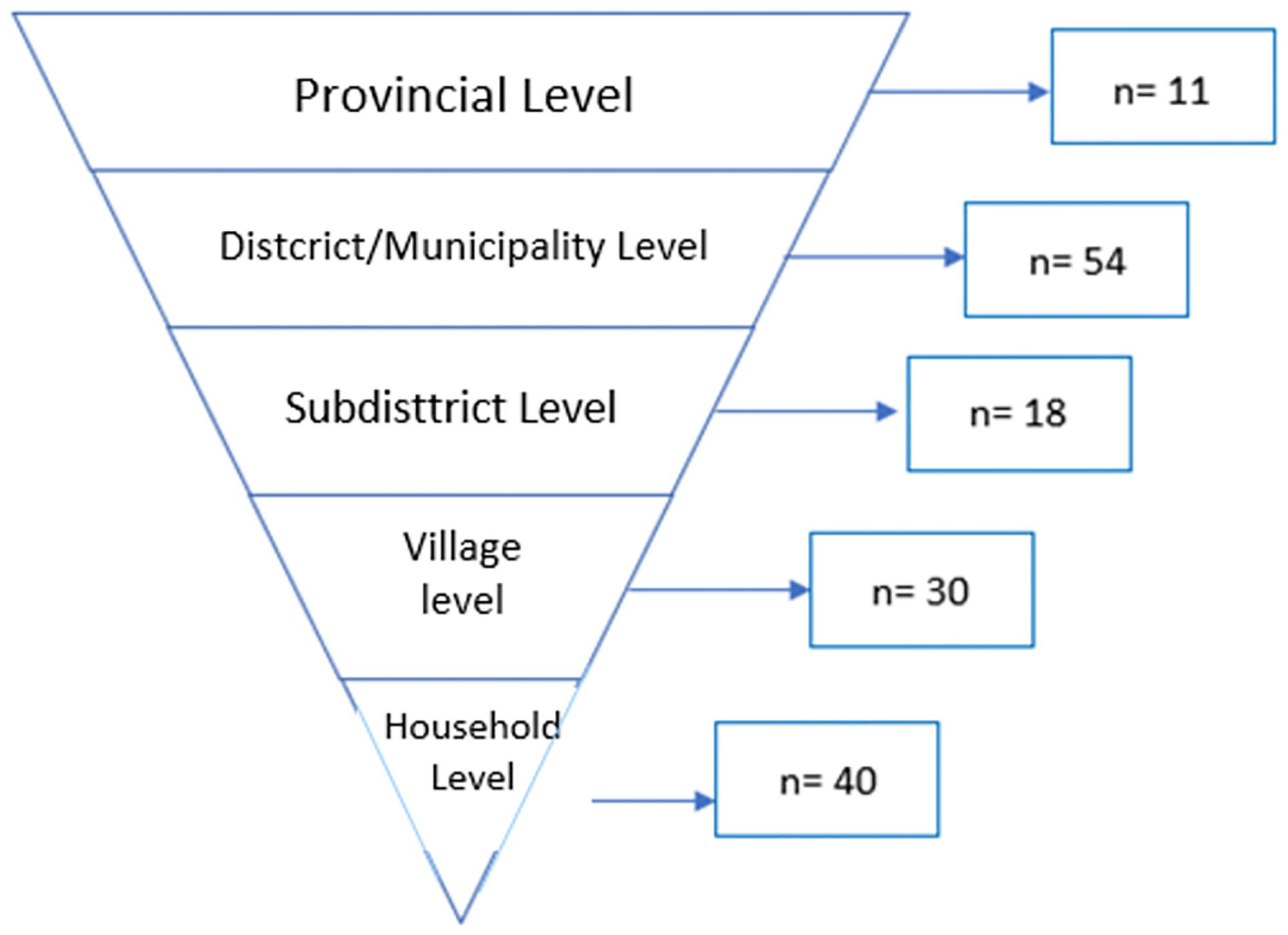
Diagram of levels of informant groups.

As shown in Fig 2, this study includes policymakers, policy managers, and target groups comprised of five levels of informant groups.

1. Provincial Level: Head/staff of BAPPEDA (Regional Development Planning, Research, and Development Agency), BPM (Community Empowerment Agency), Public Health Office, Public Works and Housing Office, Agriculture and Food Security Office, and Social Service.
2. District/Municipality Level: Head/staff of BAPPEDA, DPMN (Community and Village Empowerment Office), Public Health Office, head of Public Works and Housing Office, Agriculture Office, Food Security, and Social Service.
3. Sub-district Level: Head of Public Health Center, TPG (Nutritionist), Village Midwife.
4. Village Level: Head of the village, PKK (Family Welfare Development), (Village Forum Agency) and KPM (Cadres of Community Empowerment).
5. Household Level: Head of the family with stunting, expectant mothers, and breastfeeding mothers.

This study involved policymakers, managers, and forty target groups. The distributions of these informants are shown in Table 1.

**Table 1:** Distributions of participating informants.

Techniques	Sector	Informant			
		West Sumatra Province	District A^a^	District B^b^	Total
		n	n	n	n
Group interviews at the provincial and district level	Health	3	2	3	8
	Agriculture	1	4	2	7
	Fisheries and Food Security	1	2	8	11
	Public Works and Housing	1	2	2	5
	Social	3	2	6	11
	Community Empowerment	1	4	6	11
	Regional Development Planning	1	2	4	7
	Population and Family Planning	-	-	5	5
	Public Health Center	-	8	10	18
**Total**		11	26	46	83
Group interviews at Village level	Head of Village	-	2	3	5
	Secretary of Village	-	2	3	5
	Forum Agency	-	2	3	5
	Cadre	-	3	5	8
	Family Welfare Development	-	2	2	4
	Village Staff	-	-	3	3
	**Total**	0	11	19	30
In-depth interviews at the household level	Mothers of children under five years of age, expectant mothers, and breastfeeding mothers.		20	20	40

^a^ district that failed to reduce the stunting rate (failed district).

^b^ district that has successfully reduced stunting significantly (successful district).

The Public Health Office and Community Health Center informants were asked about implementing specific nutrition interventions. Meanwhile, BAPPEDA, Community Empowerment Agency, Public Works and Housing, Agriculture Office, Food Security Office, and Social Service informants were queried about implementing sensitive nutrition interventions. The village-level informants were asked about the village funds spent on stunting prevention. In-depth interviews with mothers of stunted children under the age of five and expectant mothers were conducted in order to cross-check the implementation of specific and sensitive programs and ensure that they met the intended targets. The target groups’ information is used to implement sensitive and specific interventions, such as the availability of latrines, clean water facilities, KIA (Maternal and Child Health) books, and so forth. The information gathered from the target groups includes the implementation of sensitive and specific interventions such as the availability of latrines, clean water facilities, KIA (Maternal and Child Health) books, PKH (Family Hope Program) cards, National Health Insurance cards, government-supplied additional food, and participation in classes for expectant mothers and mothers with children under the age of five. In addition to these in-depth interviews, the team visited the target groups at their homes and documented any findings related to the stunting program.

### Data collection

Prior to data collection, the following procedures were followed to select informants:

1. Informants at Provincial, district, and village levels.
  We first identified the provincial, district, and village stakeholders involved in the stunting reduction program. We then sent them a letter requesting consent to participate as an informant. We scheduled an in-depth interview once both parties agreed. All informants agreed to participate in the study voluntarily.
2. Informants at household level
  The village midwife selected household informants based on data from the Public Health Center on stunting toddlers. Village cadres and midwives then accompanied the researchers to interview the selected households. All informants agreed to be interviewed after hearing about the study from village cadres or midwives.

The team included five nutritionists, one pediatrician, one policy expert, one sociologist, and one public health expert who worked together on data collection and analysis. Before data collection, training was conducted to establish and ensure that the informants would be asked the necessary questions. Before the interview, checklist sheets for observation and document review were prepared.

The information was gathered through group discussions with policymakers and program managers and in-depth interviews with the target groups. Table 2 shows the details of the stunting program that the district government must implement.

**Table 2:** Programs related to stunting by the district government and its various agencies.

Agency	Activity
Health	Development in increasing community nutritional status. Development in increasing community nutritional knowledge. Development in stunting prevention. Implementation of health promotion strategies Increasing Nutritional Surveillance. Strengthening interventions of nutrition supplementation for expectant mothers and children under five years of age. Supplying supplementary food for expectant mothers with Chronic Energy Deficiency (CED). Supplying supplementary food for children under five years of age with malnutrition (Supplementation of micronutrients). Development in increasing delivery services in Health Facilities. Development in implementations of Community-Based Total Sanitation (STBM). Services in Filariasis and Helminthiasis control.
Agriculture and Food Security	KRPL (Sustainable Home Garden Area program) Self-fulfilled food area.
Marine and Fisheries	Marketing and promoting marine and fisheries products
Public Works and Housing	Intensive-labour sanitation in villages. PAMSIMAS (Community-based supply for drinking water and sanitation)/SPAM (Drinking Water Supply System) in villages.
Family Planning	Increasing promotion of nursery of the first 1000 days of life.
Social	Family Development Session (FDS) in the PKH (Family Hope Program). KPM (Human Empowerment Cadre) receives food as a social aid.
Village Community Empowerment	Use of village funds
Regional Development and Planning	Coordination of budgeting for stunting reduction acceleration program. Strengthening coordination of planning for stunting reduction acceleration. Advocacy of implementation of stunting reduction acceleration policy.

In-depth interviews with mothers of stunted children under the age of five and expectant mothers were conducted in order to cross-check the implementation of specific and sensitive programs and ensure that they met the intended targets. The information gathered from the target groups includes the implementation of sensitive and specific interventions such as the availability of latrines, clean water facilities, KIA (Maternal and Child Health) books, PKH (Family Hope Program) cards, National Health Insurance cards, government-supplied additional food, and participation in classes. In addition to these in-depth interviews, the team visited the target groups’ homes and recorded any findings related to the stunting program.

### Data analysis

The interviews were transcribed by research assistants who carefully listened to the audio recording three to four times. In order to achieve transcription accuracy, they also corrected the errors. We analyzed the data using content analysis, which allowed us to group words into several content-related categories and count the number of instances that fell into each category [18]. We then read the transcription carefully to get a sense of the whole from the research before breaking it down into smaller meaning units. Each identified meaning unit was assigned a code, which should be understood in context [19].

The deductive coding method was used for coding. We had created a coding list in this manner before beginning the analysis process. To this end, the conceptual framework and a list of research questions were used. These are Input (Man; Money; Material; and Method); Process (Planning; Budgeting; Organizing; Implementing; Monitoring, and Evaluation); and Output (Coverage of Specific and Sensitive Intervention), which developed into a category and subcategory [20, 21]. This study’s theme is *Driving and Inhibiting Factors of Stunting Reduction Acceleration Programs*, and the sub-theme is *Stunting Factors* and *Inhibiting Factors of Stunting Reduction Acceleration Programs*.

The codes were also analyzed by frequency to determine and compare their occurrence in each category (e.g., "the majority of answers stated…"). As is customary in content analysis, the written summary included the number of people who provided similar responses, and qualitative quotes were used to exemplify each response category [22]. We then compared the conditions in each district category: the district that successfully reduced its stunting was compared to the district that failed. Finally, the discovered categories were related to the research theme.

### Ethics approval and consent to participate

The research ethics committee of the Faculty of Medicine at Andalas University granted ethical approval (No: 616/UN.16.2/KEP-FK/2022) for this research on March 4, 2022 by Dr. dr. Yuliarni Syafrita, SpS (K) and Dr. dr. Afriwardi, SH. Sp.KO,MA. Before any data was collected, informed consent was obtained. In response to their willingness to participate, stakeholders provided informed consent in the form of a letter. Meanwhile, household informants provided verbal informed consent, which was audio-recorded.

## Results

The following sections present the conceptual framework’s input, process, and output categories and subcategories. We present results from District A and District B for each theme.

### Driving and inhibiting factors related to input availability

#### Man

Human resources play a critical role in implementing stunting reduction programs. District B has the required human resources at the district and village levels. Although the Community Health Centers staff expressed concerns about their workload, they all agreed they had adequate human resources. Furthermore, the Integrated Service Stations (Posyandu) under study were aware of their critical roles as the program’s spearhead. As a result, the Community Health Centers established a regular schedule for the paramedics to visit and work at the Integrated Service Stations in turn.

*We are aware that our Community Health Center plays a larger role in the stunting reduction program*. *We invite all of our paramedics to attend and share the workload in relation to the schedule of our services*.

**(Head of Community Health Center in District B**)

On the other hand, District A demonstrated a lack of staff at the Community Health Centers to implement specific stunting prevention programs. Due to this shortage, the intended goals were not reached. Additionally, the area’s size should be taken into account. To put it into perspective, one village midwife must oversee and coordinate four Community Health Centers. This problem seems connected to the KPM in this area (Human Empowerment Cadre). On closer examination, it became apparent that KPM employees had not performed their duties properly. Instead of empowering the locals to increase public awareness of the importance of preventing and treating stunting in children under age five, they were more preoccupied with administrative tasks like reporting and disbursing village funds.

#### Money

The government agencies in District B were found to have allocated funds for stunting programs in the form of DAK (Special Grant). The district’s Community Health Centers received the most funding from the BOK (Health Operational Aid) and the Village Fund. This is in contrast to District A. Most government agencies in this district reported that they had not received a Special Grant for stunting reduction programs. In fact, only a few government agencies used the stunting prevention fund to build infrastructure, as the Public Works and Housing Office did. Another issue was found to have related to ineffective budgeting in that a significant portion of the budget was only allocated for empowerment activities, coordination, synergy, assistance, and monitoring and evaluation, while funding for program implementation was overlooked. To complicate things, the Community Health Centers found it difficult to allocate funds for stunting reduction due to the rigid funding process. Furthermore, the Community Health Centers were not permitted to use village funds managed by the village administration. As a result, most stunting programs fell short of their goals.

*“We only manage funds received from the central government because the activities here are more about empowerment and coordination*. *There is no special grant available*.’

**(Staff of DPMN in District A**)

*“The fund was spent on activities*, *such as coordination*, *synergy*, *assistance*, *and monitoring and evaluation*, *not particularly on stunting*”

**(Staff of BAPPEDA in District A**)

*“We received IDR 15 million from the village fund … it is not enough*. *We can’t cover all of the expenses*. *We were asked to find experts and went to the field*. *Such operational expenses are lacking*. *There is no budget for stunting*, *but for Covid expenses*, *we allocated some*”

(**Staff of PKK in District A**)

#### Material

Both District A and District B have something in common regarding the availability of tools for measuring the height of children under five years of age. While the Health Office had already budgeted for anthropometric equipment for Community Health Centers and Integrated Service Stations, it was insufficient to meet the demands in the field. Other sources of procurement were still required. The village apparatuses, for example, could purchase anthropometric equipment with village funds. They did do so. However, the measurement equipment they purchased did not meet the standard and could not be used. In general, the two districts’ supporting facilities and infrastructure for stunting interventions remain limited. As previously stated, one of the most important supporting tools is height-measuring devices that meet the standard and that are portable during home visits for children under five.

*The Village administration provides the facilities*. *But*, *a lot of the equipment we use at the Community Health Center is on loan*. *It is insufficient*. *We probably only have two sets of equipment*. *In the meantime*, *we receive three or even five visits each day*. *Most of the equipment that the Village administration procured does not meet the standard*. *They were bought with money from the village fund*.

**(Head of Community Health Center in District B**)

*“The village administration does allocate budget for the procurement because it is their policy and*, *therefore*, *it must provide the equipment*. *But*, *the equipment (it procured) does not meet the WHO standard*. *We can use the results for filtering but not as standards*”

**(Staff of Community Health Center in District A**)

#### Method

District agencies generally refer to the Regent’s Regulation as the main guideline in implementing stunting reduction acceleration programs. Most district agencies in District B had clear technical manuals obtained from the central government, provincial government, or BAPPEDA. Interestingly, the Community Health Centers developed their own SOPs for implementing stunting reduction activities while remaining committed to the Minister of Health Regulation.

*We refer to the Minister of Health Regulation No*. *82/2020 in implementing the specific interventions*. *This regulation provides guidelines*. *(We also refer to) the Minister of Health Regulation No*. *2/2020 regarding the standard anthropometric equipment*. *The Community Health Centers also produce SOPs*.

(**Staff of Community Health Center in District B**)

On the other hand, it was learned that most government agencies in District A lacked SOPs or guidelines for program implementation. Guidelines for implementing stunting interventions are not available at Community Health Centers. Consequently, these health centers lack formal rules governing the Minister of Health Regulation’s derivatives for stunting prevention.

*“(In terms of SOP) for stunting*, *I think we haven’t had one*. *As for the decree for stunting locus*, *yes*, *we have one*”

**(Staff of Community Health Center in District A**)

### Driving and inhibiting factors related to process

#### Planning

During the planning stage, the availability of data on stunting was critical because it allowed government agencies at all levels to focus their efforts. In terms of data availability, the program planning in the successful district (District B) was arguably well-planned. BAPPEDA, the government agency in charge and acting coordinator, played an important role in this case because it had collected valid stunting data that it could share with government agencies from the district to the village level. BAPPEDA has taken an innovative step in this successful district by improving stunting data regarding stunting program implementations since 2019/2020. BAPPEDA distributed data both offline and online. All team members of the stunting reduction acceleration program were involved in the offline publications by visiting sub-districts and villages in the district, whereas the online publications were made through online media.

*(In terms of) Integrated Monitoring and Evaluation of Stunting Data Improvement*, *the innovation that we have taken*, *is more about identifying the main problems*. *What we have improved is the data*, *and our concern is there*. *And (in doing this) BAPPEDA is not acting alone*. *We work with various sectors; we monitor and evaluate them*.

**(Staff of BAPPEDA in District B**).

The story is in contrast to the failed district (District A), whose overall programs appear to have been ineffective. Obstacles remain in the district, particularly the lack of data on stunting from the district to the village level. When the data is available, there is some doubt about the measurement’s validity. To make matters more complicated, the actors involved in data use displayed a sense of institutional egocentricity.

#### Budgeting

Sufficient budgeting is unquestionably essential in any running stunting-related activity. The successful district had sufficient funding to run its many stunting-related activities. For example, the Health Office in charge of specific interventions allocated sufficient funds for stunting programs. It spent approximately IDR 6.1 billion, which was saved in a separate account (added to this fund is the Delivery Security Fund, around IDR 2.5 billion). Other government agencies also provided Special Grants for a similar purpose. It was learned that the village administration collaborated with the Community Health Center in its area. Such cooperation, for example, was demonstrated by the former’s willingness to allocate significant funds to support the implementation of stunting prevention programs, particularly activities held at the Integrated Service Station in its vicinity, which served as the program’s spearhead.

*If we allocate funds for Integrated Service Stations*, *they will receive more funding*, *right*? *This is because activities at the stations are closely linked to stunting reduction programs such as mass weighing and classes for expectant mothers and mothers with children under five years of age*. *The allocated fund is expected to exceed 50%*.

(**Head of Community Health Center in District B**)

The failed district shows different funding-wise circumstances. Several government agencies in this district stated that the funds they were in charge of were not specifically for stunting prevention programs. Instead, funds for any stunting-related programs were included in other coordinated activities. Furthermore, the budget for specific nutrition intervention programs was deemed insufficient by the Community Health Centers. For example, the PMT (Supplementary Food Provision) budget for Integrated Service Stations has stopped for the last two years. Furthermore, the village administration rejected Community Health Centers’ budgeting because it had already allocated funds for similar activities. The situation worsened as the village administration’s budget remained insufficient: in 2021, the village administration allocated only IDR 15 million for stunting prevention programs. The funds were used to pay salaries or wages for staff at the Integrated Service Stations and KPM (Community Empowerment Cadres), operational costs for activities such as consumption, and remunerations for invited experts.

*“The budget is insufficient because*, *in general*, *the village fund falls short*, *such as the fund used to distribute Supplementary Food Provision (PMT)*. *We will provide more nutritional intake for expectant mothers and children under the age of five if we have more funds*.”

**(Staff of PKK in District A**)

#### Organizing

Since stunting reduction programs involve multiple sectors, it is natural that the government agencies involved require close coordination. Following the Acceleration of Stunting Prevention Guidelines, all government agencies working on the convergence of stunting prevention programs must collaborate in a coordinated manner to ensure that the activities run smoothly. BAPPEDA was crucial in this regard. The BAPPEDA of District B was found to be in charge of coordinating all activities run by the related government agencies. In addition, the BAPPEDA served as the government agency in charge of carrying out the stunting convergence activities. Furthermore, in organizing all stunting-related activities, government agencies coordinated with one another for information delivery, data sharing, and so on. BAPPEDA, as technical coordinator, ensured coordination by visiting other government agencies at all levels.

*Yes*. *Our team has good coordination*. *The head (of BAPPEDA) acts as the director*, *the person in charge*. *Apart from that*, *BAPPEDA*, *acting as the technical coordinator*, *works with several government agencies*. *This is the main thing*. *Put differently*, *the main government agency has direct responsibility for the program*.

(**Staff of BAPPEDA in District B**)

*Coordinations for the stunting reduction programs involve many government agencies*, *including Health Office*, *BKKBM*, *BAPPEDA*, *and DPMN*. *This program is integrated with other programs beyond Health Office*. *At the district meeting (Musrembang)*, *it was discussed that the cooperation would be administered multi-sectorally*.

(**Staff of Community Health Service in District B**)

On the other hand, coordination among government agencies did not appear to be working well in District A. It is known that the district’s BAPPEDA did not perform optimally in its role as the primary agency in charge of all stunting-related activities. Several inhibiting factors were discovered. According to an informant working at a provincial-level agency, the other government agencies of districts/municipalities in West Sumatra responded indifferently to stunting prevention programs. For example, the informant said they would send their subordinates (staff) rather than a high-ranking official(s) from their agency when they were invited to coordination meetings. Worse, the coordination meetings were rarely followed up on, as if they did not value the stunting programs. Cross-sectoral cooperation or assistance among government agencies is lacking in the organization of sensitive programs, such as healthy latrine programs, basic sanitation programs, and clean water supply for families with stunting toddlers from the PUPR (Public Works and Spatial Planning Office).

#### Implementing

Most of the actors in the successful district’s stunting reduction programs were able to perform their duties and functions to the best of their abilities. The Community Health Centers, for example, successfully implemented classes for expectant mothers and mothers with children under the age of five, which was one of the most important interventions to break the vicious circle of stunting. The Community Health Centers were committed to and focused on implementing such interventions in this regard. According to one of the informants:

*Each class for expectant mothers is held four times*. *Each group has ten participants*, *and each class is held all year round*. *We give a reward for a selected participant at the last meeting (the fourth session)*. *We also give certificates to all the participants*. *There is one Integrated Service Center (Posyandu) in which the staff holds a graduation ceremony*.

**(Staff Community Health Center in District B**)

Furthermore, the Community Health Centers organized a sanitation-related collective program in a hamlet. Other sensitive programs were implemented in a variety of ways. For example, the Social Service ensured that deserving families receive PKH (Family Hope Program) and BPNT (Non-Cash Food Assistance) for the first 1000 days of life, as well as parenting and nutrition education, promotion, and socialization. BAPPPEDA performed satisfactorily as the government agency in charge of and coordinator of activities.

Meanwhile, the failed district experienced difficulties carrying out many stunting prevention activities. When undertaking specific intervention activities, such as weighing months, the cadres continued to use the conventional meter as a height measurement tool. This was particularly the case when they swept children’s homes because they could not take the microtoise with them. Sweeping was required because many children under the age of five did not visit the Community Health Center. The cadres were asked to assist them in making home visits for weighing. In reality, despite having been trained through capacity building to revitalize the Integrated Service Station annually, the cadres frequently made mistakes in installing the measuring instruments. Another obstacle is the implementation of the Integrated Service Station and monitoring the growth and development of children under the age of five in specific areas. A large Community Health Center was responsible for 52 Integrated Service Stations. This complicated monitoring. Also, the transition from a locus to a non-locus Community Health Center resulted in the loss of special treatment for stunting prevention in the Community Health Center in question’s working area. Furthermore, most government-run activities failed to reach the targeted households with stunting cases. Government agencies’ data shortage on stunting was responsible for this failure.

*“However*, *when we are sweeping*, *the cadres use the conventional meter again*. *They are the ones who do the sweeping*. *There are many children to measure and it makes it impossible for the all the midwives to participate*. *The Intergrated Service Stations undrgoes a revitalitaion annually which is aimed to train the cadres to do their jobs properly*, *such as installing the measurement unit and the microtoise*. *Often*, *they did not do as they were trained in the field*, *though*. *Apart from that*, *we have a large working areas and have to monitor 52 Intergrated service Stations*. *This is impossible*, *right*?”

**(Staff of Community Health Center in District A**)

#### Monitoring and evaluation

The stunting activities in District B were well monitored and evaluated. The integrated monitoring and evaluation activities directed by BAPPEDA allowed for proper supervision in implementing stunting-related activities. The Health Office, BAPPEDA, DPMN, and Social Service team randomly selected and monitored certain Integrated Service Stations. The visits took place without prior notice. Such unannounced inspections were performed to discover how Integrated Service Stations worked and whatever obstacles they encountered, including human resources, management, etc. Monitoring and evaluation also successfully supervised the sensitive intervention activities.

*In this integrated monitoring and evaluation*, *we picked up randomly the Integrated Service Stations to be visited by the team from Health Office*, *BAPPEDA*, *DPMN*, *and Social Service*. *We visited the stations without prior notification*. *We wanted to see the real conditions of the Integrated Service Stations*

(**Staff of BAPPEDA in District B**)

On the other hand, the supervision of the implementation of stunting prevention programs in District A raised concerns. The Community Health Centers doubted their cadres’ ability to measure and weigh children under five years old and their services for expectant mothers. The Community Health Centers were uncertain whether the cadres did their jobs properly. The Community Health Centers’ staff admitted that they could not monitor all service station activities in the areas under their control when implementing community programs such as Integrated Service Stations. However, based on the reports shared by the village midwives and cadres, it is clear that they encountered challenges in the field, such as a lack of weighing measurements or steelyards.

### Output

The findings of interviews and observations in households with stunting cases are as follows. In District B, most household respondents already had a gooseneck closet (70%), and all closets were also connected to septic tanks. Meanwhile, in District A, while most household respondents had a closet (71%), only a small proportion of these closets (33%) were connected to septic tanks. In terms of clean water availability, 95% of household respondents in the successful district had access to clean water from PDAM (regional water company) (60%) and protected wells (40%). Data from the failed district show that all household respondents also had access to clean water, but the sources varied slightly. While most had access to clean water from PDAM (62%) and wells (24%) in the district, some had to rely on spring water (14%). However, it is unclear whether the springs are protected.

Regarding supplementation obtained by mothers and children, the successful district showed that most respondents (90%), and a large percentage of children under five (85%), received Fe tablets. Similarly, 100% of respondents in the failed district received FE tablets, and 90% of children under five received vitamin A. 80% of respondents from the successful district stated that they received the Supplementary Food Provision (PMT) program from the government through biscuit distribution. However, only 67% of those polled in the failed district reported receiving the PMT. During pregnancy, the biscuit program was designed to provide only a few packs of biscuits. Some respondents even claimed that they were not given biscuits.

Another distinction between District A and District B is in terms of ownership of the KMS (Growth Chart Card) and KIA (Maternal and Child Health) books. Most respondents in District B (80%) stated that they had the KMS book; 80% of them also stated that they had the KIA book. Furthermore, most respondents (90%) stated that they had a JKN (National Health Security) card or were registered as JKN members. Meanwhile, in District A, most respondents (81%) also claimed to have a KMS book, and the majority (81% claimed to have a KIA book). However, only a small proportion of respondents in this district reported having a JKN card or were registered as JKN members (48%).

Another distinctive characteristic between the two districts is associated with respondents’ participation in related classes. Only 30% of respondents in District B attended the class for expectant mothers, while 75% attended the class for mothers with children under the age of five. This is a large number. In District A, 52% of respondents attended the class for expectant mothers. However, no respondents reported having attended the class for mothers with children under the age of five.

The output results are briefly explained in Table 3 below:

**Table 3:** Implementation of stunting prevention activities in households with stunting case.

Activity Indicator		District. B**	District. A*
Clean latrine	a. Closet	70%	71%
	b. Septic Tank	100%	33%
Clean water	a. Regional Water Company	60%	62%
	b. Well	40%	24%
	c. Spring	-	14%
Supplementation received by mothers and children.	a. Tablet Fe	90%	100%
	b. Vitamin A	85%	90%
Supplementary food (biscuits) provided by the government		80%	67%
KMS Book		80%	81%
KIA Book		80%	81%
JKN Card		90%	48%
Participation	a. Class for expectant mothers	30%	52%
	b. class for mothers with children under five years of age.	75%	0%

The descriptions above allow us to group the factors driving and inhibiting stunting in West Sumatra province, as shown in Table 4.

**Table 4:** Driving and inhibiting factors of stunting reduction programs.

Item	Driving Factor	Inhibiting Factor
**Input**		
Man	Human resources related to stunting reduction programs are sufficient at the government agency or village level.	Lack of human resources, particularly at the village level; Community Empowerment Cadres merely deal with administrative tasks, overlooking community empowerment.
Money	A large fund is available and is used for funding activities in the stunting locus.	The fund is limited and is not specifically allocated for stunting locus; activities are mainly in the form of empowerment and coordination.
Material		Anthropometric equipment is insufficient and unstandardized; poor coordination between Community Health Center and village administration in procuring weighing and scaling equipment.
Method	Comprehensive regulations are available and issued by the central, provincial, and regional governments and Center Health Centers independently produce SOPs.	SOP derivatives are not produced by the Community Health Center or village administration regarding stunting programs.
Machine		
**Process**		
Planning	Strong coordination led by BAPPEDA among the government agencies in managing stunting-related data to be used by government agencies and village administration; BAPPEDA makes innovations in stunting-related activities by improving stunting-related data; Data publications are done via offline and online media.	Stunting-related data are unavailable at all levels; institutional egocentrism remains among government agencies related to using stunting-related data.
Budgeting	Most government agencies allocate sufficient budgets for stunting prevention programs. A large fund (IDR 6.1 billion) is allocated for executing specific programs; Village administrations allocate sufficient funds for the stunting-related program.	Community Health Centers have no sufficient funds for specific programs; many funds for stunting-related programs are disapproved as they are relocated for Covid-19 measures; villages have a small fund, which is only enough for PMT and for paying the cadres of Integrated Service Stations and KPM.
Organizing	Convergence of stunting prevention programs should be done in a coordinated manner in which BAPPEDA plays a technical coordinator down to the lowest level of government.	Government agencies usually give slow responses to stunting-related programs; coordinating meetings are not followed up, as if the programs are not important; government agencies are not well coordinated regarding sensitive programs for households with stunting cases.
Implementing	Most actors in stunting prevention programs perform their tasks and function optimally; Community Health Centers are committed to and focus on implementing specific interventions.	Cadres still use a conventional meter as a height measurement tool when sweeping children under five years of age; Weighing is done with a steelyard, but the steelyard comes without a sliding weight, as seen in photos; Community Health Centers cover such large areas that it is difficult to monitor all health stations. Midwives are exhausted (due to their workload); Midwives are apprehensive if many stunted children under five years of age are found in their working areas; the disparity between the number of children under five years of age and the number of midwives monitoring them is blamed for being the factor behind the high rate of stunted children in the area; Change of status from locus to non-locus Community Health Centers results in their losing mandate to provide special treatment for stunting prevention in the areas.
Monitoring and Evaluation	Integrated monitoring and evaluation are undertaken in Integrated Service Stations chosen at random by a coordinated team comprised of the Health Office, BAPPEDA, DPMN, and Social Service; unannounced inspections are conducted to determine the true state of affairs at the Integrated Service Stations.	Community Health Center’s staff remain doubtful about the cadres’ performance in measuring and weighing children under five years of age and expectant mothers; Staff of the Community Health Center admit the impossibility of monitoring all the activities run by the Integrated Service Stations in their working area.
**Output**	Most members of society at the stunting locus have been well-exposed to programs, such as clean latrines, nutritious supplementation, PMT (Supplementary Food Provision), KIA book, and JKN card; Sensitive programs prioritize the community’s access to clean water.	

## Discussion

The current study sheds new light on the factors driving and inhibiting the implementation of the stunting reduction acceleration program by conducting a qualitative study at the district level.

Indonesia is firmly committed to reducing stunting by implementing a national strategy for stunting reduction and prevention (Stunting National Strategy). This strategy is based on a multi-sectoral approach through a convergence program at all levels of government. The convergence program has proven to be successful in reducing stunting prevalence [23, 24]. It was found that the program’s success depends on the teams or actors acting as leaders or coordinators to direct or manage all the relevant sectors. Such leadership is important to ensure effective coordination and implementation on the ground and in line with indicators and effective utilization of every opportunity to accelerate actions [25, 26]. The results show that good coordination is one of the driving factors for successfully implementing stunting reduction. District B demonstrates this quality. On the other hand, in District A, the interventions by a government agency, individually or with the related agencies, were not well coordinated and integrated; instead, each agency appeared to run its program separately. In other words, each sector tended to work independently. The lack of cross-sectoral cooperation is reflected at the central level, where each sector focuses on its sectoral priorities [27]. Such circumstances certainly hinder the implementation of multi-sectoral activities. These results are similar to other studies, which find that the lack of collaboration among related sectors can pose a challenge to multi-sectoral implementation.

The major challenge in cross-sectoral coordination is to manage separately the funding orientation, budget control, planning, monitoring, and accountability needed to support collaborative actions [28]. The results show that limited funding has become an inhibiting factor in stunting reduction programs in many government agencies in the failed district. Other studies have also found that insufficient internal funding is a major obstacle to the program’s sustainability [27]. Several programs, such as sanitation, PMT (Supplementary Food Provision), and counseling services, received limited funding. The budget should have been sourced from the village funds. In reality, however, the village allocated an insufficient budget for such a stunting program. Ideally, the village funds can significantly reduce stunting prevalence and support the improvement of essential services and stunting prevention services (by supporting the services of Integrated Service Stations), integrated nutrition counseling, clean water, sanitation, and social protection [29, 30].

Budgeting in District B is different. In this district, most government agencies, including the village administration, allocated specific budgets to stunting programs. In this district, a large sum of the village funds was allocated for stunting-related activities, especially at the Integrated Service Stations. Undoubtedly, stunting prevention programs depend heavily on the availability of a budget to support the implementation of specific and sensitive programs within the community. In this case, it is essential to increase the heads of villages’ awareness of the importance of health programs such that they can produce a policy in which the village funds can be partly allocated to stunting early prevention activities [30].

This study found that the failed district was unable to reduce its stunting rate for the following reasons. One is related to the fact that service providers and program implementers had not performed their duties and functions optimally. Also, the district did not yet have the required innovative program to accelerate stunting reduction and prevention significantly. This is because the geographic location, organization, administration, intervention deliveries, and targeted population affect the overall effectiveness of the intervention. An area may have a dominant factor causing stunting such that simple interventions against this factor can result in a great impact [31]. One of Brazil’s key success factors in reducing stunting is the implementation of multi-sectoral policies. However, this factor does not occur in isolation; instead, it should be supported by programs funded in such a way as to encourage cross-sectoral collaboration across various parties at the local level. The school lunch program is a case in point [32].

It is widely accepted that a mother’s education is associated with stunting [33–35]. In contrast to the failed district, the Community Health Centers in the successful district developed innovative activities for implementing stunting-related activities. For example, the Community Health Centers were highly committed and focused on running classes for expectant mothers and mothers of children under five through innovative activities that drew the mothers’ attention. These classes are critical interventions in breaking the chain of stunting as such activities are intended to improve mothers’ understanding of child development. Nigeria demonstrated this very case, emphasizing maternal education as one of its programs [36, 37]. In contrast, the failed district showed that health services for expectant mothers and mothers with children under the age of five did not function optimally, most likely due to heavy workloads and broad coverage. This shortfall hampered its efforts to reduce stunting.

Furthermore, it could be argued that implementing sensitive programs in District B was optimal. Any stunting reduction program-related activities were accompanied by education, promotion, and socialization to the targets about parenting and nutrition. Meanwhile, sensitive programs did not run optimally in District A for various reasons. These include a lack of funds, inadequate data on stunting, coordination, and human resources.

This study also found that BAPPEDA introduced innovative stunting activities to improve stunting-related data in the successful district. The information was made public and distributed to the sub-districts. Undoubtedly, this innovation is one of the driving factors behind the district’s success in lowering the stunting rate. All government agencies in the district implementing stunting programs up to the village level must have access to stunting data. Its availability enables more effective implementation of activities in households with stunting cases (s). This is similar to the situation in Peru, where the collection of national and subnational data on stunting plays an important role in its stunting reduction programs. Annual data availability is critical for a collaborative approach to program monitoring and adaptation. Furthermore, high-quality data can reveal possible problems in program design [38]. Vietnam, for example, has successfully reduced malnutrition. This accomplishment necessitates the country developing capacity for regional planning and nutrition policy implementation through a data-driven process that can be tailored to local contexts [39].

The primary goal of monitoring and evaluation is to understand what has been accomplished through a project or activity and how well the program has run [40]. The findings in District A showed that the supervisory function is still lacking. This is attributed to a lack of human resources and a budget for monitoring specific community programs. District B had a different situation. In this district, BAPPEDA’s integrated monitoring and evaluation activities enabled proper supervision of stunting program implementation. For instance, the integrated monitoring and evaluations were conducted at the Integrated Service Stations. The inspections were conducted at random and unannounced to determine what obstacles, including human resources and management, were present in the service stations. Based on the findings, it is possible to conclude that good supervision, monitoring, and evaluation of stunting-related programs are responsible for lowering the stunting rate.

## Conclusion

On the one hand, stunting reduction programs are successfully implemented for a variety of reasons. These include the important role of BAPPEDA serving as the coordinator and district government agency in charge of convergence actions related to stunting reduction programs. Others include the high priority given to improving the quality of data related to stunting, significant financial support, high commitment from the Community Health Centers regarding the health of expectant mothers, newborns, and children under the age of five through pertinent classes. The well-executed tasks by the implementing actors in specific and sensitive programs and good supervision also contributed to the achievement. On the other hand, the inhibiting factors include poorly convergent program implementations, restricted finance, inadequate performance of roles and responsibilities by relevant actors, a lack of supervisory function, and a lack of innovative programs that enable program implementations to reach the stunting locus community.

Recommendations for accelerating stunting reduction rest with district government agencies. In this regard, the reduction in stunting prevalence must be used as a performance indicator for government agencies. For the program to be more convergent, improved communication and coordination between government agencies are necessary. Also, to support stunting prevention programs at the village level, assistance is needed to synchronize its planning and budgeting. Furthermore, innovative programs are required to address each district’s primary causes of stunting. As for sensitive and specific nutrition interventions targeting families at risk of stunting, an efficient and focused budget is required.

Planning and procurement of anthropometric measuring instruments are expected to meet the standards. In addition, routine antenatal checks during pregnancy are necessary, with the provision of Fe tablets for expectant mothers (at least 90 tablets) and adolescent girls. Finally, it is expected that the actors implementing stunting activities can build a mindset among the community that perceives stunting as a health problem. Such a conception should be spread through continuous KIE (Communication, Information, Education) campaigns at all levels, including the village level, using a variety of methods and channels.

## Supporting information

S1 File (PDF)

S2 File (PDF)
