# Supplementary material for: Factors driving and inhibiting stunting reduction acceleration programs at district level: A qualitative study in West Sumatra
Source: PLoS One. 2023 Mar 31;18(3):e0283739. doi: 10.1371/journal.pone.0283739 (PMC10065257; doi:10.1371/journal.pone.0283739)
Supplement: S1 File — (PDF) [file pone.0283739.s001.pdf]

KEMENTERIAN KESEHATAN REPUBLIK INDONESIA

SSGI
Studi Status Gizi Indonesia

Buku Saku

Hasil Studi Status Gizi Indonesia (SSGI) Tingkat Nasional, Provinsi, dan Kabupaten/Kota Tahun 2021

Senin, 27 Desember 2021

DAFTAR ISI

Latar Belakang	6
Tujuan	7
Metodologi dan Sampling SSGI 2021	8
Kerangka konsep dan variable	9
Prevalensi status gizi balita nasional tahun 2019-2021	10
Prevalensi balita stunted (tinggi badan menurut umur), SSGI 2021	11
Prevalensi balita wasted (berat badan menurut tinggi badan), SSGI 2021	12
Prevalensi balita underweight (berat badan menurut umur), SSGI 2021	13
Sebaran Status Gizi Balita Berdasarkan Komposit Tinggi Badan Menurut Umur Dan Berat Badan Menurut Tinggi Badan Di Indonesia, SSGI 2021	14
Prevalensi Balita *Stunted* (Tinggi Badan Menurut Umur), Riskesdas 2007, 2013 Dan 2018, SSGBI 2019, SSGI 2021	15
Prevalensi Balita *Stunted* (Tinggi Badan Menurut Umur) Berdasarkan Provinsi, SSGI 2021	16
Prevalensi Balita *Wasted* (Berat Badan Menurut Tinggi Badan) Berdasarkan Provinsi, SSGI 2021	17

KEMENTERIAN KESEHATAN REPUBLIK INDONESIA

SSGI Studi Status Gizi Indonesia

Prevalensi Balita *Underweight* (Berat Badan Menurut Umur) Berdasarkan Provinsi, Ssgi 2021 18

Prevalensi Balita Overweight (Berat Badan Menurut Tinggi Badan) Berdasarkan Provinsi, Ssgi 2021 19

Prevalensi Balita *Stunted* Menurut Provinsi, Ssgbi 2019 Dan Ssgi 2021 20

Masalah Gizi Pada Baduta Dan Balita Di Indonesia, Ssgi 2021 21

Kontribusi *Stunted* Menurut Kelompok Umur Terhadap Total *Stunted* Di Indonesia, SSGI 2021 22

Kontribusi *Wasted* Menurut Kelompok Umur Terhadap Total *Wasted* Di Indonesia, SSGI 2021 23

Kontribusi *Underweight* Menurut Kelompok Umur Terhadap Total *Underweight* Di Indonesia, SSGI 2021 24

Tren & Target Penurunan Stunting 2020-2024 25

Gambaran Determinan Masalah Gizi Di Indonesia, Riskesdas 2018 Dan SSGI 2021 (1) 26

Gambaran Determinan Masalah Gizi Di Indonesia, Riskesdas 2018 Dan SSGI 2021 (2) 27

Gambaran Determinan Masalah Gizi Di Indonesia, Riskesdas 2018 Dan SSGI 2021 (3) 28

Gambaran Determinan Masalah Gizi Di Indonesia, Riskesdas 2018 Dan SSGI 2021 (4) 29

Proporsi Ibu Hamil Yang Pernah Mendapatkan Tablet Tambah Darah Menurut Provinsi, SSGI 2021 30

Proporsi Wanita Hamil Usia 10-54 Tahun Yang Bersalin Di Fasilitas Kesehatan Menurut Provinsi, SSGI 2021 31

Proporsi Wanita Usia Subur/Pasangan Yang Menggunakan Kb Modern Menurut Provinsi, SSGI 2021 32

KEMENTERIAN KESEHATAN REPUBLIK INDONESIA

SSGI
Studi Status Gizi Indonesia

Proporsi Balita Dengan Berat Badan Lahir< 2500 Gram Menurut Provinsi, SSGI 2021	33
Proporsi Balita Dengan Panjang Badan Lahir <48 Cm Menurut Provinsi, SSGI 2021	34
Proporsi Balita Yang Mendapatkan Inisiasi Menyusu Dini Menurut Provinsi, SSGI 2021	35
Proporsi Bayi Usia 0-5 Bulan Mendapatkan Asi Eksklusif Menurut Provinsi, SSGI 2021	36
Proporsi Bayi Usia 6-23 Bulan Mendapatkan Asi Eksklusif Menurut Provinsi, SSGI 2021	37
Proporsi Baduta Saat Pertama Kali Diberikan Mpasi Pada Usia ≥ 6 Bulan Menurut Provinsi, SSGI 2021	38
Proporsi Anak Usia 0-23 Bulan Mengonsumsi Makanan Beragam Menurut Provinsi, SSGI 2021	39
Proporsi Balita Yang Memiliki Buku KIA, SSGI 2021	40
Proporsi Balita Yang Melakukan Penimbangan Berat Badan Sesuai Standar (≥ 8x Setahun), SSGI 2021	41
Proporsi Balita Yang Melakukan Pengukuran Tinggi Badan Sesuai Standar (≥ 2x Setahun), SSGI 2021	42
Proporsi Anak 12-23 Bulan Yang Mendapatkan Imunisasi Dasar Lengkap, SSGI 2021	43
Proporsi Anak Usia 6-59 Bulan Mendapatkan Vitamin A Menurut Provinsi, SSGI 2021	44
Proporsi Baduta Yang Masih Disusui Menurut Provinsi, SSGI 2021	45
Proporsi Balita Yang Menderita Ispa Berdasarkan Diagnosis/Gejala Menurut Provinsi, SSGI 2021	46

Proporsi Balita Yang Menderita Pneumonia Berdasarkan Diagnosis/Gejala Menurut Provinsi, SSGI 2021 47
Proporsi Balita Yang Menderita Diare Berdasarkan Diagnosis/Gejala Menurut Provinsi, SSGI 2021 48
Proporsi Balita Yang Menderita Kecacingan Berdasarkan Diagnosis/Gejala Menurut Provinsi, SSGI 2021 49
Proporsi Balita Yang Menderita Campak Berdasarkan Diagnosis/Gejala Menurut Provinsi, SSGI 2021 50
Proporsi Anggota Rumah Tangga Yang Memiliki Jaminan Pelayanan Kesehatan, SSGI 2021 51
Proporsi Balita Sakit Yang Berobat Ke Fasilitas Pelayanan Kesehatan, SSGI 2021 52
Proporsi Rumah Tangga Yang Memiliki Akses Sarana Air Minum Layak Menurut Provinsi, SSGI 2021 53
Proporsi Rumah Tangga Yang Memiliki Akses Sanitasi Layak Menurut Provinsi, SSGI 2021 54
Presentase Balita Stunted Menurut Kuintil Aset Kepemilikan Dan Provinsi, SSGI 2021 55
KESIMPULAN 56
REKOMENDASI 57
Definisi Operasional Indikator 58
Angka Stunted Tingkat Kabupaten Dan Kota 63
Angka Wasted Tingkat Kabupaten Dan Kota 98
Angka Underweight Tingkat Kabupaten Dan Kota 133

LATAR BELAKANG

1. Perpres nomor: 72 tahun 2021 tentang Percepatan Penurunan *Stunting*. **Kemenkes mempunyai tanggung jawab publikasi angka *stunting* kabupaten/kota setiap tahun** (Lampiran Perpres, hal 44)
2. Pemenuhan laporan ***Disbursement Link Indicator* (DLI)** Program *Investment in Nutrition and Early Years* (INEY) World Bank yang dikoordinasikan oleh Kantor Wakil Presiden, salah satunya publikasi angka stunting kabupaten/kota setiap tahun.
3. Dasar penetapan **Dana Insentif Daerah (DID) oleh Kemenkeu, sehingga diperlukan penyediaan angka tingkat** kabupaten/kota setiap tahun.
4. Dasar evaluasi dan penilaian kemajuan intervensi konvergensi (spesifik dan sensitif) dari berbagai kementerian dan lembaga, baik di Pusat maupun Daerah (**Aksi Delapan Konvergensi**)

TUJUAN :

1. Memperoleh besaran masalah status gizi balita (*stunted, wasted* dan *underweight*) tingkat Nasional, Provinsi, Kabupaten/Kota

2. Memperoleh faktor determinan terjadinya *stunted, wasted* dan *underweight* di Indonesia

KEMENTERIAN KESEHATAN REPUBLIK INDONESIA

SSGI

METODOLOGI DAN SAMPLING SSGI 2021

- Desain penelitian : Stratified two stage sampling
- Populasi : Rumah Tangga Balita di Indonesia
- Sampel : 153.228 Rumah Tangga Balita di 14.889 Blok Sensus, Susenas Maret 2021

Tempat : Di 34 Provinsi dan 514 Kabupaten/Kota

Waktu : Penelitian dilaksanakan mulai bulan Januari- Desember 2021

KEMENTERIAN KESEHATAN REPUBLIK INDONESIA

SSGI
Studi Status Gizi Indonesia

KERANGKA KONSEP

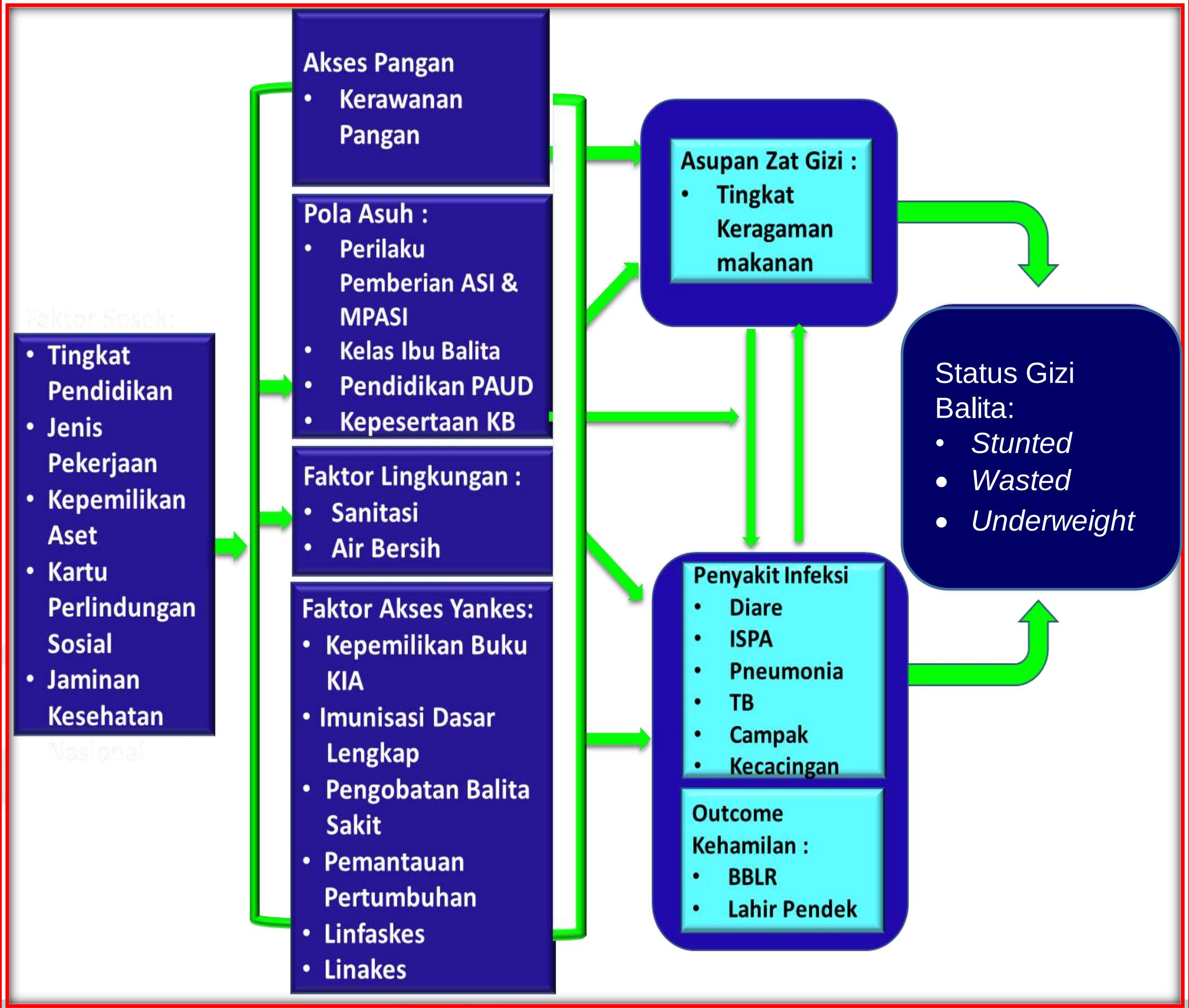

VARIABEL

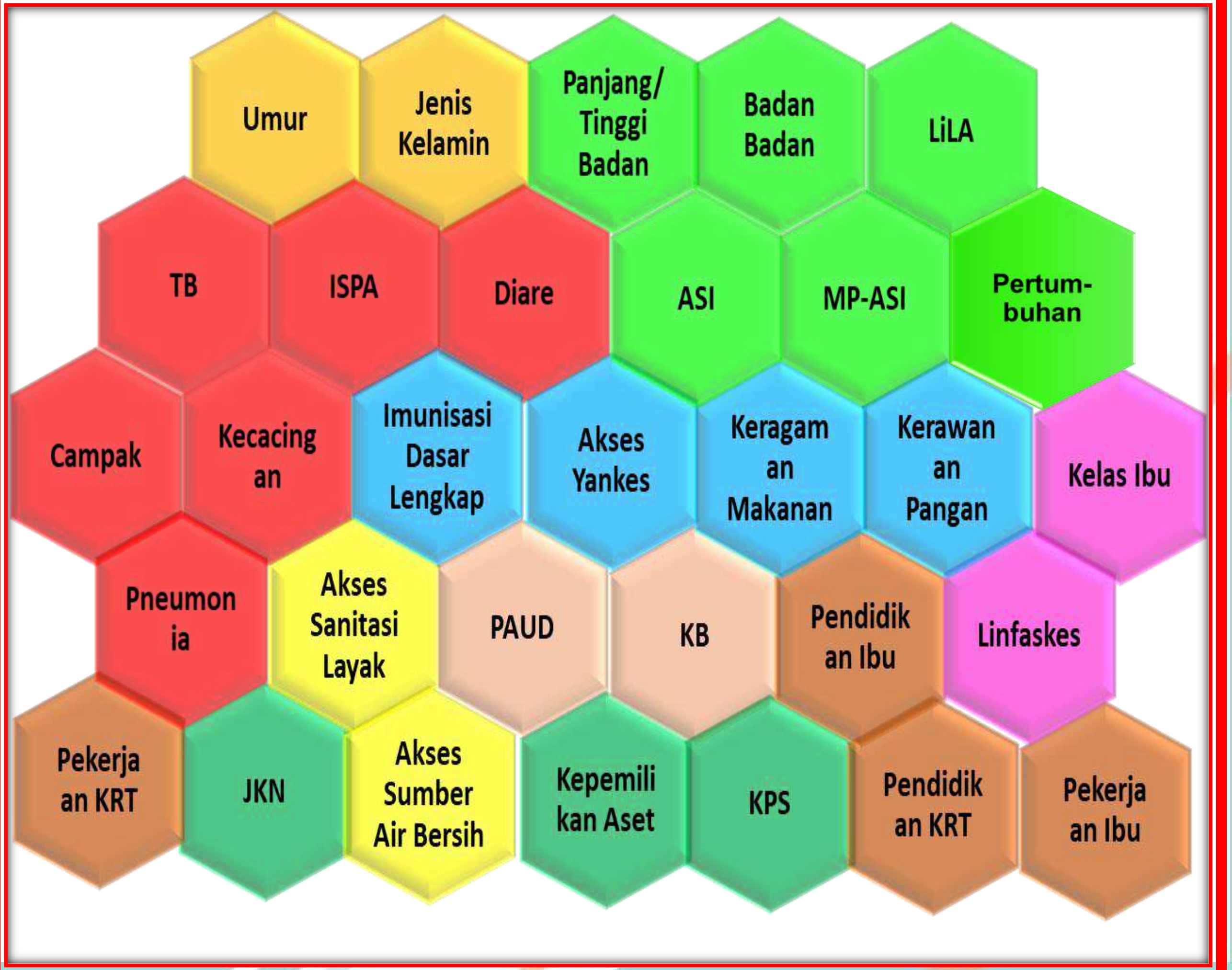

KEMENTERIAN KESEHATAN REPUBLIK INDONESIA

SSGI
Studi Status Gizi Indonesia

PREVALENSI STATUS GIZI BALITA NASIONAL TAHUN 2019-2021

No	Status Gizi Balita	SSGBI 2019	Prediksi 2020	SSGI 2021
1	*Stunted*	**27,7%** (27,2-28,1)	26,9% (23,8 – 29,9)	**24,4%** (23,9-24,9)
2	*Wasted*	7,4% (7,2 – 7,7)	-	7,1% (6,8-7,3)
3	*Underweight*	16,3% (15,9-16,7)	-	17,0% (16,6-17,4)

KEMENTERIAN KESEHATAN REPUBLIK INDONESIA

SSGI Studi Status Gizi Indonesia

PREVALENSI BALITA STUNTED (TINGGI BADAN MENURUT UMUR), SSGI 2021

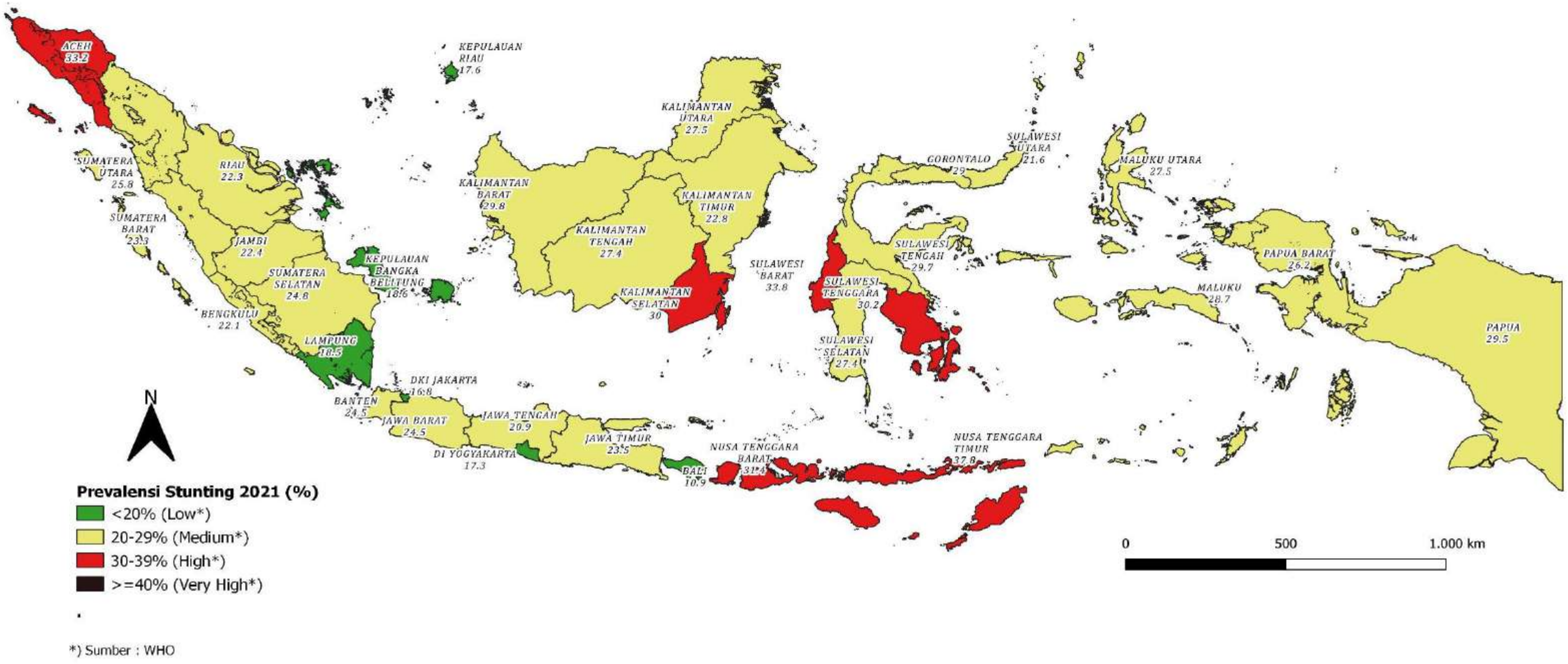

KEMENTERIAN KESEHATAN REPUBLIK INDONESIA

SSGI
Studi Status Gizi Indonesia

PREVALENSI BALITA WASTED (BERAT BADAN MENURUT TINGGI BADAN), SSGI 2021

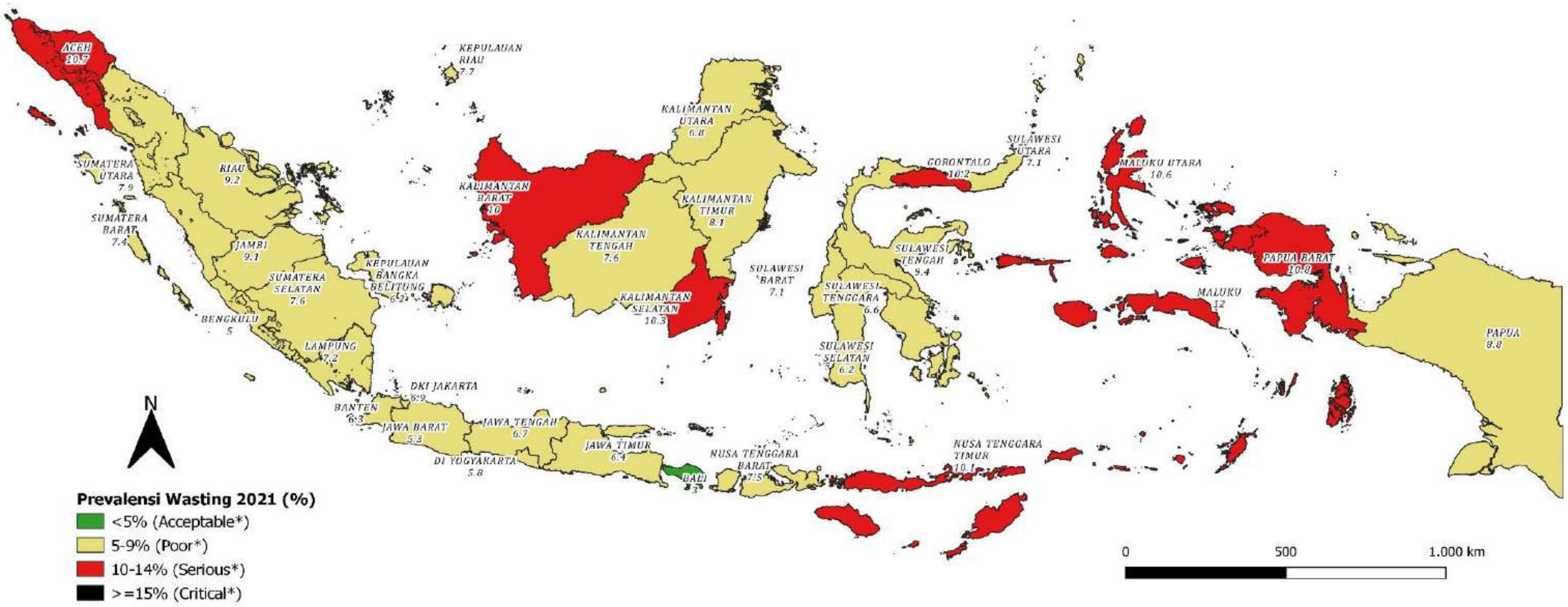

*) Sumber : WHO

KEMENTERIAN KESEHATAN REPUBLIK INDONESIA

PREVALENSI BALITA UNDERWEIGHT (BERAT BADAN MENURUT UMUR), SSGI 2021

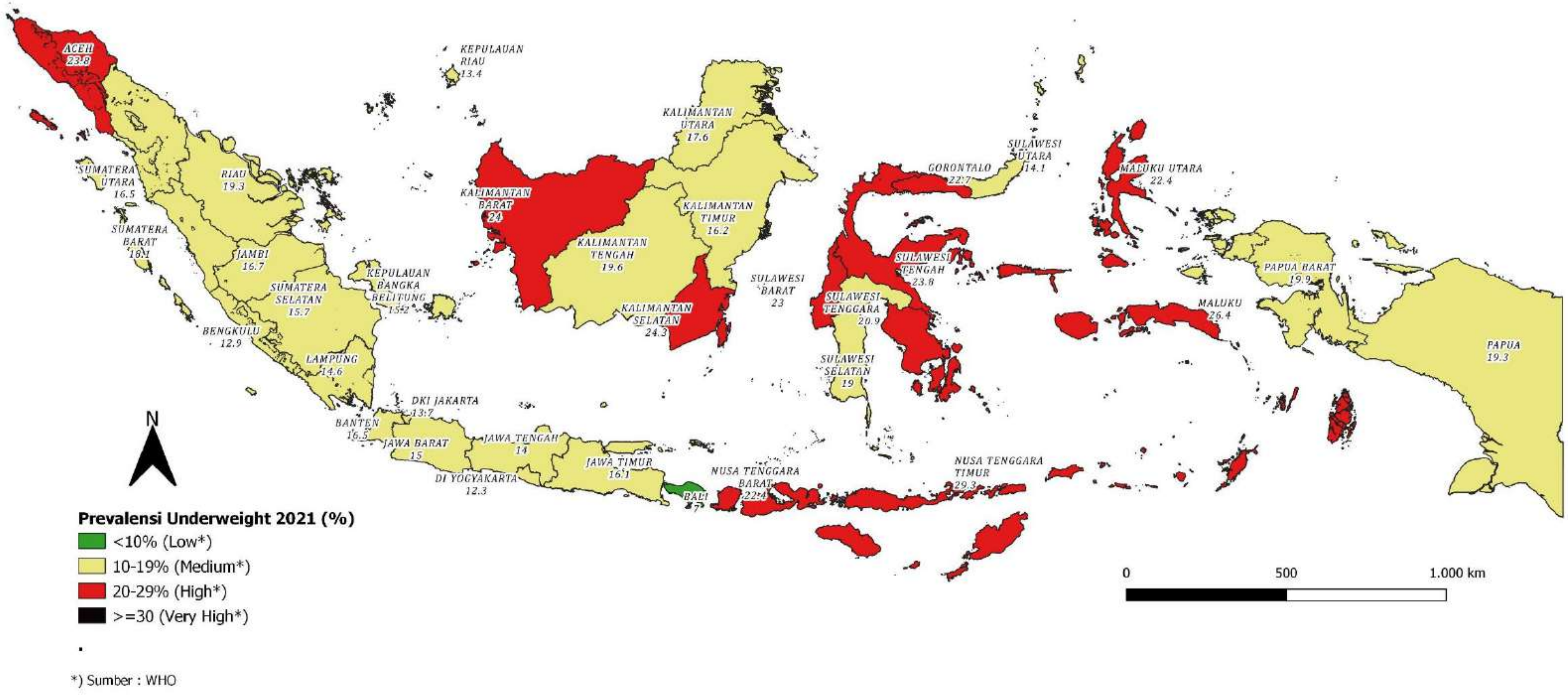

SEBARAN STATUS GIZI BALITA BERDASARKAN KOMPOSIT TINGGI BADAN MENURUT UMUR DAN BERAT BADAN MENURUT TINGGI BADAN DI INDONESIA, SSGI 2021

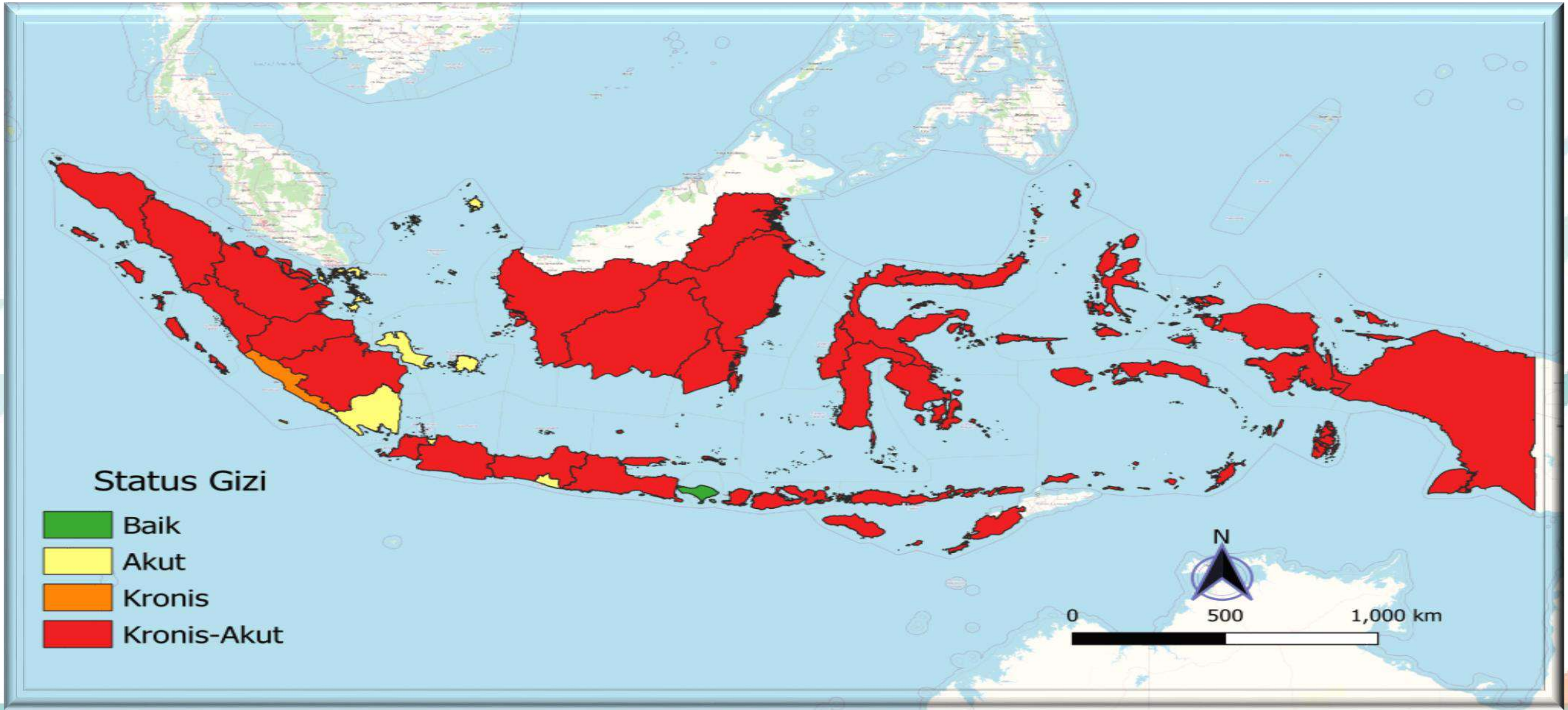

KEMENTERIAN KESEHATAN REPUBLIK INDONESIA

SSGI

PREVALENSI BALITA STUNTED (TINGGI BADAN MENURUT UMUR), RISKESDAS 2007, 2013 DAN 2018, SSGBI 2019, SSGI 2021

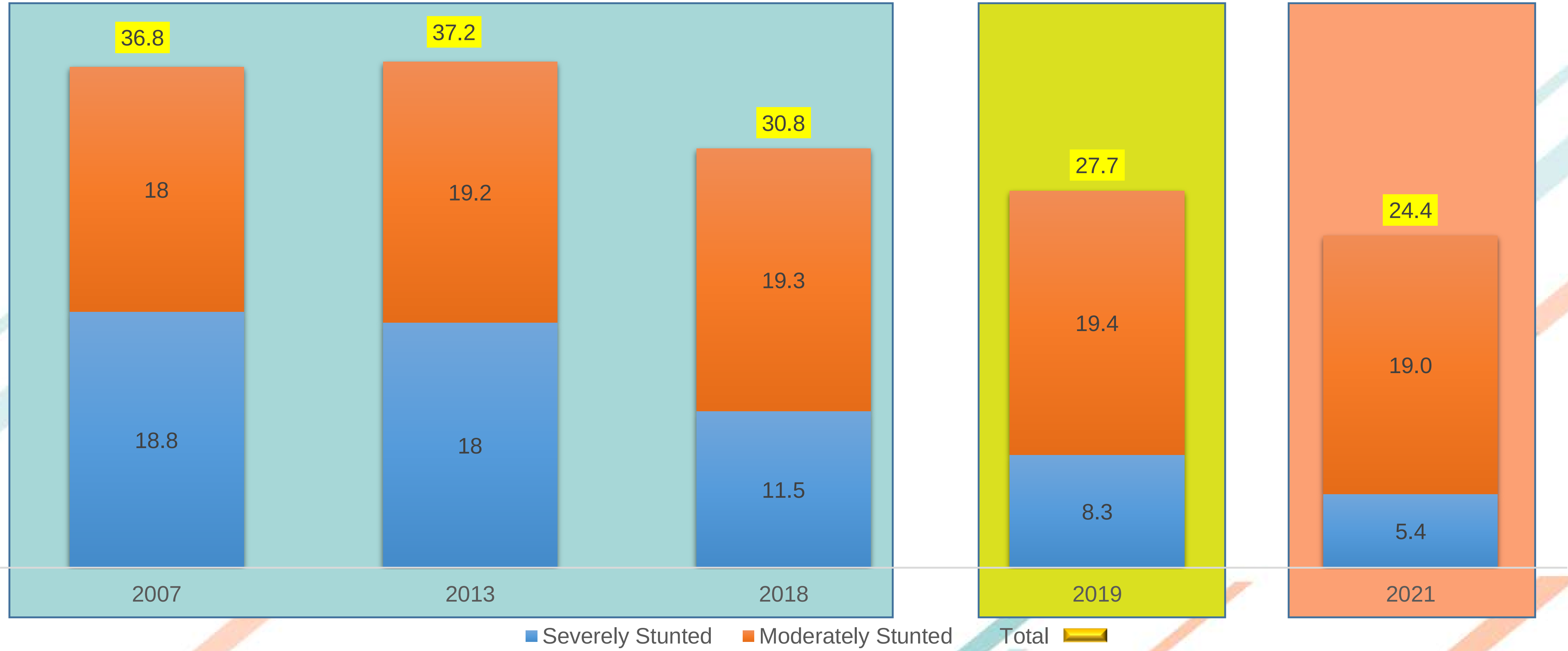

Z-Score<-3 SD **-3 SD< Z-Score< -2SD** **Z-Score<-2 SD**

KEMENTERIAN KESEHATAN REPUBLIK INDONESIA

SSGI

PREVALENSI BALITA STUNTED (TINGGI BADAN MENURUT UMUR) BERDASARKAN PROVINSI, SSGI 2021

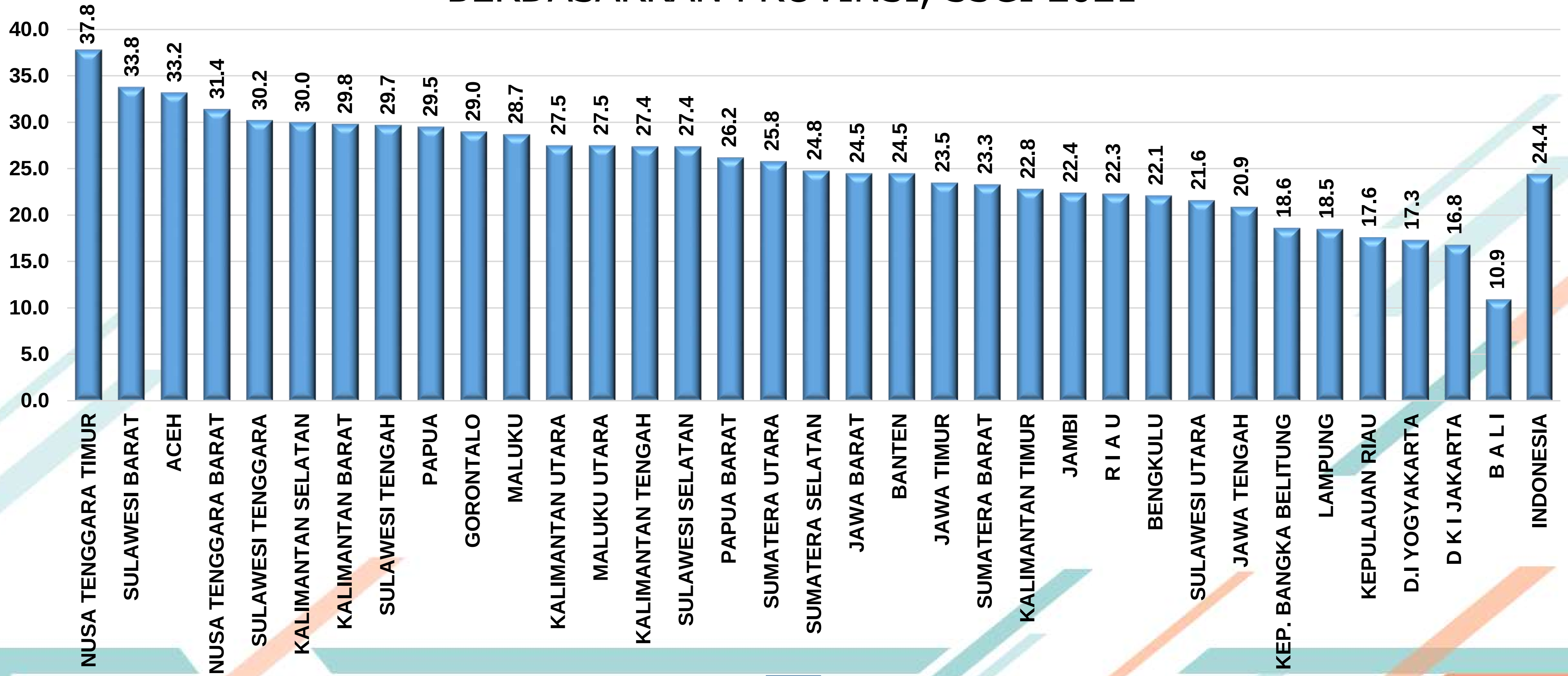

KEMENTERIAN KESEHATAN REPUBLIK INDONESIA

SSGI

PREVALENSI BALITA WASTED (BERAT BADAN MENURUT TINGGI BADAN) BERDASARKAN PROVINSI, SSGI 2021

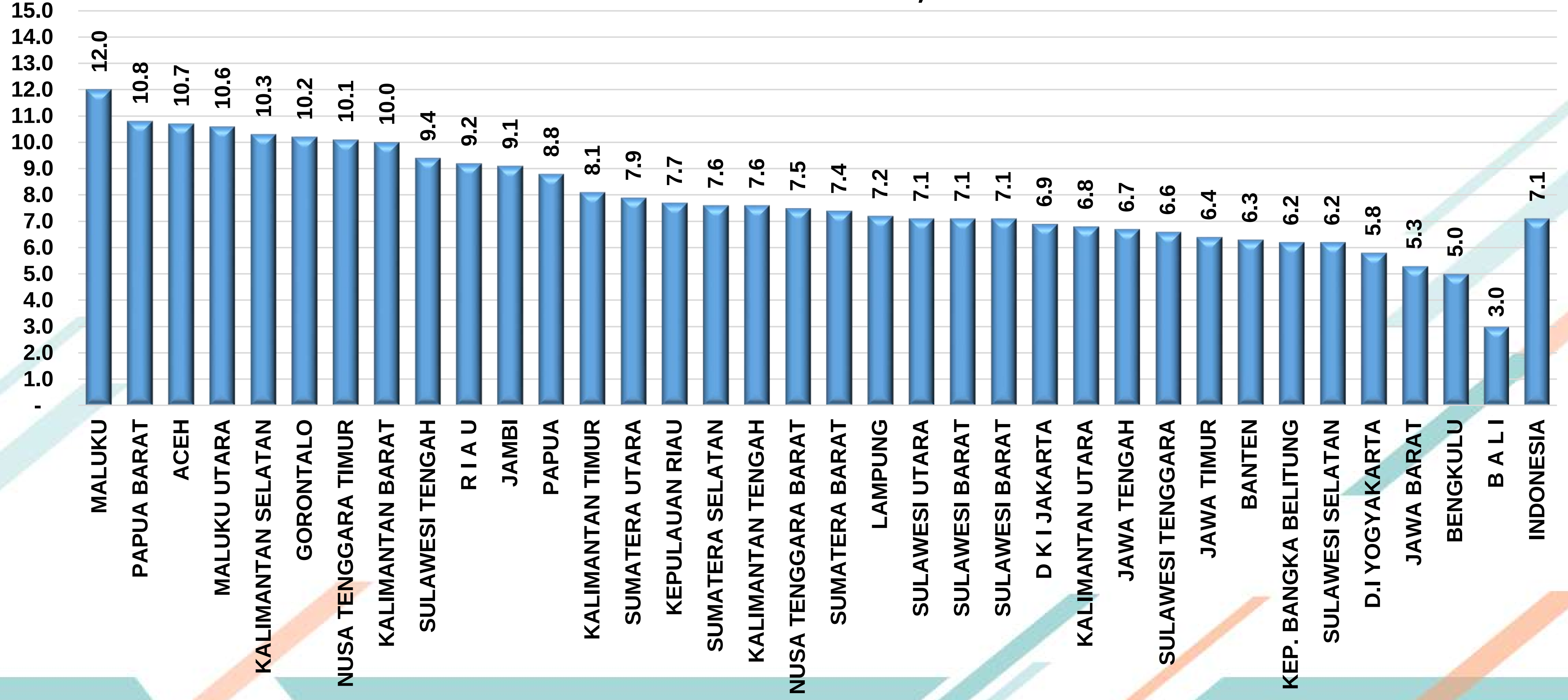

KEMENTERIAN KESEHATAN REPUBLIK INDONESIA

SSGI

PREVALENSI BALITA UNDERWEIGHT (BERAT BADAN MENURUT UMUR) BERDASARKAN PROVINSI, SSGI 2021

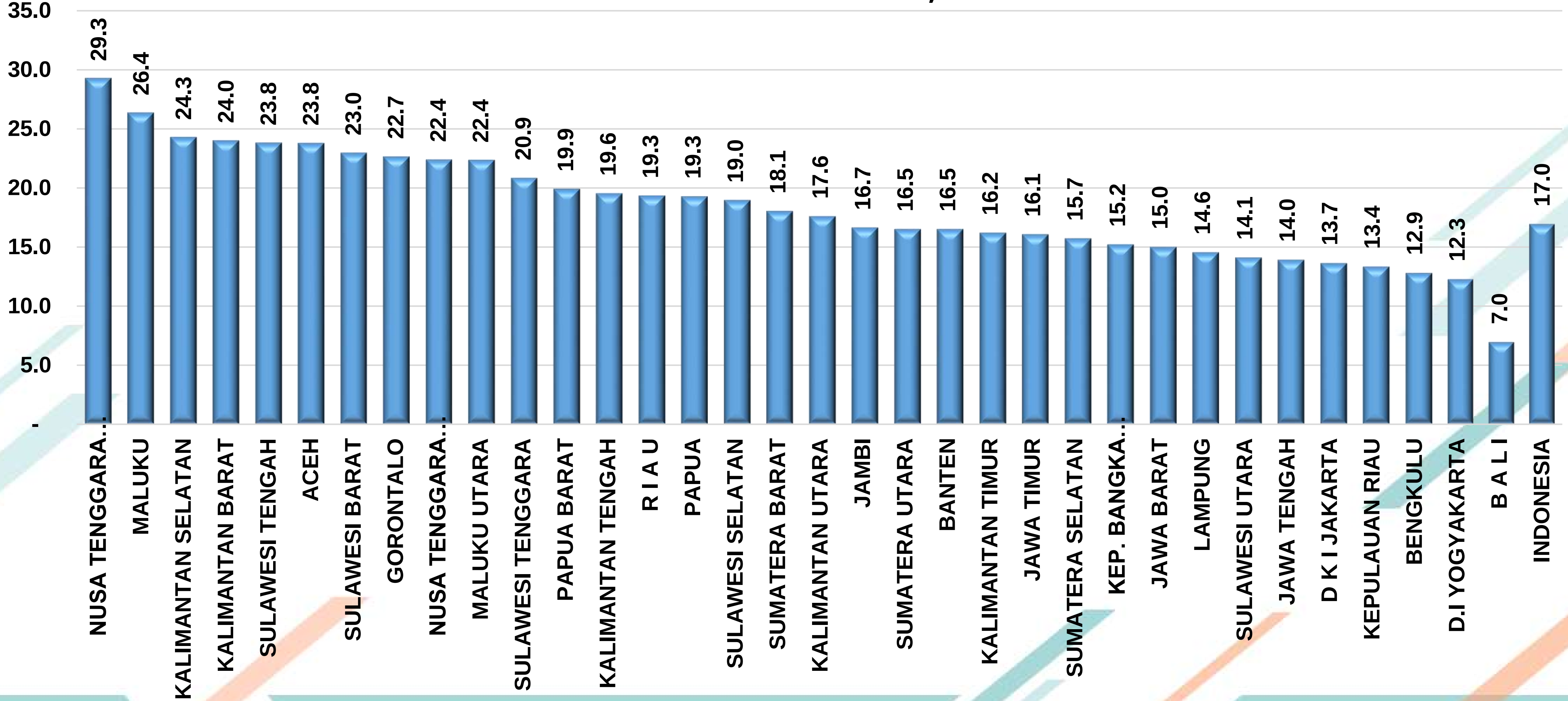

KEMENTERIAN KESEHATAN REPUBLIK INDONESIA

SSGI

PREVALENSI BALITA OVERWEIGHT (BERAT BADAN MENURUT TINGGI BADAN) BERDASARKAN PROVINSI, SSGI 2021

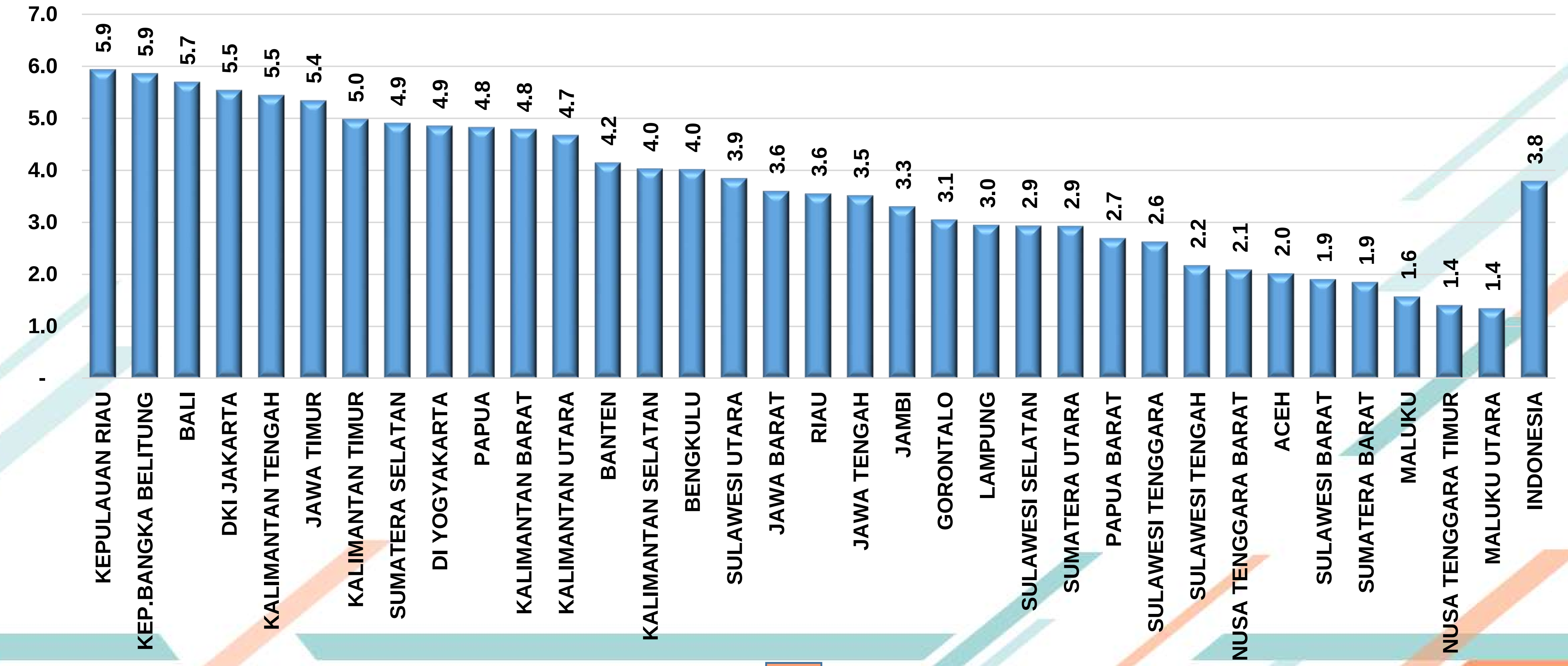

PREVALENSI BALITA STUNTED MENURUT PROVINSI, SSGBI 2019 DAN SSGI 2021

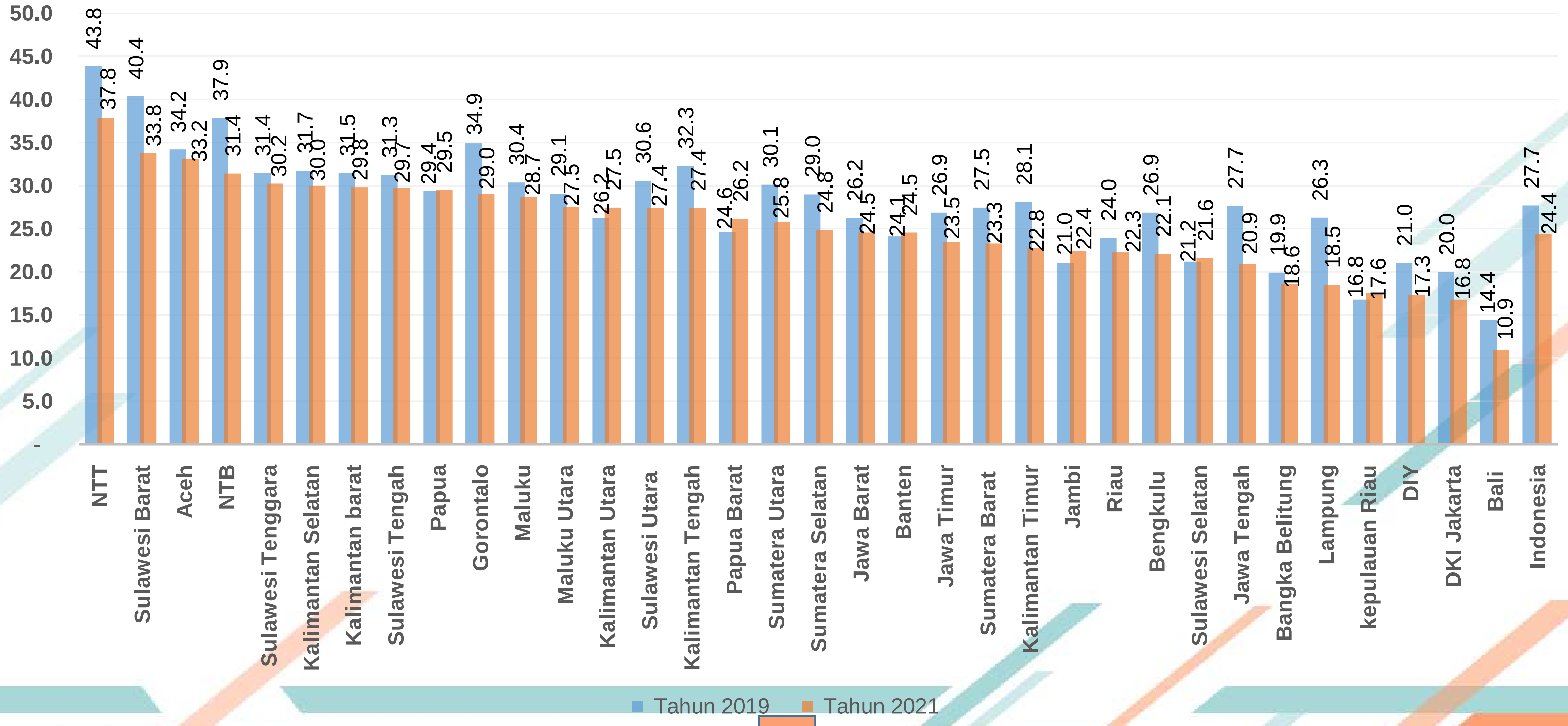

MASALAH GIZI PADA BADUTA DAN BALITA DI INDONESIA, SSGI 2021

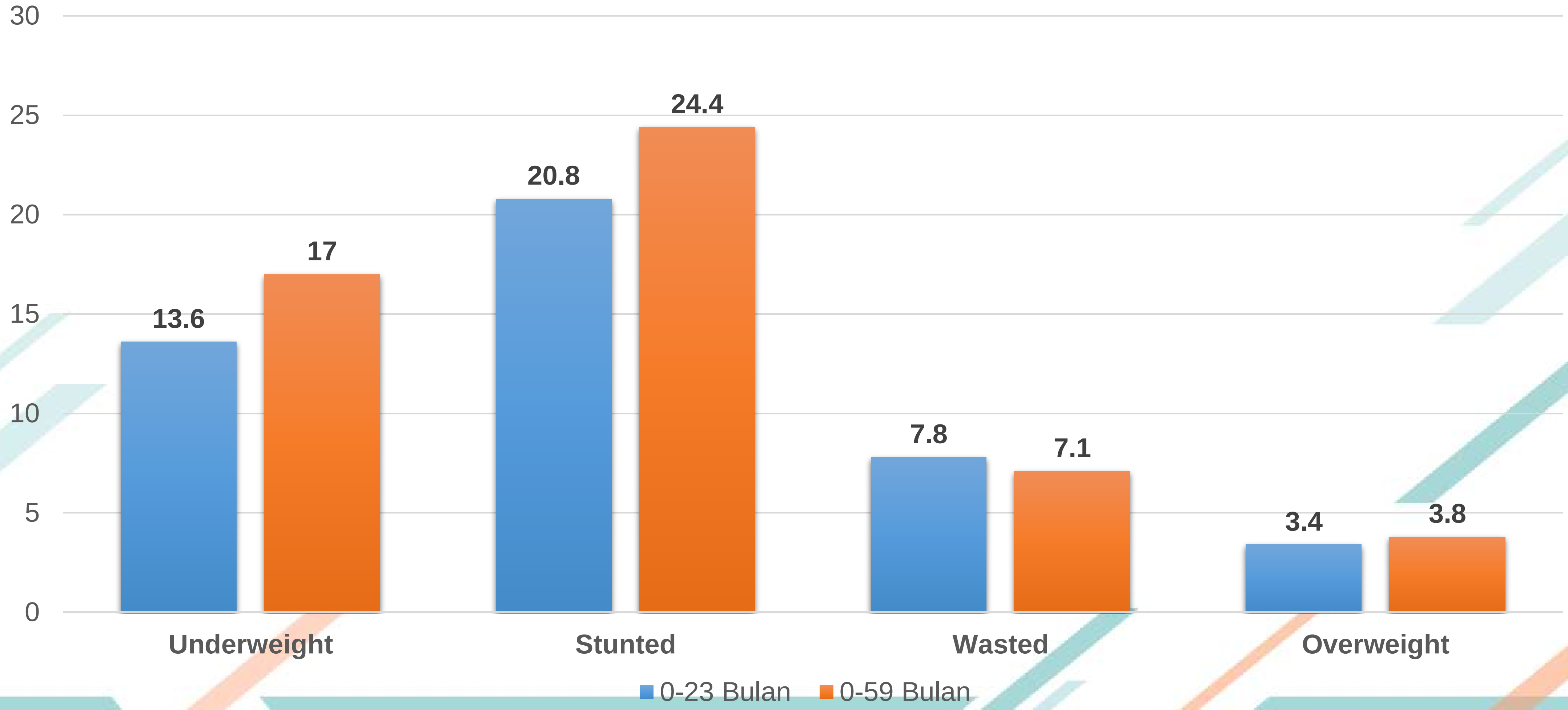

DISTRIBUSI STUNTED MENURUT KELOMPOK UMUR DI INDONESIA, SSGI 2021

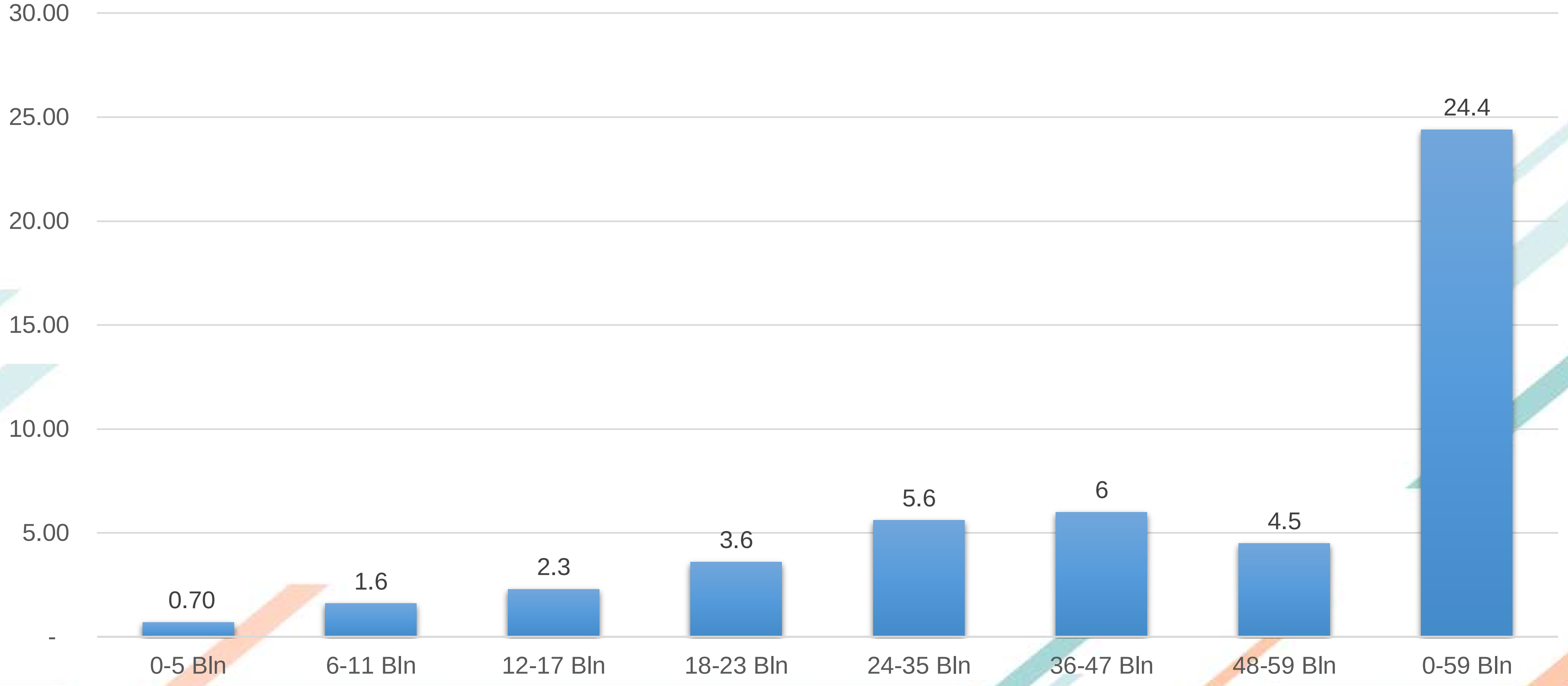

KEMENTERIAN KESEHATAN REPUBLIK INDONESIA

SSGI Studi Status Gizi Indonesia

DISTRIBUSI WASTED MENURUT KELOMPOK UMUR DI INDONESIA, SSGI 2021

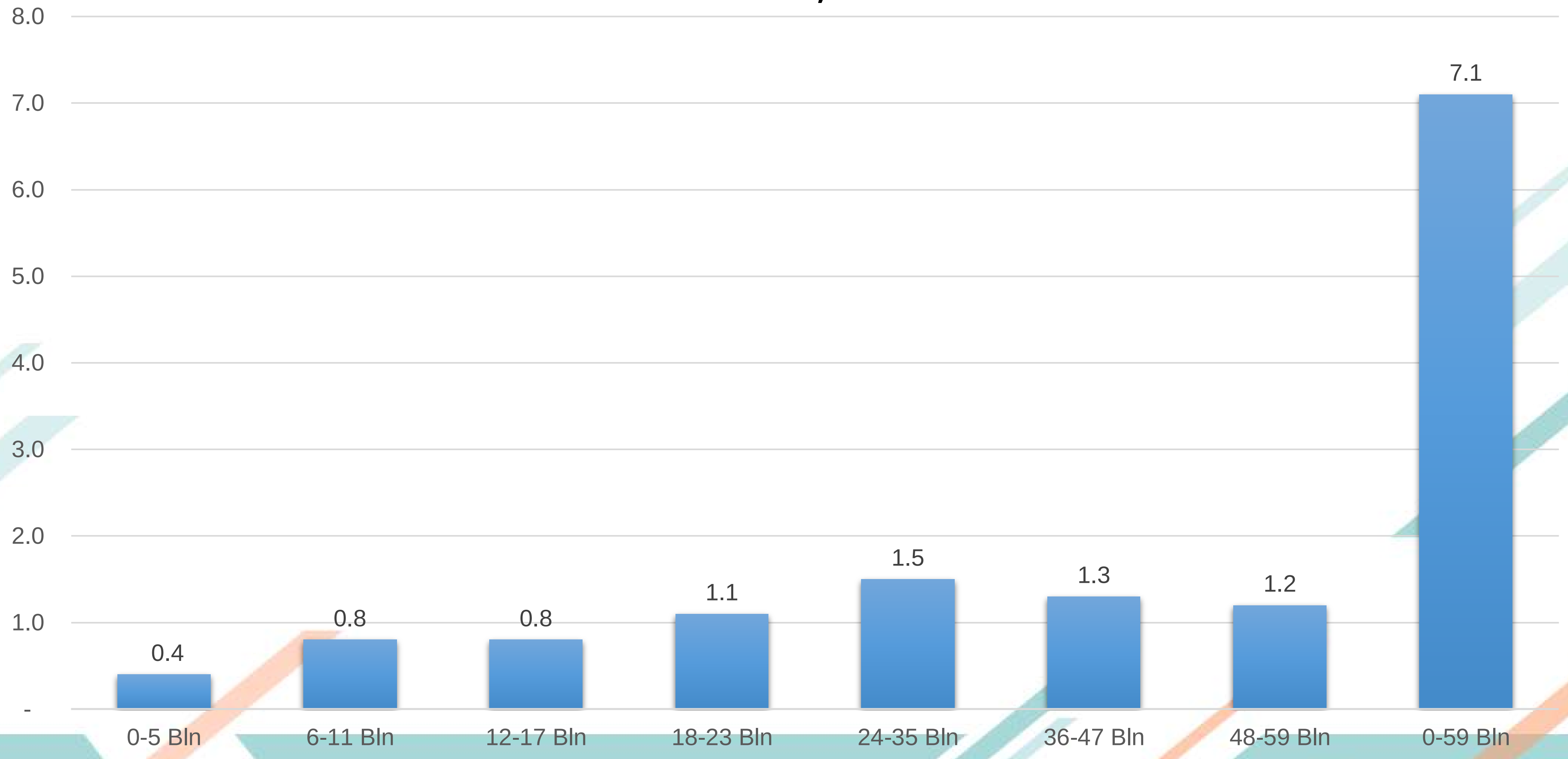

DISTRIBUSI UNDERWEIGHT MENURUT KELOMPOK UMUR DI INDONESIA, SSGI 2021

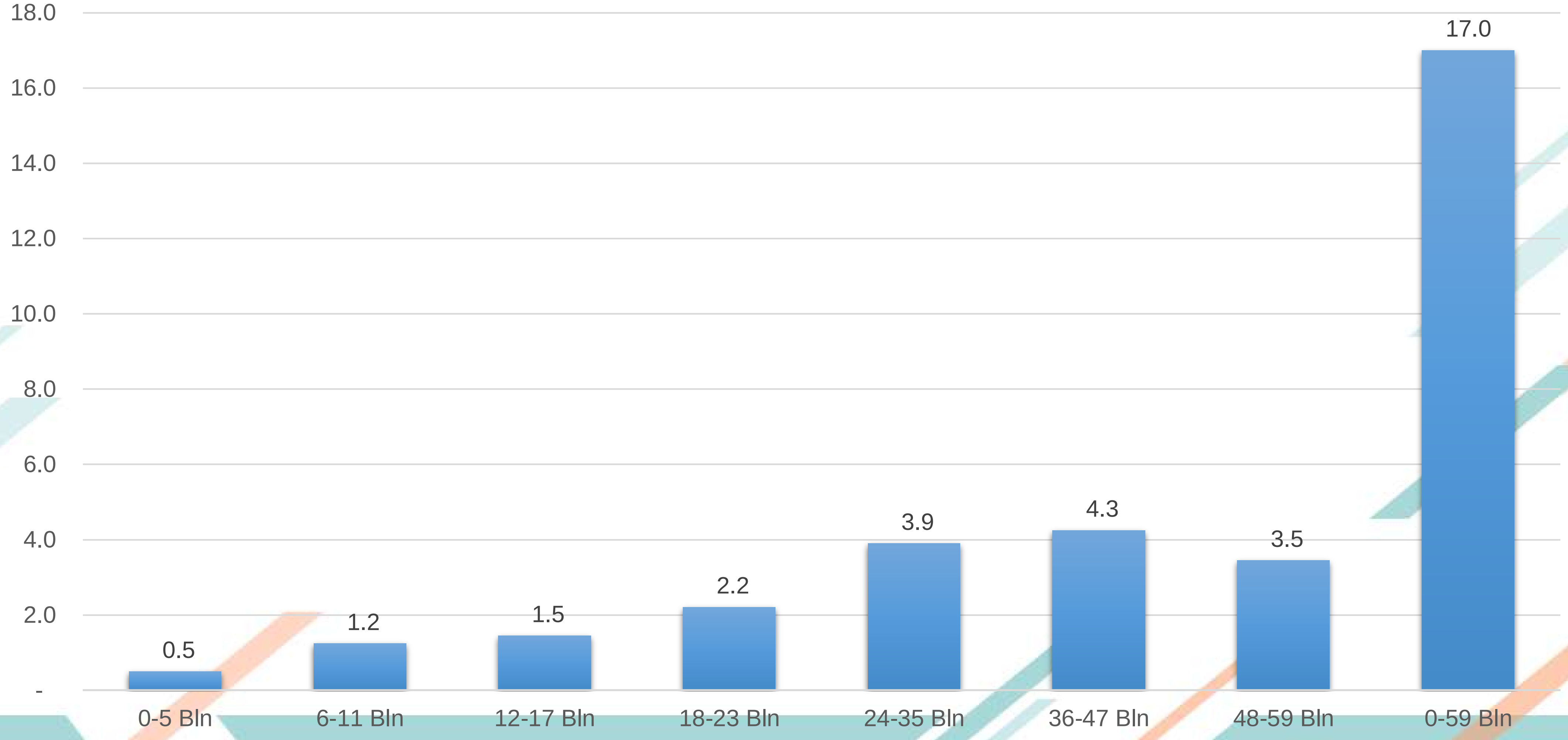

TREN & TARGET PENURUNAN STUNTING 2020-2024

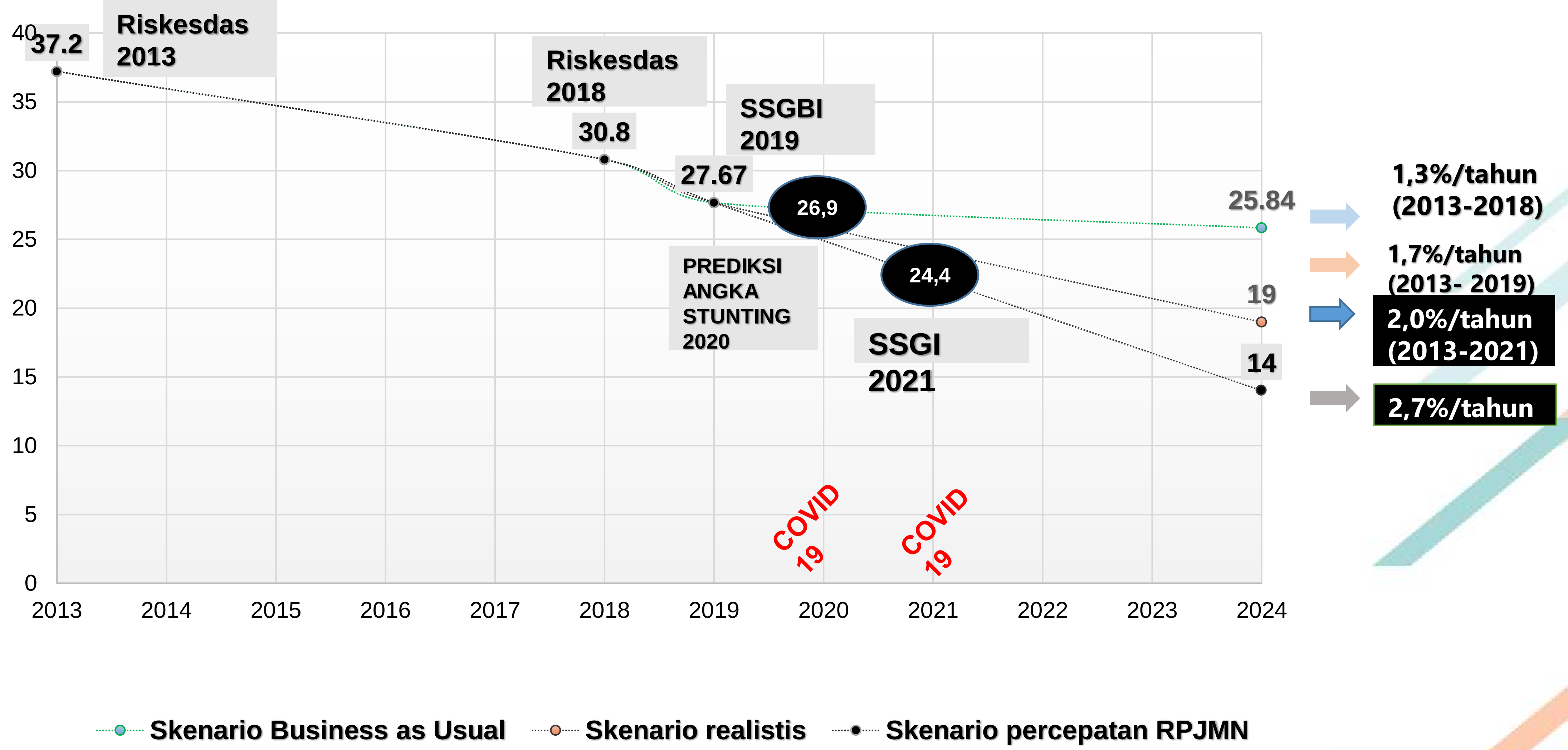

Sumber : Bappenas 2020, Kemenkes, 2021

KEMENTERIAN KESEHATAN REPUBLIK INDONESIA

SSGI
Studi Status Gizi Indonesia

GAMBARAN DETERMINAN MASALAH GIZI DI INDONESIA, RISKESDAS 2018 DAN SSGI 2021 (1)

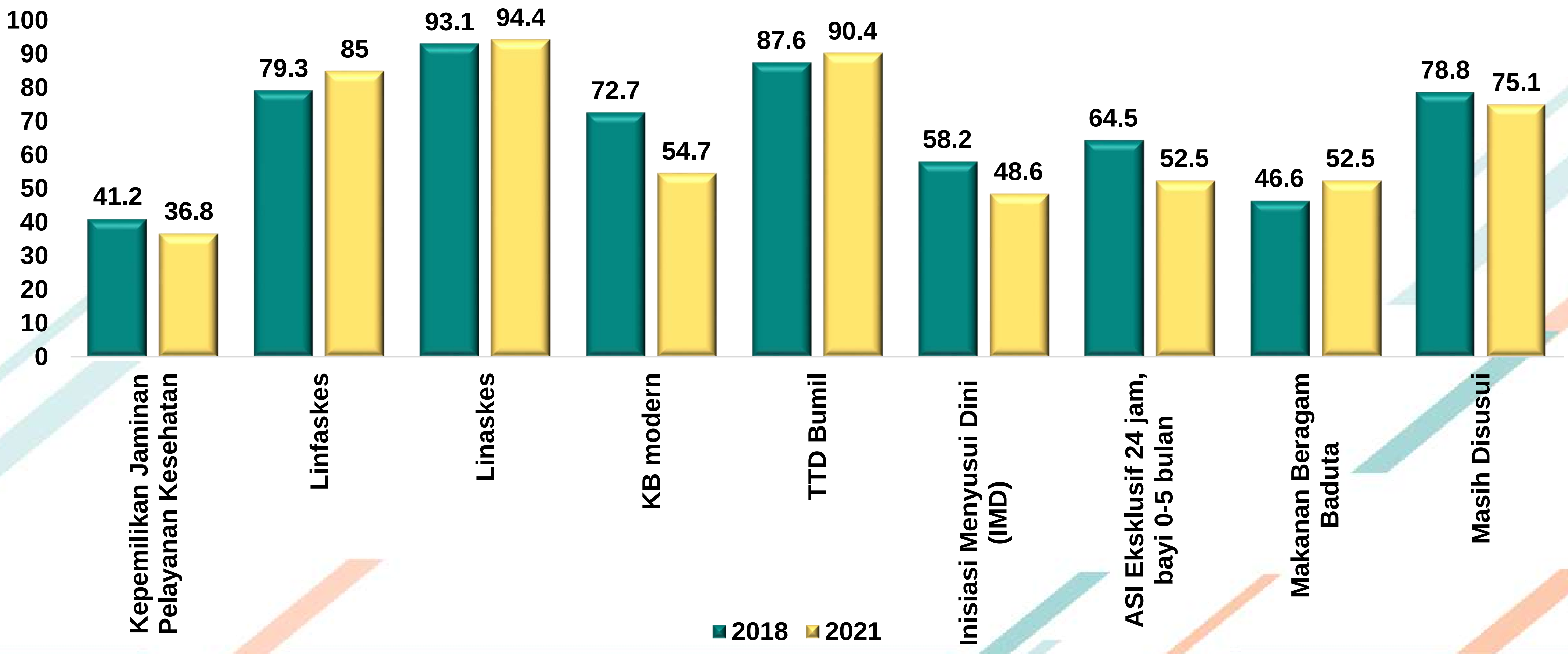

GAMBARAN DETERMINAN MASALAH GIZI DI INDONESIA, RISKESDAS 2018 DAN SSGI 2021 (2)

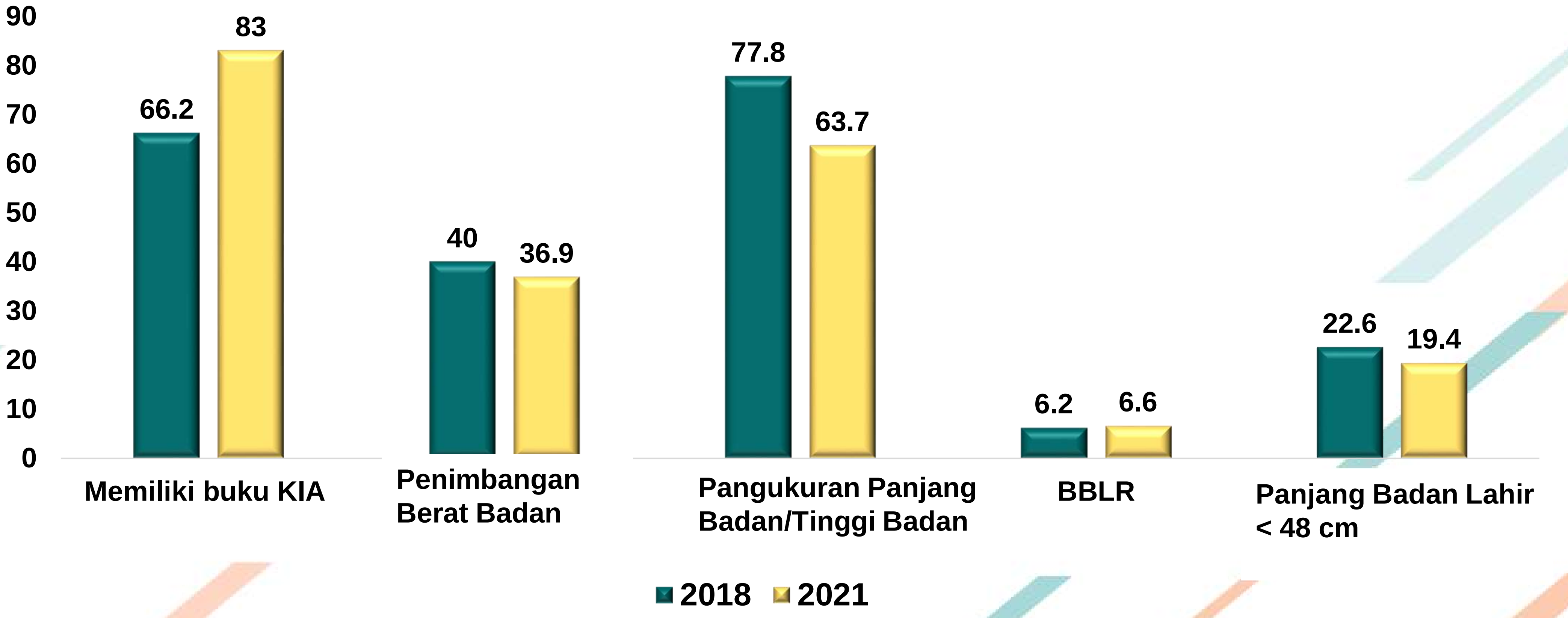

KEMENTERIAN KESEHATAN REPUBLIK INDONESIA

SSGI
Studi Status Gizi Indonesia

GAMBARAN DETERMINAN MASALAH GIZI DI INDONESIA, RISKESDAS 2018 DAN SSGI 2021 (3)

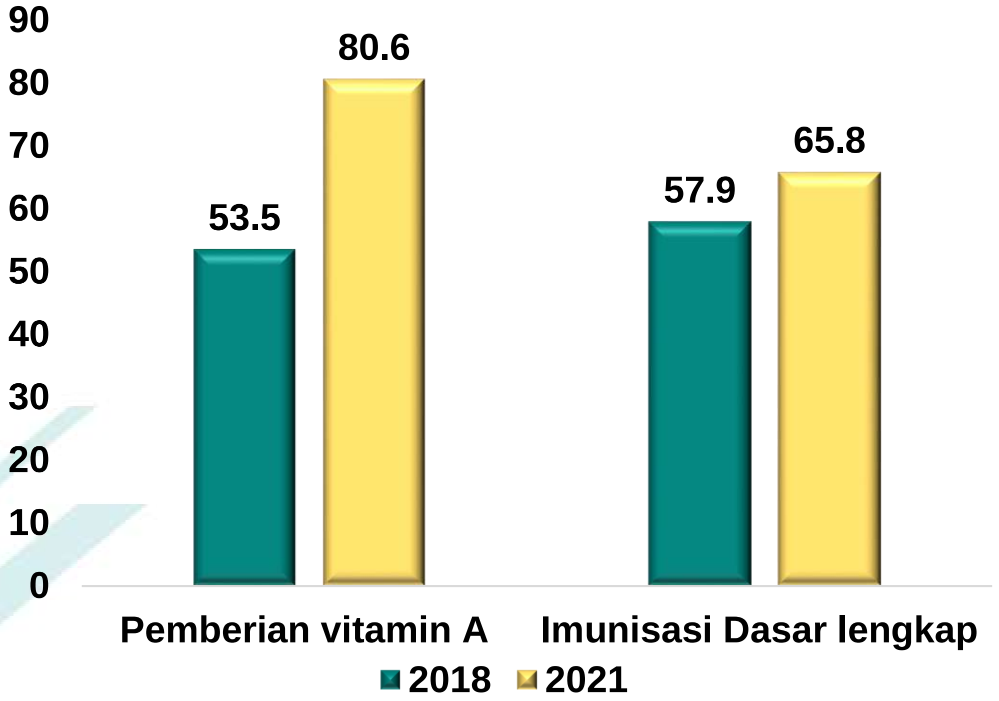

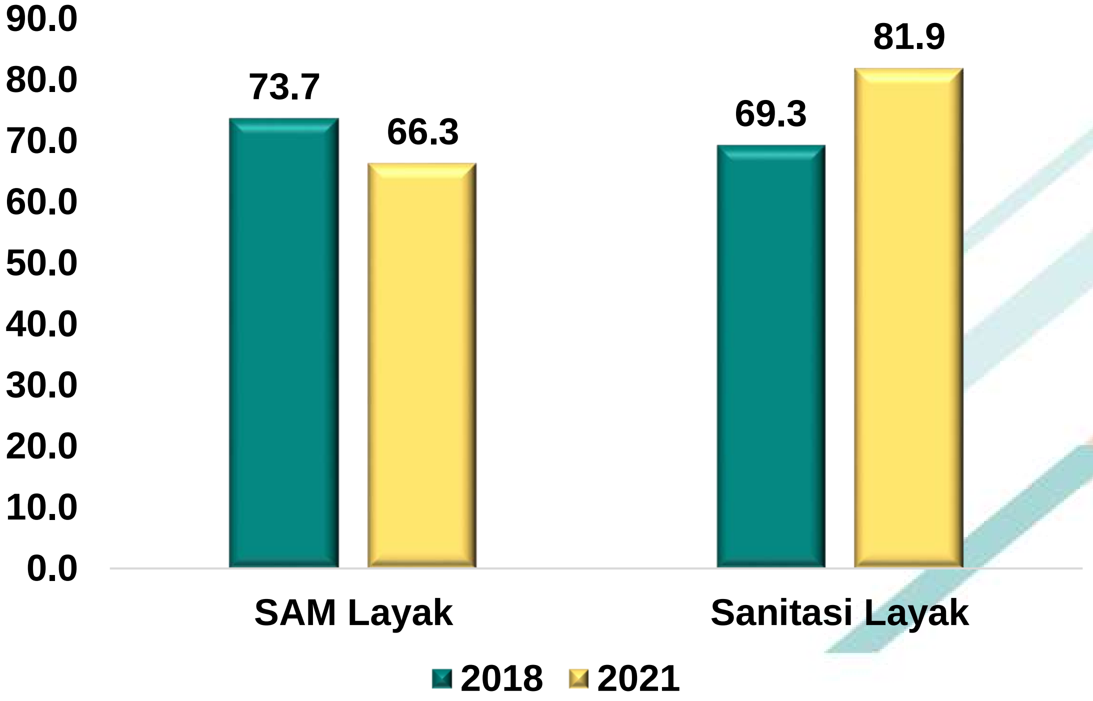

*** Memperhitungkan jarak sumur gali terlindung, mata air terlindung, sumur bor atau sumber pompa ≥ 10 meter dari sumber pencemaran**

GAMBARAN DETERMINAN MASALAH GIZI DI INDONESIA, RISKESDAS 2018 DAN SSGI 2021 (4)

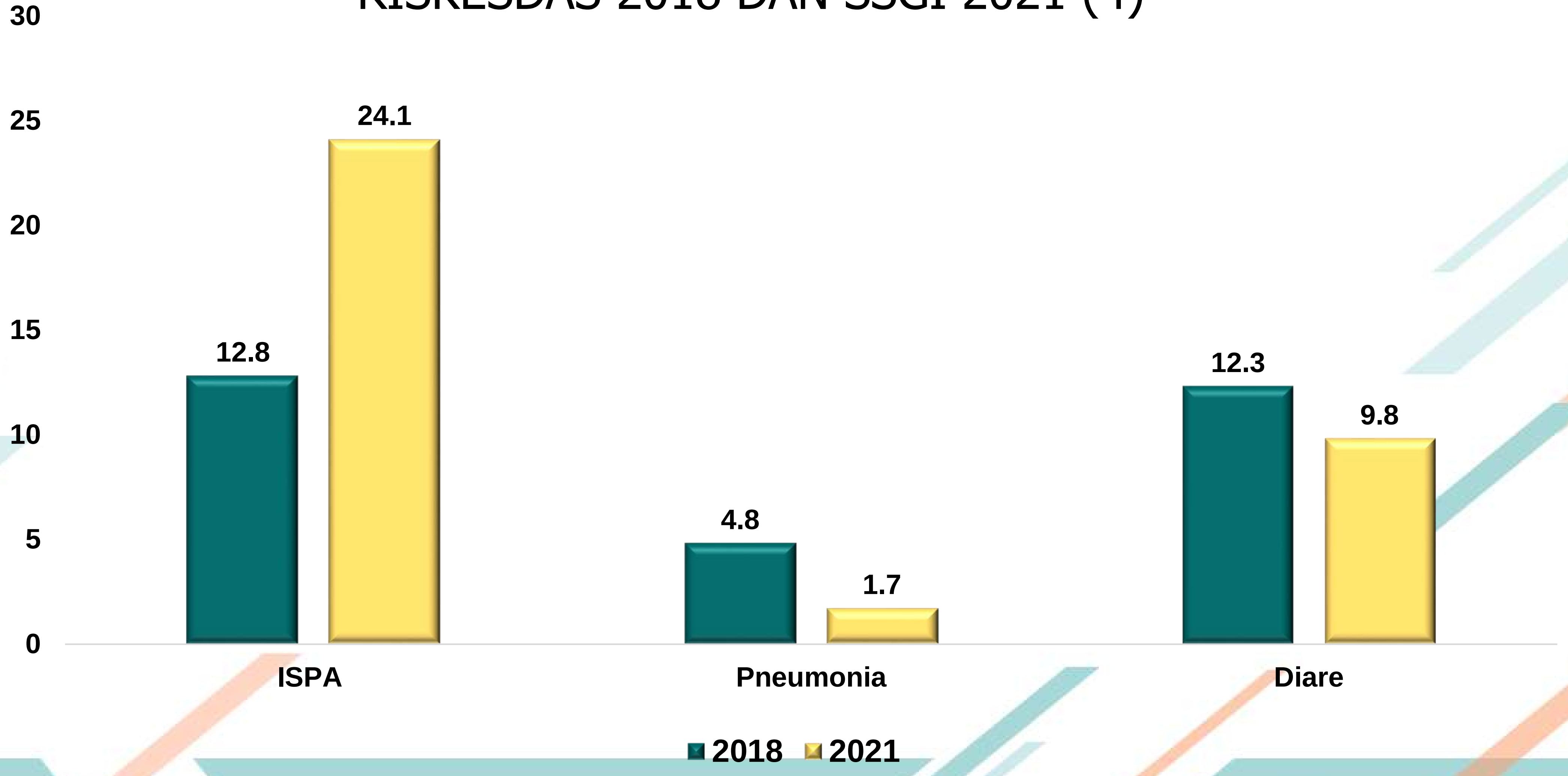

KEMENTERIAN KESEHATAN REPUBLIK INDONESIA

SSGI
Studi Status Gizi Indonesia

PROPORSI PEREMPUAN HAMIL YANG PERNAH MENDAPATKAN TABLET TAMBAH DARAH MENURUT PROVINSI, SSGI 2021

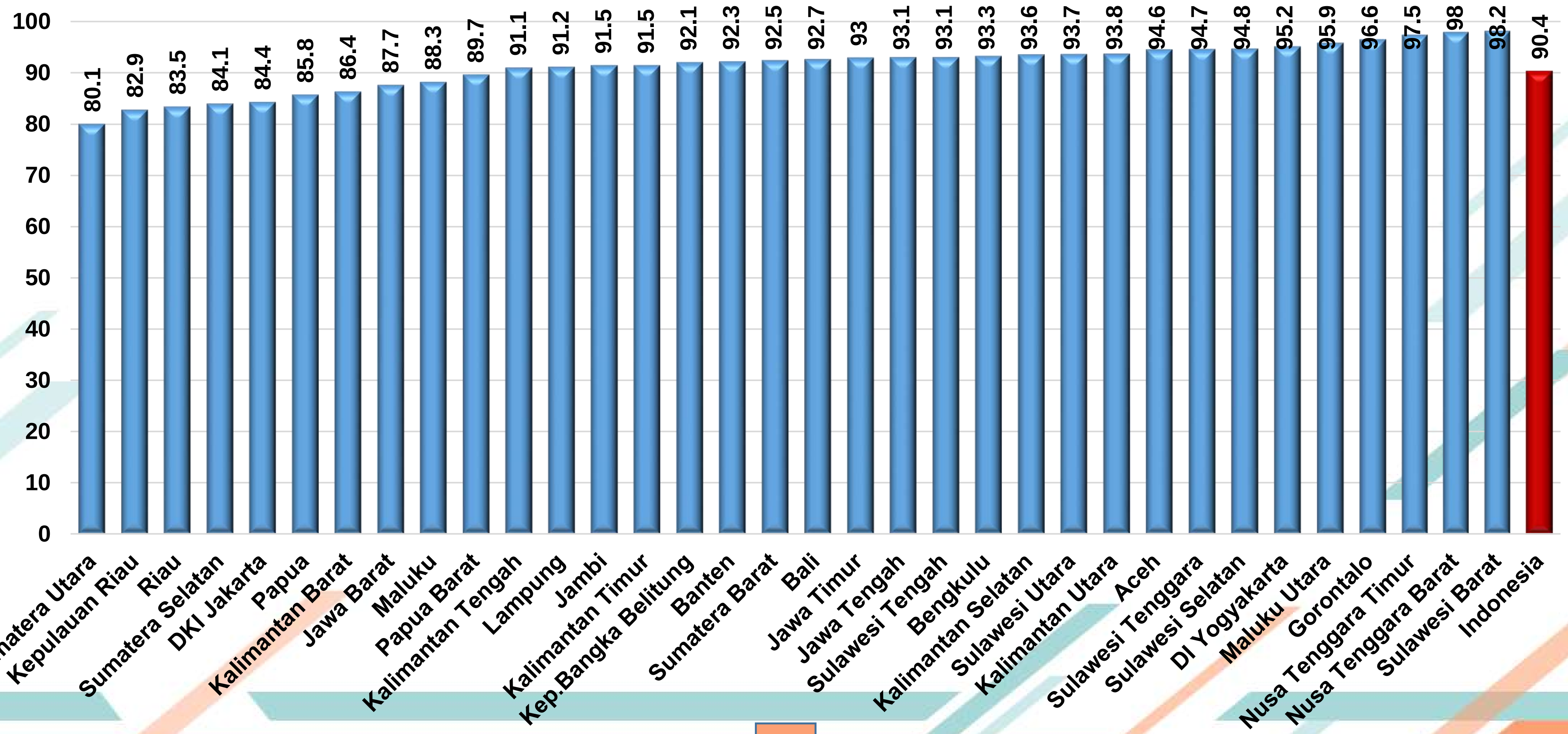

PROPORSI PEREMPUAN HAMIL USIA 10-54 TAHUN YANG BERSALIN DI FASILITAS KESEHATAN MENURUT PROVINSI, SSGI 2021

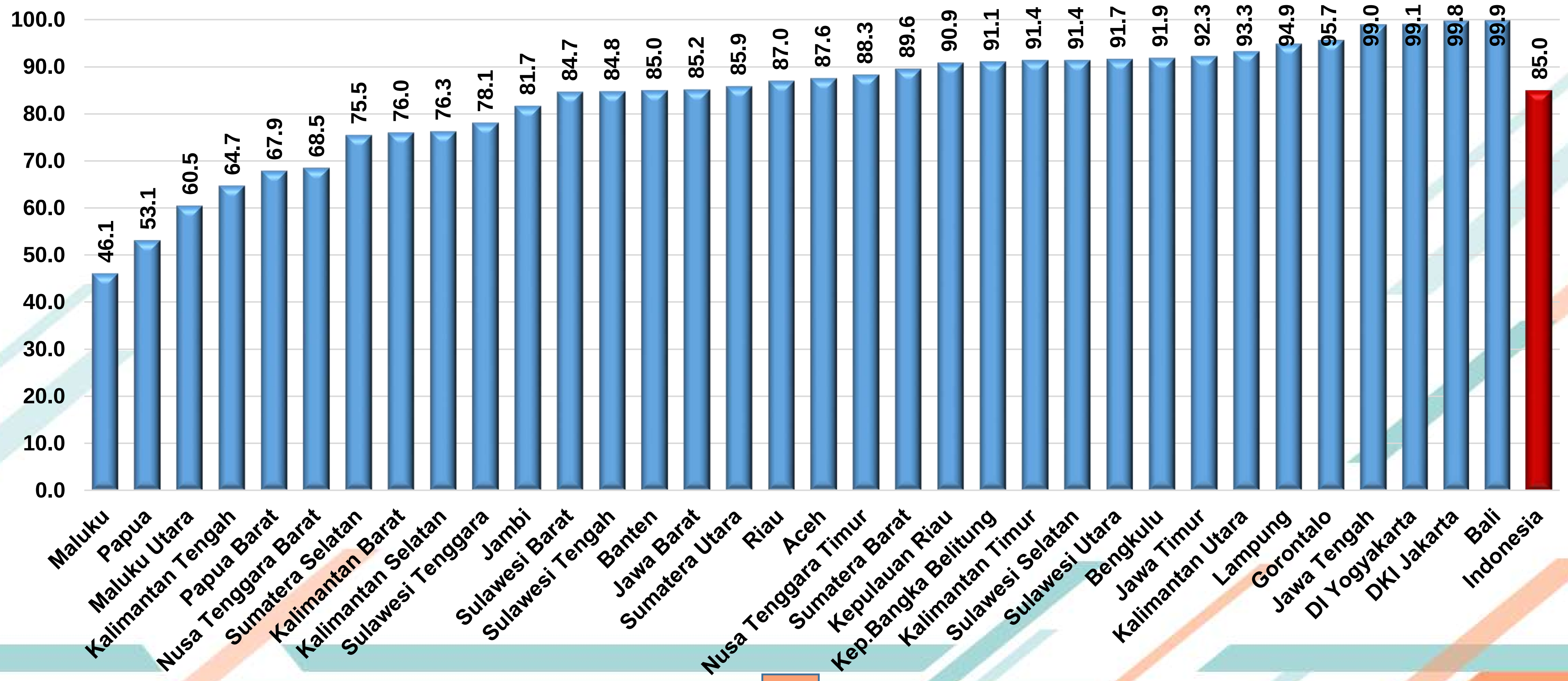

KEMENTERIAN KESEHATAN REPUBLIK INDONESIA

SSGI
Studi Status Gizi Indonesia

PROPORSI WANITA USIA SUBUR/PASANGAN YANG MENGGUNAKAN KB MODERN MENURUT PROVINSI, SSGI 2021

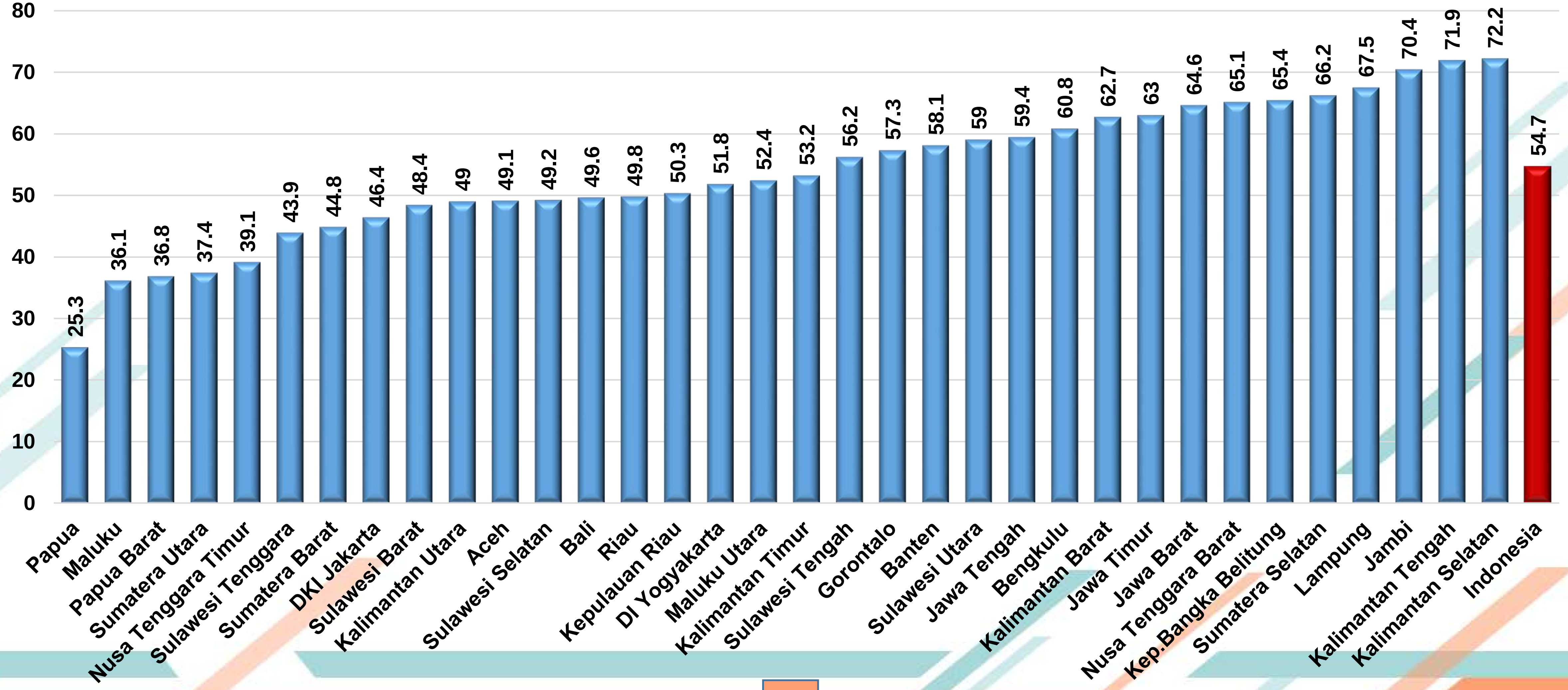

PROPORSI BALITA DENGAN BERAT BADAN LAHIR < 2500 GRAM*
MENURUT PROVINSI, SSGI 2021

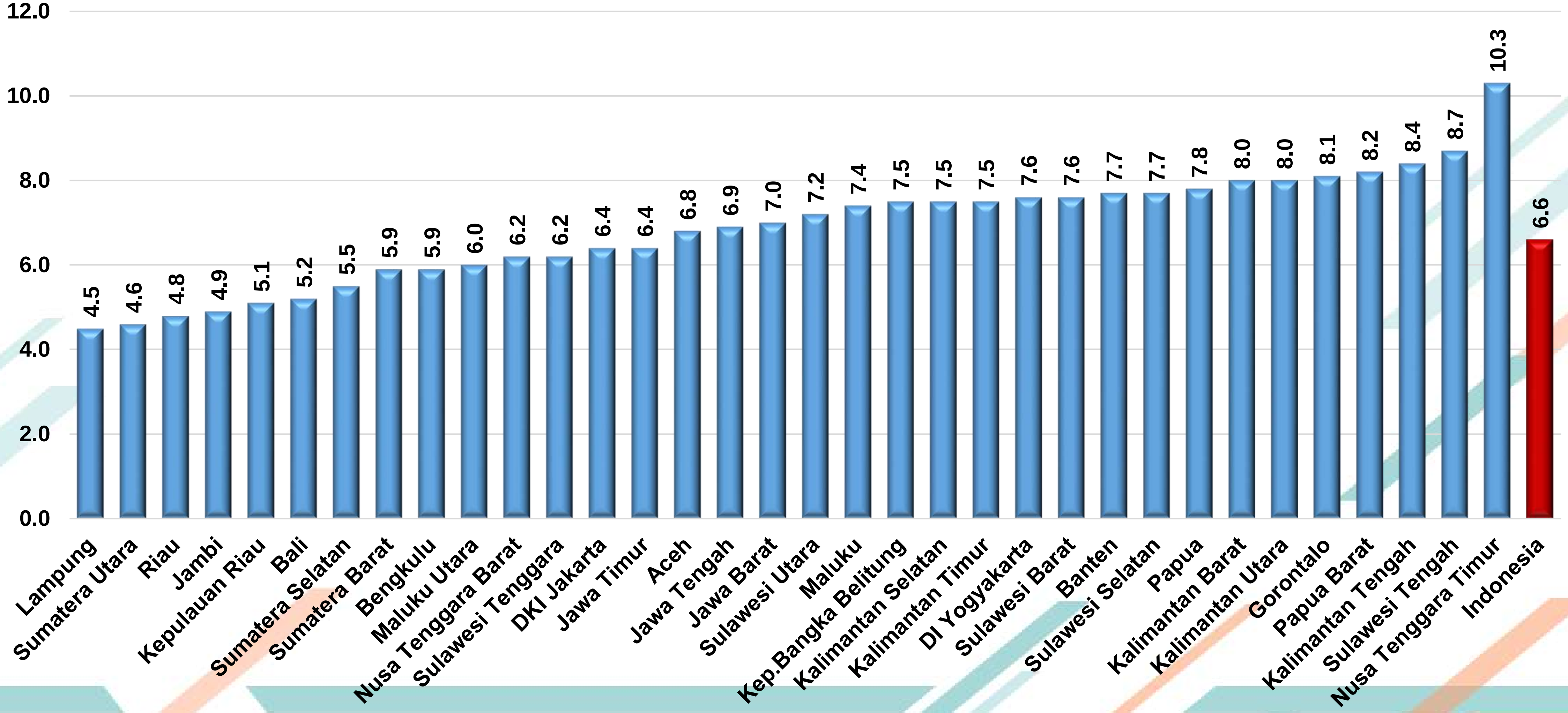

* Berdasarkan catatan dan ingatan

PROPORSI BALITA DENGAN PANJANG BADAN LAHIR <48 CM* MENURUT PROVINSI, SSGI 2021

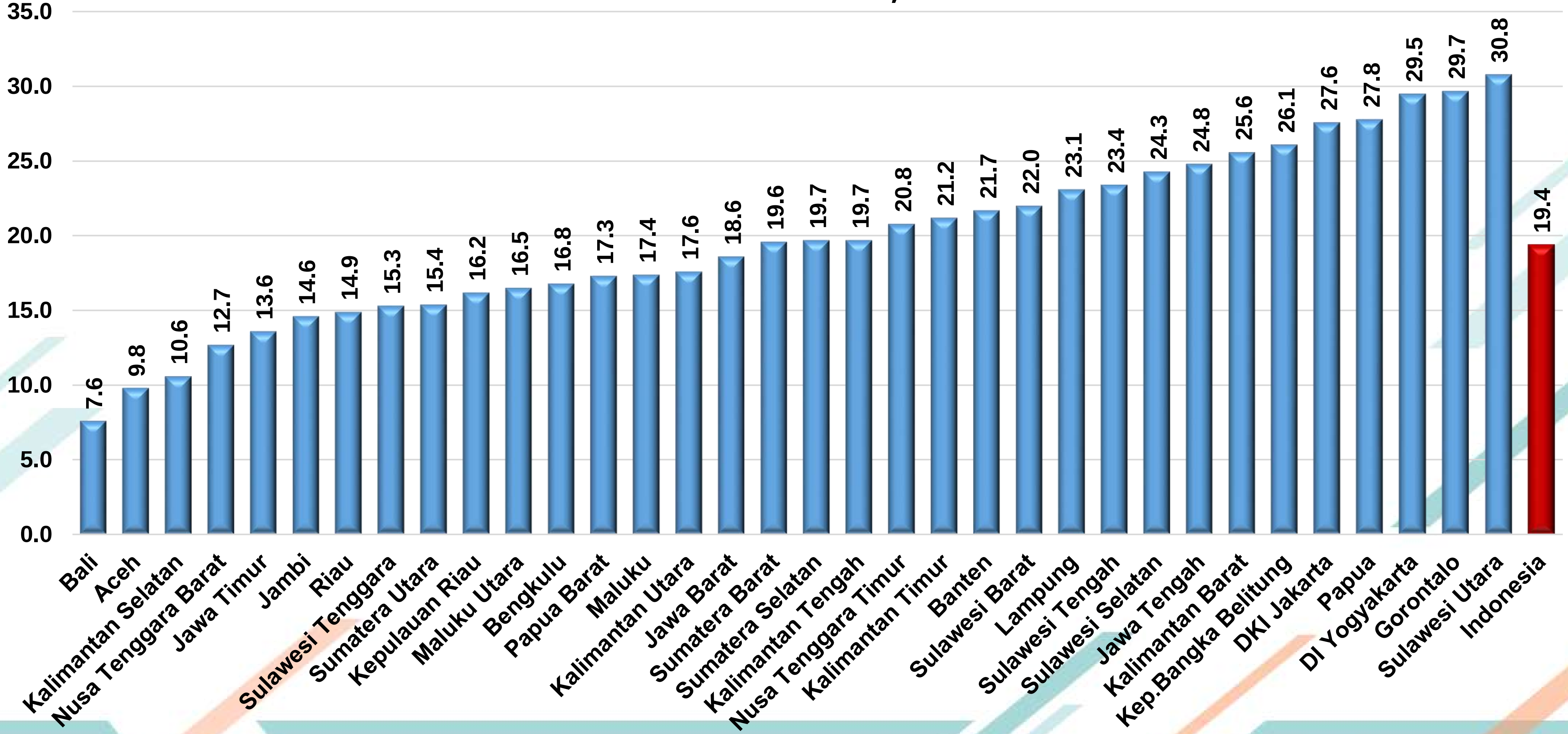

* Berdasarkan catatan dan ingatan

KEMENTERIAN KESEHATAN REPUBLIK INDONESIA

SSGI
Studi Status Gizi Indonesia

PROPORSI BALITA YANG MENDAPATKAN INISIASI MENYUSU DINI MENURUT PROVINSI, SSGI 2021

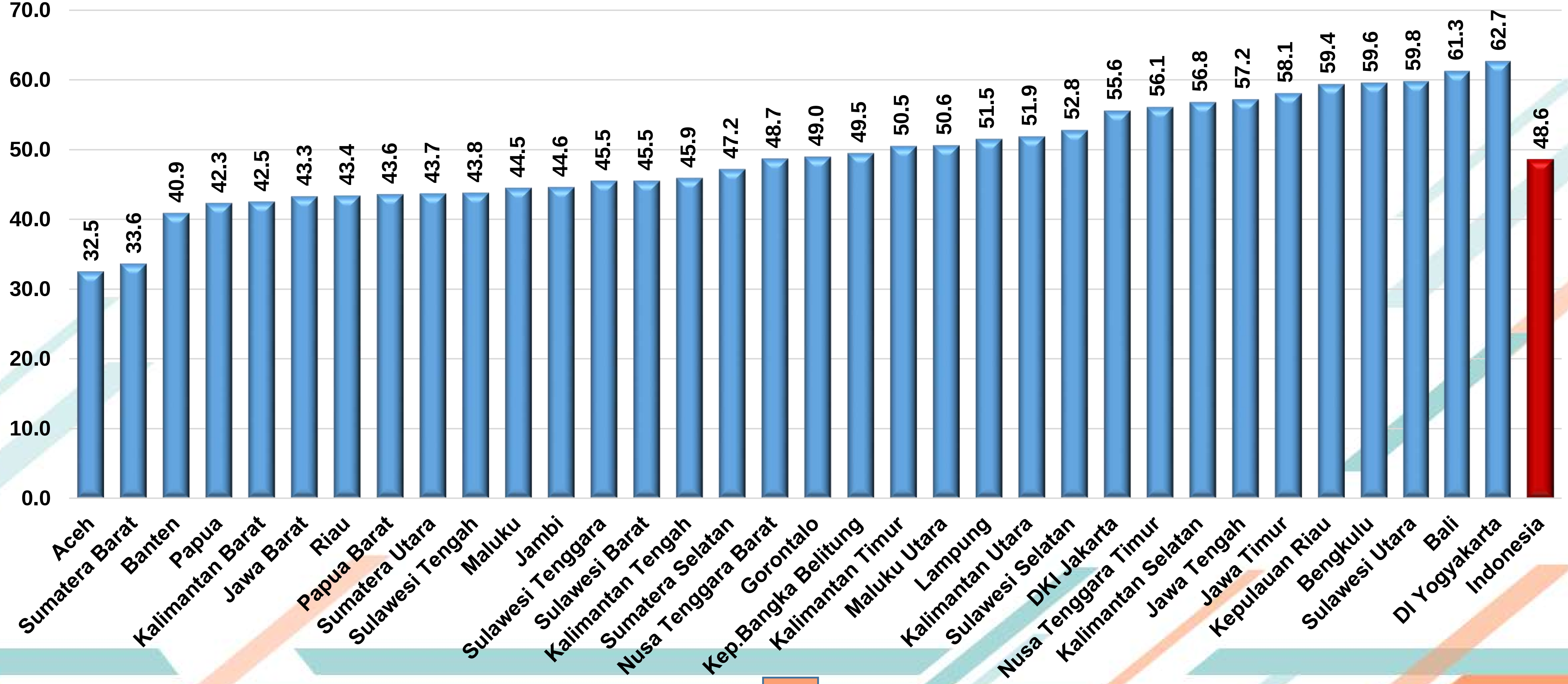

KEMENTERIAN KESEHATAN REPUBLIK INDONESIA

SSGI
Studi Status Gizi Indonesia

PROPORSI BAYI USIA 0-5 BULAN MENDAPATKAN ASI EKSKLUSIF MENURUT PROVINSI, SSGI 2021

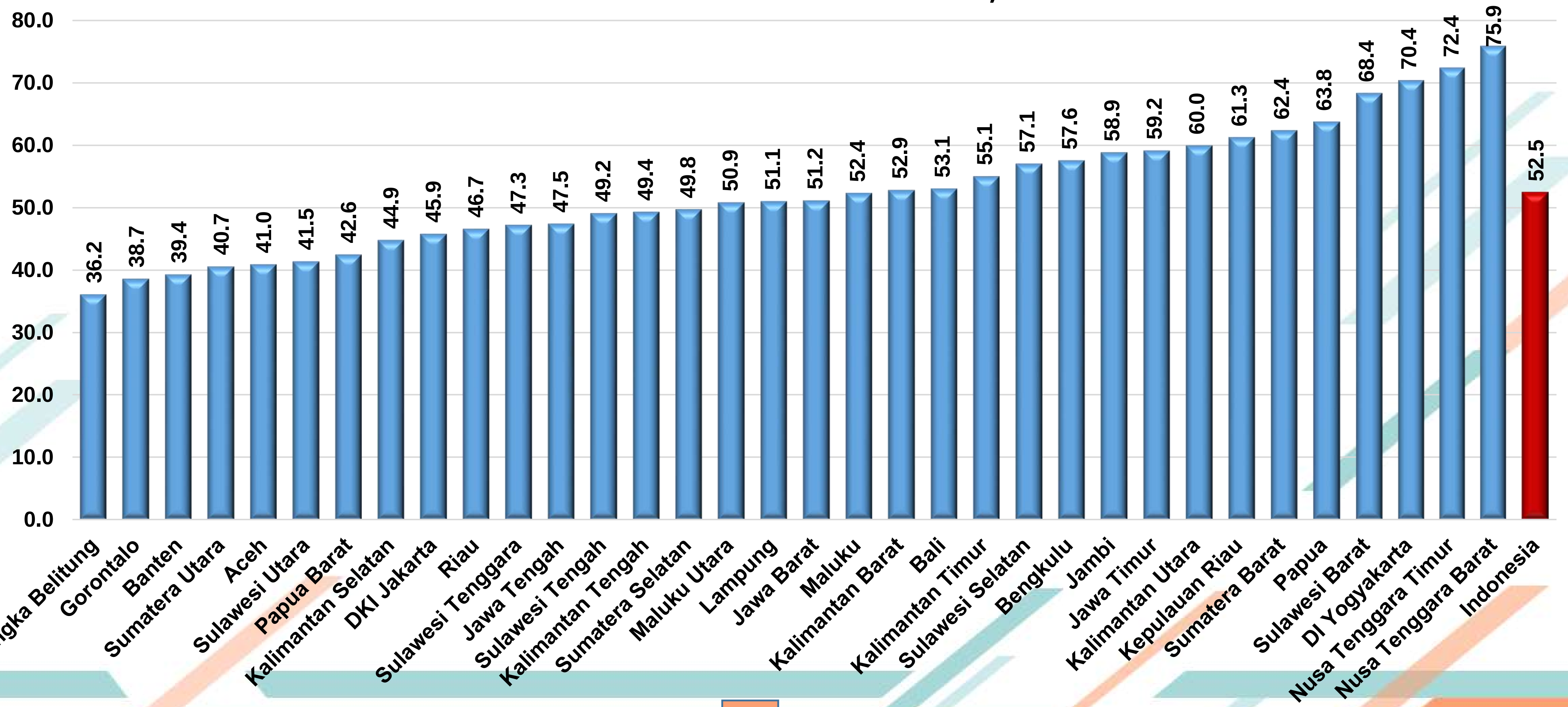

KEMENTERIAN KESEHATAN REPUBLIK INDONESIA

SSGI
Studi Status Gizi Indonesia

PROPORSI BAYI USIA 6-23 BULAN MENDAPATKAN ASI EKSKLUSIF MENURUT PROVINSI, SSGI 2021

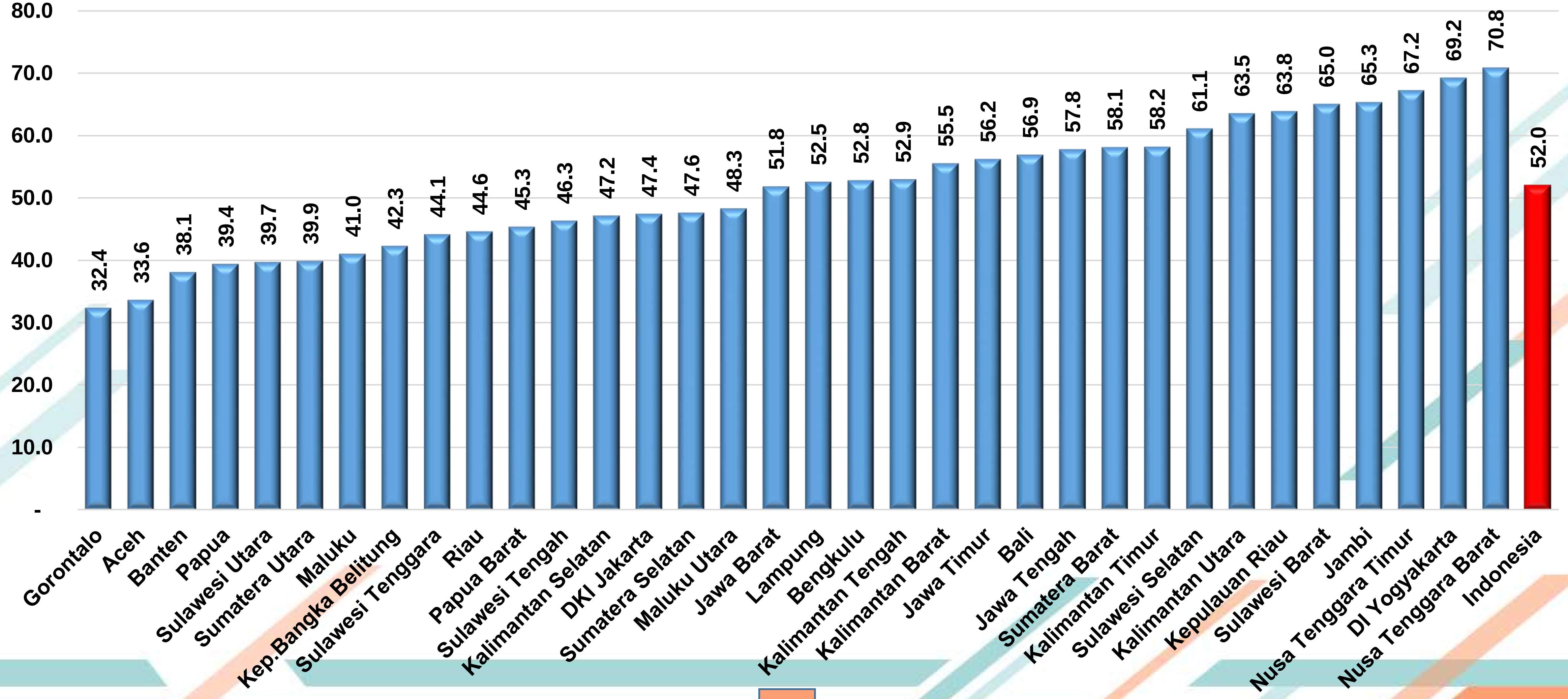

KEMENTERIAN KESEHATAN REPUBLIK INDONESIA

SSGI Studi Status Gizi Indonesia

PROPORSI BADUTA SAAT PERTAMA KALI DIBERIKAN MPASI PADA USIA ≥ 6 BULAN MENURUT PROVINSI, SSGI 2021

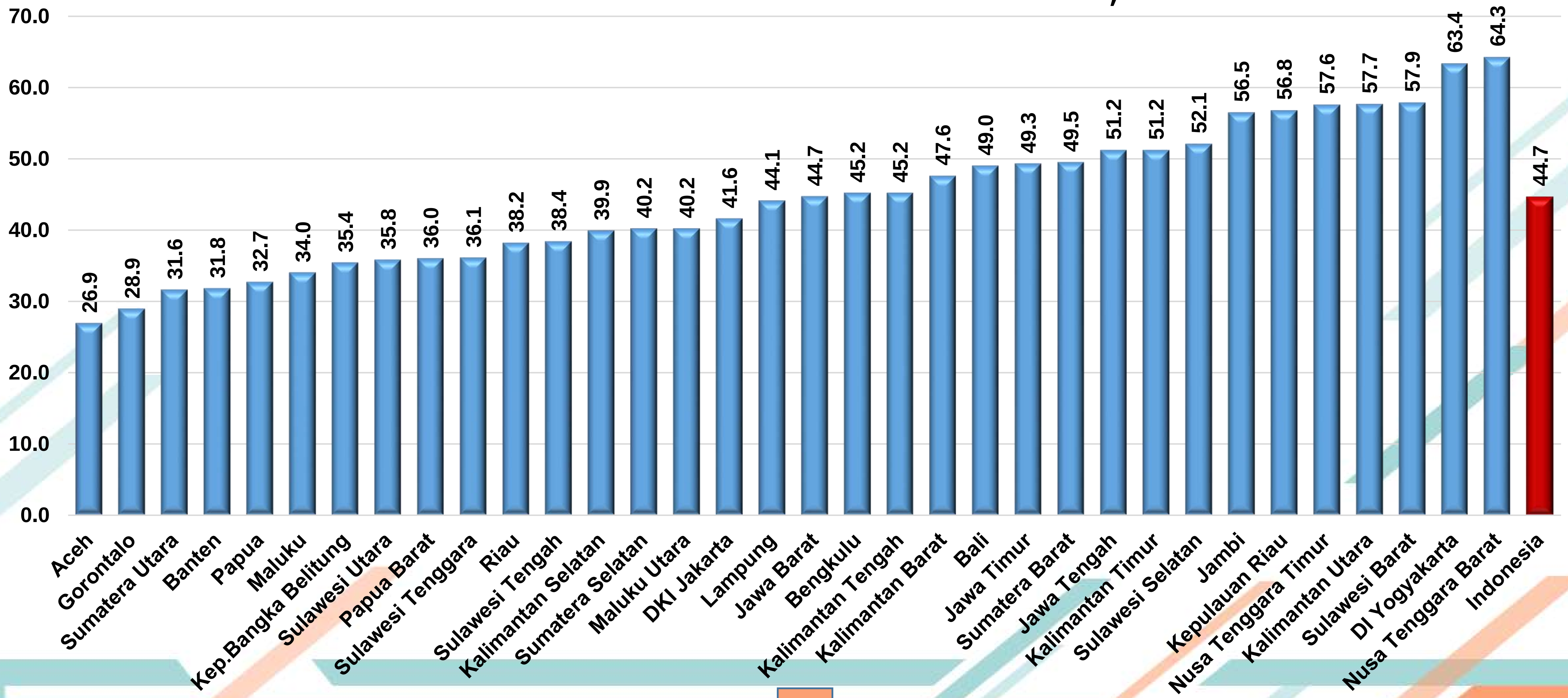

KEMENTERIAN KESEHATAN REPUBLIK INDONESIA

SSGI Studi Status Gizi Indonesia

PROPORSI ANAK USIA 0-23 BULAN MENGONSUMSI MAKANAN BERAGAM MENURUT PROVINSI, SSGI 2021

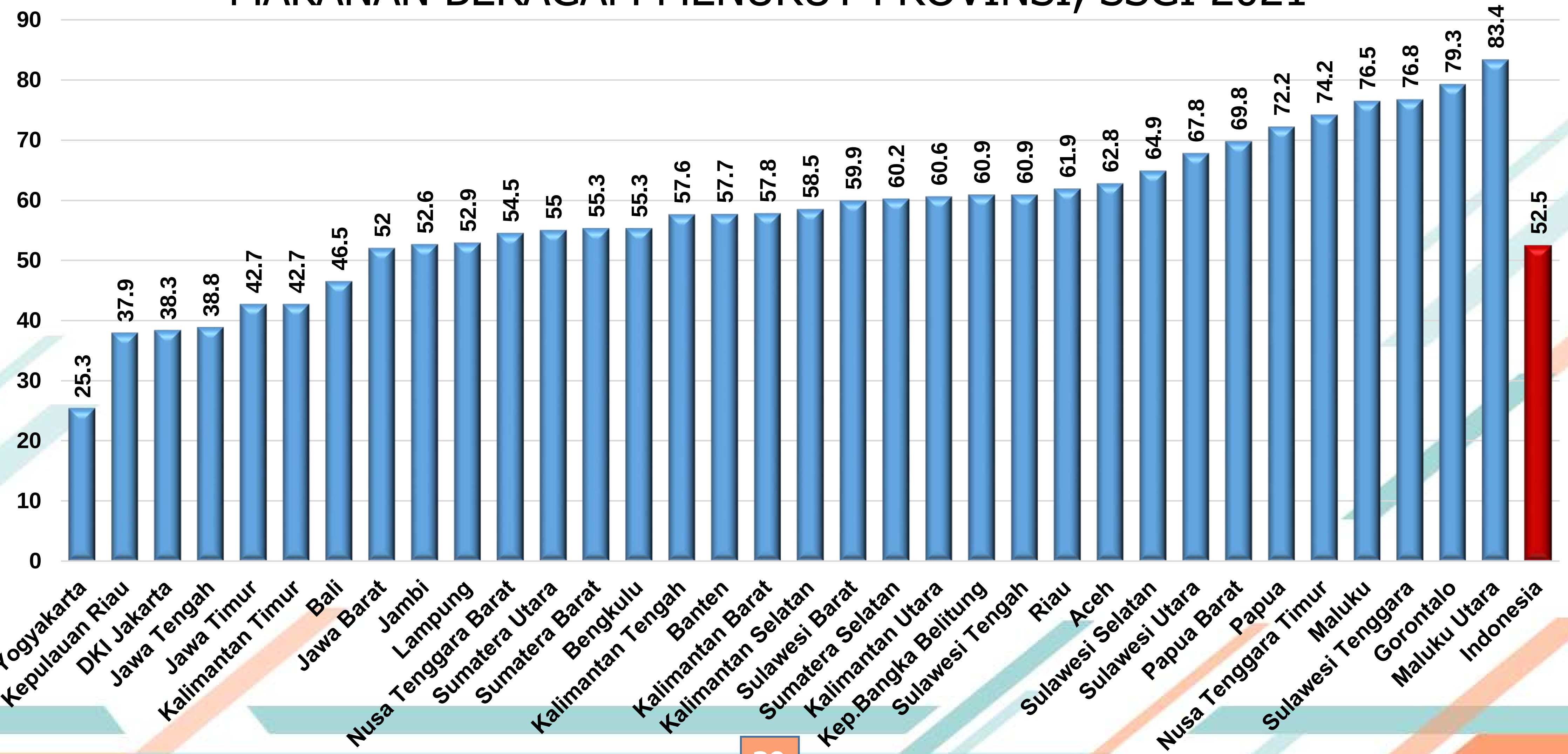

KEMENTERIAN KESEHATAN REPUBLIK INDONESIA

SSGI Studi Status Gizi Indonesia

PROPORSI BALITA YANG MEMILIKI BUKU KIA, SSGI 2021

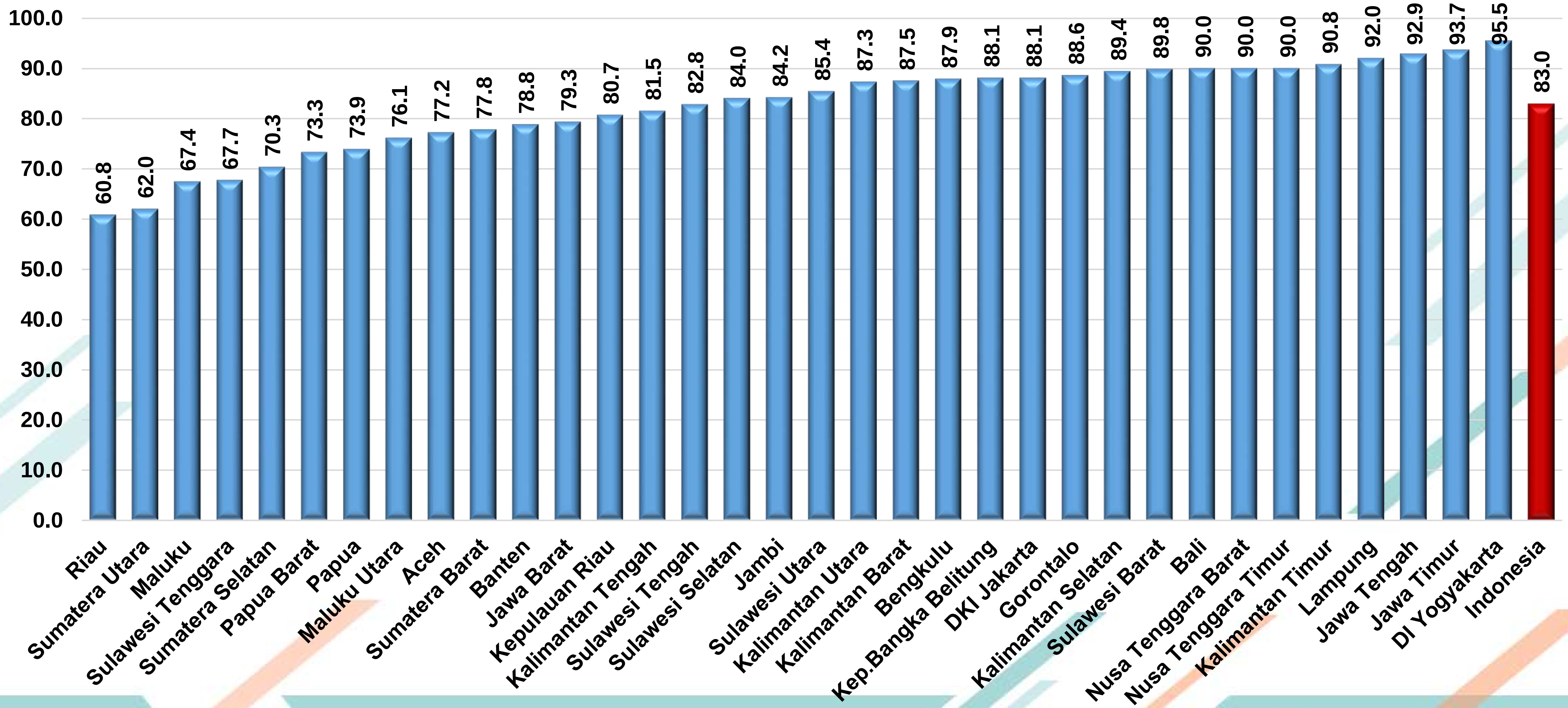

KEMENTERIAN KESEHATAN REPUBLIK INDONESIA

SSGI
Studi Status Gizi Indonesia

PROPORSI BALITA YANG MELAKUKAN PENIMBANGAN BERAT BADAN SESUAI STANDAR (≥ 8x SETAHUN), SSGI 2021

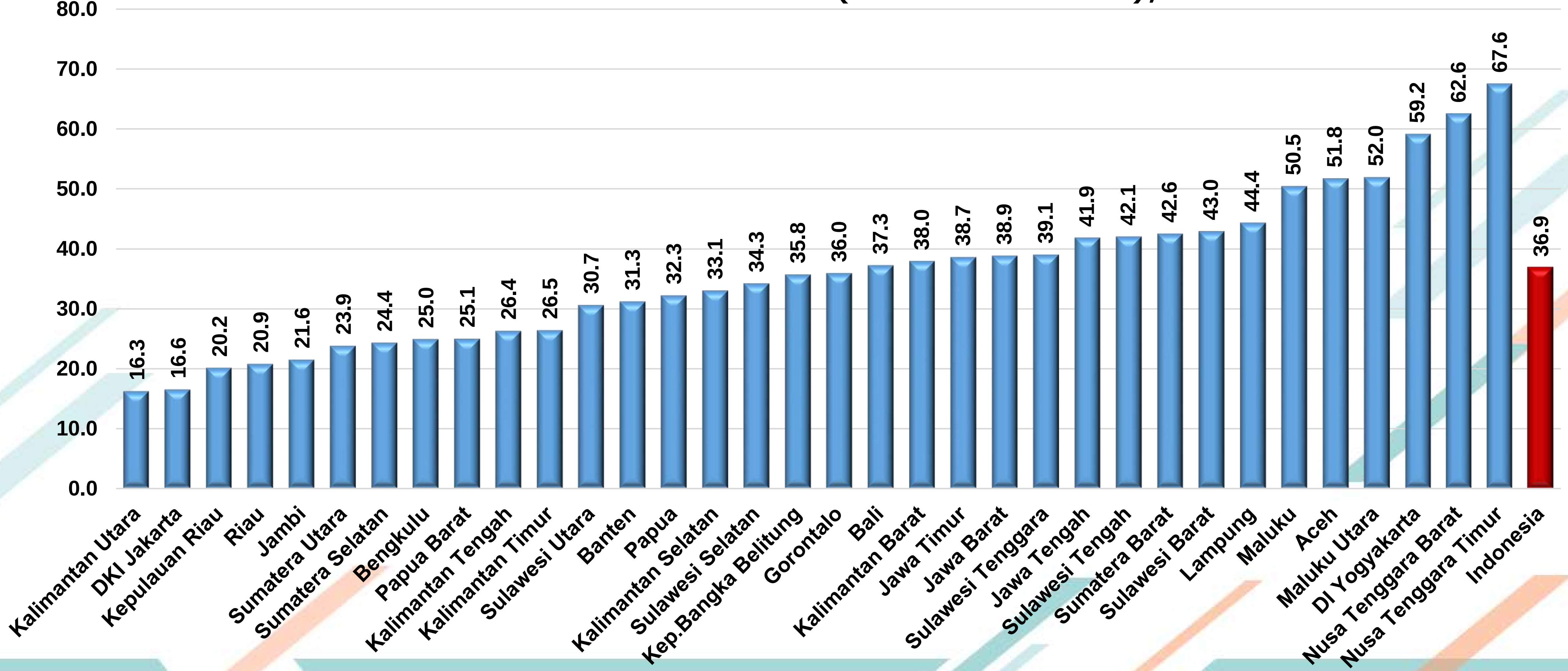

PROPORSI BALITA YANG MELAKUKAN PENGUKURAN TINGGI BADAN SESUAI STANDAR (≥ 2x SETAHUN), SSGI 2021

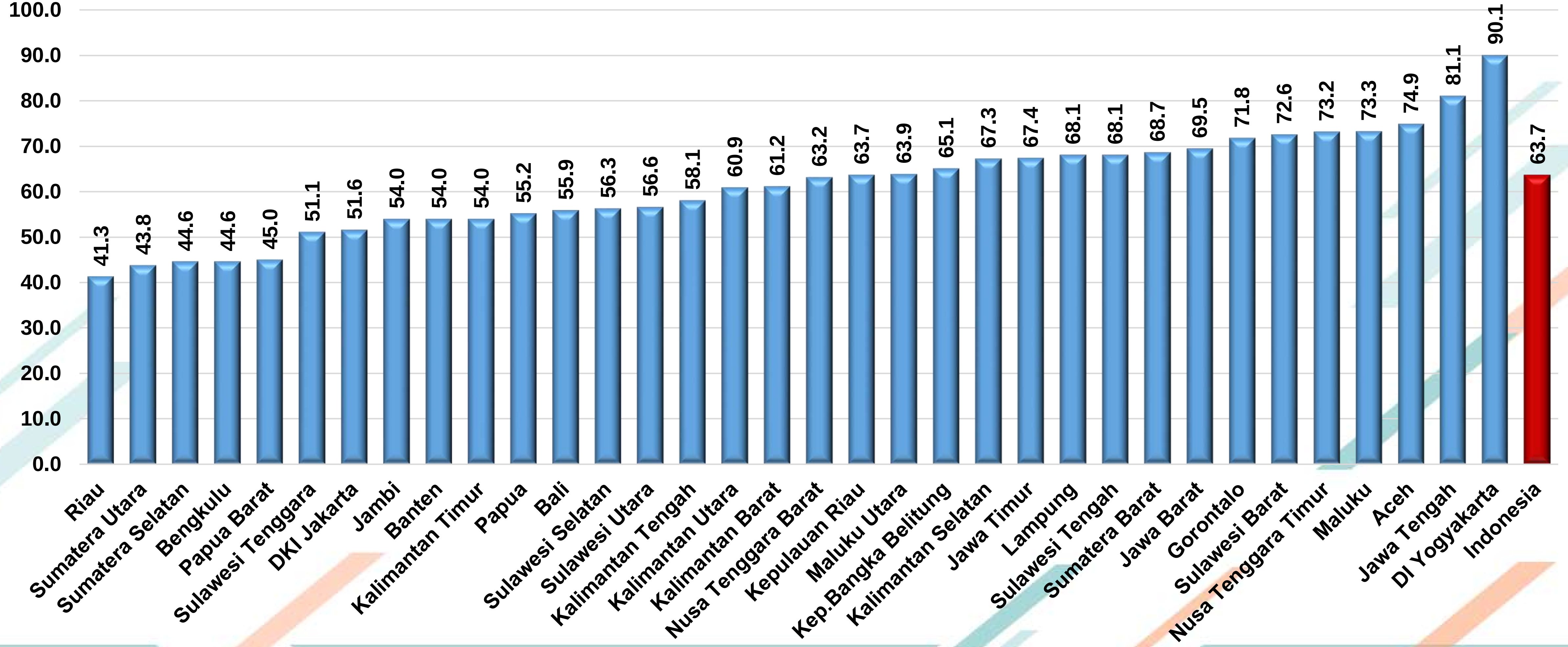

KEMENTERIAN KESEHATAN REPUBLIK INDONESIA

SSGI Studi Status Gizi Indonesia

PROPORSI ANAK 12-23 BULAN YANG MENDAPATKAN IMUNISASI DASAR LENGKAP, SSGI 2021

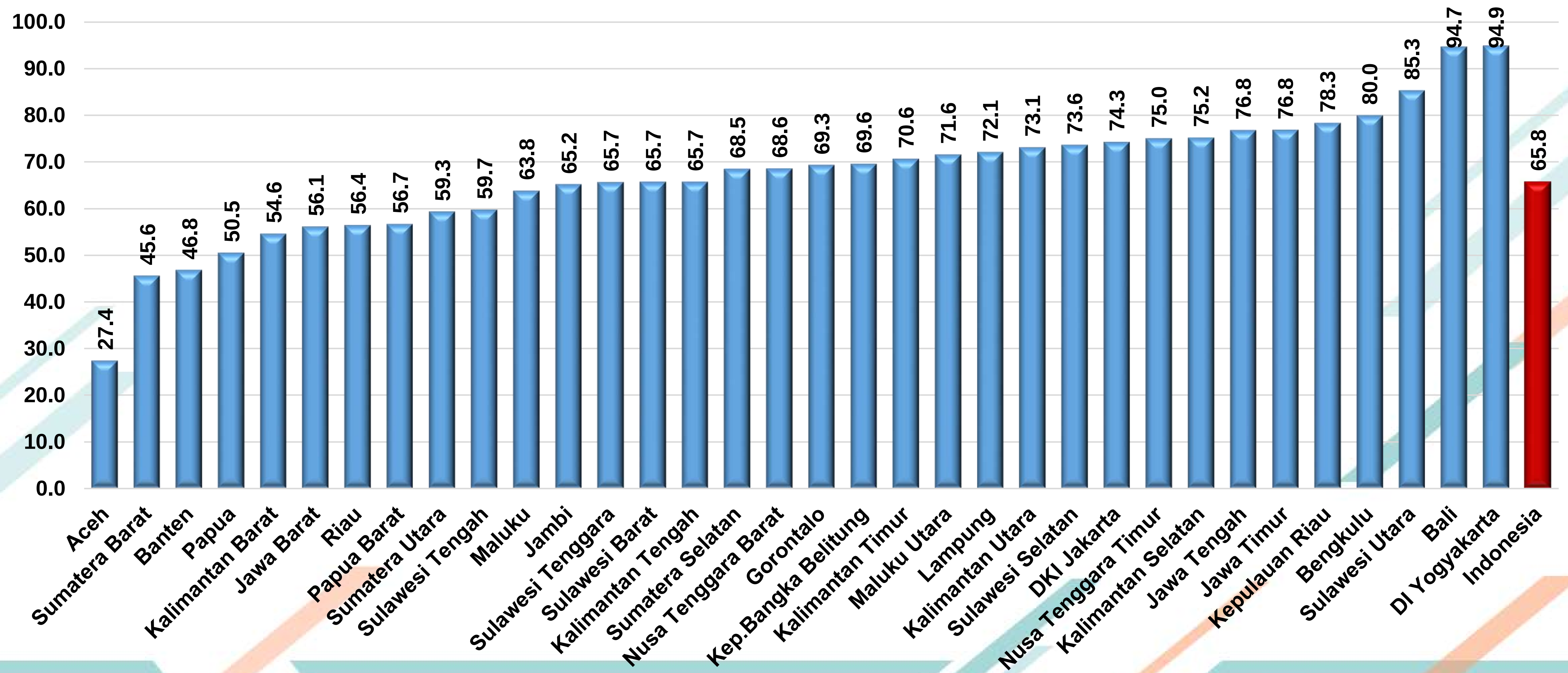

PROPORSI ANAK USIA 6-59 BULAN MENDAPATKAN VITAMIN A MENURUT PROVINSI, SSGI 2021

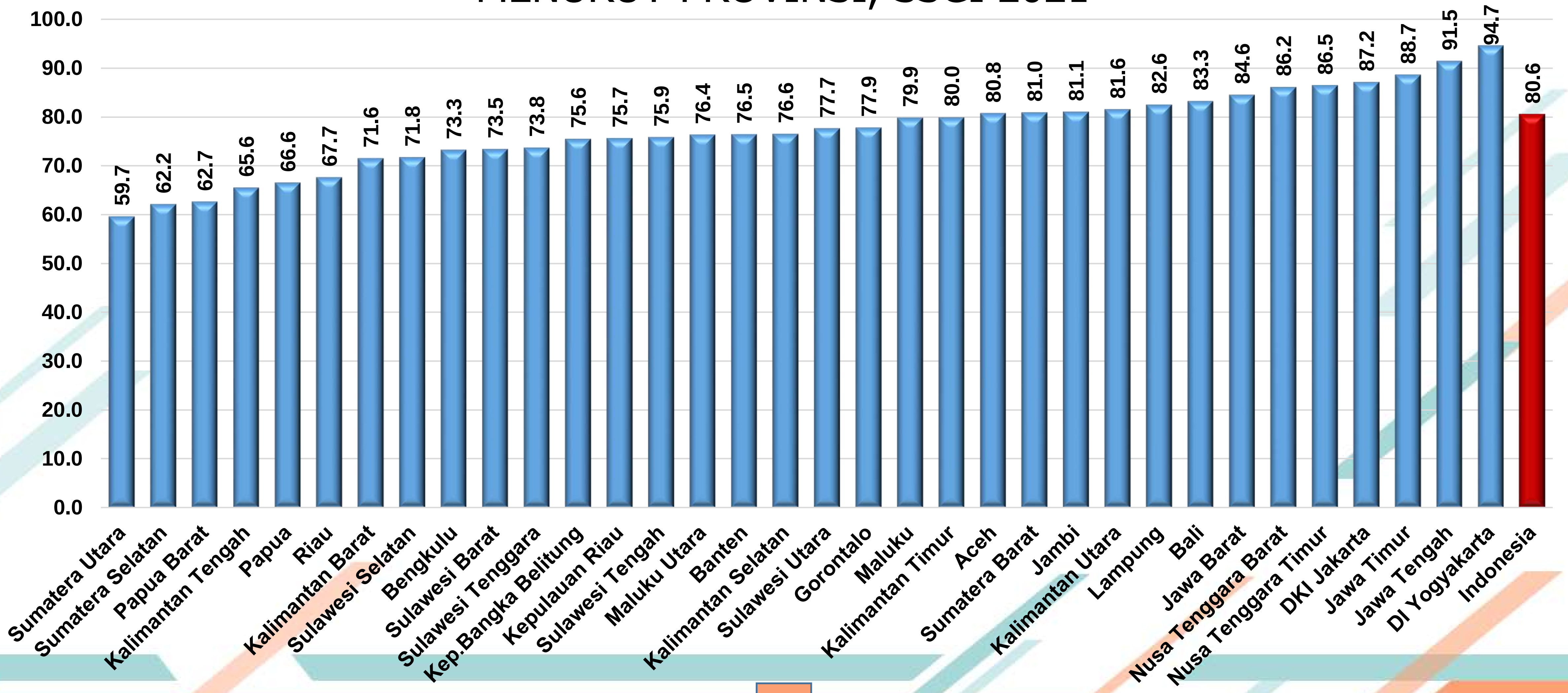

PROPORSI BADUTA YANG MASIH DISUSUI MENURUT PROVINSI, SSGI 2021

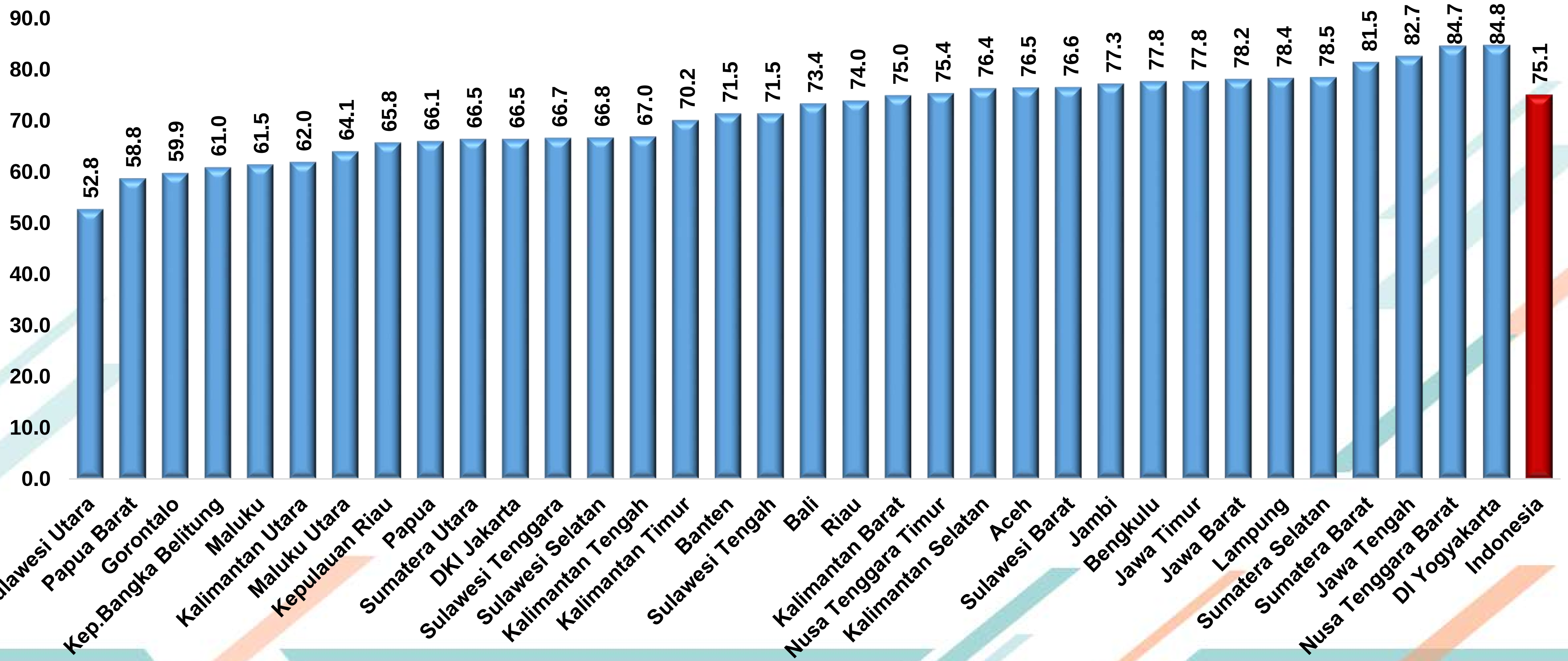

KEMENTERIAN KESEHATAN REPUBLIK INDONESIA

SSGI
Studi Status Gizi Indonesia

PROPORSI BALITA YANG MENDERITA ISPA BERDASARKAN DIAGNOSIS/GEJALA MENURUT PROVINSI, SSGI 2021

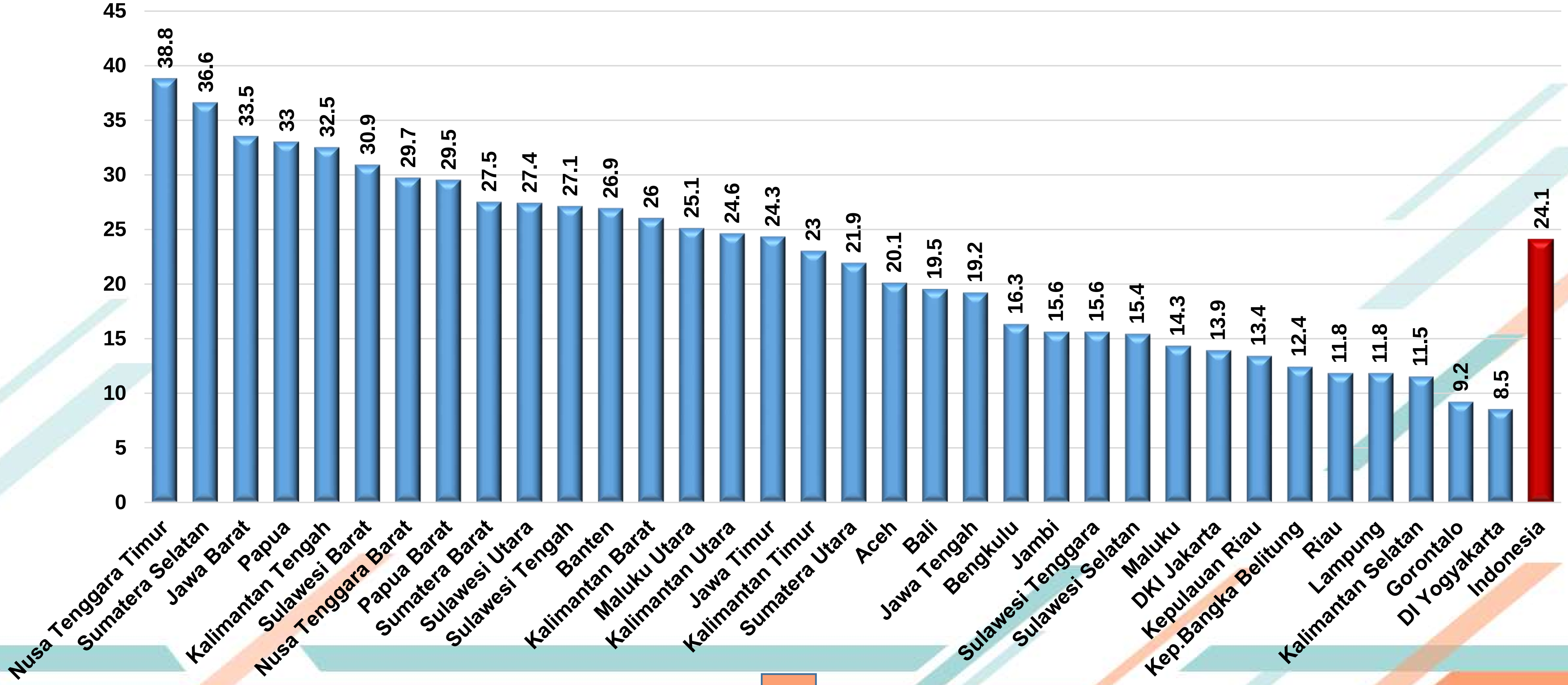

PROPORSI BALITA YANG MENDERITA PNEUMONIA BERDASARKAN DIAGNOSIS/GEJALA MENURUT PROVINSI, SSGI 2021

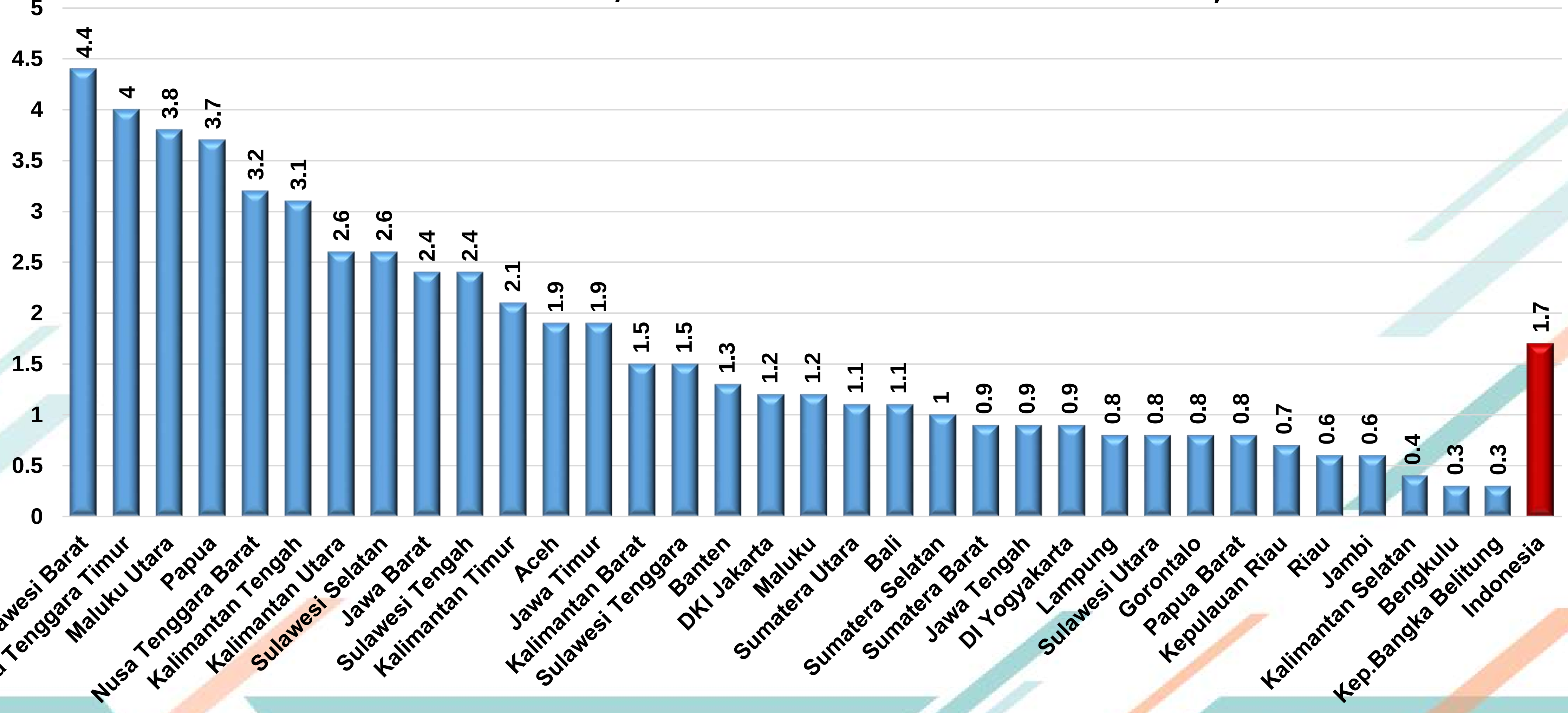

PROPORSI BALITA YANG MENDERITA DIARE BERDASARKAN DIAGNOSIS/GEJALA MENURUT PROVINSI, SSGI 2021

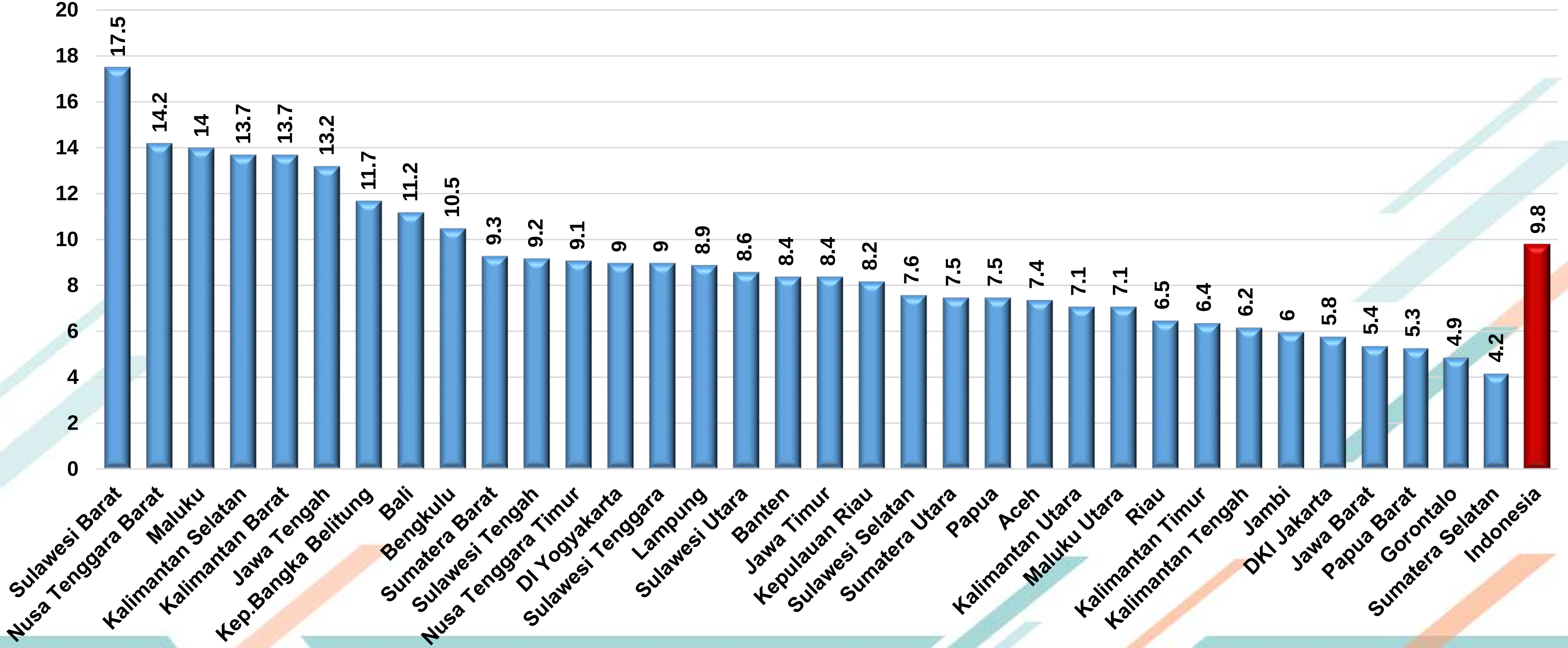

KEMENTERIAN KESEHATAN REPUBLIK INDONESIA

SSGI
Studi Status Gizi Indonesia

PROPORSI BALITA YANG MENDERITA KECACINGAN BERDASARKAN DIAGNOSIS/GEJALA MENURUT PROVINSI, SSGI 2021

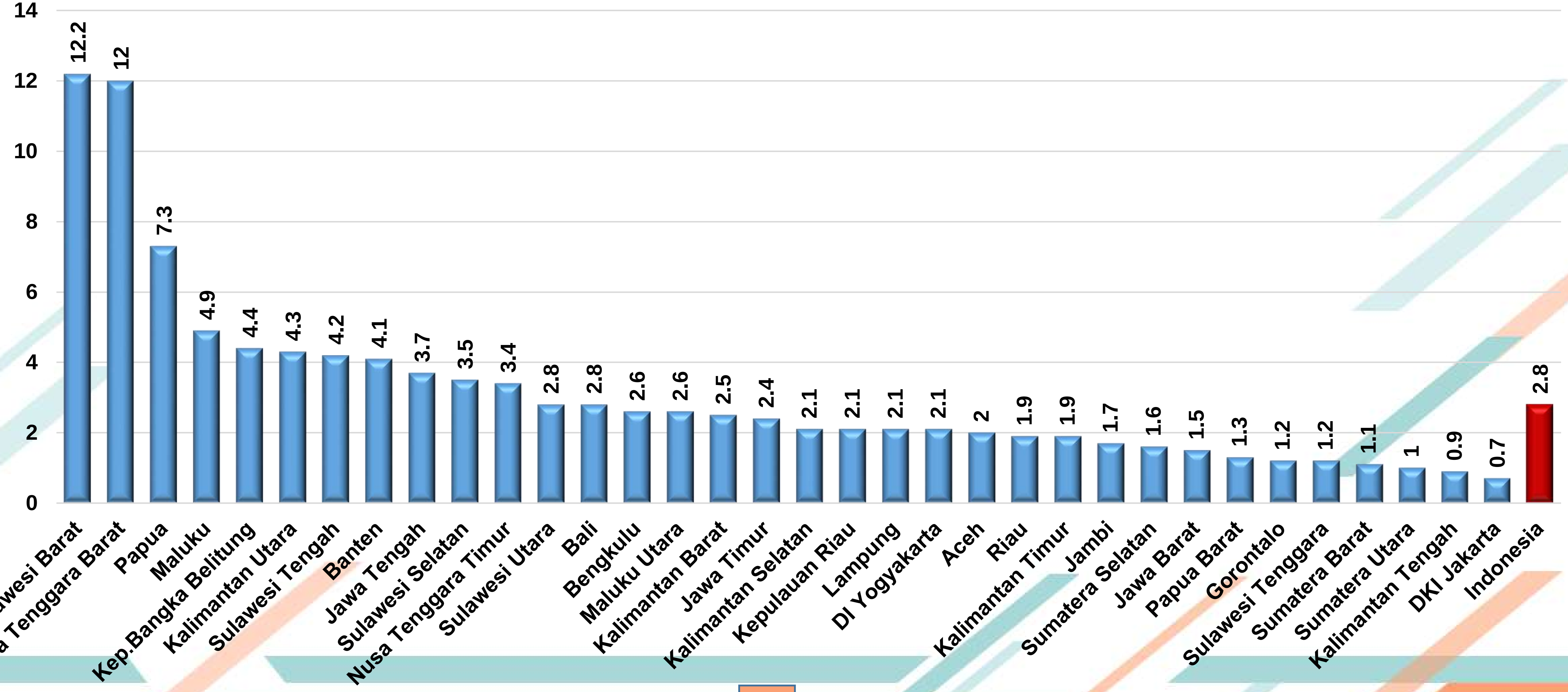

KEMENTERIAN KESEHATAN REPUBLIK INDONESIA

SSGI Studi Status Gizi Indonesia

PROPORSI BALITA YANG MENDERITA CAMPAK BERDASARKAN DIAGNOSIS/GEJALA MENURUT PROVINSI, SSGI 2021

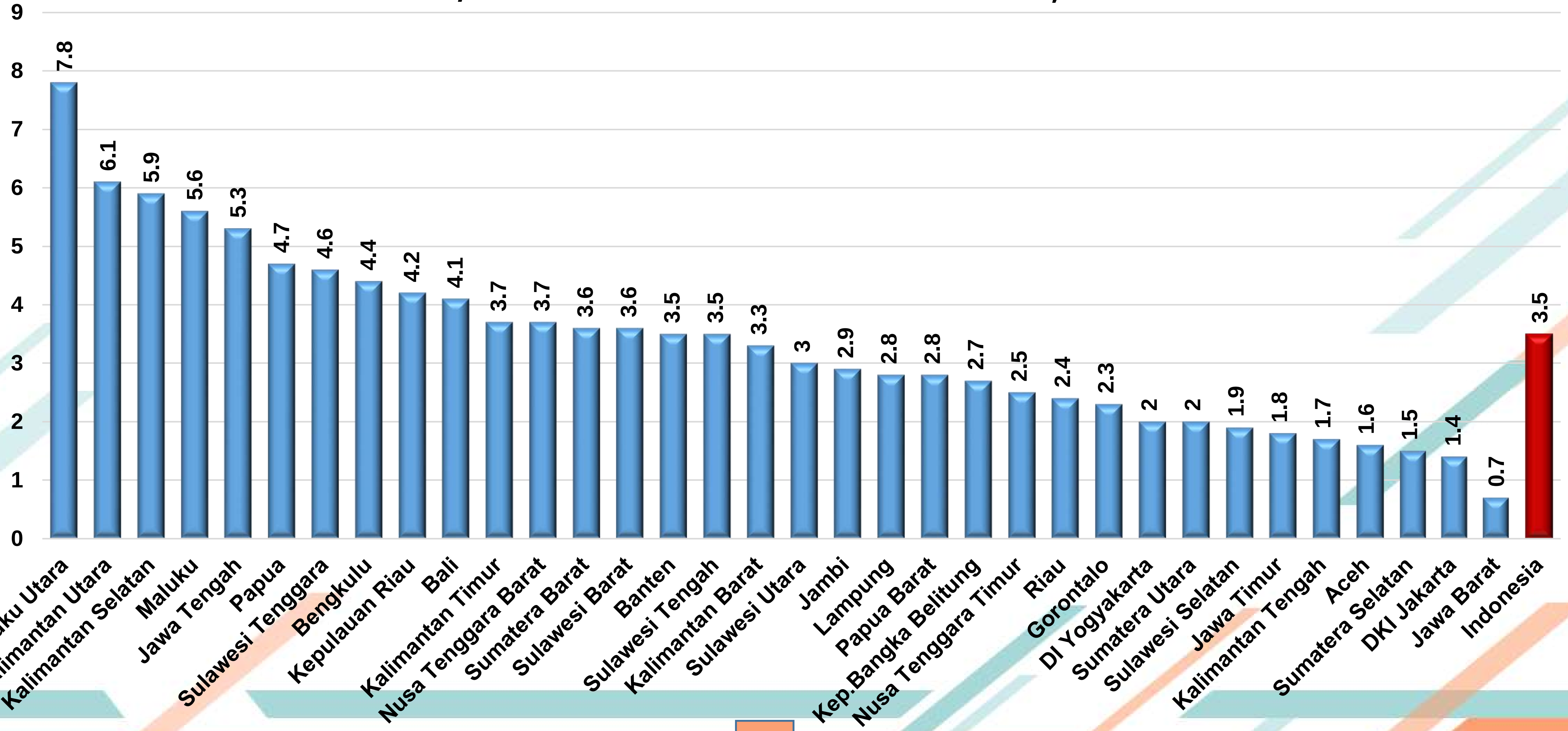

KEMENTERIAN KESEHATAN REPUBLIK INDONESIA

SSGI
Studi Status Gizi Indonesia

PROPORSI ANGGOTA RUMAH TANGGA YANG MEMILIKI JAMINAN PELAYANAN KESEHATAN, SSGI 2021

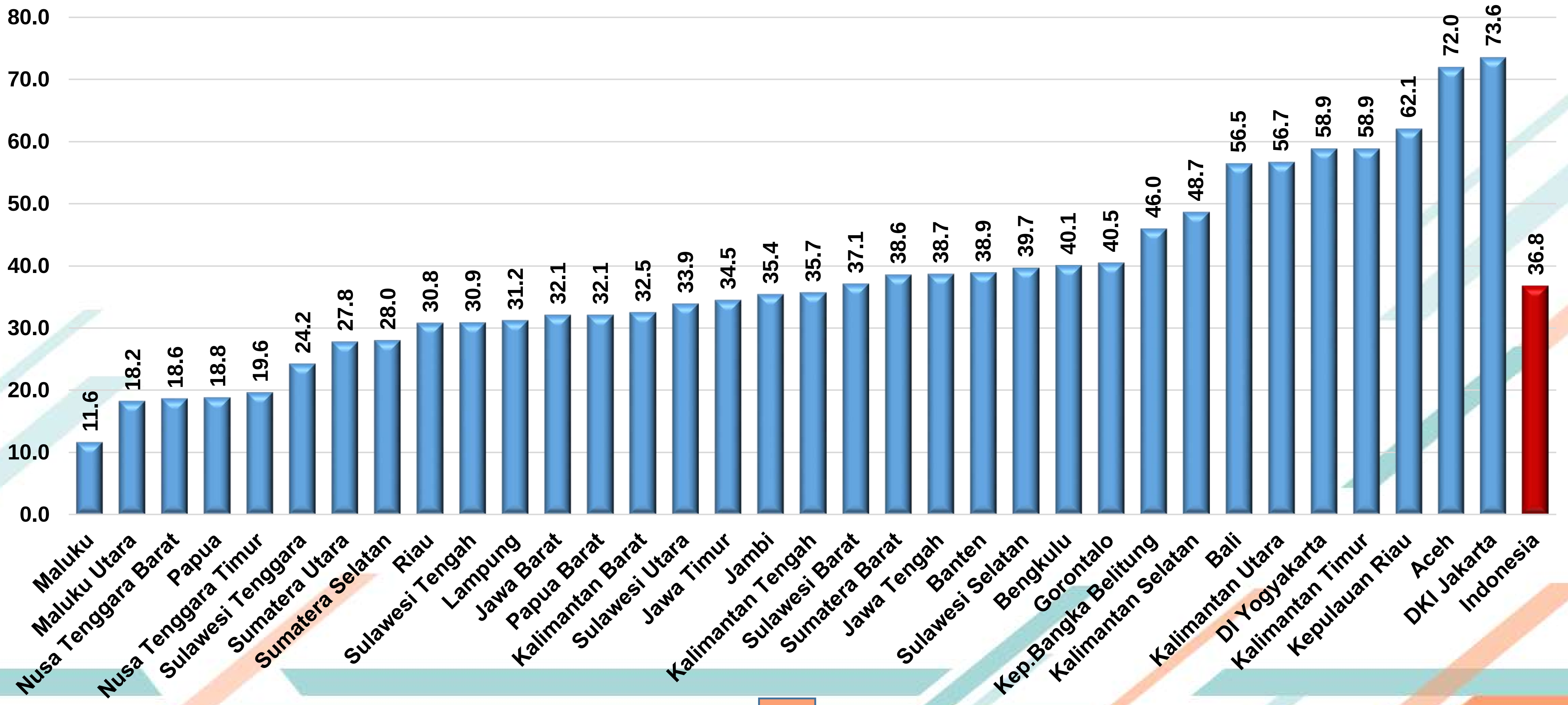

KEMENTERIAN KESEHATAN REPUBLIK INDONESIA

SSGI Studi Status Gizi Indonesia

PROPORSI BALITA SAKIT YANG BEROBAT KE FASILITAS PELAYANAN KESEHATAN, SSGI 2021

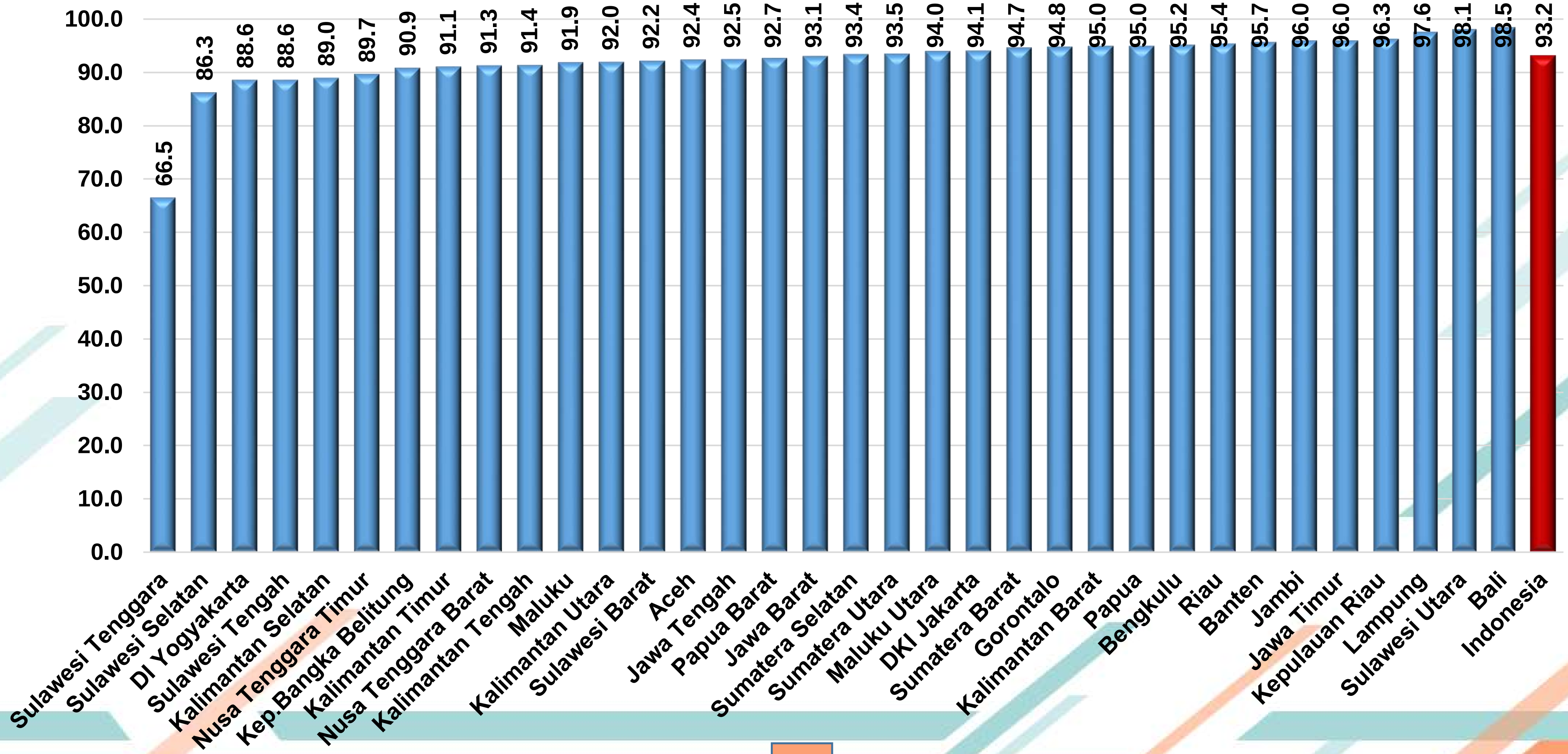

KEMENTERIAN KESEHATAN REPUBLIK INDONESIA

SSGI
Studi Status Gizi Indonesia

PROPORSI RUMAH TANGGA YANG MEMILIKI AKSES SARANA AIR MINUM LAYAK* MENURUT PROVINSI, SSGI 2021

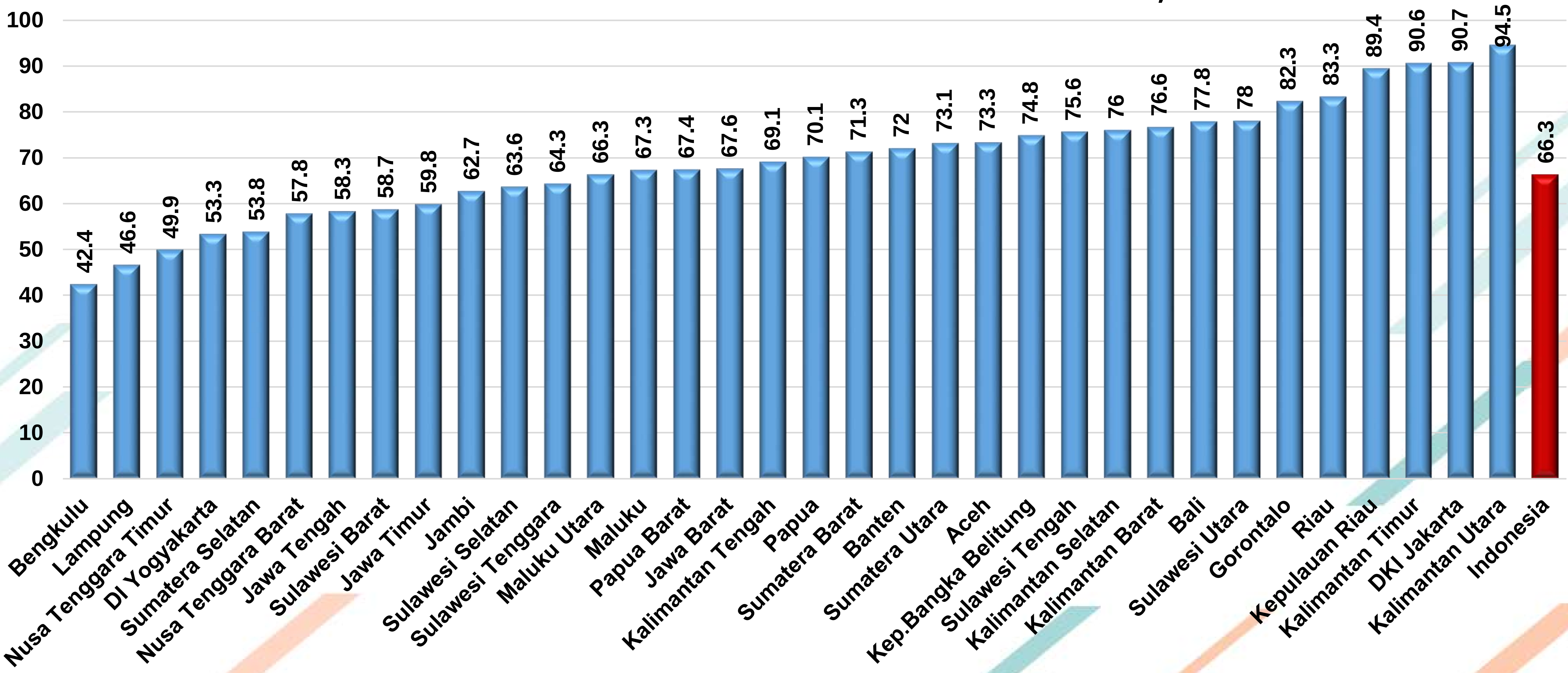

- Memperhitungkan jarak sumur gali terlindung, mata air terlindung, sumur bor atau sumber pompa ≥ 10 meter dari sumber pencemaran

PROPORSI RUMAH TANGGA YANG MEMILIKI AKSES SANITASI LAYAK MENURUT PROVINSI, SSGI 2021

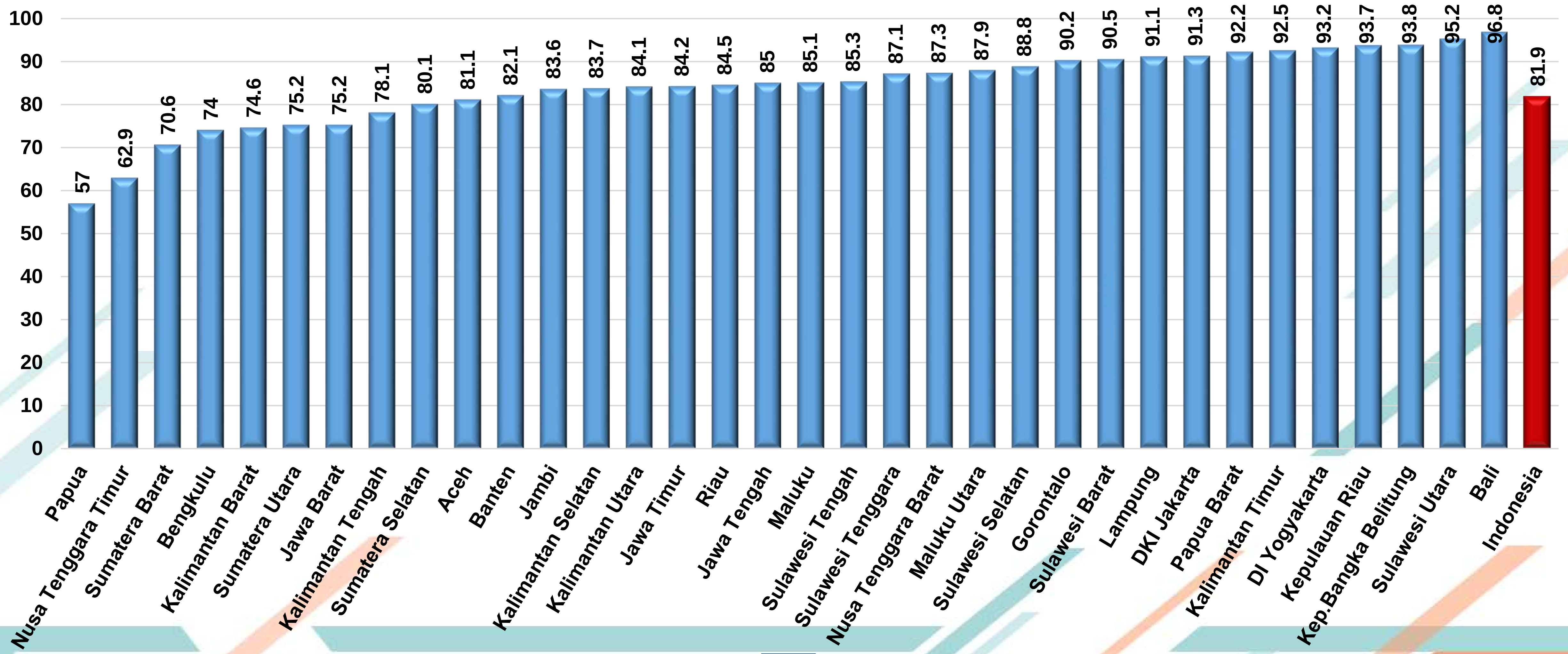

PRESENTASE BALITA STUNTED MENURUT KUINTIL ASET KEPEMILIKAN DAN PROVINSI, SSGI 2021

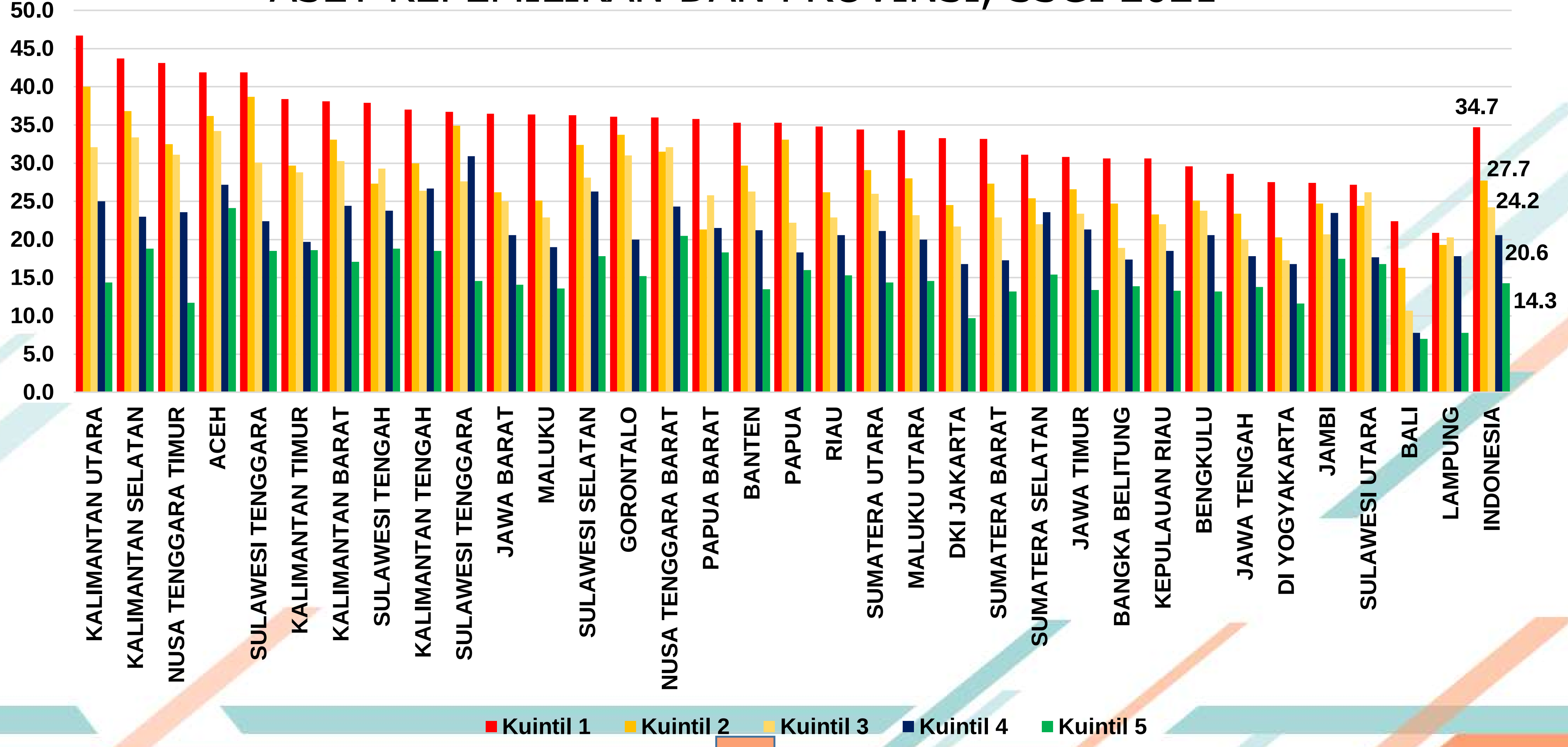

KESIMPULAN

Dari 34 Provinsi :

- **Satu provinsi** dengan kategori **Baik (Stunted<20% dan Wasted<5%)** yaitu Provinsi **Bali**
- **Lima provinsi** yang mempunyai masalah gizi dengan kategori **Akut (Stunted<20% dan Wasted ≥ 5%)** yaitu **Lampung, Kep. Bangka Belitung, Kep. Riau, DKI Jakarta dan DI Yogyakarta**
- **Satu provinsi** termasuk kategori **Kronis** (**Stunted ≥ 20% dan Wasted<5%**) yaitu Provinsi **Bengkulu**
- **27 Provinsi** termasuk kategori **Kronis-Akut** (**Stunted ≥ 20% dan Wasted ≥ 5%**)

REKOMENDASI

1. **Pencapaian rata-rata pertahun penurunan stunting sebesar 2,0% (2013 – 2021) dengan angka prevalensi stunting tahun 2021 sebesar 24,4%. Perlu upaya inovasi dalam pencapaian 2,7% pertahun agar mencapai target 14% (target RPJMN) dengan ketepatan intervensi yang dilakukan.**

2. **27 Provinsi, masalah gizi bersifat Akut-Kronis. Upaya konvergensi harus sudah mulai menuju kualitas intervensi berimbang di dua intervensi utama yaitu intervensi spesifik dan intervensi sensitif.**

3. **Antisipasi kedepan dengan meningkatkan pemantauan pertumbuhan balita di posyandu maupun di faskes lainnya, karena prevelensi *underweight (*Berat badan menurut Umur*)* terjadi kenaikan hampir 1%. Ini terdampak adanya pandemic COVID-19 sehingga standar pemantauan pertumbuhan balita (≥ 8 kali) rendah secara nasional sekitar 39%.**

KEMENTERIAN KESEHATAN REPUBLIK INDONESIA

SSGI
Studi Status Gizi Indonesia

Definisi Operasional Indikator

No	Indikator	Definisi Operasional
1	Kepemilikan Jaminan Pelayanan Kesehatan	Persentase anggota rumah tangga yang memiliki JKN/Jamkesda/Jaminan kesehatan lainnya terhadap seluruh seluruh anggota rumah tangga
2	Balita sakit periksa ke Fasyankes	Persentase balita yang melakukan pengobatan saat sakit terhadap seluruh balita dalam 12 bulan terakhir
3	Memiliki buku KIA	Persentase balita yang memiliki buku KIA terhadap seluruh balita
4	Penimbangan sesuai standar ≥ 8 kali setahun	Persentase Balita yang melakukan penimbangan berat badan sesuai standar (minimal 8 kali dalam setahun) terhadap seluruh Balita dalam 12 bulan terakhir
5	Pengukuran panjang/tinggi badan sesuai standar ≥ 2 kali	Persentase Balita yang melakukan pengukuran Panjang/tinggi badan sesuai standar (minimal 2 kali dalam setahun) terhadap seluruh Balita dalam 12 bulan terakhir
6	Imunisasi Dasar lengkap	Persentase anak usia 12-23 bulan yang mendapat imunisasi lengkap (HB0 1 kali, BCG 1 kali, DPT-HIB 3 kali, OPV 4 kali+ 1 IPV atau IPV 3 kali dan campak 1 kali) terhadap seluruh anak umur 12-23 bulan

KEMENTERIAN KESEHATAN REPUBLIK INDONESIA

SSGI
Studi Status Gizi Indonesia

7	Pemberian vitamin A	Persentase balita yang mendapat vitamin A dosis tinggi sesuai standar (1 kali pada anak usia 6-11 bulan atau 2 kali pada anak usia 12-59 bulan) terhadap seluruh balita dalam 12 bulan terakhir
8	Inisiasi Menyusu Dini (IMD)	Persentase anak usia 0-23 bulan yang dilakukan inisiasi menyusu dini sesaat setelah dilahirkan selama 1 jam tanpa penghalang (skin to skin) terhadap seluruh anak usia 0-23 bulan
9	Balita masih disusui	Persentase anak usia 0-23 bulan yang masih disusui terhadap seluruh anak umur 0-23 bulan
10	Berat badan lahir rendah	Persentase balita yang memiliki berat lahir <2500 gram berdasarkan catatan atau ingatan terhadap seluruh balita
11	Panjang badan lahir	Persentase balita yang memiliki Panjang lahir < 48 cm berdasarkan ingatan atau catatan terhadap seluruh balita
12	Ibu hamil mendapat Tablet Tambah Darah	Persentase perempuan usia 10-54 tahun pernah hamil yang mendapat tablet tambah darah (TTD) selama kehamilan terhadap seluruh perempuan 10-54 tahun pernah hamil

KEMENTERIAN KESEHATAN REPUBLIK INDONESIA

SSGI
Studi Status Gizi Indonesia

13	ASI eksklusif 24 jam	Persentase balita 0-5 bulan yang diberikan ASI saja dalam 24 jam terakhir terhadap seluruh balita 0-5 bulan
14	Usia pertama kali diberikan MPASI ≥ 6 bulan	Persentase anak usia ≥ 6 bulan yang diberi MPASI pertama kali pada usia ≥6 bulan terhadap seluruh anak umur ≥ 6 bulan
15	Usia pertama kali diberikan MPASI ≥ 6 bulan	Persentase anak usia ≥ 6 bulan yang diberi MPASI pertama kali pada usia ≥6 bulan terhadap seluruh anak umur ≥ 6 bulan
16	Persalinan dibantu oleh nakes linfaskes	Persentase perempuan usia 10-54 tahun bersalin ditolong oleh dokter kandungan/dokter umum/bidan/perawat terhadap seluruh ibu melahirkan dalam 2 tahun terakhir terhadap seluruh perempuan usia 10-54 tahun
17	Persalinan di fasilitas pelayanan kesehatan	Persentase perempuan usia 10-54 tahun yang melakukan persalinan di fasilitas pelayanan kesehatan (RS, RB/Klinik , Puskesmas, Pustu, Praktik mandiri nakes) terhadap seluruh ibu melahirkan dalam 2 tahun terakhir terhadap seluruh perempuan usia 10-54 tahun

18	Menggunakan KB modern	Persentase perempuan usia 10-54 tahun yang menggunakan alat kontrasepsi modern (Suntik, MOP, MOW, Implan, IUD, Pil, Metode Amenore Lactacy (MAL) terhadap seluruh perempuan usia 10-54 tahun
19	Menggunakan Metode KB Jangka Panjang (MKJP)	Persentase perempuan usia 10-54 tahun yang menggunakan metode kontrasepsi jangka Panjang yaitu AKDR/IUD, AKBK/Implan atau susuk, Tubektomi, Vasekstomi terhadap seluruh perempuan usia 10-54 tahun
20	Akses SAM Layak (Sarana Air Minum dengan jarak 10 ke sumber pencemar)	Persentase rumah tangga yang menggunakan sumber air minum (air kemasan, air ledeng, kran umum, hydrant, hydrant umum, sumur gali terlindung, mata air terlindung, sumur bor atau pompa dengan jarak ke sumber pencemaran >=10 m terhadap seluruh rumah tangga
21	Akses Sanitasi Layak	Persentase rumah tangga yang menggunakan jamban dengan closet leher angsa dan tempat pembuangan tinja IPAL atau septik tanc terhadap seluruh rumah tangga
22	ISPA	Persentase balita yang didiagnosis dan atau mengalami gejala ISPA (demam, batuk, pilek dan atau sakit tenggorokan) terhadap total balita dalam 1 bulan terakhir

KEMENTERIAN KESEHATAN REPUBLIK INDONESIA

SSGI
Studi Status Gizi Indonesia

23	Pneumonia	Persentase balita yang didiagnosis dan atau mengalami gejala Pneumonia (demam tinggi, batuk, kesulitan bernafas atau sesak nafas dan nafas cepat) terhadap seluruh balita dalam 6 bulan terakhir
24	Diare	Persentase balita yang didiagnosis dan atau mengalami gejala Gejala (BAB lebih cair dan lebih 3 kali sehari, dan atau BAB cair tidak bercampur darah) terhadap seluruh balita dalam 1 bulan terakhir
25	Kecacingan	Persentase balita yang didiagnosis dan atau mengalami gejala kecacingan (lemas, sering mengantuk, perut buncit diare dan kehilangan nafsu makan) terhadap seluruh balita dalam 6 bulan terakhir
26	Campak	Persentase balita yang didiagnosis dan atau mengalami gejala Campak (panas, demam, panas tinggi, batuk pilek dan timbul ruam merah [pada seluruh tubuh) terhadap seluruh balita dalam 6 bulan terakhir

ANGKA *STUNTED*
TINGKAT KABUPATEN DAN KOTA

PREVALENSI BALITA *STUNTED* (TINGGI BADAN MENURUT UMUR) BERDASARKAN KABUPATEN/KOTA DI PROVINSI ACEH, SSGI 2021

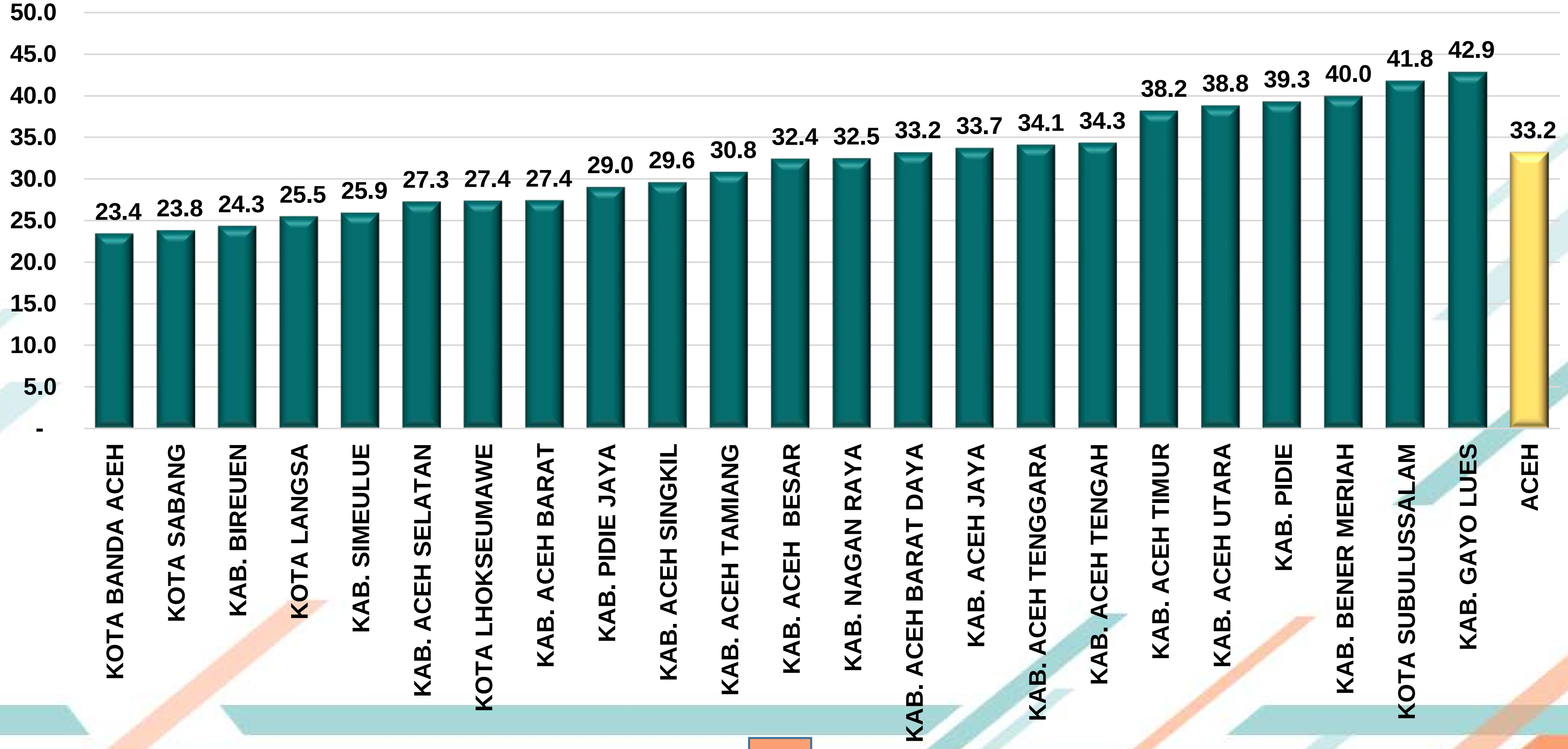

KEMENTERIAN KESEHATAN REPUBLIK INDONESIA

SSGI Studi Status Gizi Indonesia

PREVALENSI BALITA *STUNTED* (TINGGI BADAN MENURUT UMUR) BERDASARKAN KABUPATEN/KOTA DI PROVINSI SUMATERA UTARA, SSGI 2021

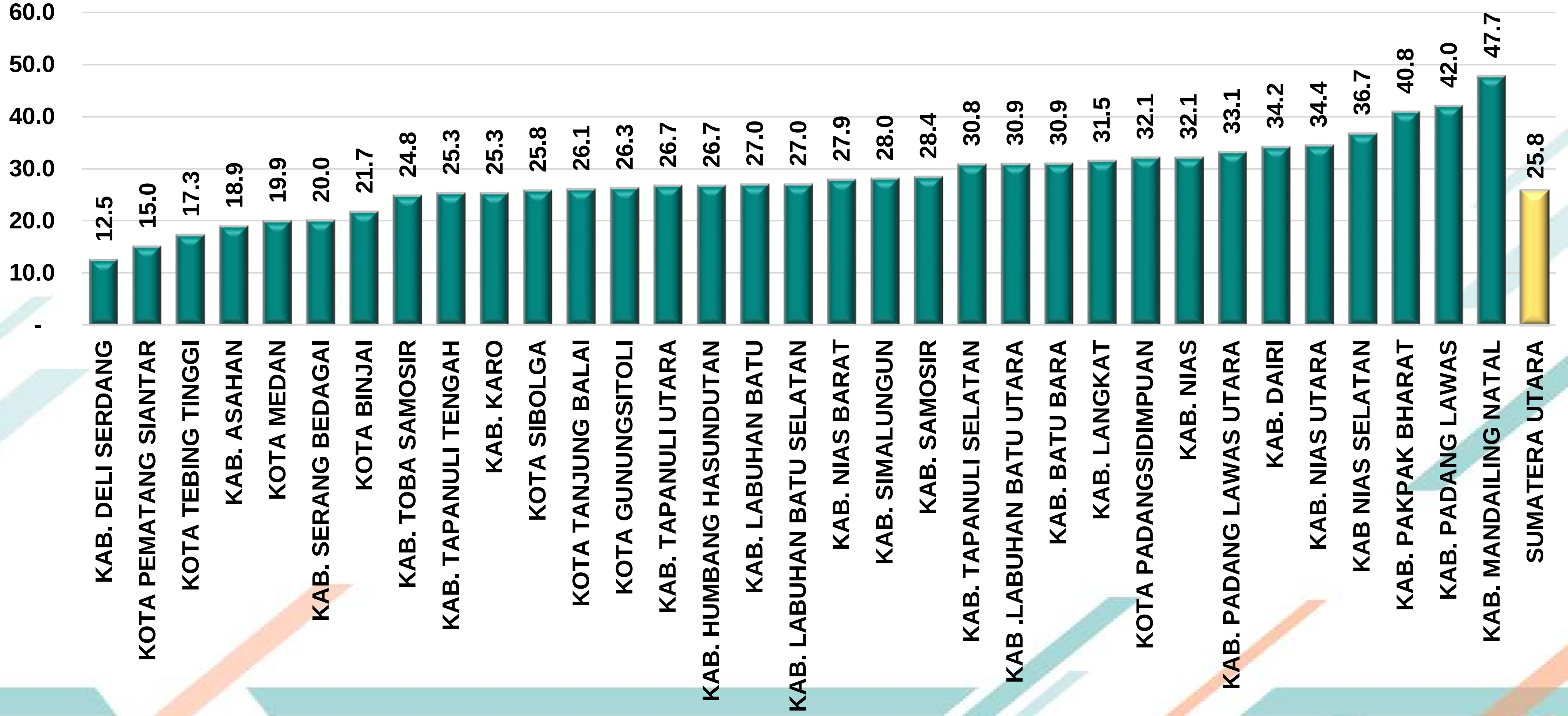

KEMENTERIAN KESEHATAN REPUBLIK INDONESIA

SSGI
Studi Status Gizi Indonesia

PREVALENSI BALITA *STUNTED* (TINGGI BADAN MENURUT UMUR) BERDASARKAN KABUPATEN/KOTA DI PROVINSI SUMATERA BARAT, SSGI 2021

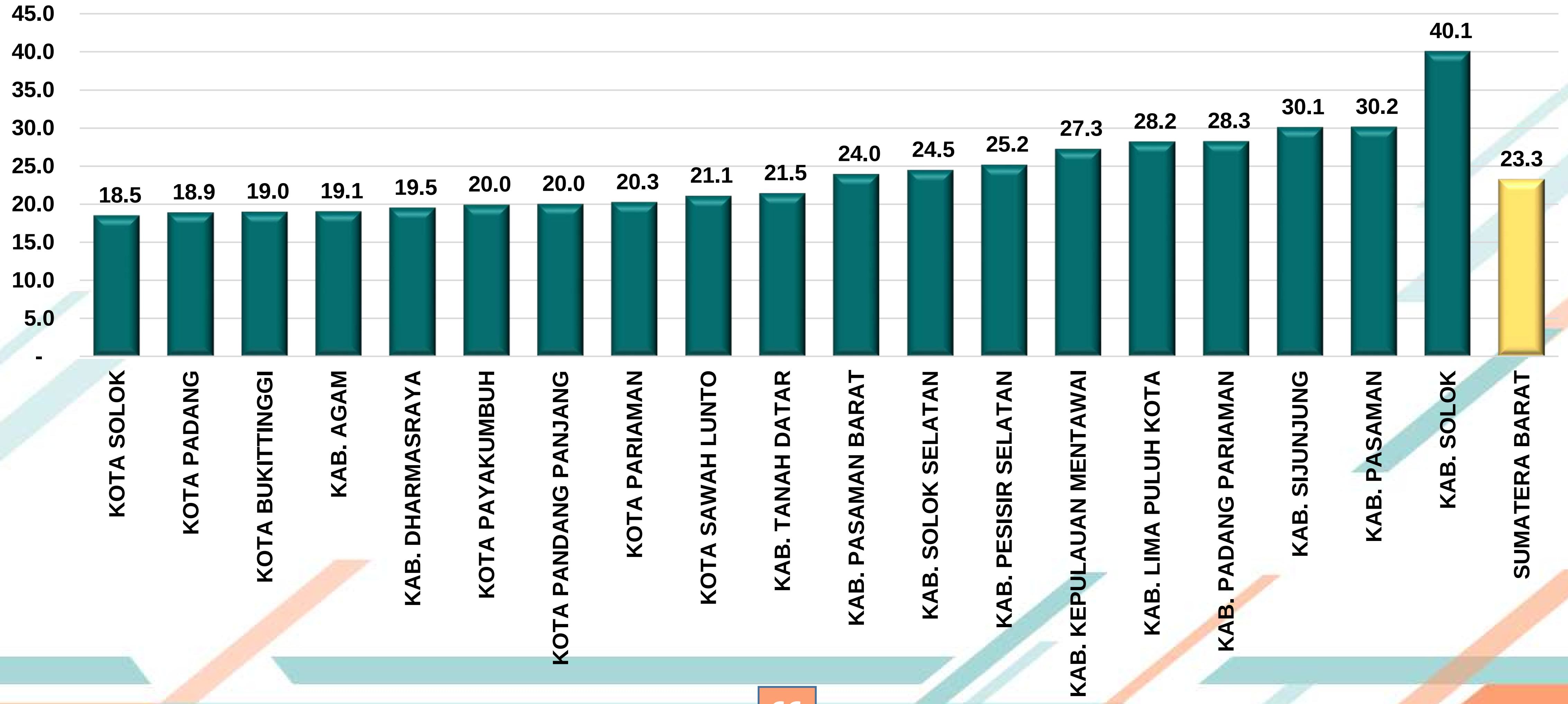

KEMENTERIAN KESEHATAN REPUBLIK INDONESIA

SSGI Studi Status Gizi Indonesia

PREVALENSI BALITA *STUNTED* (TINGGI BADAN MENURUT UMUR) BERDASARKAN KABUPATEN/KOTA DI PROVINSI RIAU, SSGI 2021

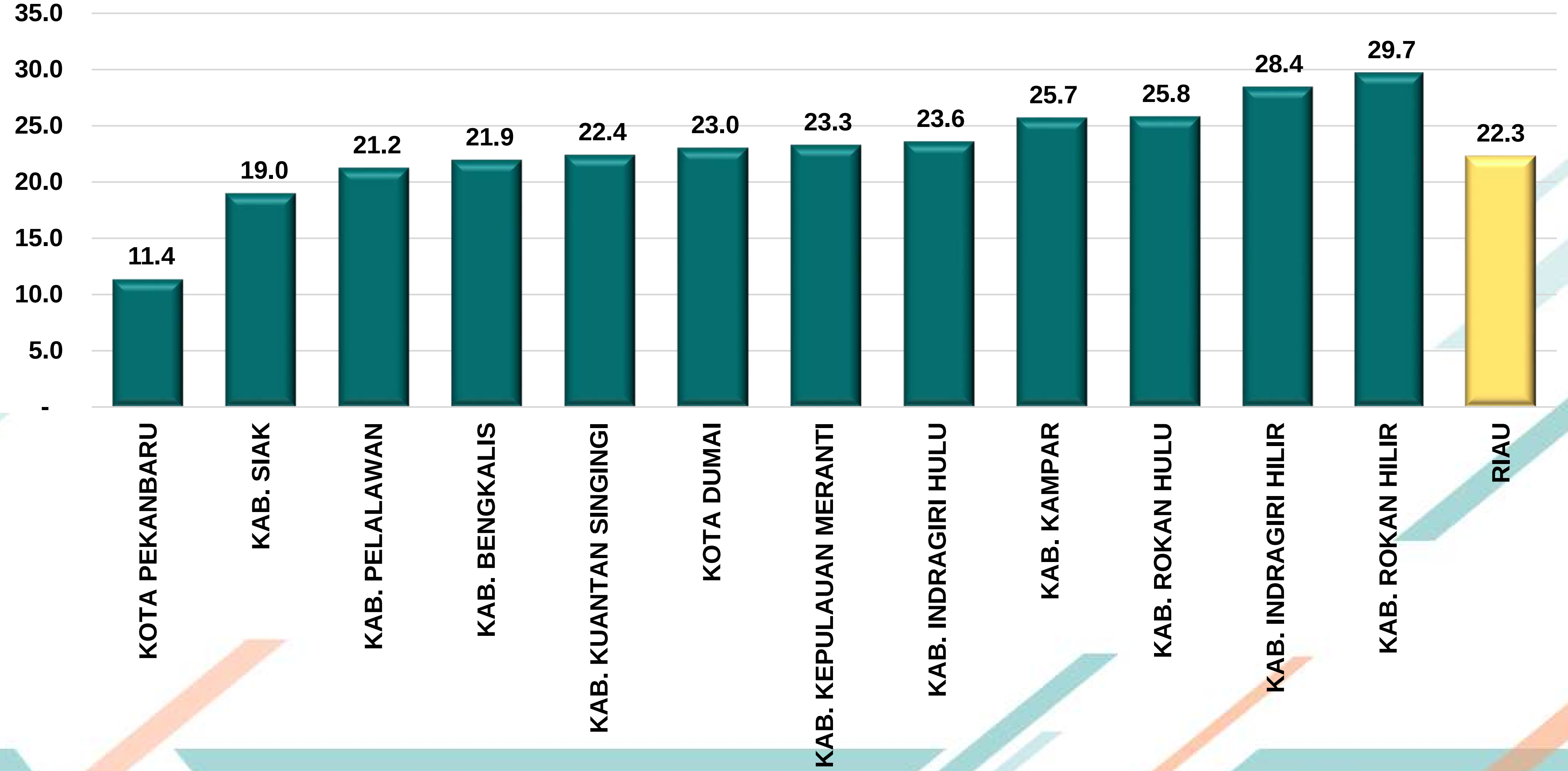

KEMENTERIAN KESEHATAN REPUBLIK INDONESIA

SSGI
Studi Status Gizi Indonesia

PREVALENSI BALITA *STUNTED* (TINGGI BADAN MENURUT UMUR) BERDASARKAN KABUPATEN/KOTA DI PROVINSI JAMBI, SSGI 2021

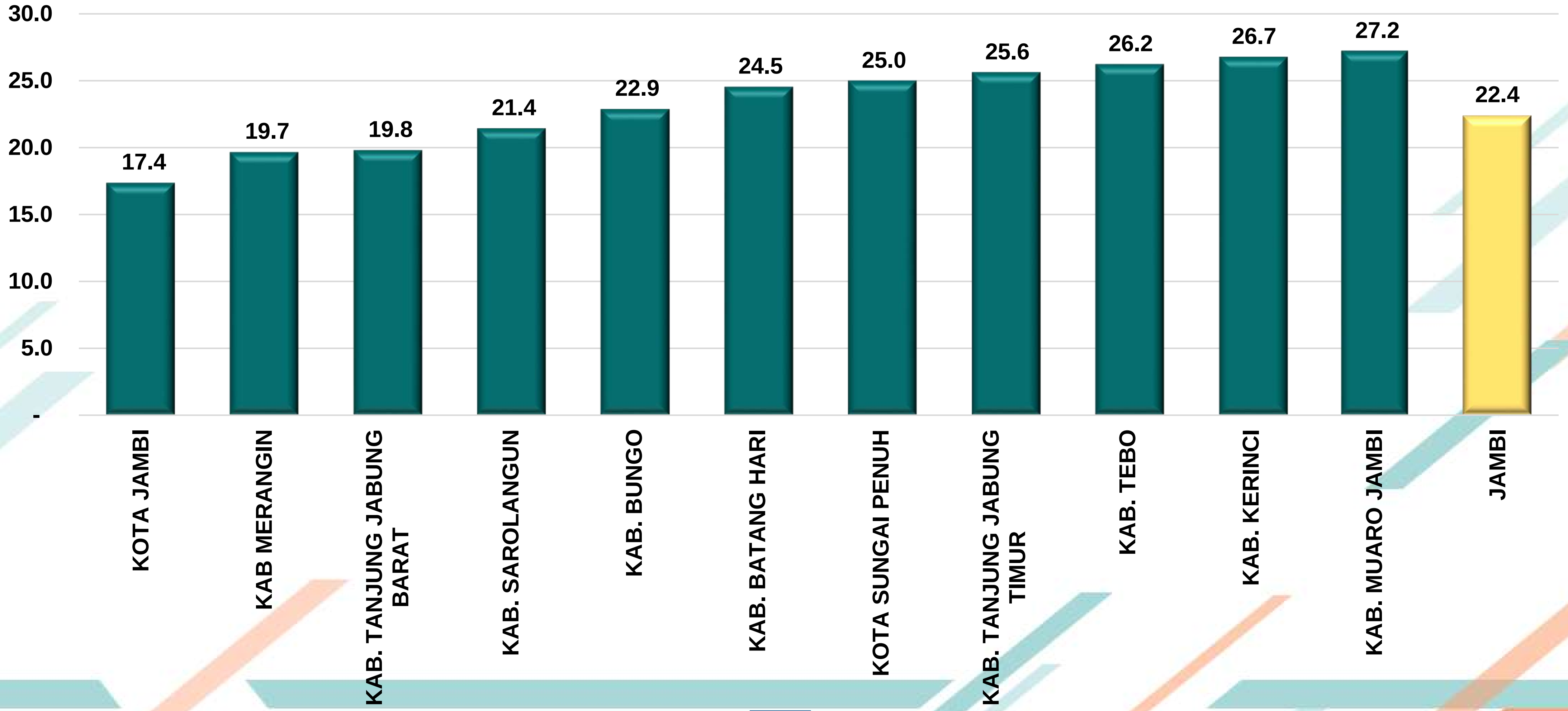

PREVALENSI BALITA *STUNTED* (TINGGI BADAN MENURUT UMUR) BERDASARKAN KABUPATEN/KOTA DI PROVINSI SUMATERA SELATAN, SSGI 2021

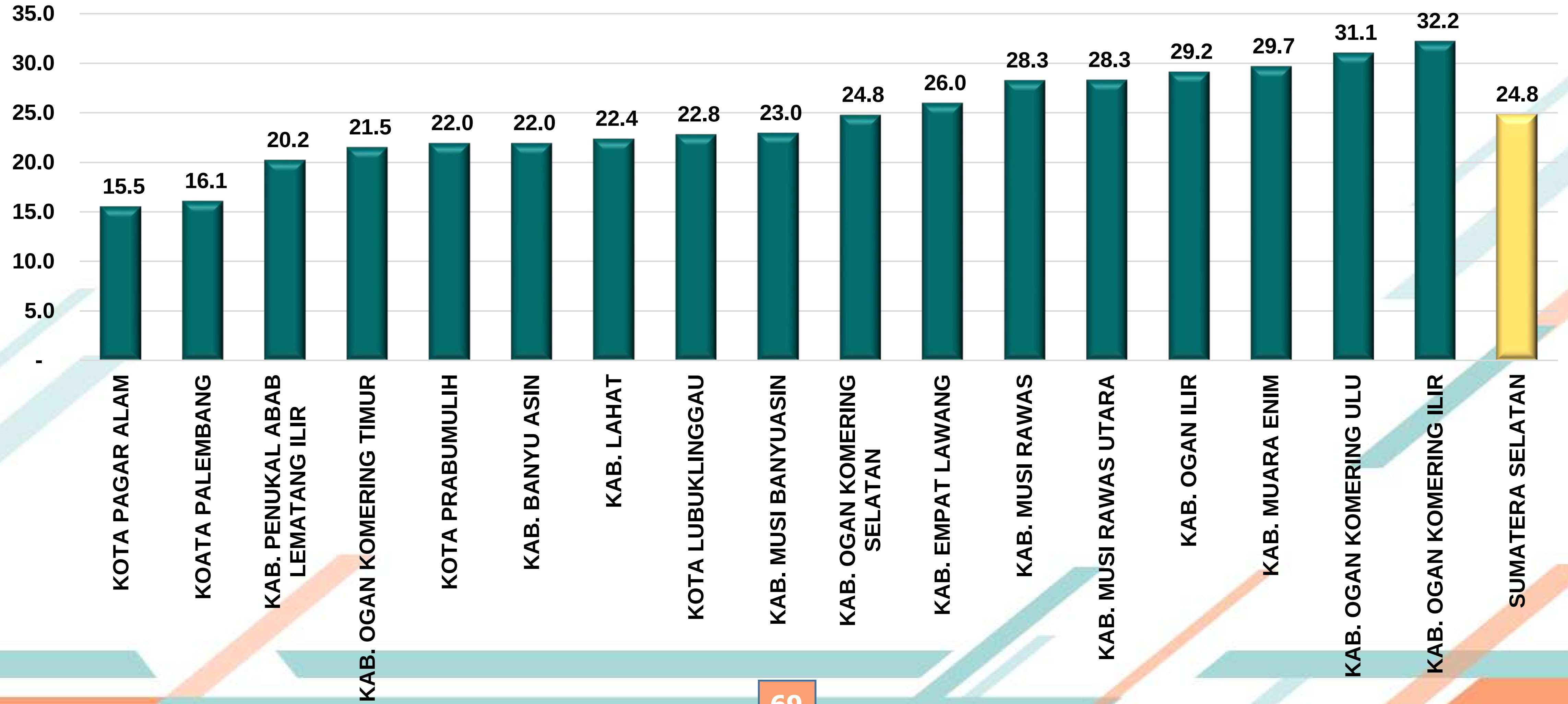

PREVALENSI BALITA *STUNTED* (TINGGI BADAN MENURUT UMUR) BERDASARKAN KABUPATEN/KOTA DI PROVINSI BENGKULU, SSGI 2021

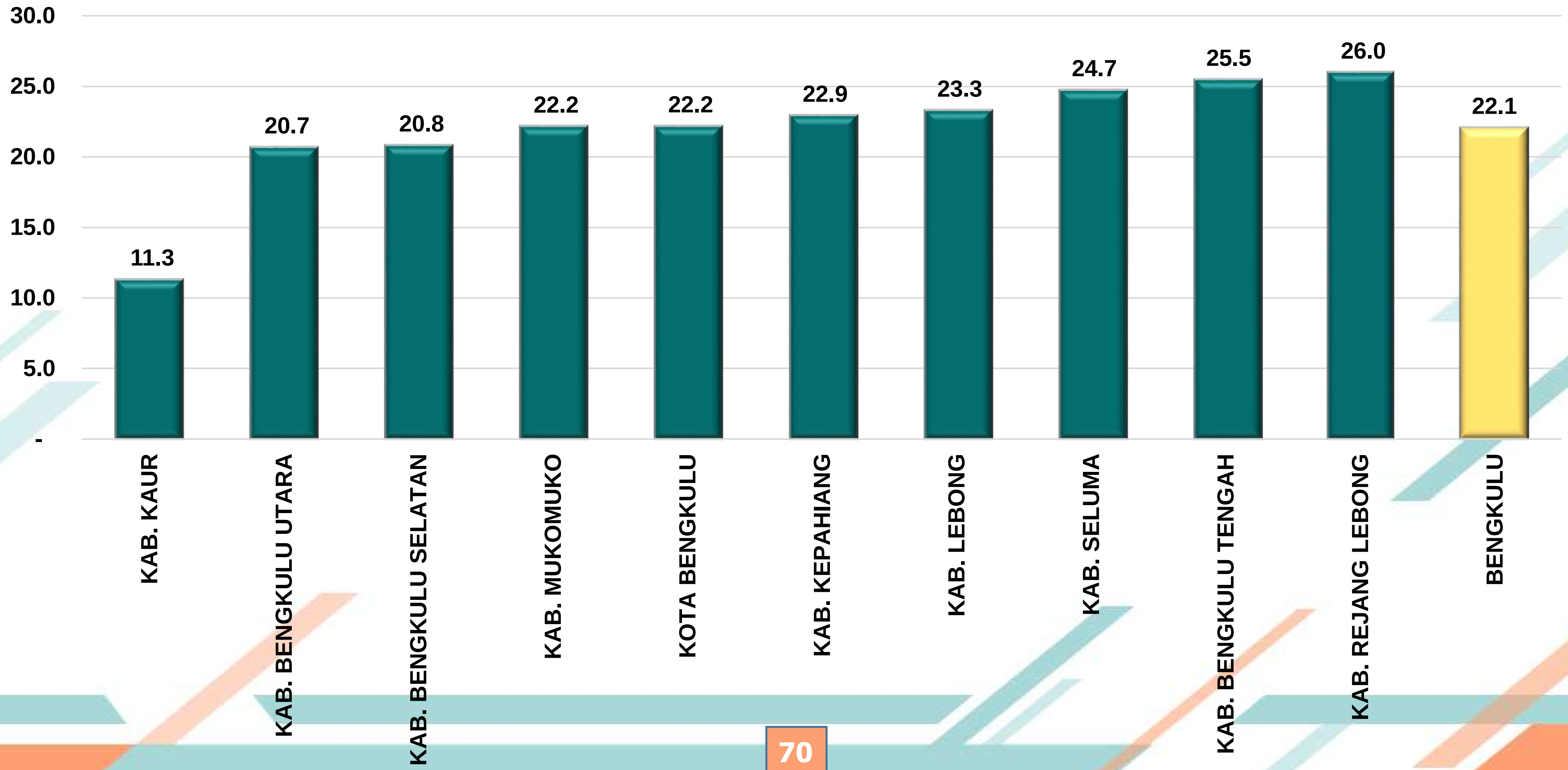

KEMENTERIAN KESEHATAN REPUBLIK INDONESIA

SSGI Studi Status Gizi Indonesia

PREVALENSI BALITA *STUNTED* (TINGGI BADAN MENURUT UMUR) BERDASARKAN KABUPATEN/KOTA DI PROVINSI LAMPUNG, SSGI 2021

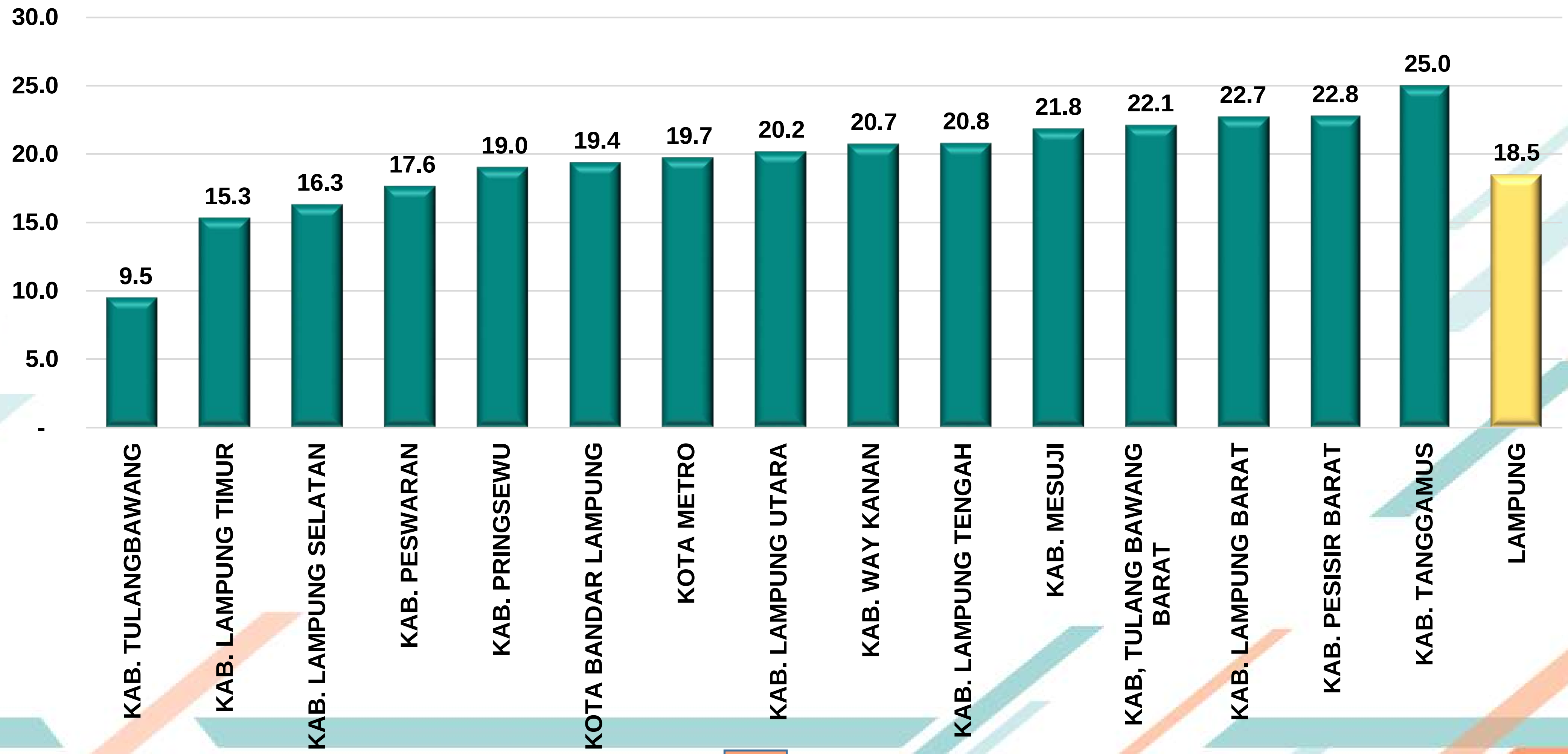

KEMENTERIAN KESEHATAN REPUBLIK INDONESIA

SSGI Studi Status Gizi Indonesia

PREVALENSI BALITA *STUNTED* (TINGGI BADAN MENURUT UMUR) BERDASARKAN KABUPATEN/KOTA DI PROVINSI KEP.BABEL, SSGI 2021

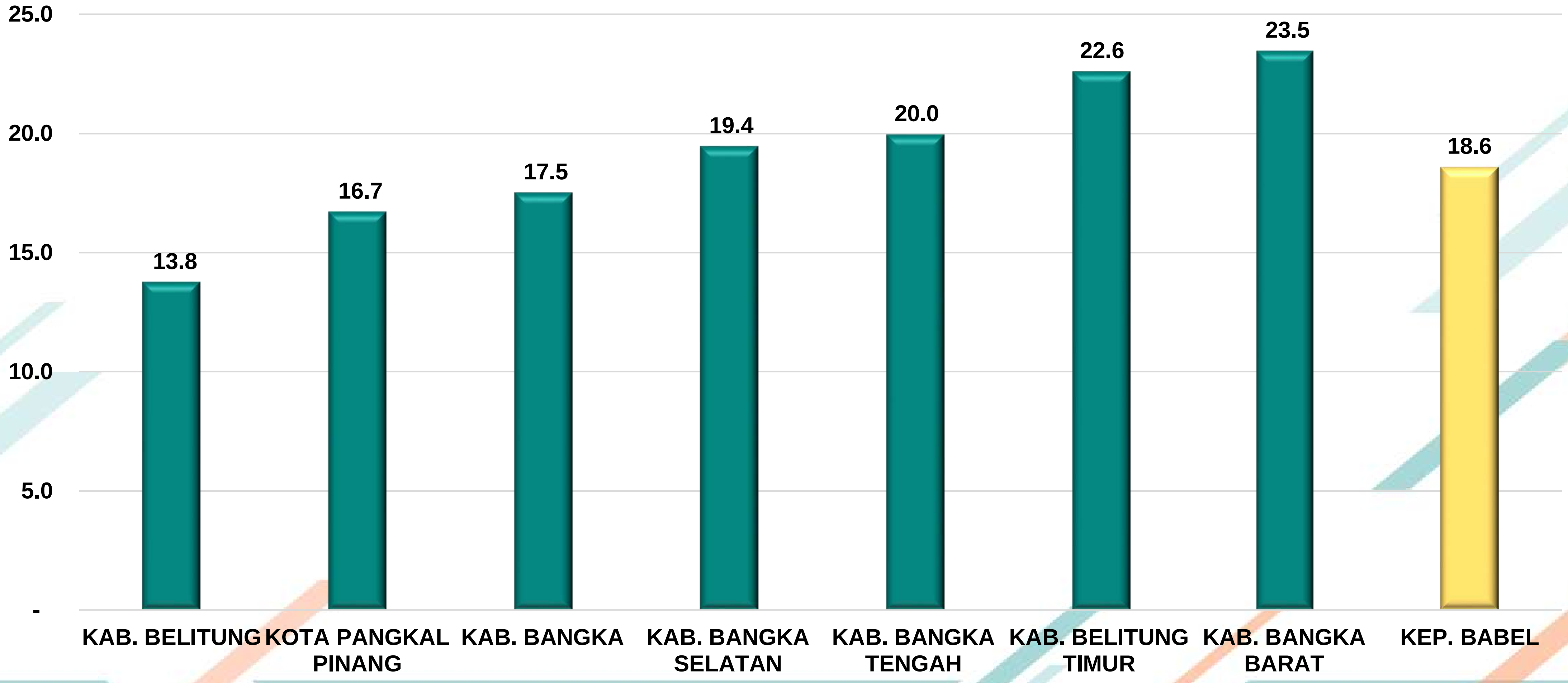

KEMENTERIAN KESEHATAN REPUBLIK INDONESIA

SSGI Studi Status Gizi Indonesia

PREVALENSI BALITA *STUNTED* (TINGGI BADAN MENURUT UMUR) BERDASARKAN KABUPATEN/KOTA DI PROVINSI KEP.RIAU, SSGI 2021

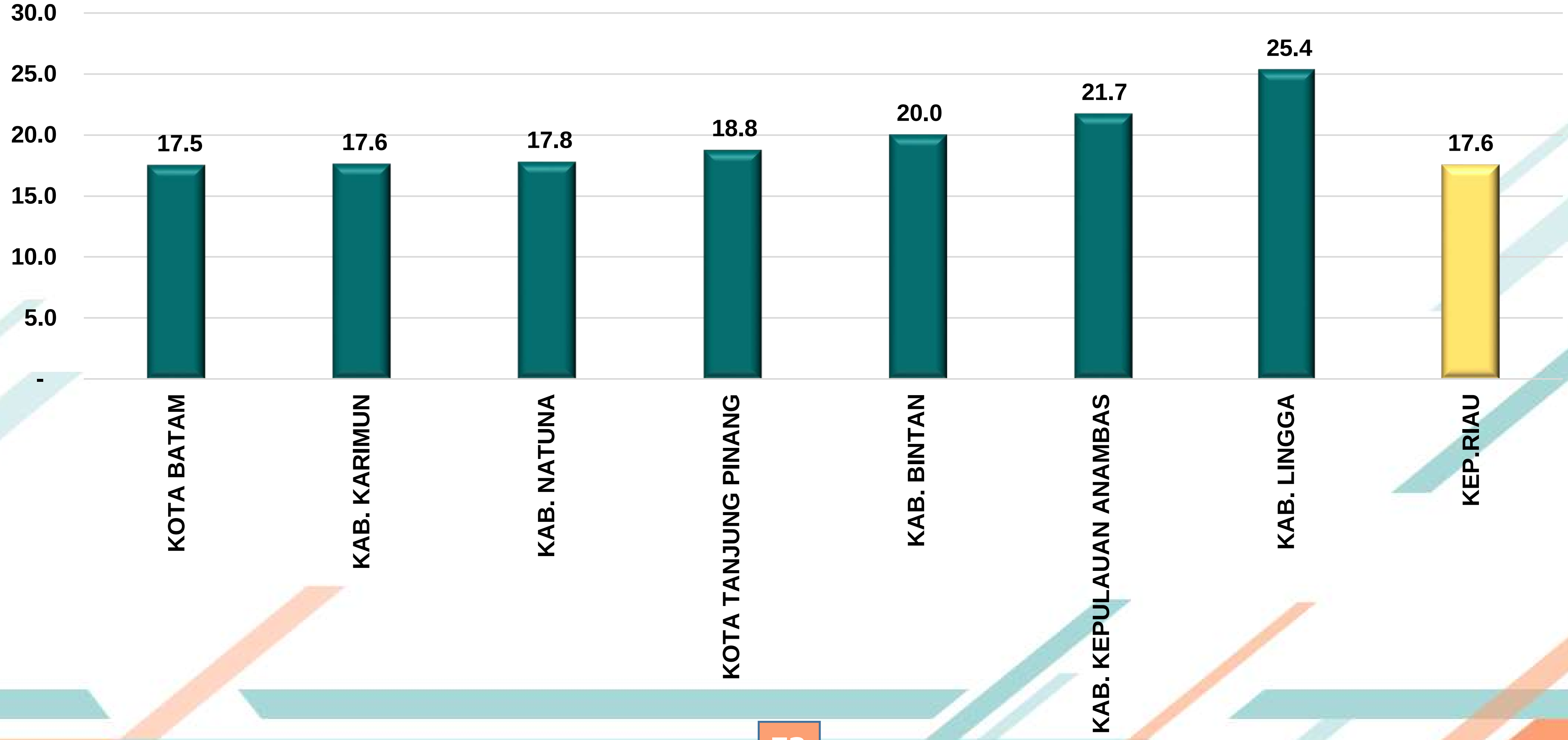

KEMENTERIAN KESEHATAN REPUBLIK INDONESIA

SSGI Studi Status Gizi Indonesia

PREVALENSI BALITA *STUNTED* (TINGGI BADAN MENURUT UMUR) BERDASARKAN KABUPATEN/KOTA DI PROVINSI DKI JAKARTA, SSGI 2021

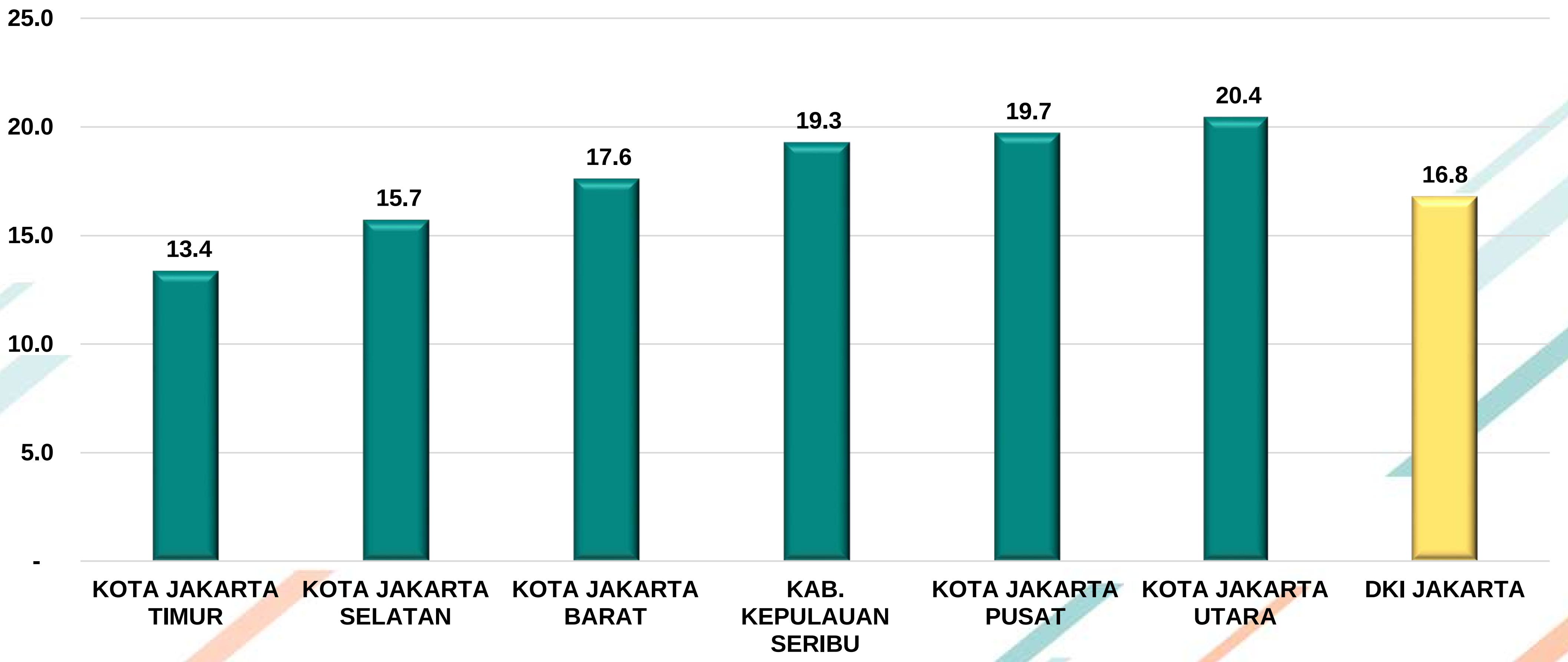

KEMENTERIAN KESEHATAN REPUBLIK INDONESIA

SSGI
Studi Status Gizi Indonesia

PREVALENSI BALITA *STUNTED* (TINGGI BADAN MENURUT UMUR) BERDASARKAN KABUPATEN/KOTA DI PROVINSI JAWA BARAT, SSGI 2021

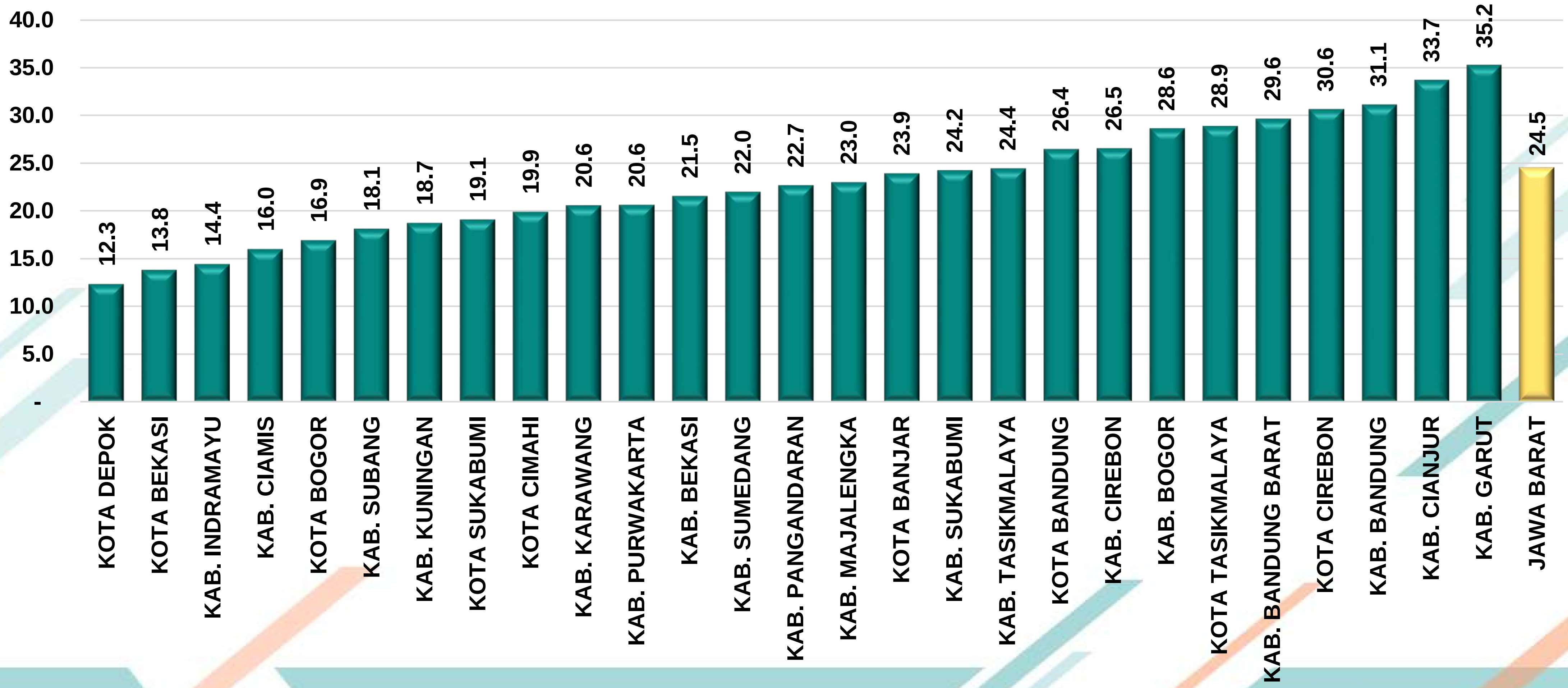

KEMENTERIAN KESEHATAN REPUBLIK INDONESIA

SSGI
Studi Status Gizi Indonesia

PREVALENSI BALITA *STUNTED* (TINGGI BADAN MENURUT UMUR) BERDASARKAN KABUPATEN/KOTA DI PROVINSI JAWA TENGAH, SSGI 2021

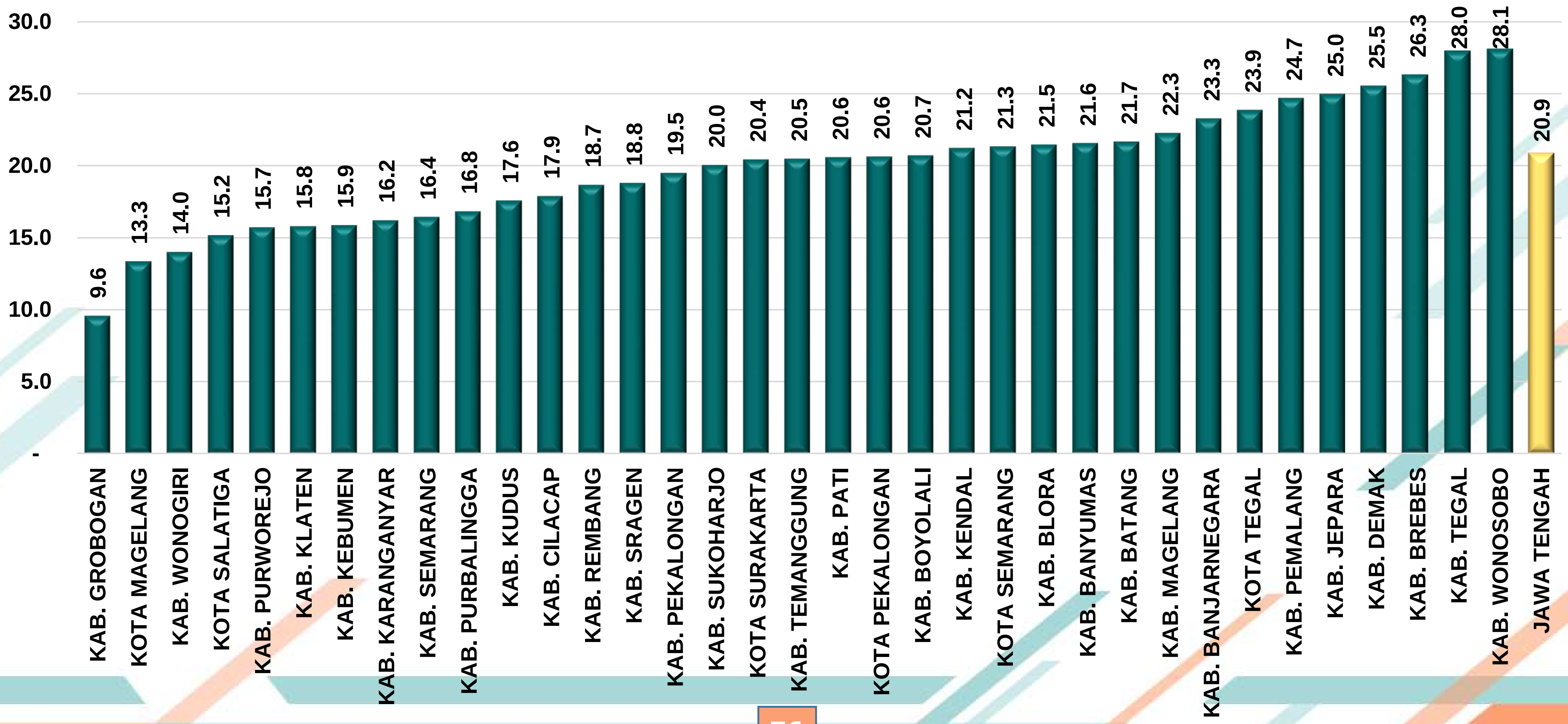

PREVALENSI BALITA *STUNTED* (TINGGI BADAN MENURUT UMUR) BERDASARKAN KABUPATEN/KOTA DI PROVINSI DIY, SSGI 2021

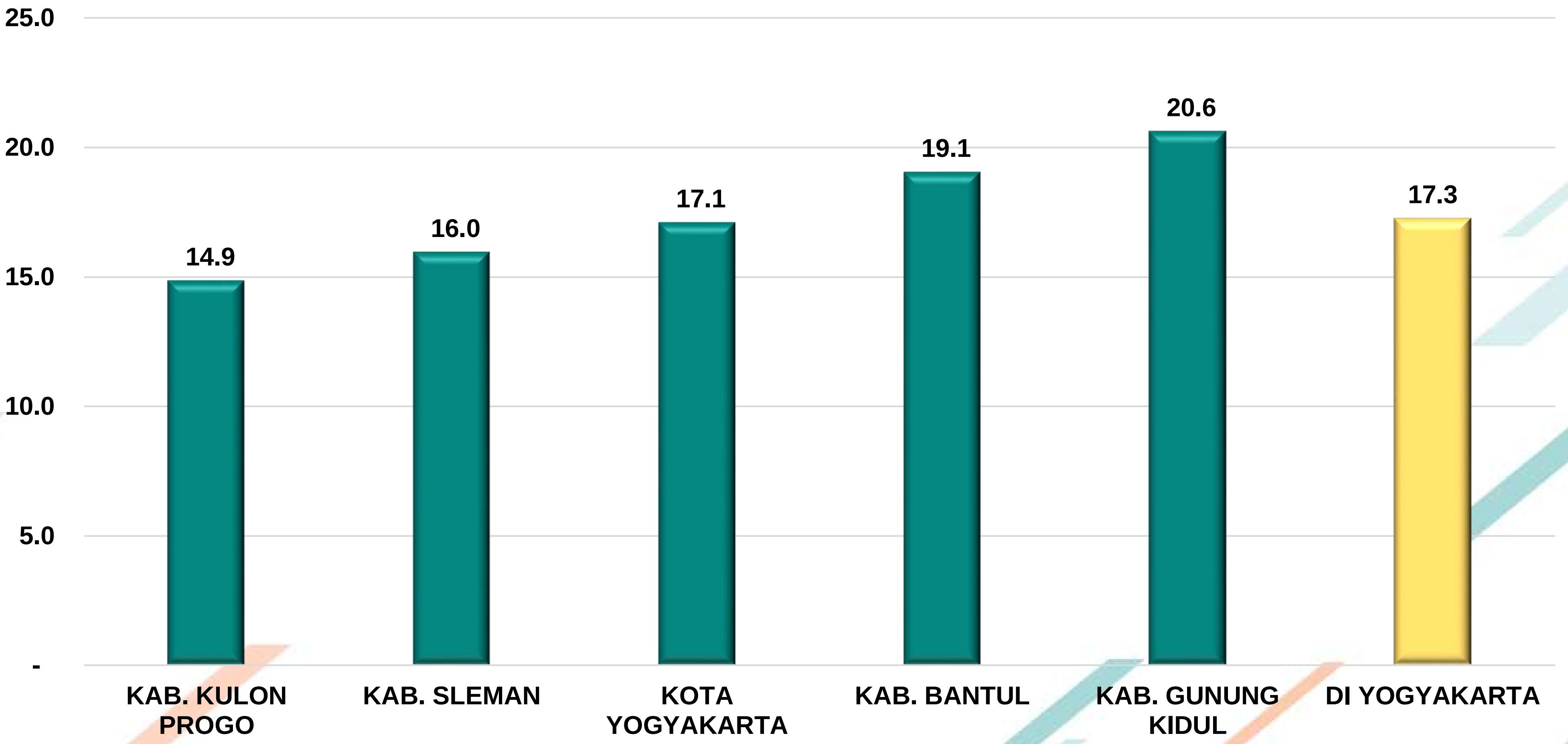

KEMENTERIAN KESEHATAN REPUBLIK INDONESIA

SSGI Studi Status Gizi Indonesia

PREVALENSI BALITA *STUNTED* (TINGGI BADAN MENURUT UMUR) BERDASARKAN KABUPATEN/KOTA DI PROVINSI JAWA TIMUR, SSGI 2021

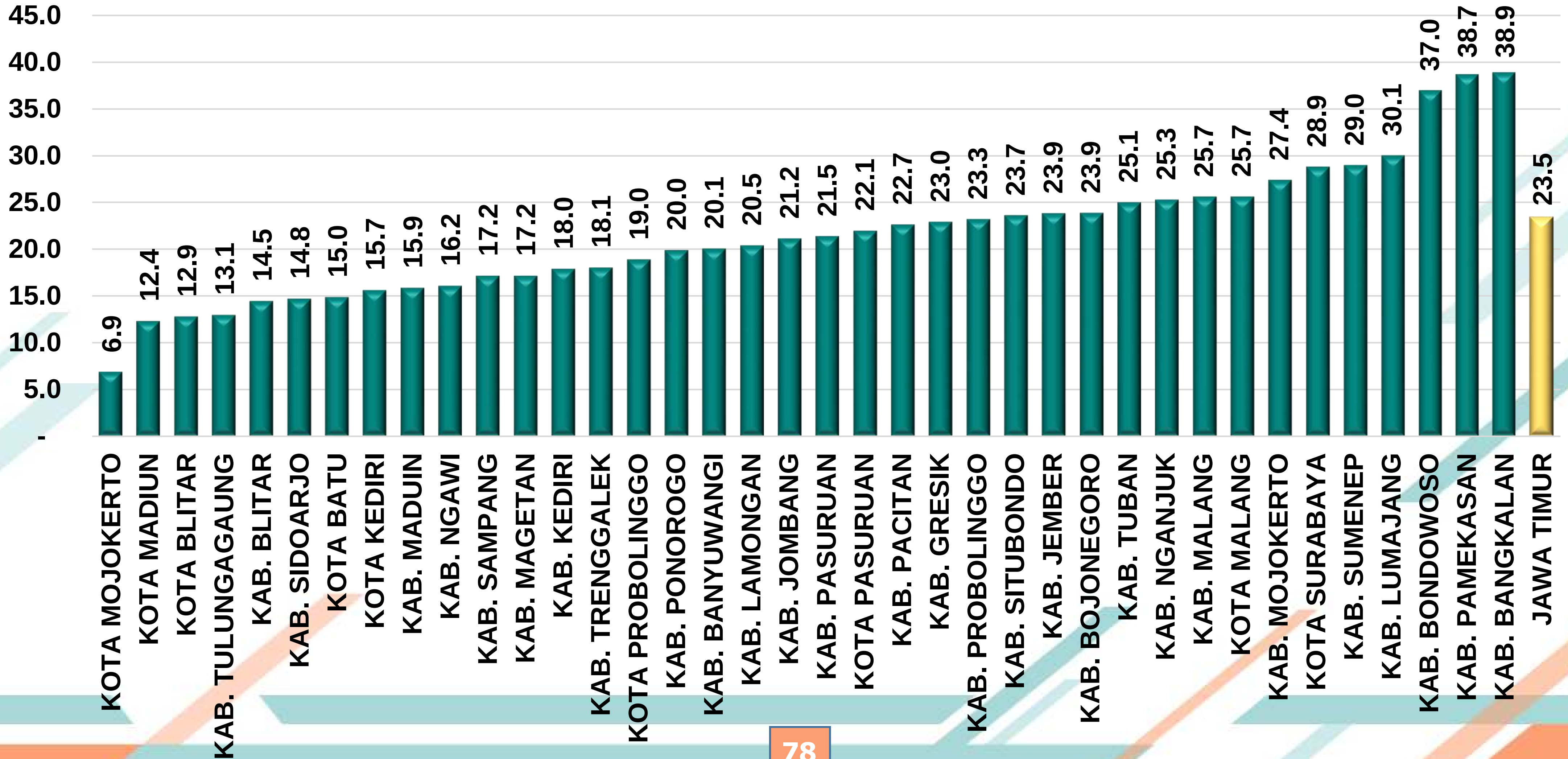

PREVALENSI BALITA *STUNTED* (TINGGI BADAN MENURUT UMUR) BERDASARKAN KABUPATEN/KOTA DI PROVINSI BANTEN, SSGI 2021

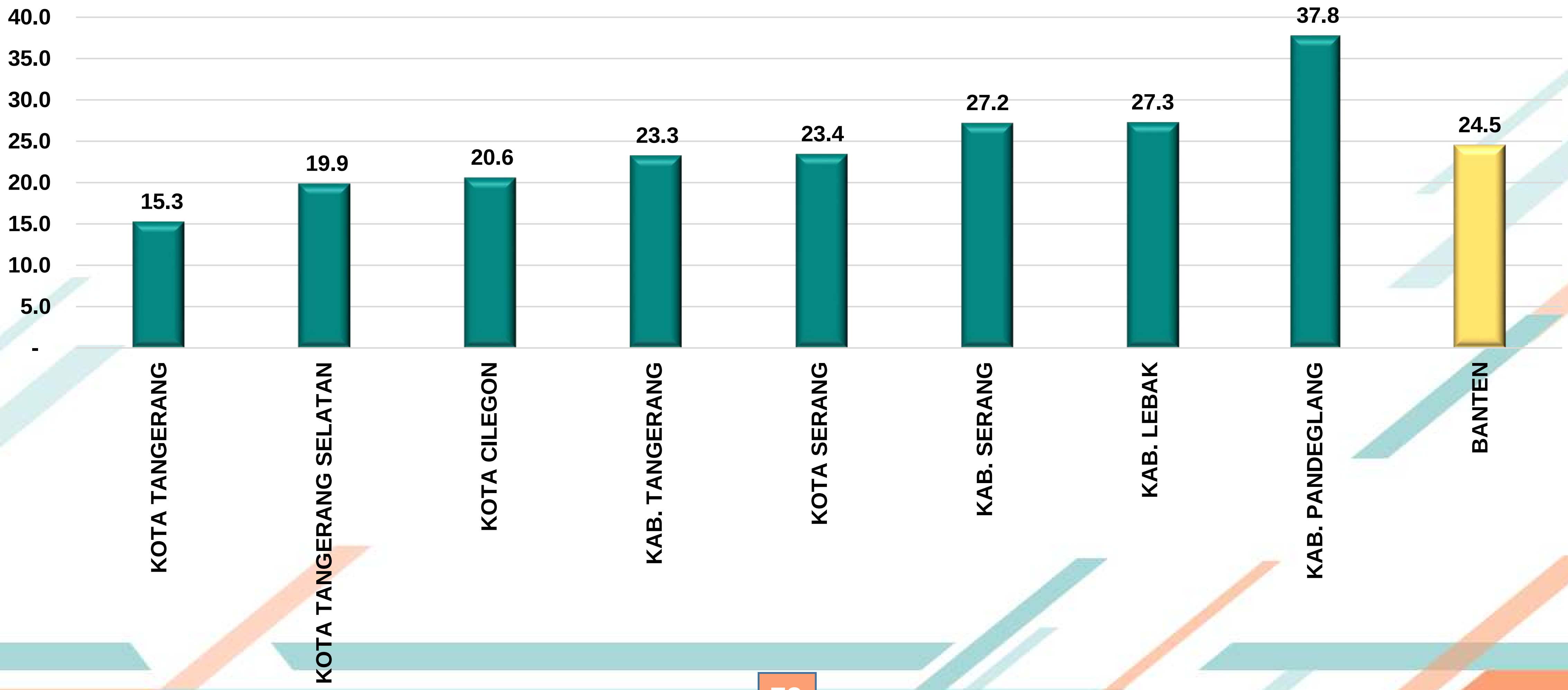

PREVALENSI BALITA *STUNTED* (TINGGI BADAN MENURUT UMUR) BERDASARKAN KABUPATEN/KOTA DI PROVINSI BALI, SSGI 2021

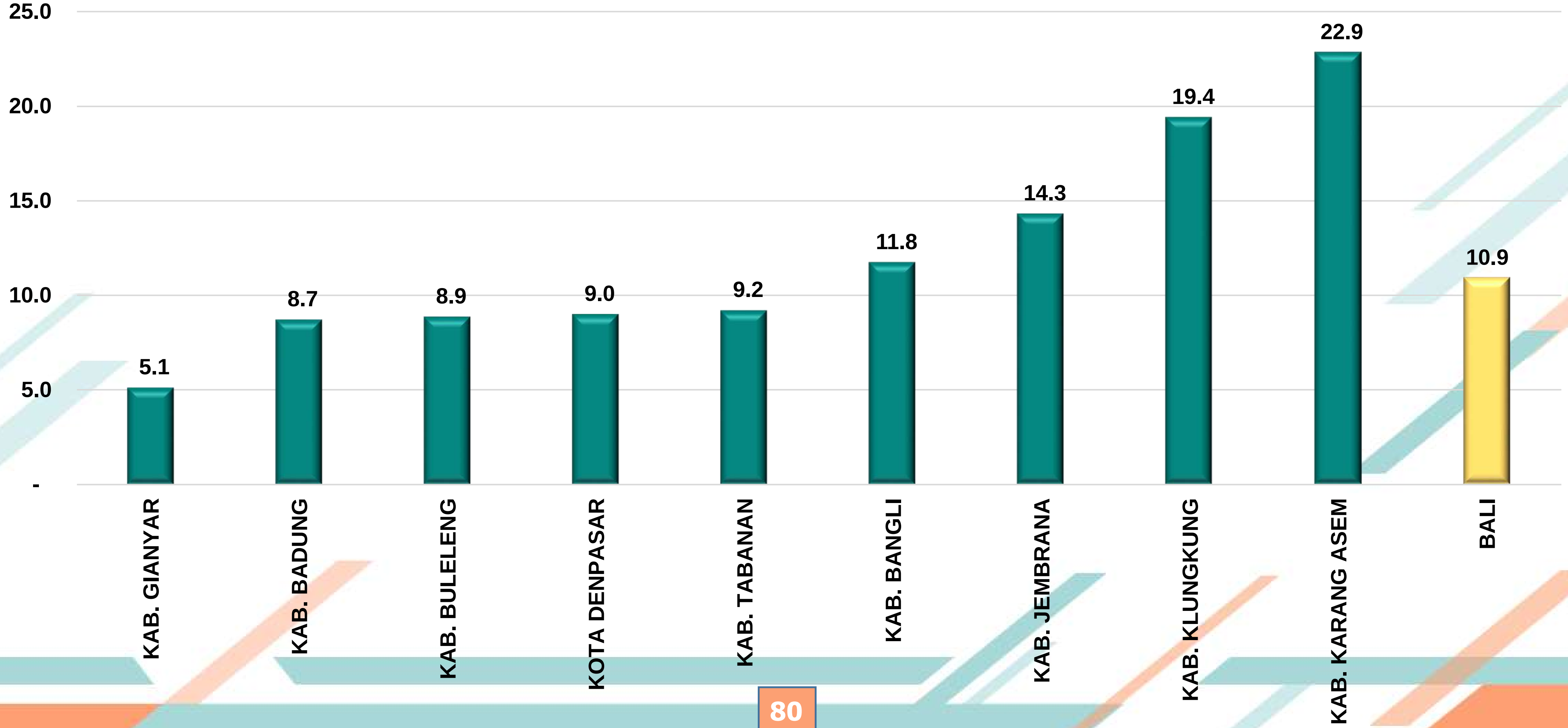

PREVALENSI BALITA *STUNTED* (TINGGI BADAN MENURUT UMUR) BERDASARKAN KABUPATEN/KOTA DI PROVINSI NTB, SSGI 2021

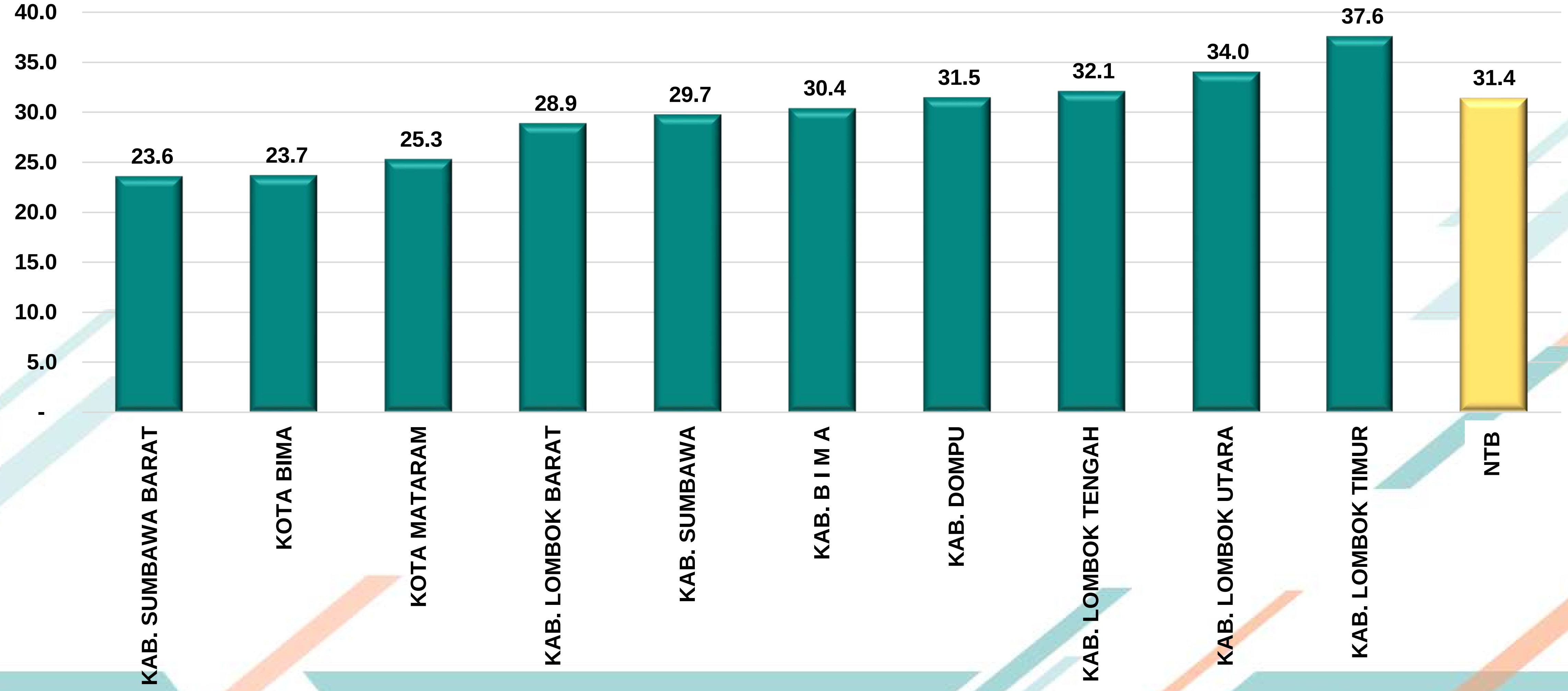

PREVALENSI BALITA *STUNTED* (TINGGI BADAN MENURUT UMUR) BERDASARKAN KABUPATEN/KOTA DI PROVINSI NTT, SSGI 2021

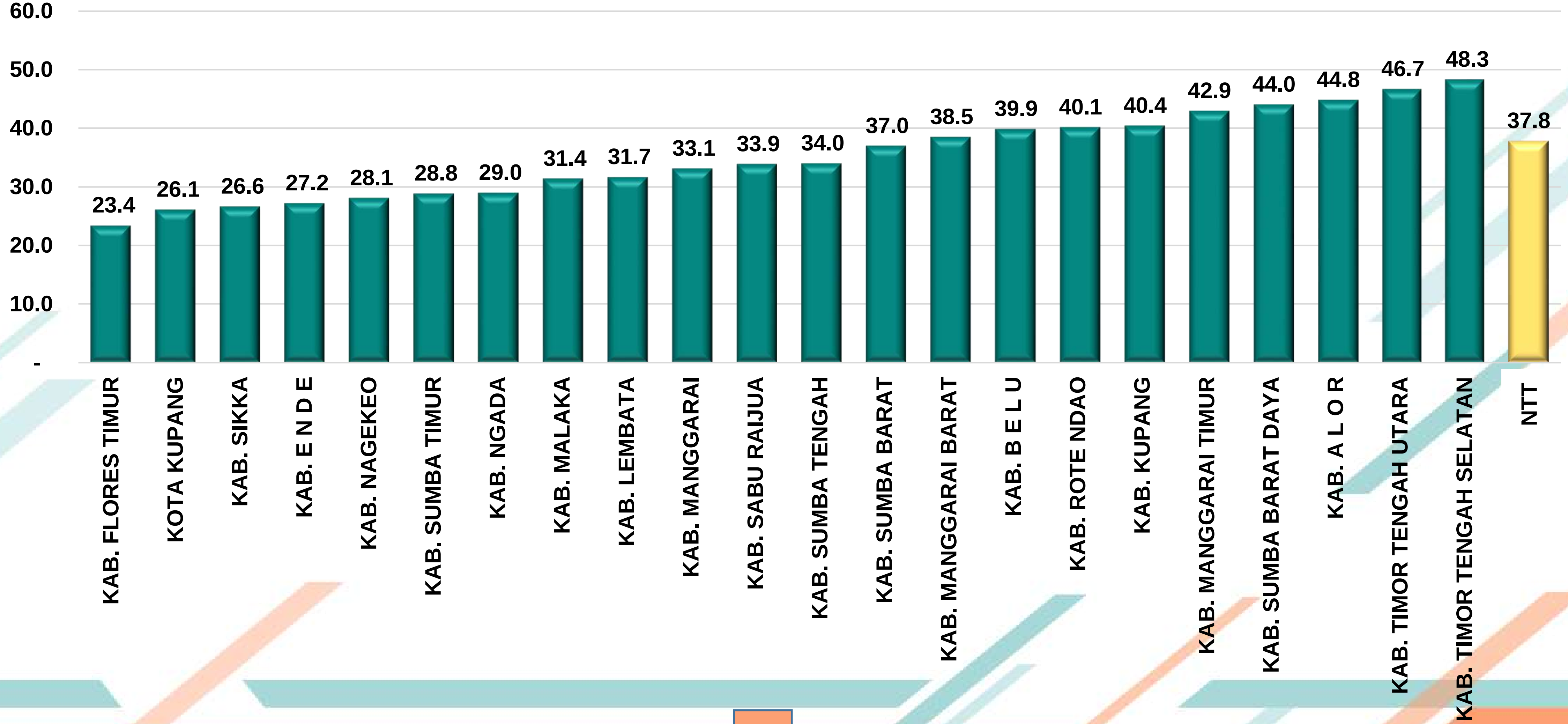

PREVALENSI BALITA *STUNTED* (TINGGI BADAN MENURUT UMUR) BERDASARKAN KABUPATEN/KOTA DI PROVINSI KALIMANTAN BARAT, SSGI 2021

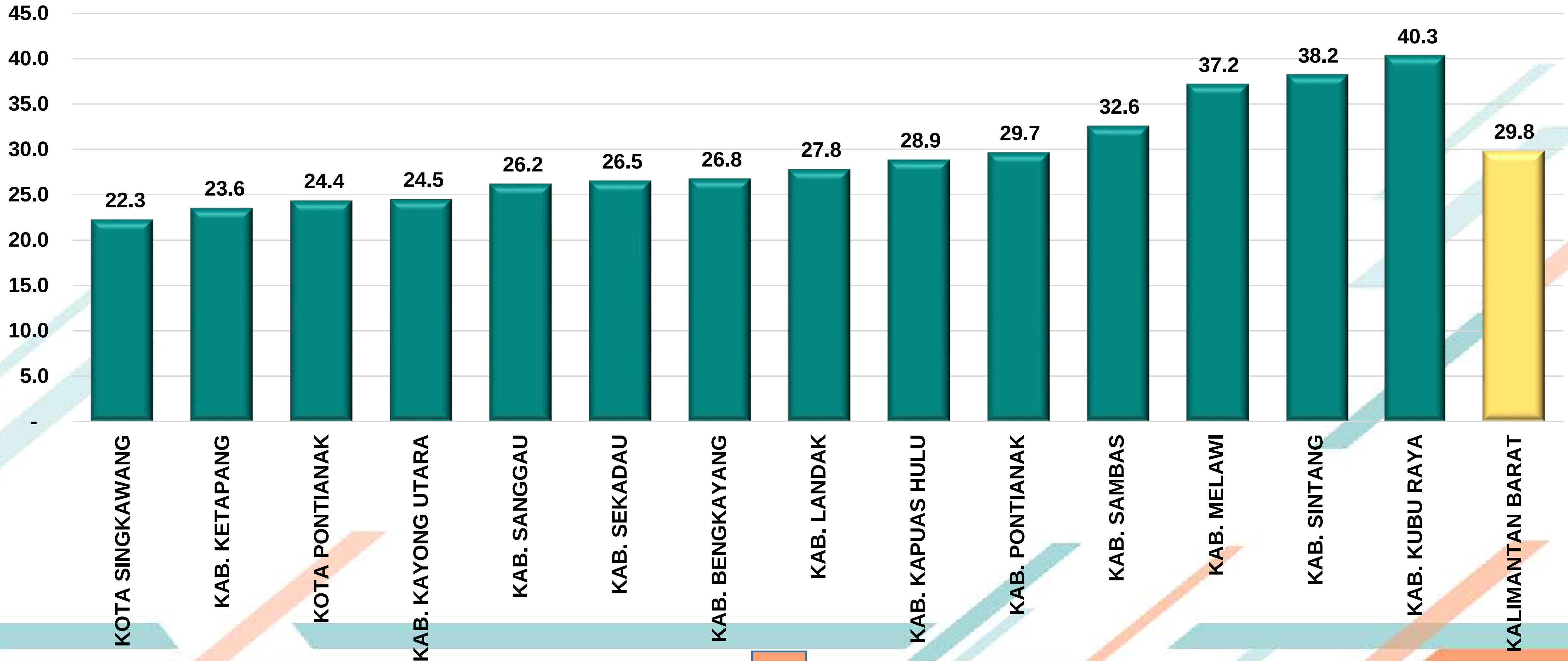

KEMENTERIAN KESEHATAN REPUBLIK INDONESIA

SSGI Studi Status Gizi Indonesia

PREVALENSI BALITA *STUNTED* (TINGGI BADAN MENURUT UMUR) BERDASARKAN KABUPATEN/KOTA DI PROVINSI KALIMANTAN TENGAH, SSGI 2021

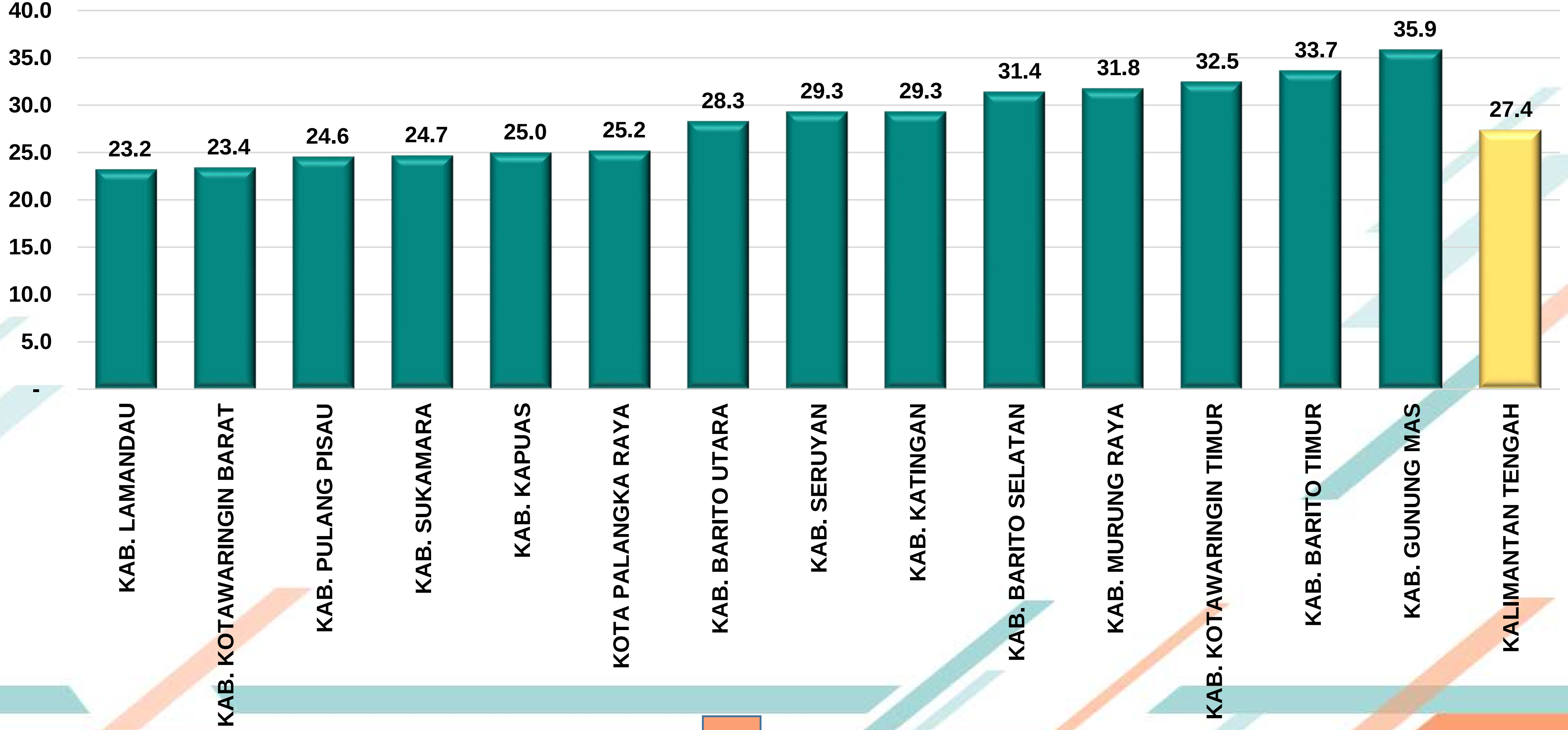

KEMENTERIAN KESEHATAN REPUBLIK INDONESIA

SSGI Studi Status Gizi Indonesia

PREVALENSI BALITA *STUNTED* (TINGGI BADAN MENURUT UMUR) BERDASARKAN KABUPATEN/KOTA DI PROVINSI KALIMANTAN SELATAN, SSGI 2021

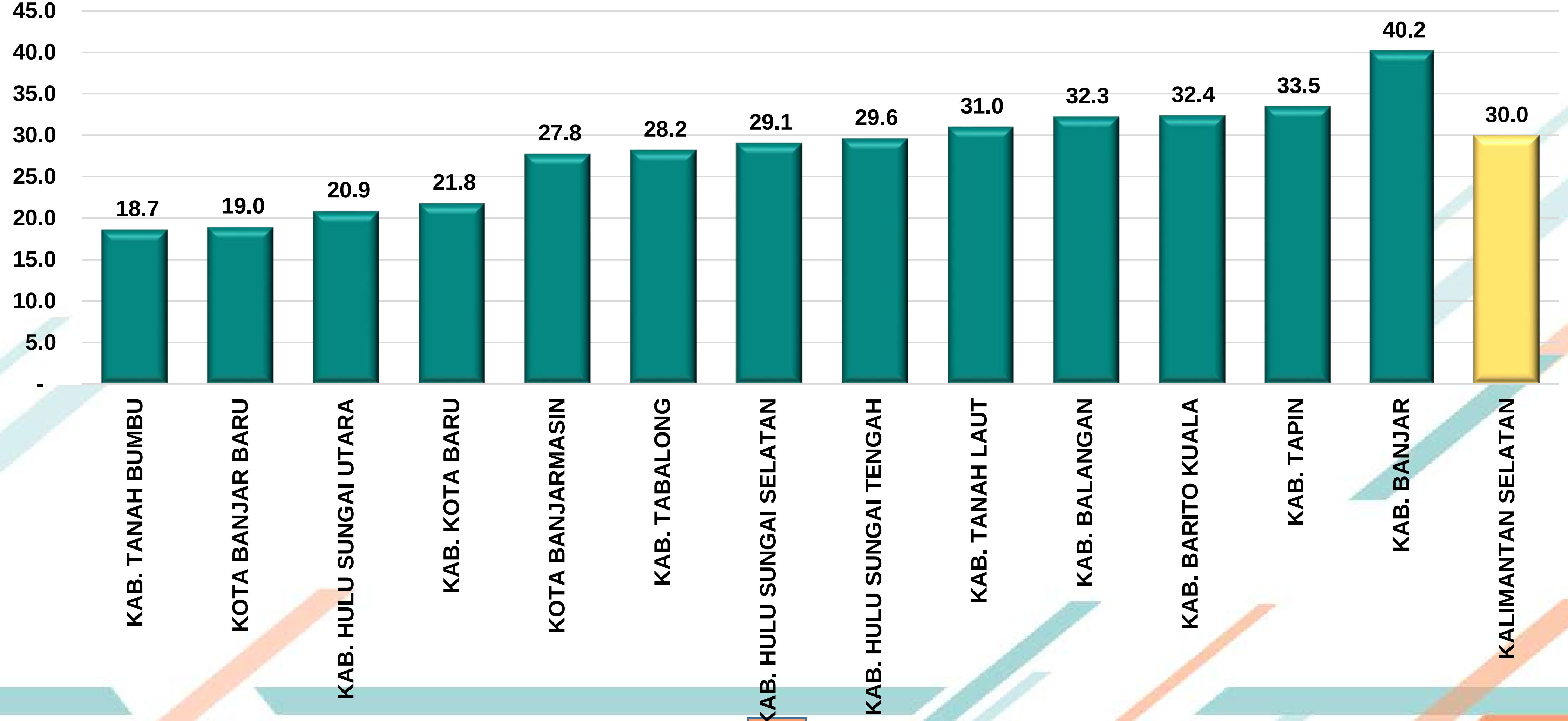

PREVALENSI BALITA *STUNTED* (TINGGI BADAN MENURUT UMUR) BERDASARKAN KABUPATEN/KOTA DI PROVINSI KALIMANTAN TIMUR, SSGI 2021

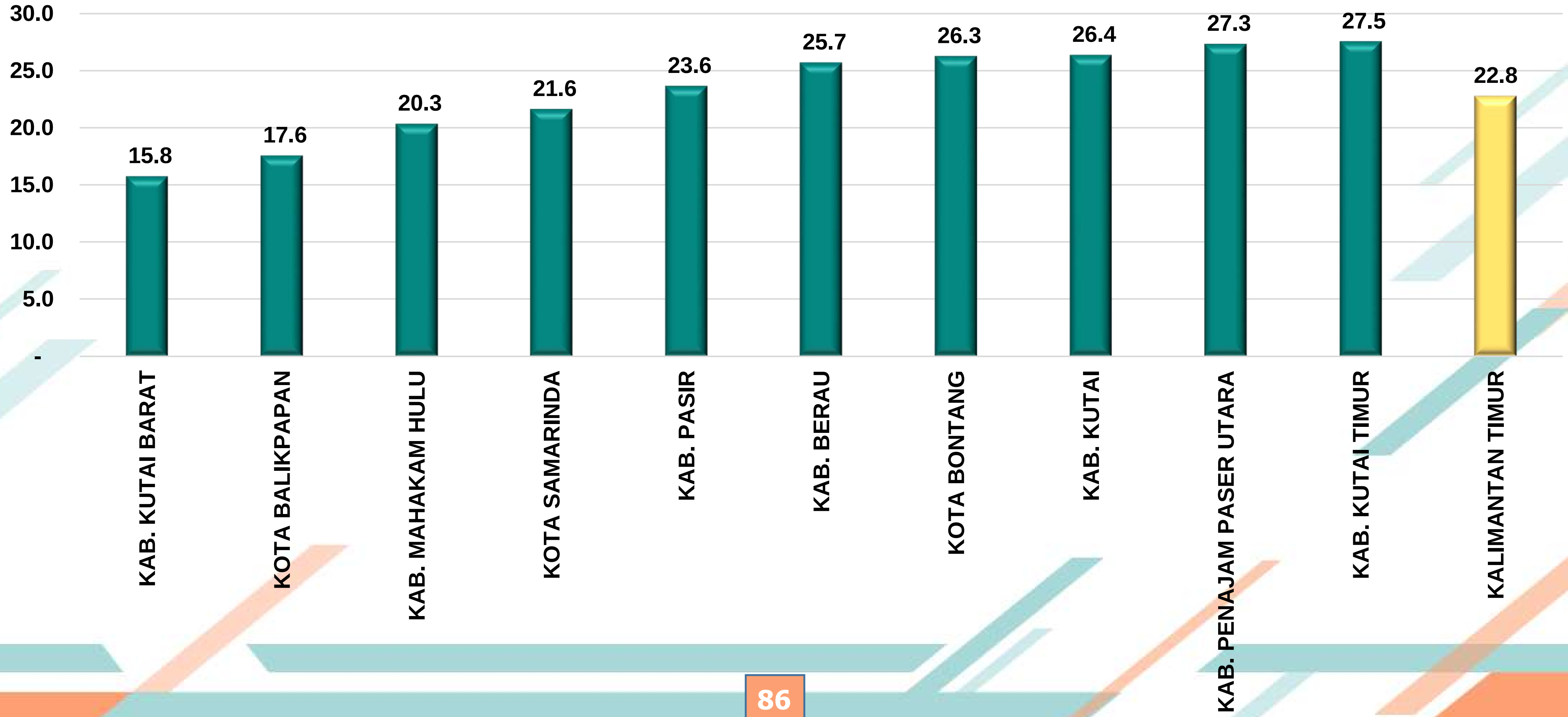

PREVALENSI BALITA STUNTED (TINGGI BADAN MENURUT UMUR) BERDASARKAN KABUPATEN/KOTA DI PROVINSI KALIMANTAN UTARA, SSGI 2021

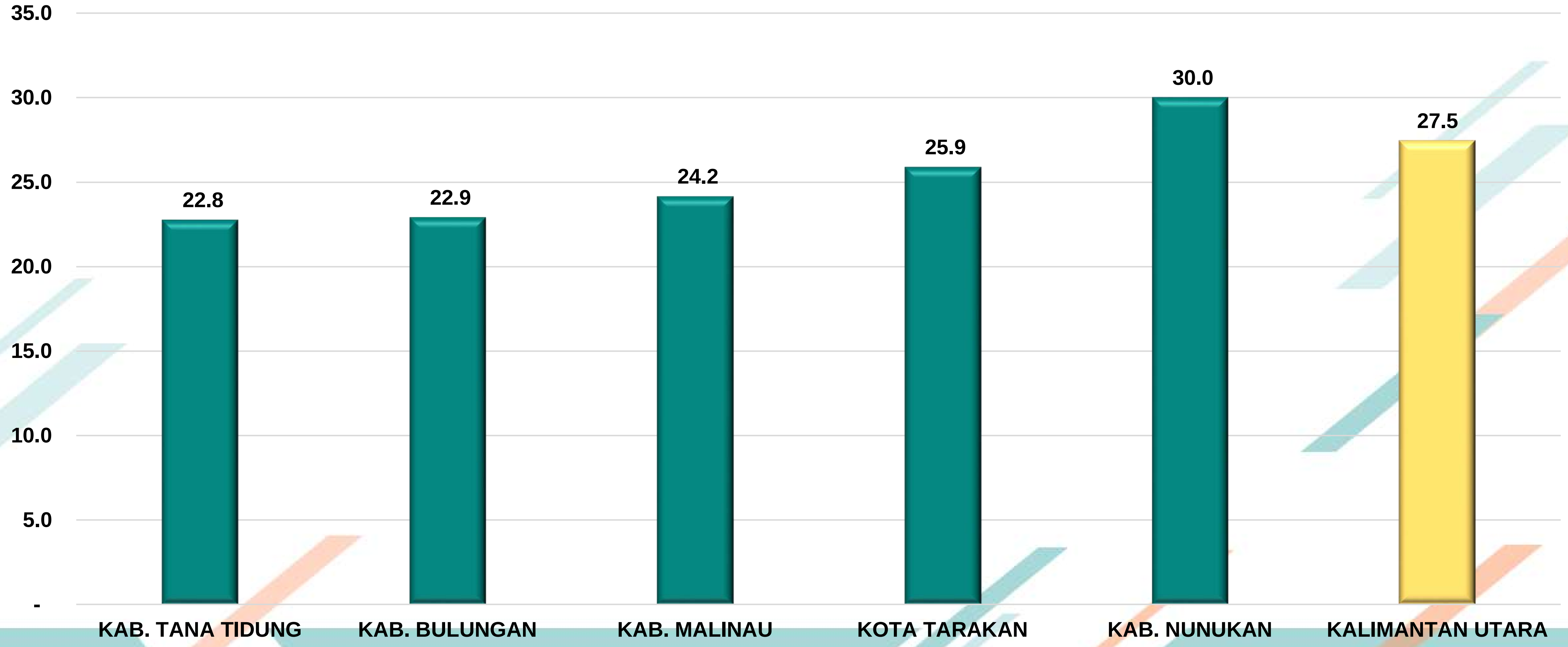

PREVALENSI BALITA *STUNTED* (TINGGI BADAN MENURUT UMUR) BERDASARKAN KABUPATEN/KOTA DI PROVINSI SULAWESI UTARA, SSGI 2021

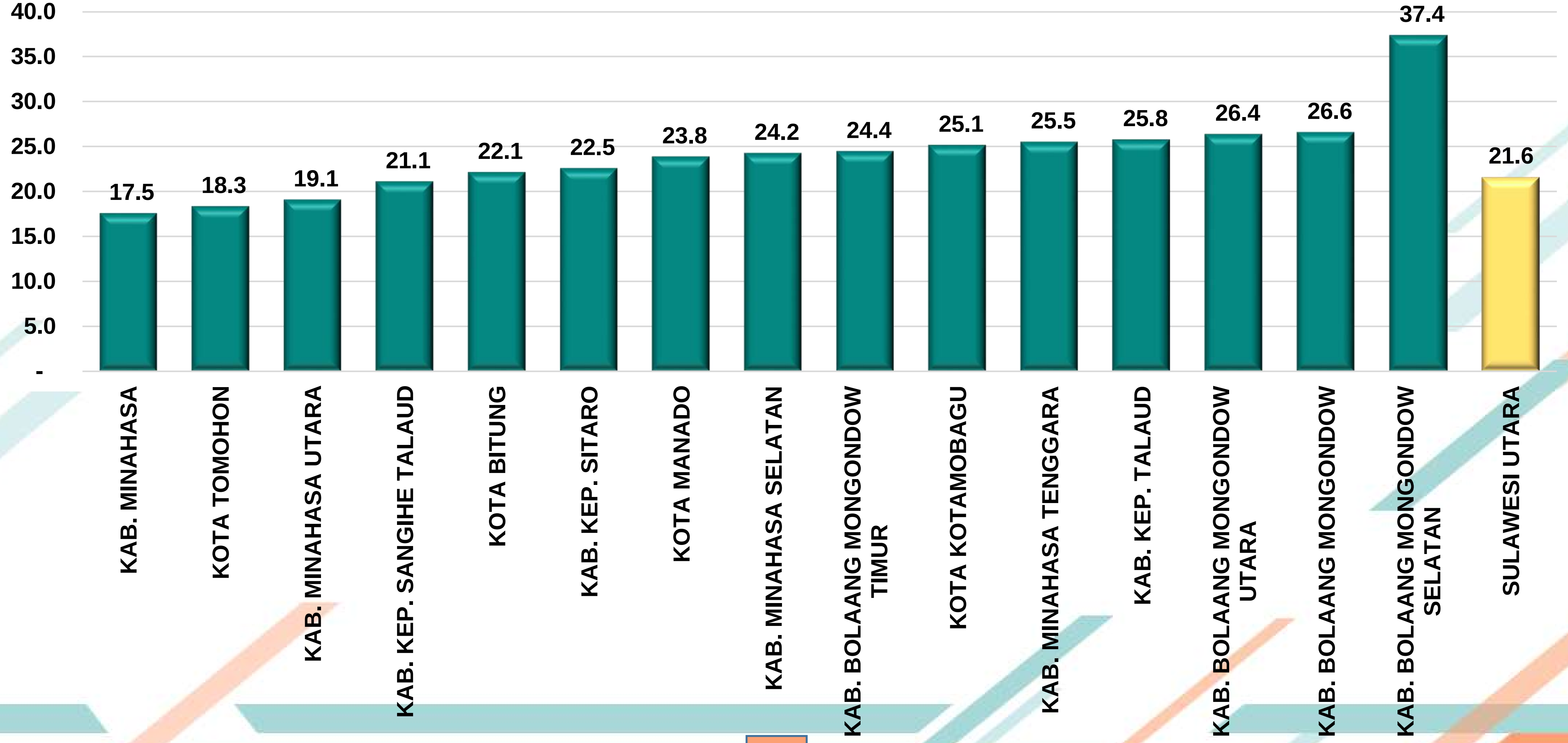

PREVALENSI BALITA *STUNTED* (TINGGI BADAN MENURUT UMUR) BERDASARKAN KABUPATEN/KOTA DI PROVINSI SULAWESI TENGAH, SSGI 2021

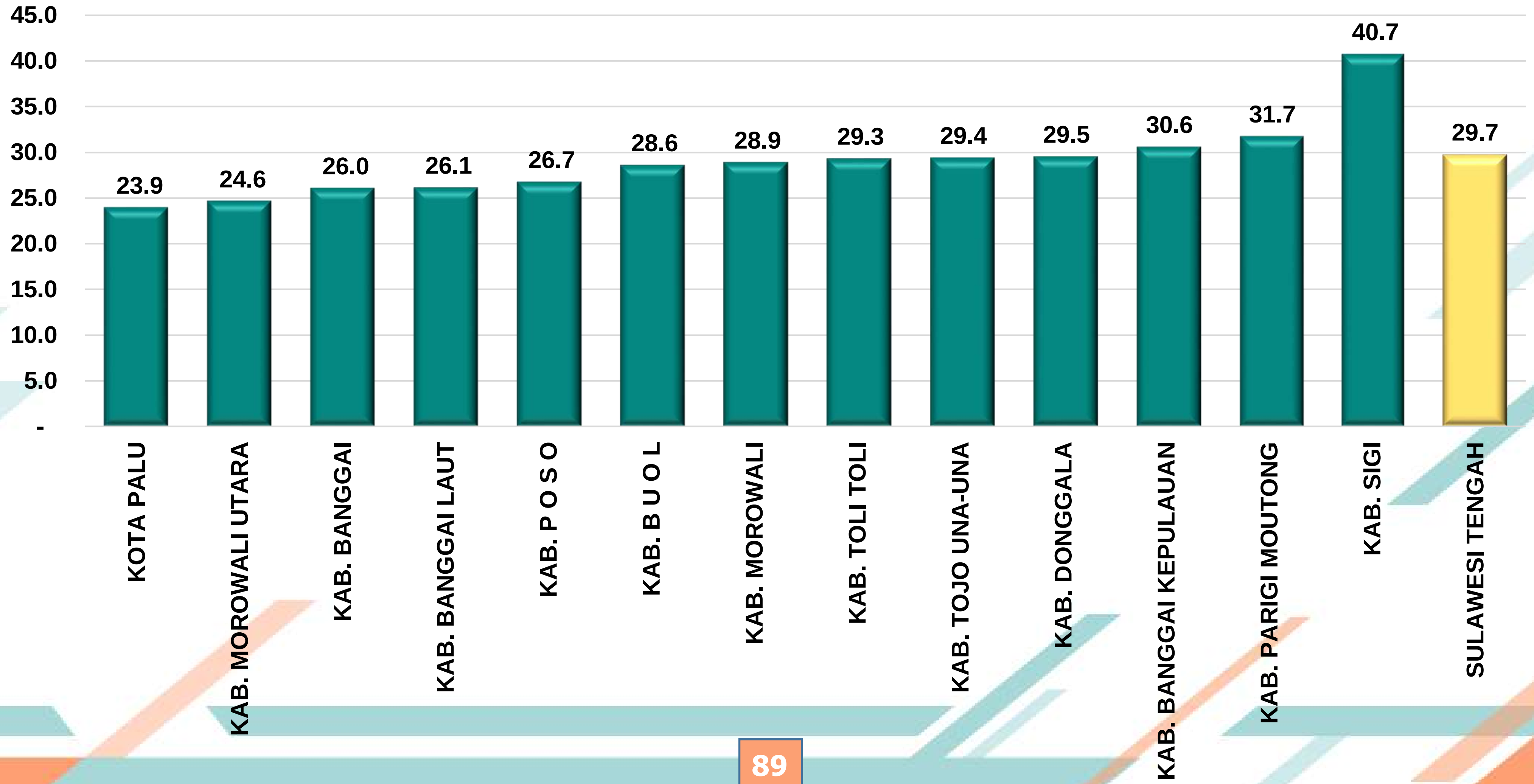

KEMENTERIAN KESEHATAN REPUBLIK INDONESIA

SSGI Studi Status Gizi Indonesia

PREVALENSI BALITA *STUNTED* (TINGGI BADAN MENURUT UMUR) BERDASARKAN KABUPATEN/KOTA DI PROVINSI SULAWESI SELATAN, SSGI 2021

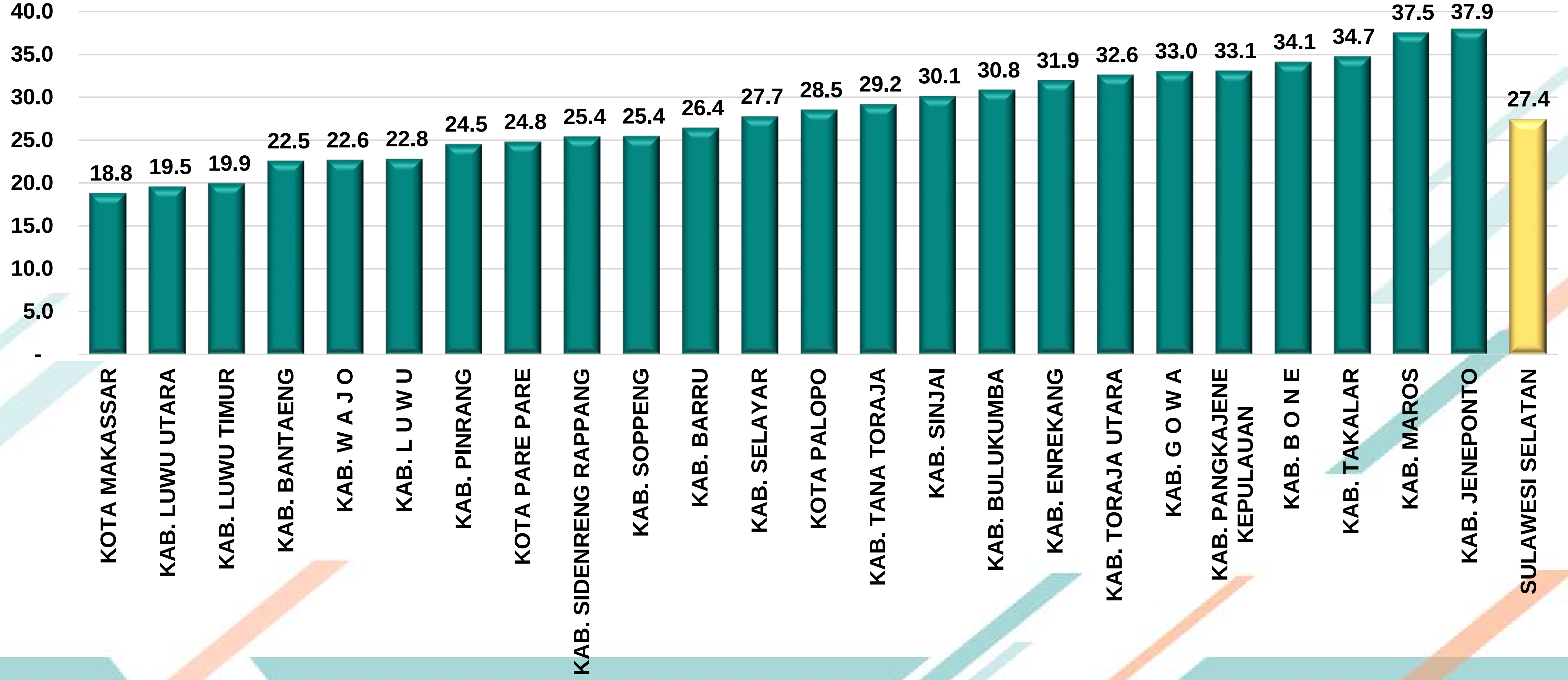

KEMENTERIAN KESEHATAN REPUBLIK INDONESIA

SSGI Studi Status Gizi Indonesia

PREVALENSI BALITA *STUNTED* (TINGGI BADAN MENURUT UMUR) BERDASARKAN KABUPATEN/KOTA DI PROVINSI SULAWESI TENGGARA, SSGI 2021

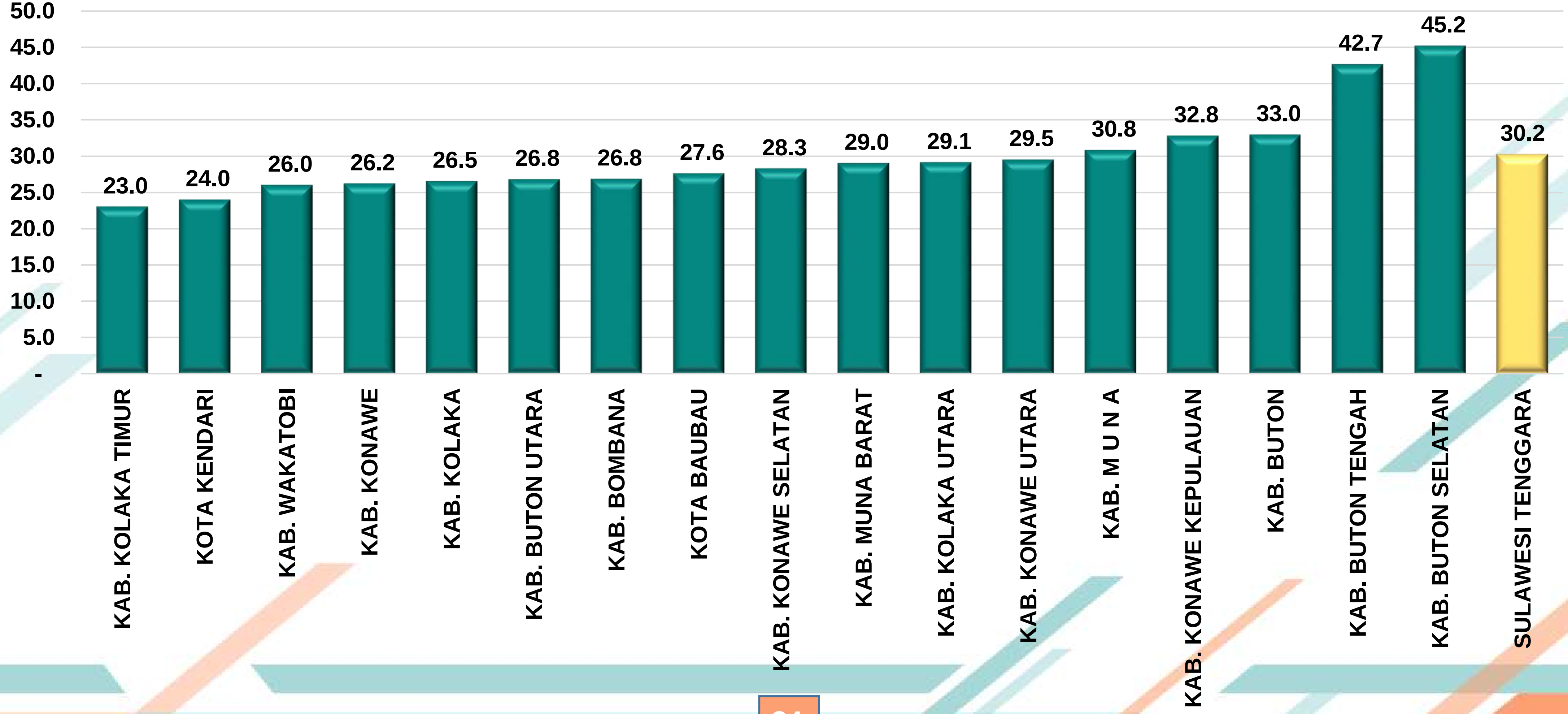

PREVALENSI BALITA *STUNTED* (TINGGI BADAN MENURUT UMUR) BERDASARKAN KABUPATEN/KOTA DI PROVINSI GORONTALO, SSGI 2021

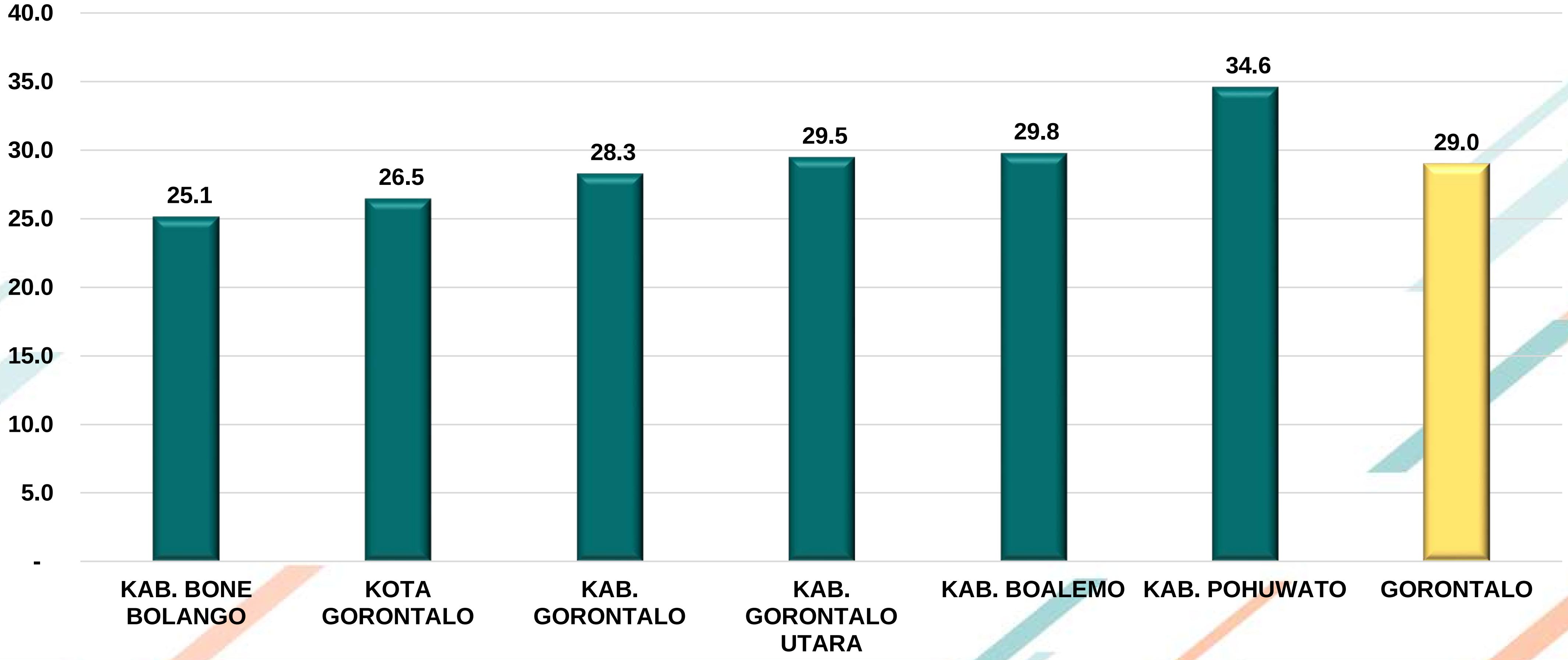

PREVALENSI BALITA *STUNTED* (TINGGI BADAN MENURUT UMUR) BERDASARKAN KABUPATEN/KOTA DI PROVINSI SULAWESI BARAT, SSGI 2021

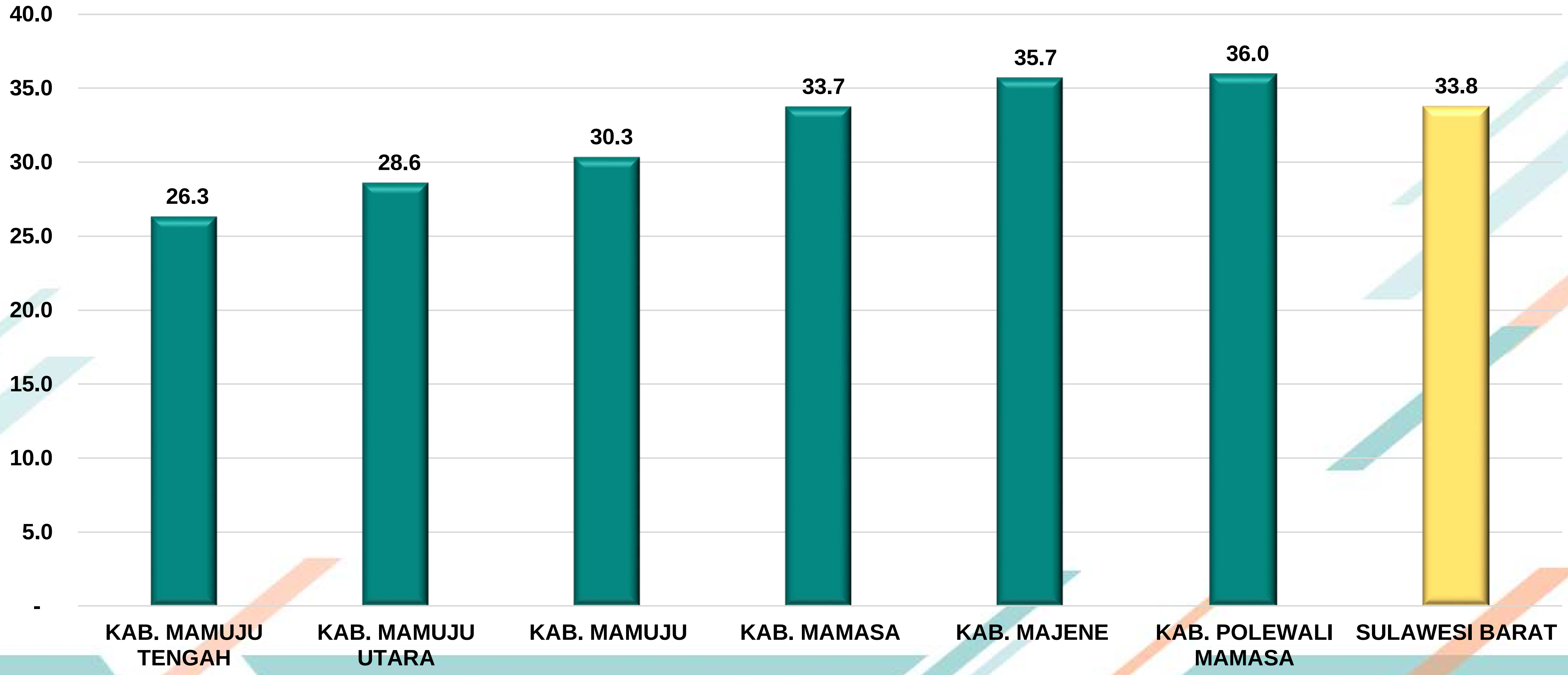

PREVALENSI BALITA *STUNTED* (TINGGI BADAN MENURUT UMUR) BERDASARKAN KABUPATEN/KOTA DI PROVINSI MALUKU, SSGI 2021

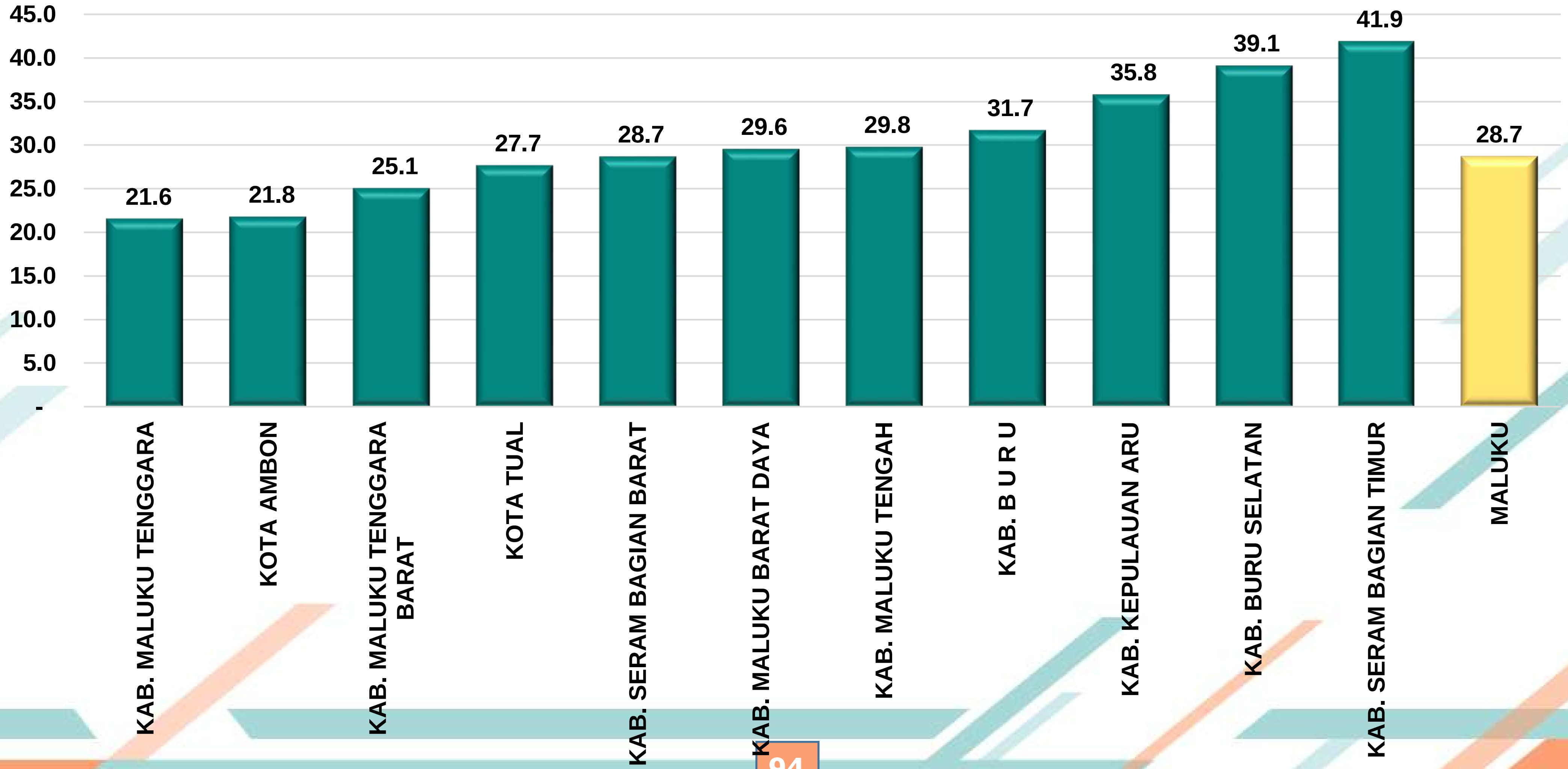

PREVALENSI BALITA *STUNTED* (TINGGI BADAN MENURUT UMUR) BERDASARKAN KABUPATEN/KOTA DI PROVINSI MALUKU UTARA, SSGI 2021

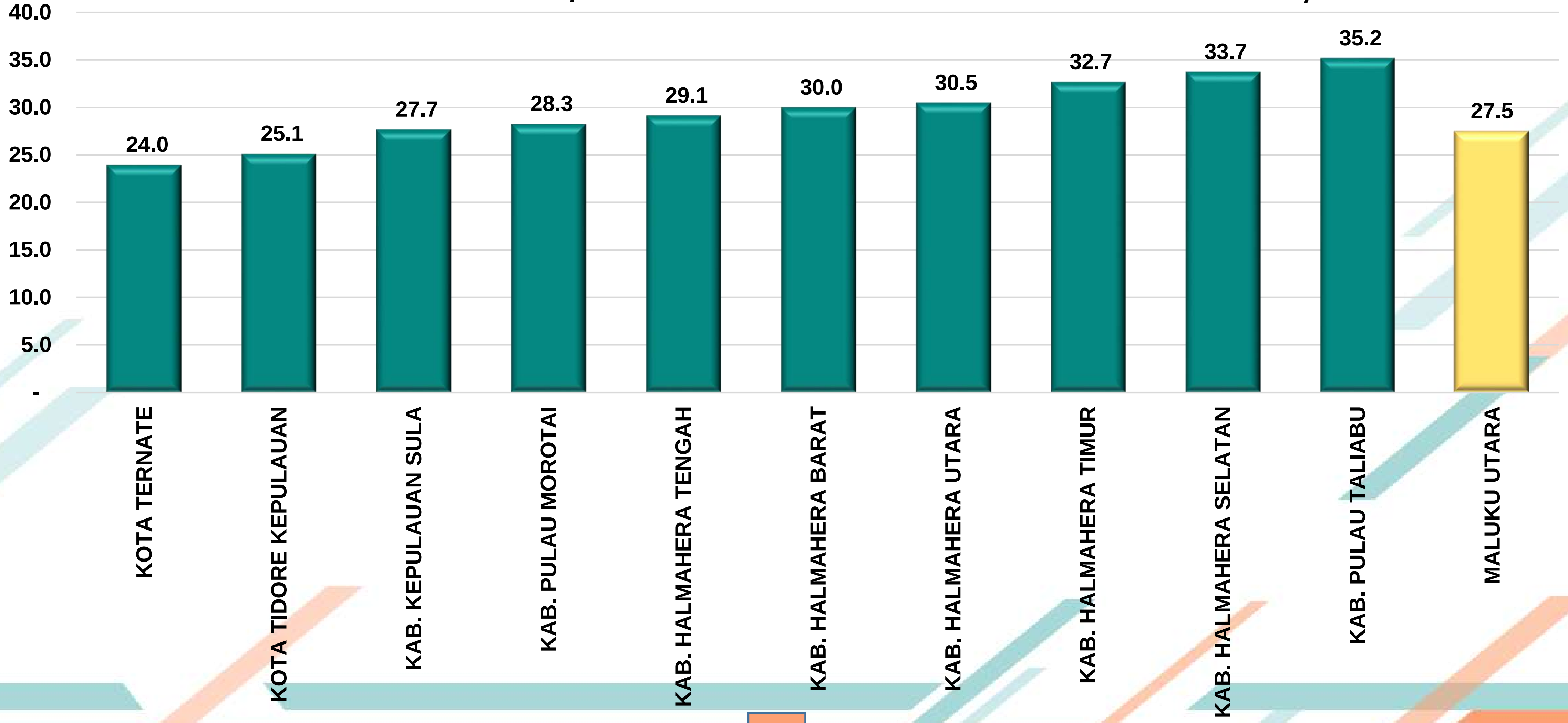

KEMENTERIAN KESEHATAN REPUBLIK INDONESIA

PREVALENSI BALITA *STUNTED* (TINGGI BADAN MENURUT UMUR) BERDASARKAN KABUPATEN/KOTA DI PROVINSI PAPUA BARAT, SSGI 2021

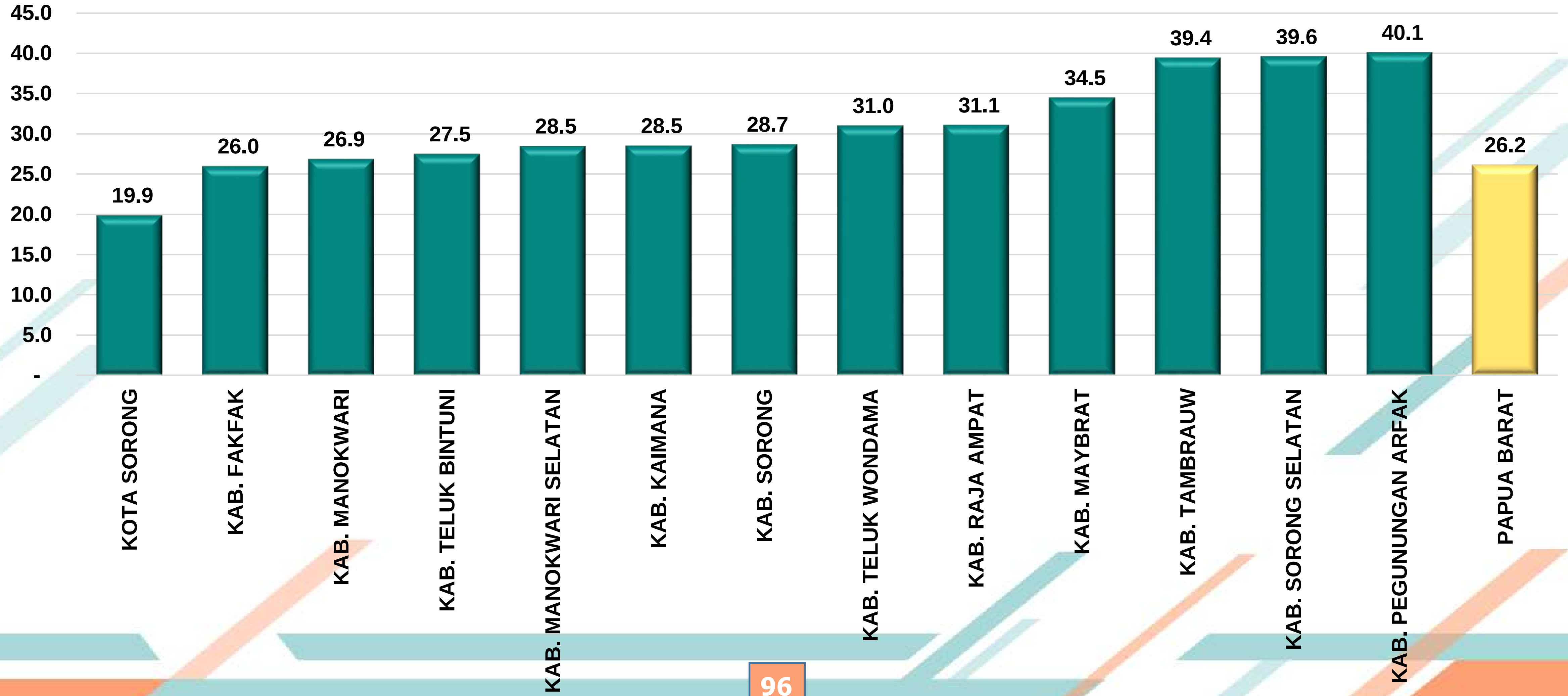

PREVALENSI BALITA *STUNTED* (TINGGI BADAN MENURUT UMUR) BERDASARKAN KABUPATEN/KOTA DI PROVINSI PAPUA, SSGI 2021

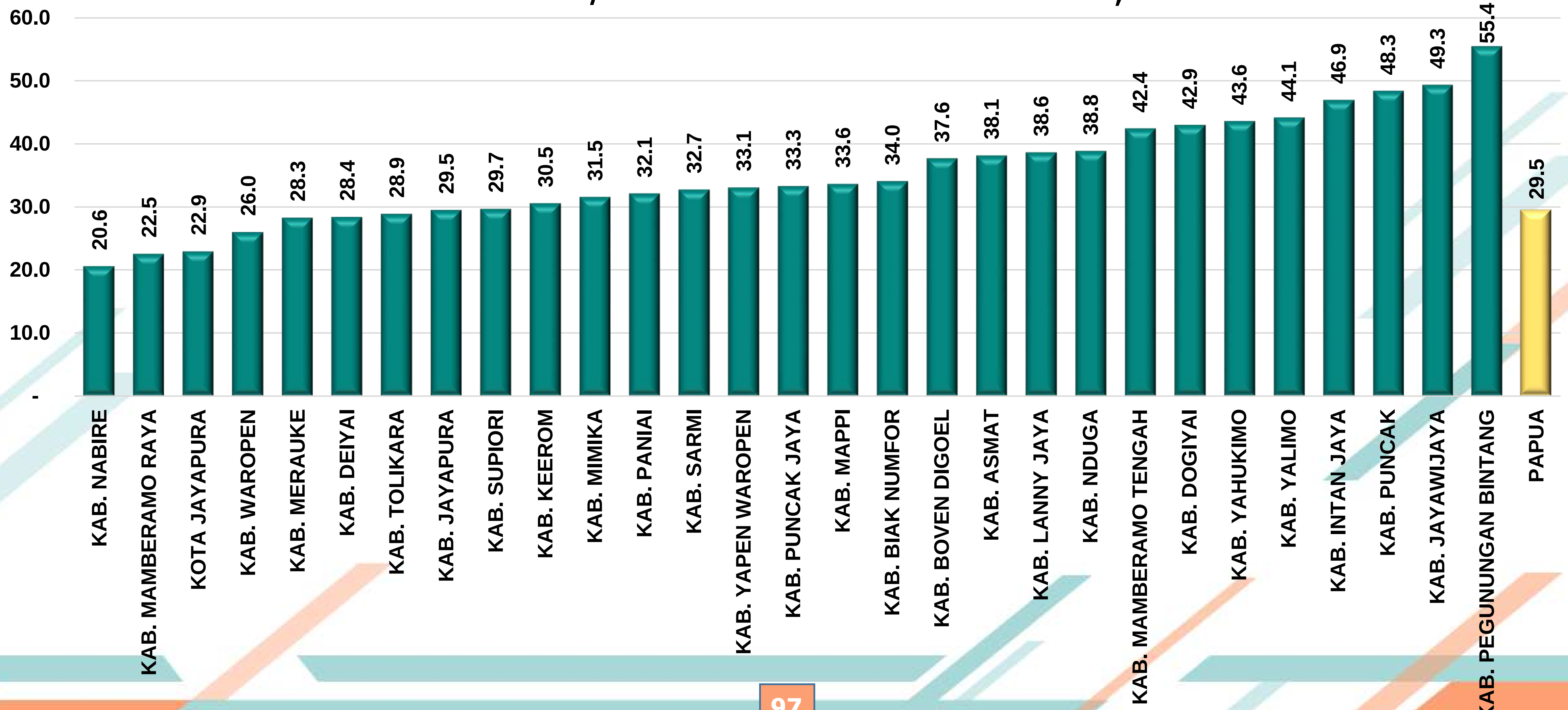

KEMENTERIAN KESEHATAN REPUBLIK INDONESIA

SSGI
Studi Status Gizi Indonesia

ANGKA *WASTED*
TINGKAT KABUPATEN DAN KOTA

KEMENTERIAN KESEHATAN REPUBLIK INDONESIA

SSGI

PREVALENSI BALITA *WASTED* (BERAT BADAN MENURUT TINGGI BADAN) BERDASARKAN KABUPATEN/KOTA DI PROVINSI ACEH, SSGI 2021

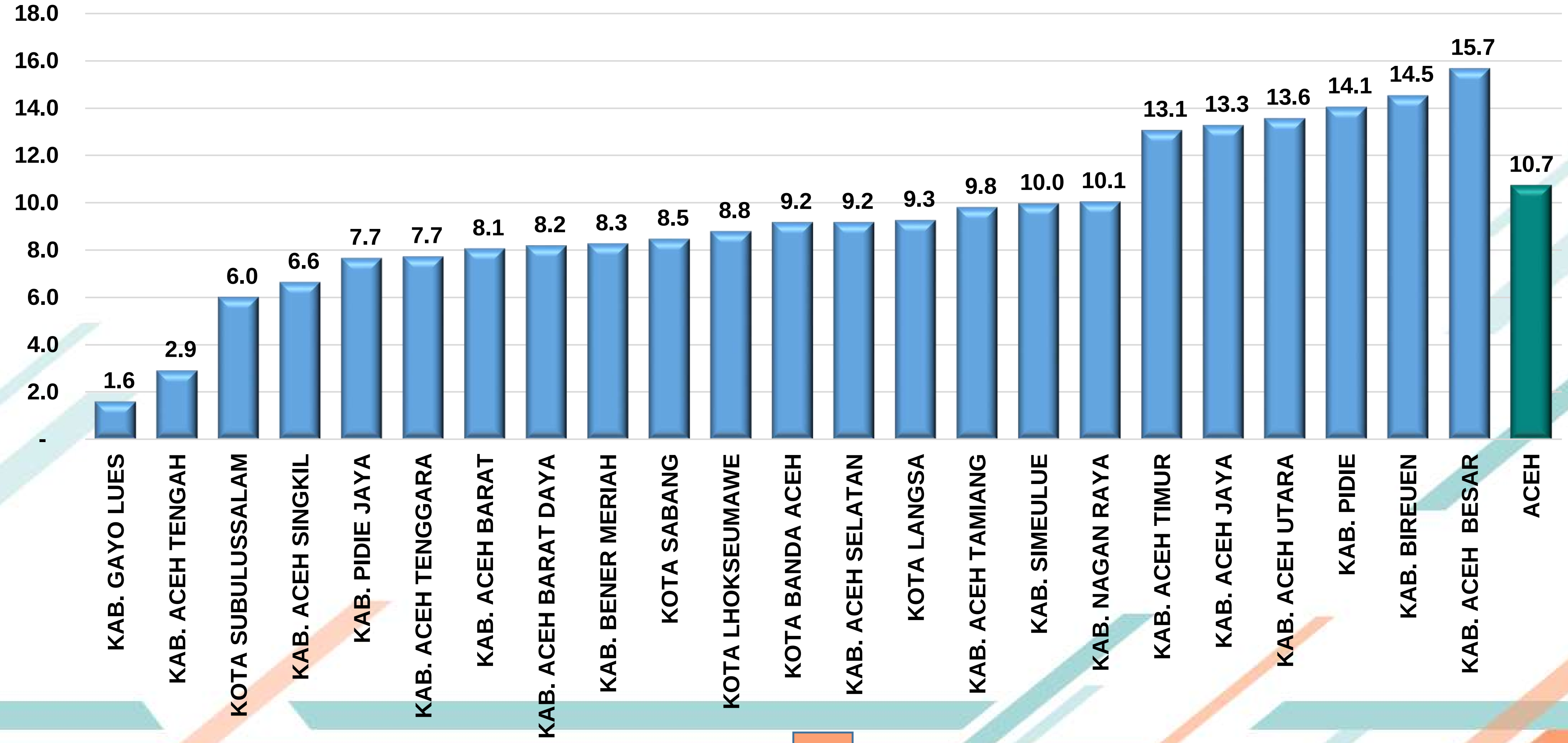

PREVALENSI BALITA *WASTED* (BERAT BADAN MENURUT TINGGI BADAN) BERDASARKAN KABUPATEN/KOTA DI PROVINSI SUMATERA UTARA, SSGI 2021

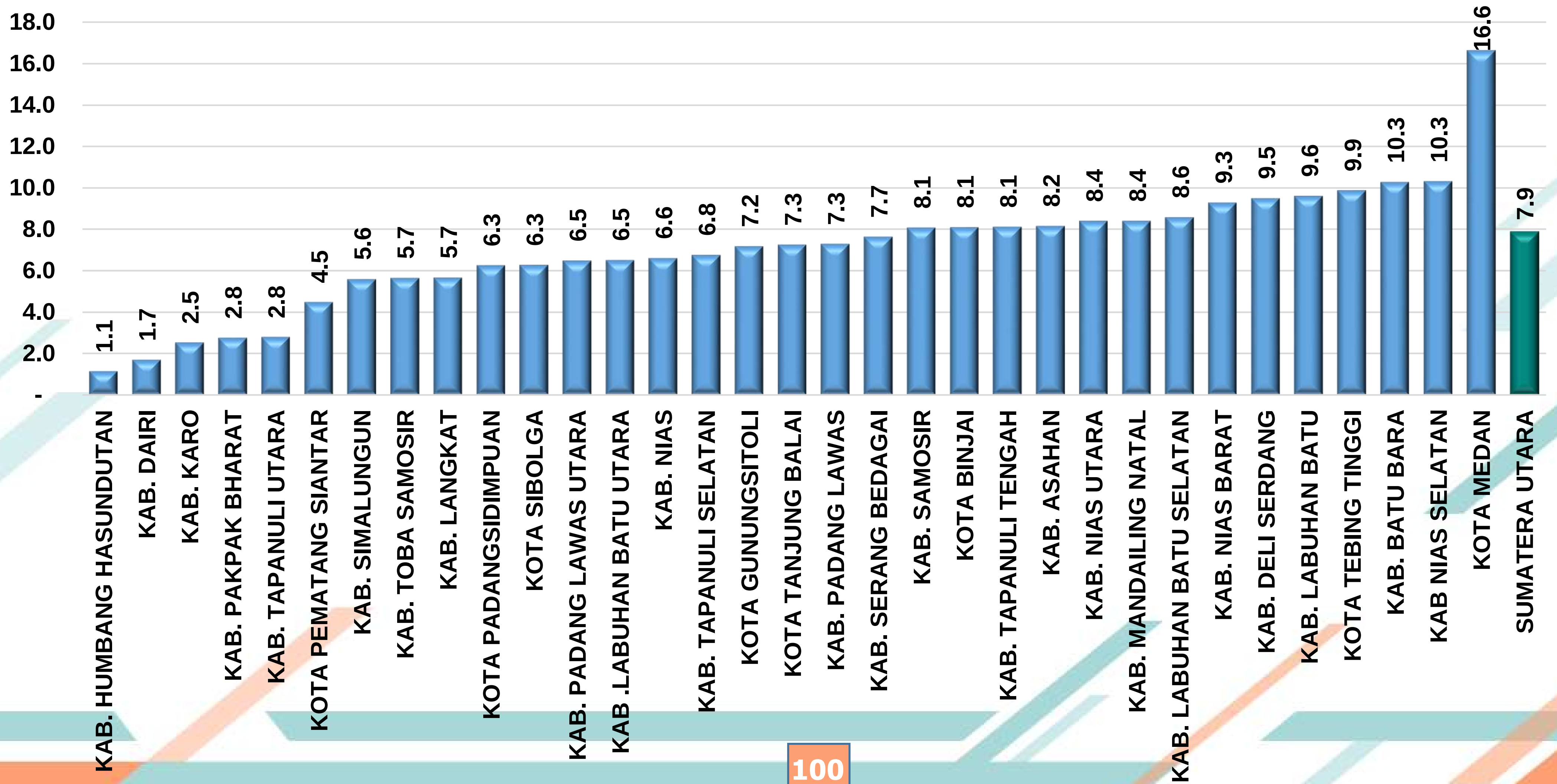

PREVALENSI BALITA *WASTED* (BERAT BADAN MENURUT TINGGI BADAN) BERDASARKAN KABUPATEN/KOTA DI PROVINSI SUMATERA BARAT, SSGI 2021

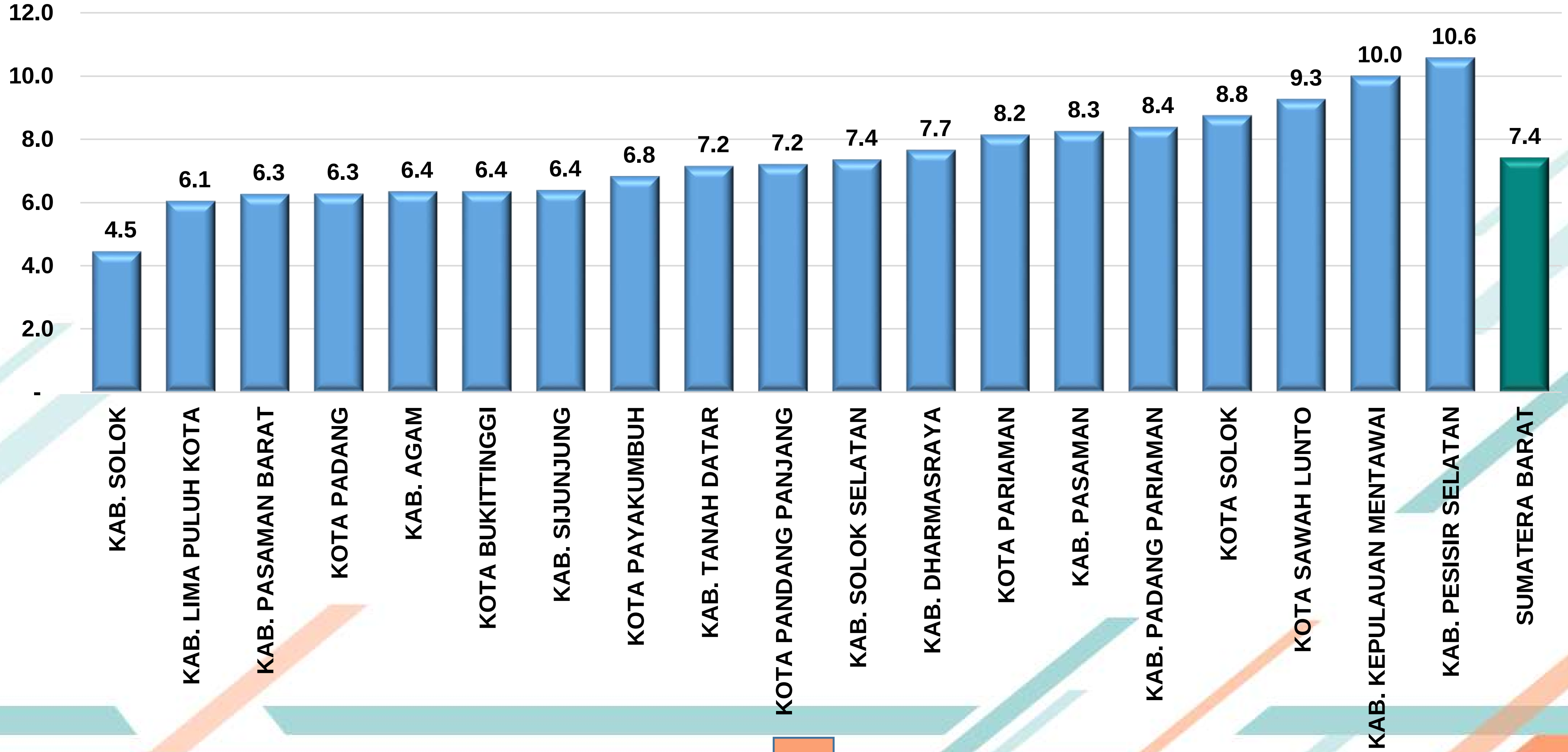

PREVALENSI BALITA *WASTED* (BERAT BADAN MENURUT TINGGI BADAN) BERDASARKAN KABUPATEN/KOTA DI PROVINSI RIAU, SSGI 2021

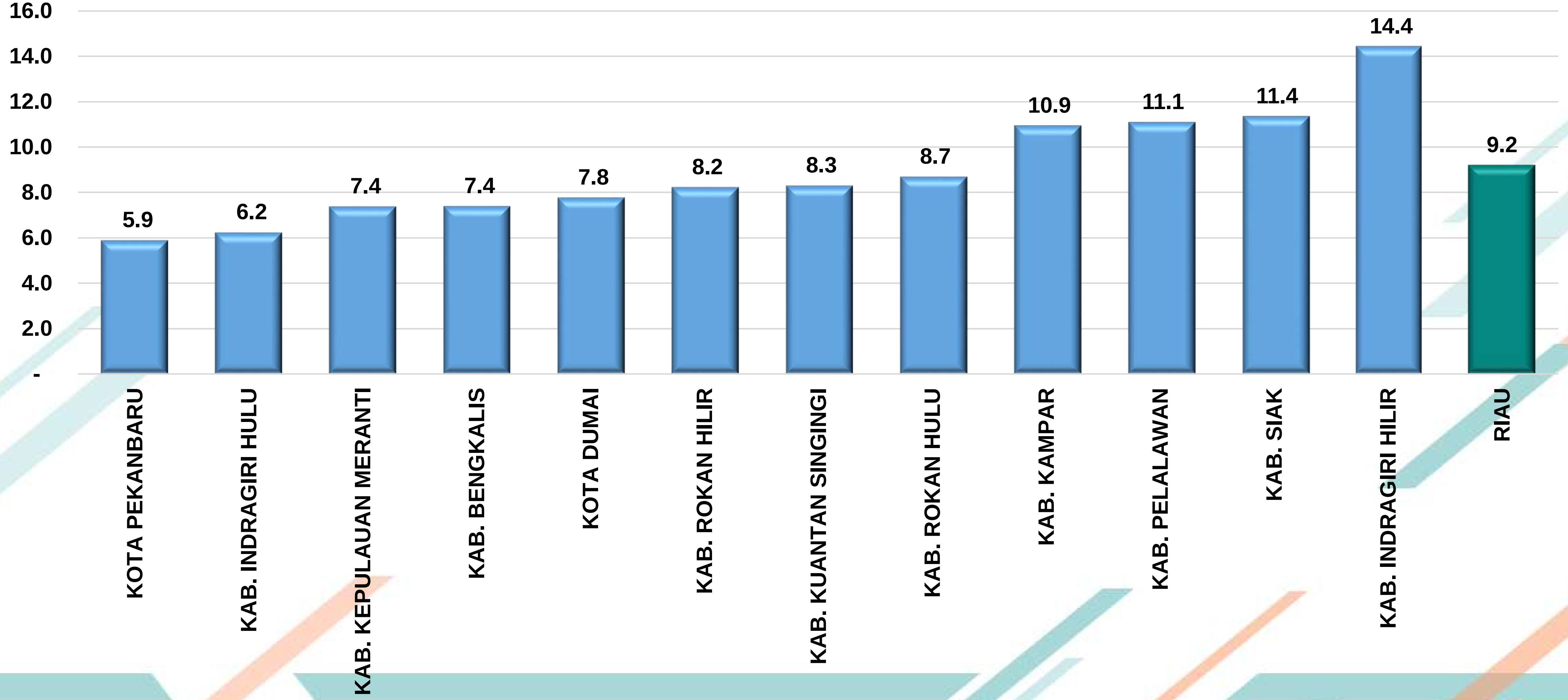

PREVALENSI BALITA *WASTED* (BERAT BADAN MENURUT TINGGI BADAN) BERDASARKAN KABUPATEN/KOTA DI PROVINSI JAMBI, SSGI 2021

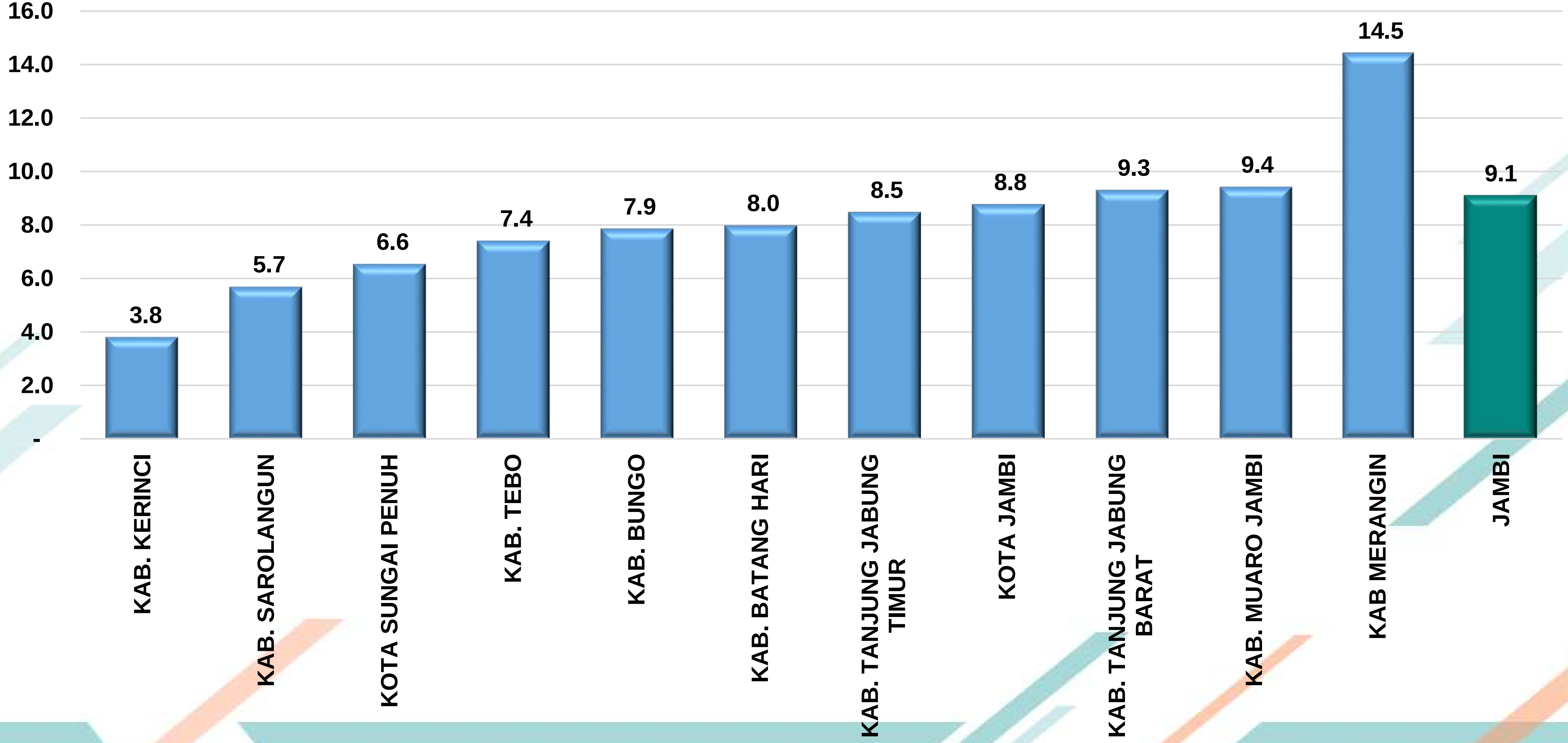

KEMENTERIAN KESEHATAN REPUBLIK INDONESIA

SSGI

PREVALENSI BALITA *WASTED* (BERAT BADAN MENURUT TINGGI BADAN) BERDASARKAN KABUPATEN/KOTA DI PROVINSI SUMATERA SELATAN, SSGI 2021

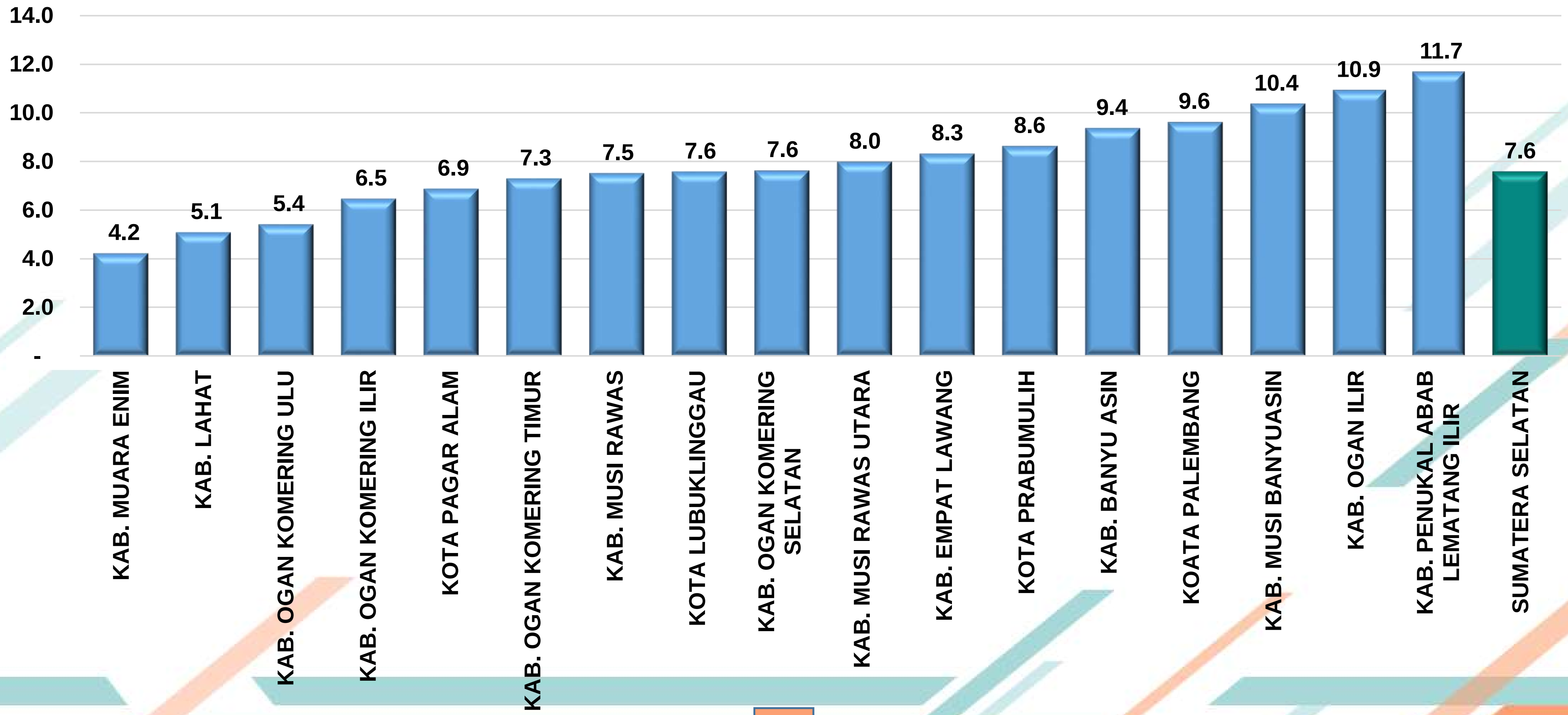

PREVALENSI BALITA *WASTED* (BERAT BADAN MENURUT TINGGI BADAN) BERDASARKAN KABUPATEN/KOTA DI PROVINSI BENGKULU, SSGI 2021

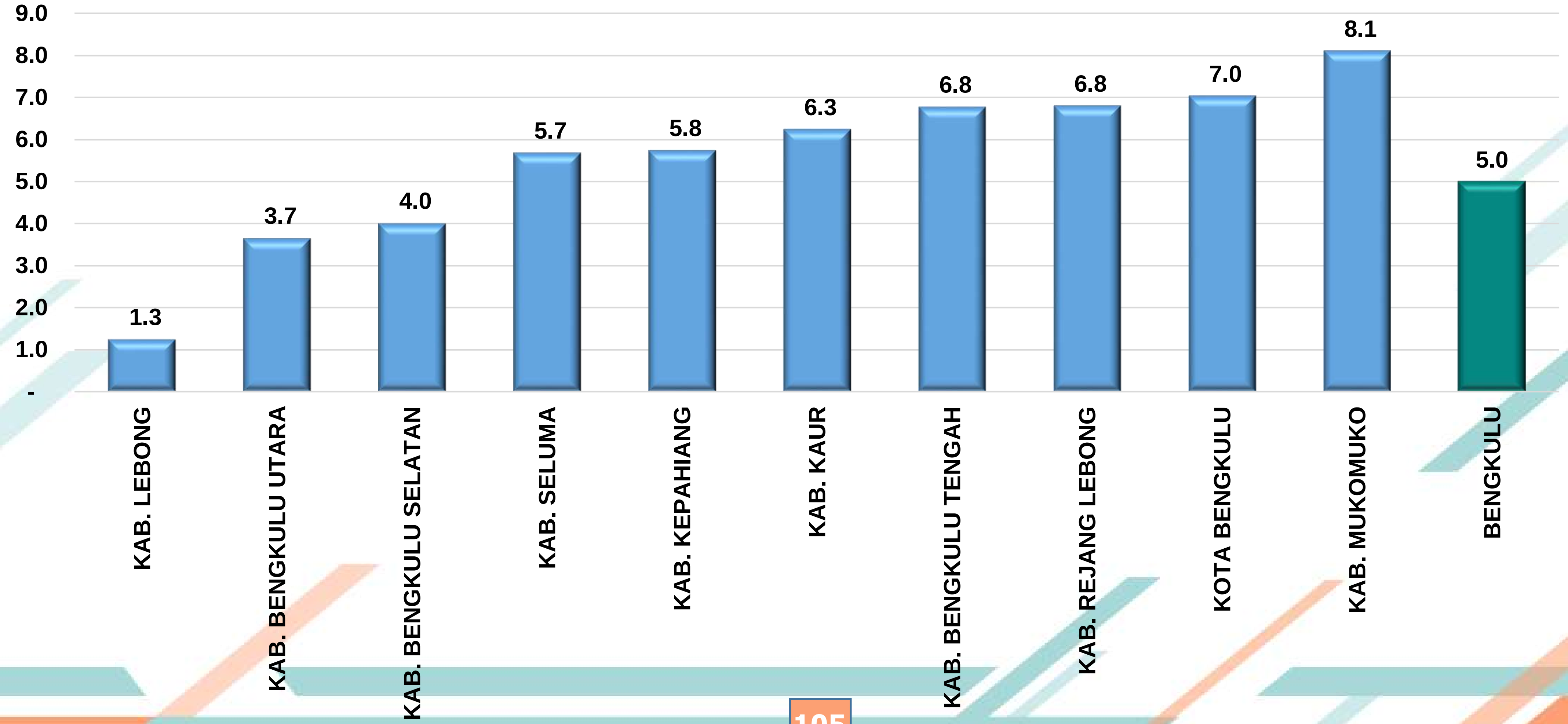

KEMENTERIAN KESEHATAN REPUBLIK INDONESIA

SSGI

PREVALENSI BALITA *WASTED* (BERAT BADAN MENURUT TINGGI BADAN) BERDASARKAN KABUPATEN/KOTA DI PROVINSI LAMPUNG, SSGI 2021

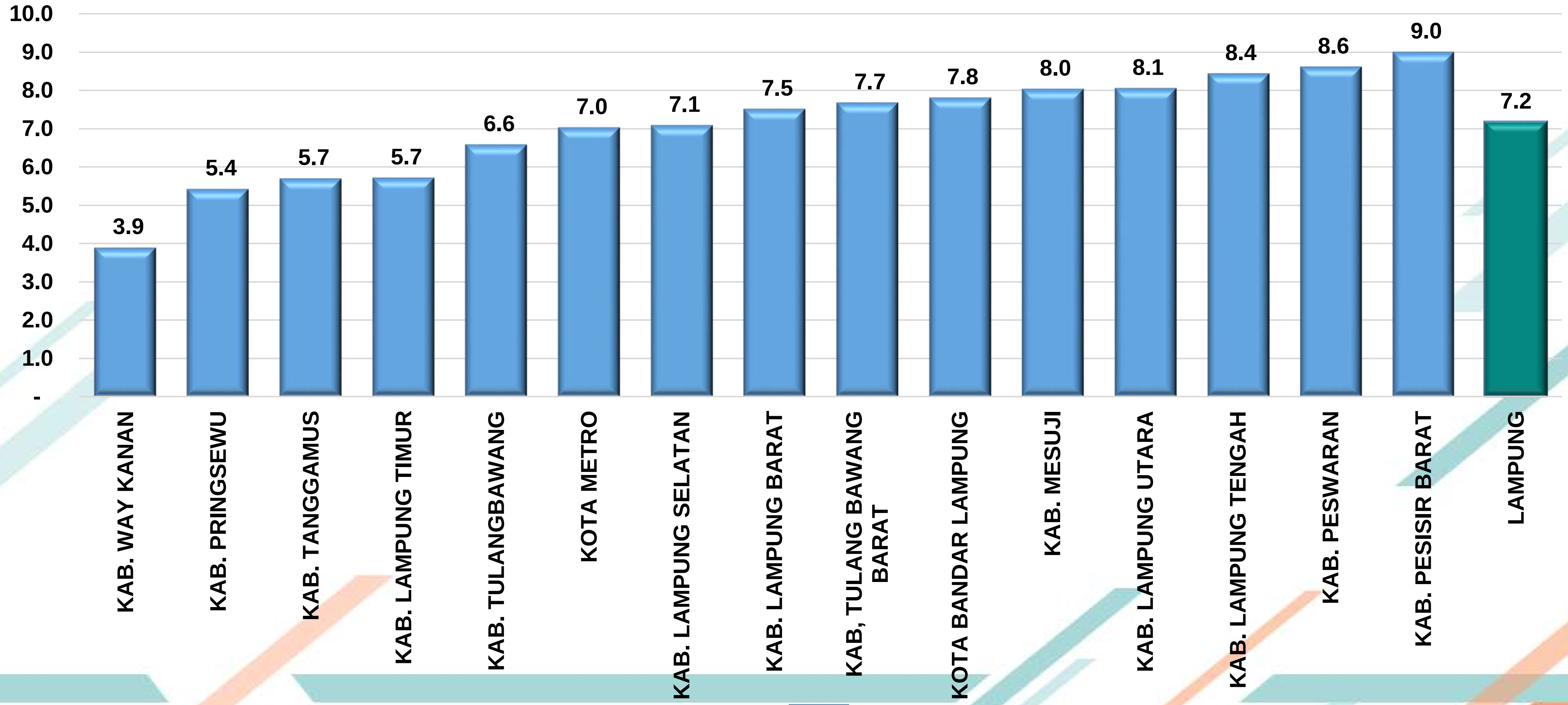

PREVALENSI BALITA *WASTED* (BERAT BADAN MENURUT TINGGI BADAN) BERDASARKAN KABUPATEN/KOTA DI PROVINSI KEP.BABEL, SSGI 2021

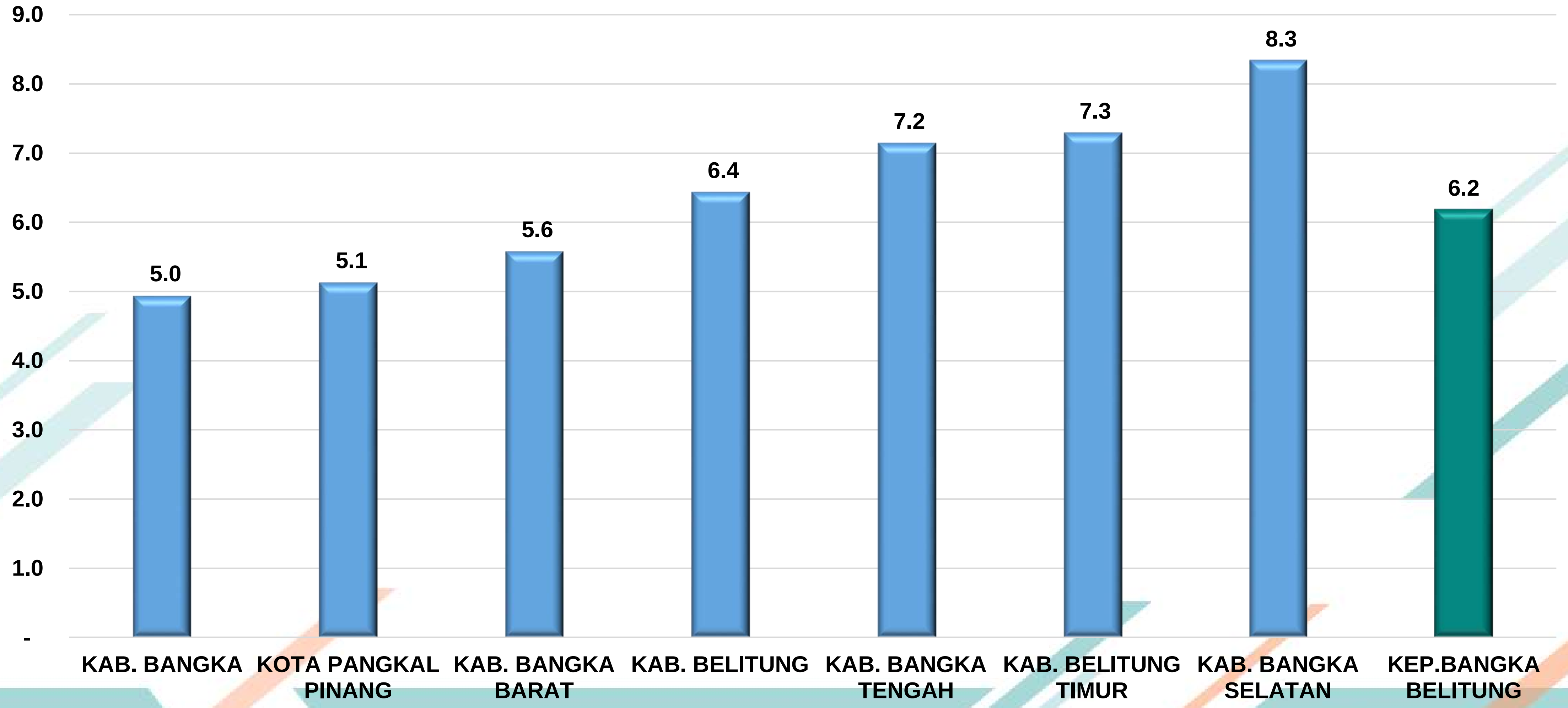

KEMENTERIAN KESEHATAN REPUBLIK INDONESIA

SSGI

PREVALENSI BALITA *WASTED* (BERAT BADAN MENURUT TINGGI BADAN) BERDASARKAN KABUPATEN/KOTA DI PROVINSI KEP.RIAU, SSGI 2021

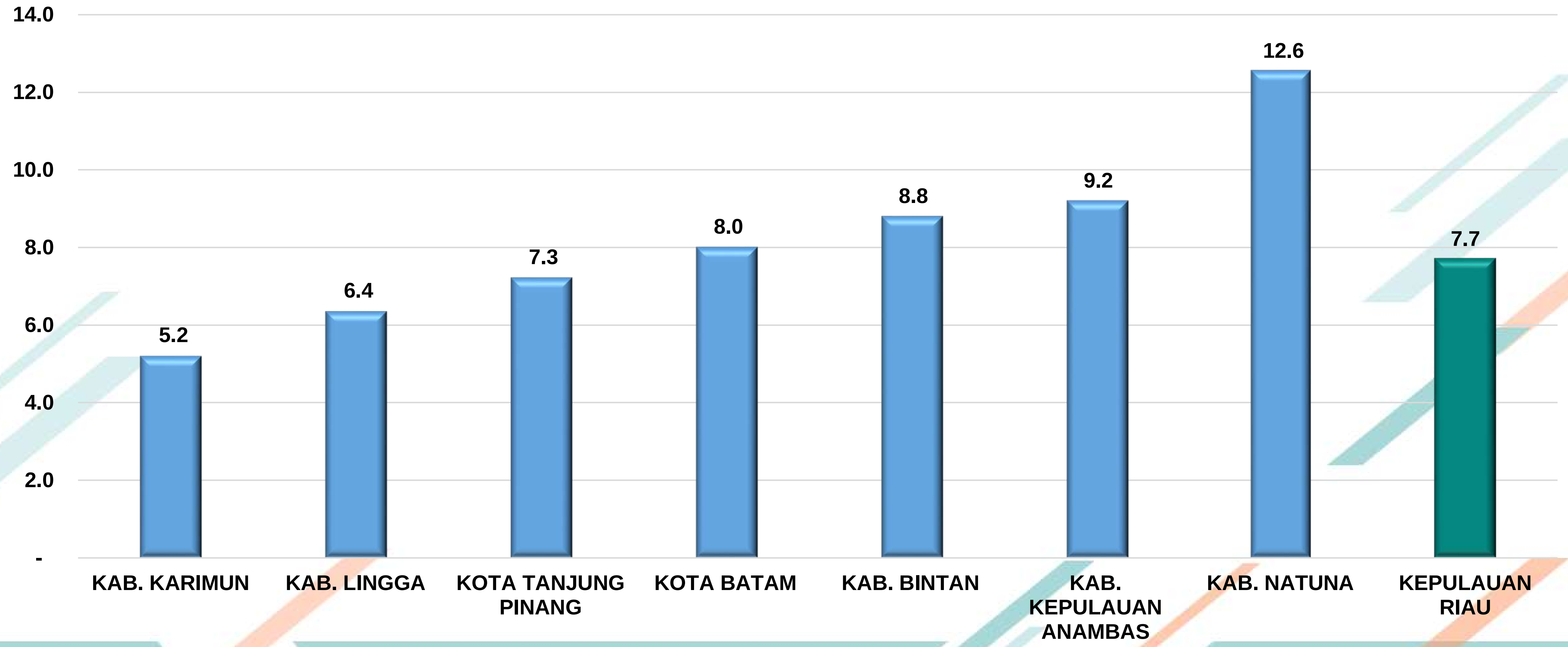

PREVALENSI BALITA *WASTED* (BERAT BADAN MENURUT TINGGI BADAN) BERDASARKAN KABUPATEN/KOTA DI PROVINSI DKI JAKARTA, SSGI 2021

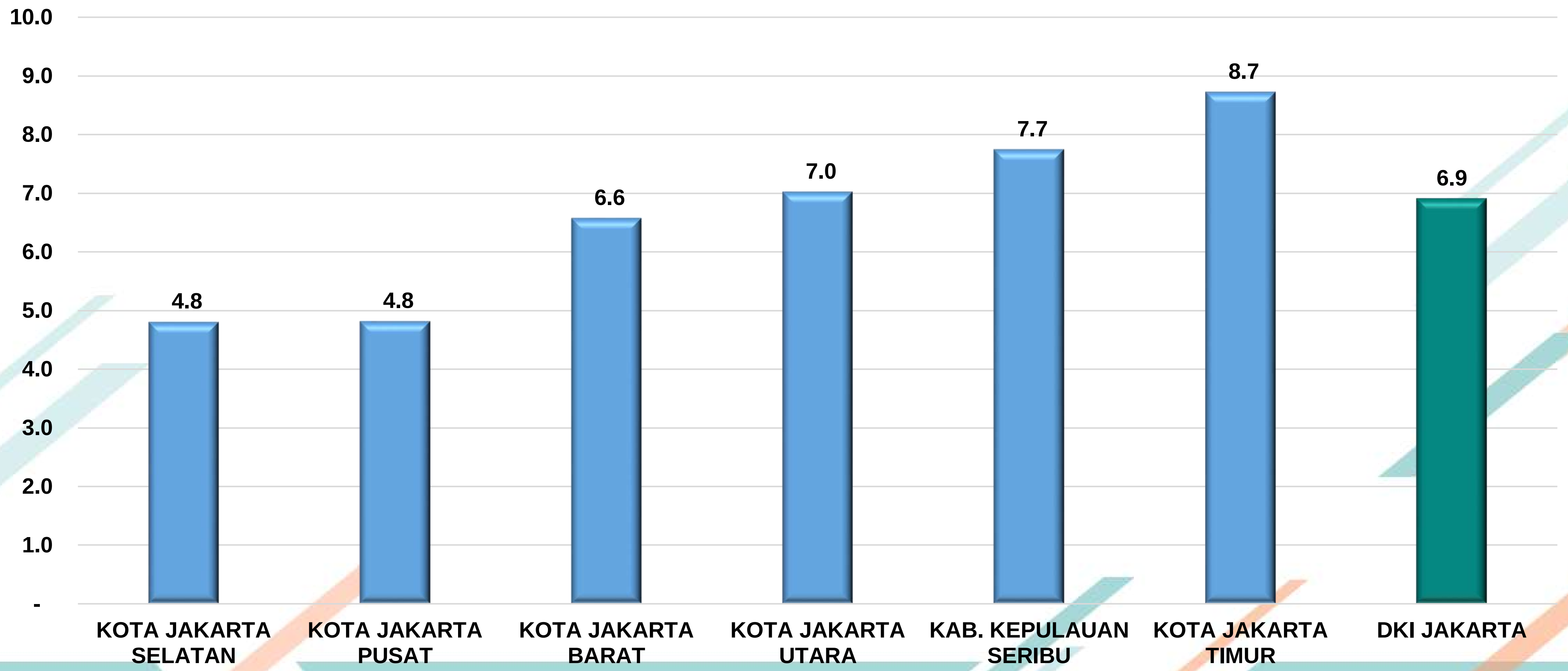

KEMENTERIAN KESEHATAN REPUBLIK INDONESIA

SSGI

PREVALENSI BALITA *WASTED* (BERAT BADAN MENURUT TINGGI BADAN) BERDASARKAN KABUPATEN/KOTA DI PROVINSI JAWA BARAT, SSGI 2021

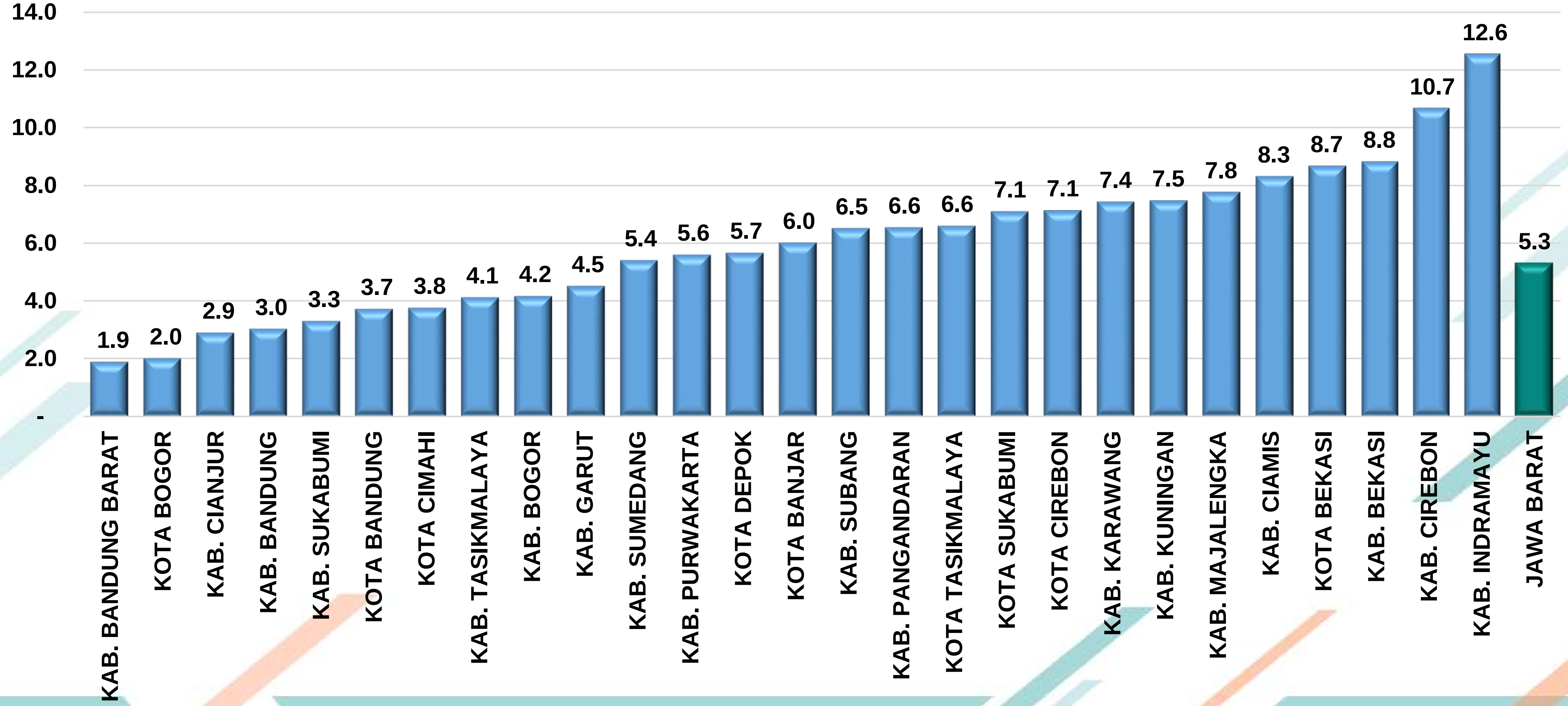

KEMENTERIAN KESEHATAN REPUBLIK INDONESIA

SSGI

PREVALENSI BALITA *WASTED* (BERAT BADAN MENURUT TINGGI BADAN) BERDASARKAN KABUPATEN/KOTA DI PROVINSI JAWA TENGAH, SSGI 2021

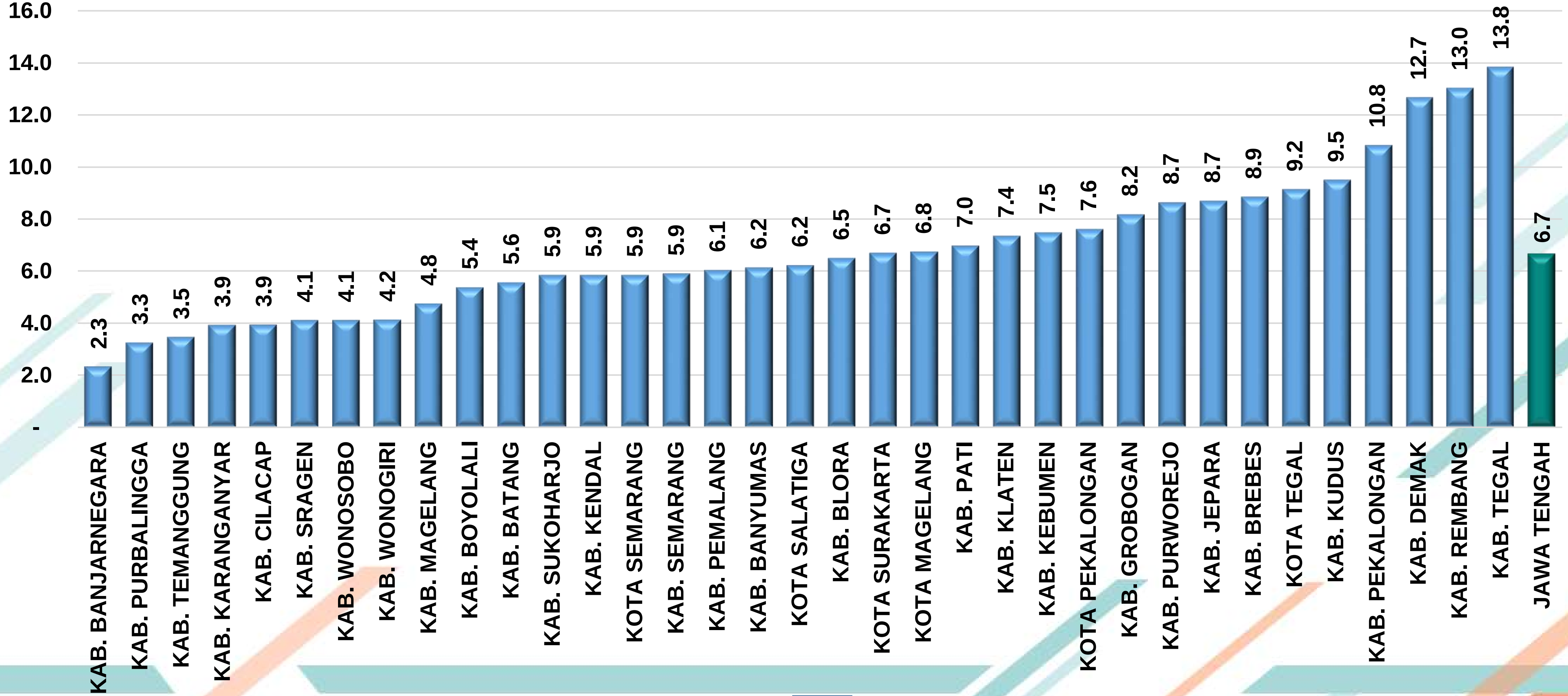

PREVALENSI BALITA *WASTED* (BERAT BADAN MENURUT TINGGI BADAN) BERDASARKAN KABUPATEN/KOTA DI PROVINSI DIY, SSGI 2021

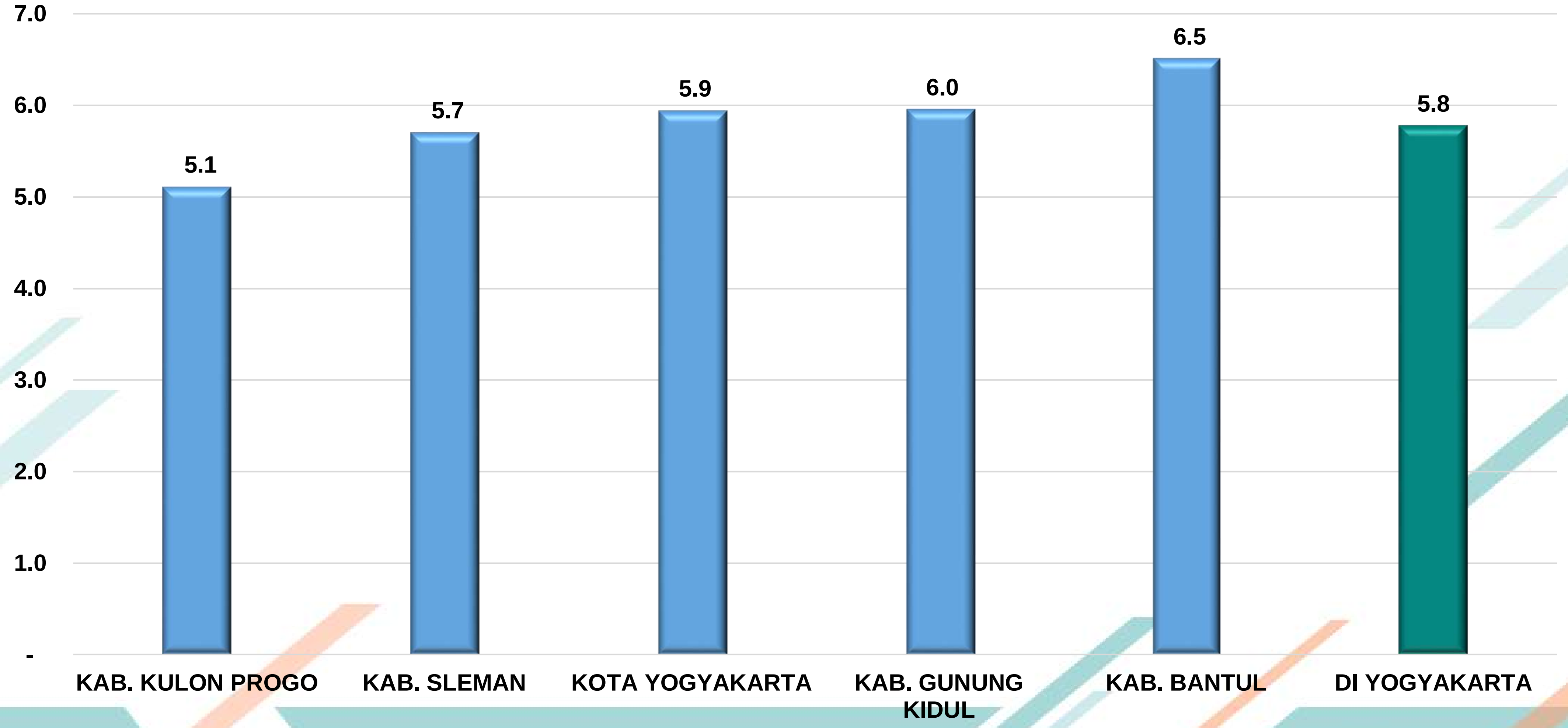

KEMENTERIAN KESEHATAN REPUBLIK INDONESIA

SSGI

PREVALENSI BALITA *WASTED* (BERAT BADAN MENURUT TINGGI BADAN) BERDASARKAN KABUPATEN/KOTA DI PROVINSI JAWA TIMUR, SSGI 2021

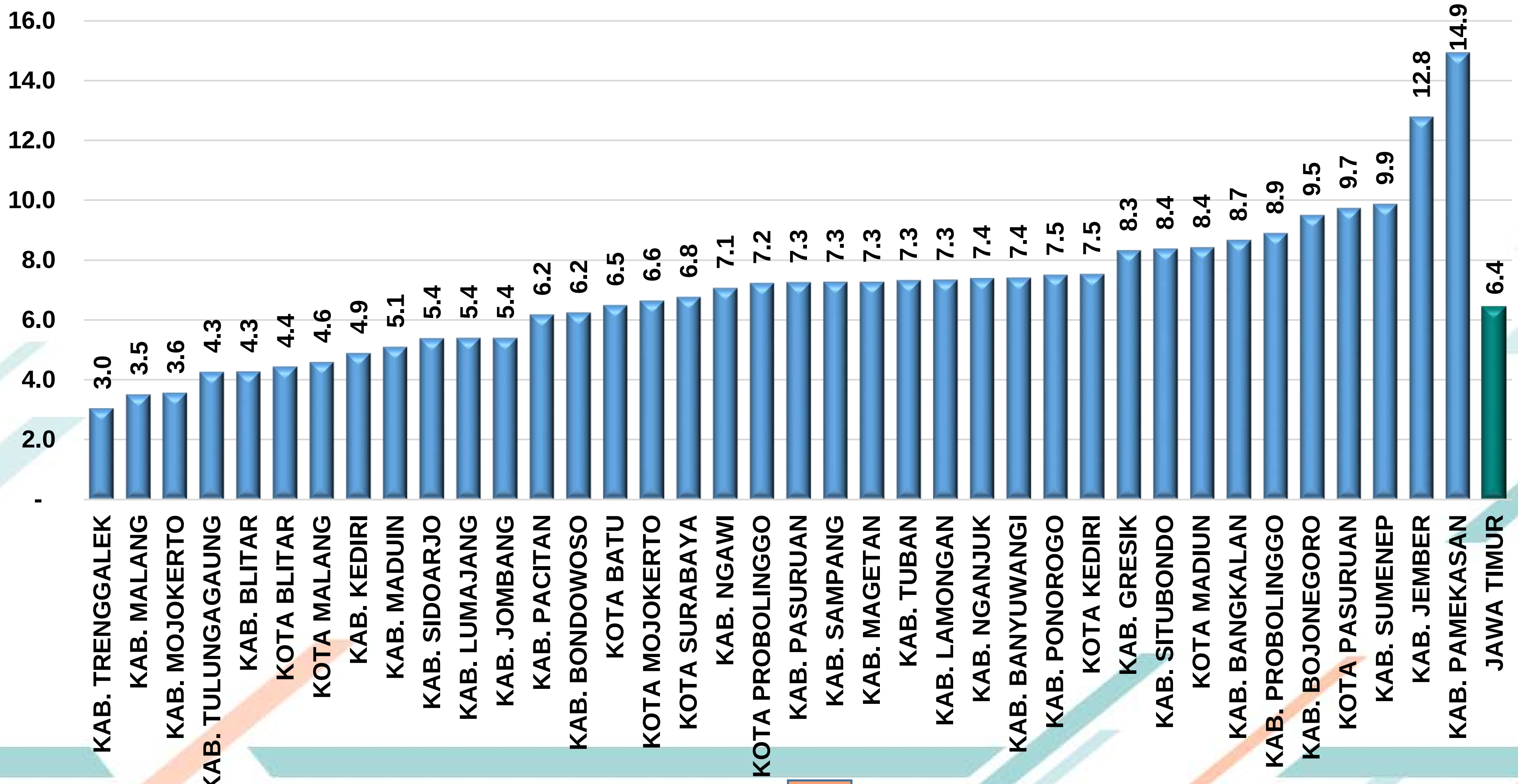

KEMENTERIAN KESEHATAN REPUBLIK INDONESIA

SSGI

PREVALENSI BALITA *WASTED* (BERAT BADAN MENURUT TINGGI BADAN) BERDASARKAN KABUPATEN/KOTA DI PROVINSI BANTEN, SSGI 2021

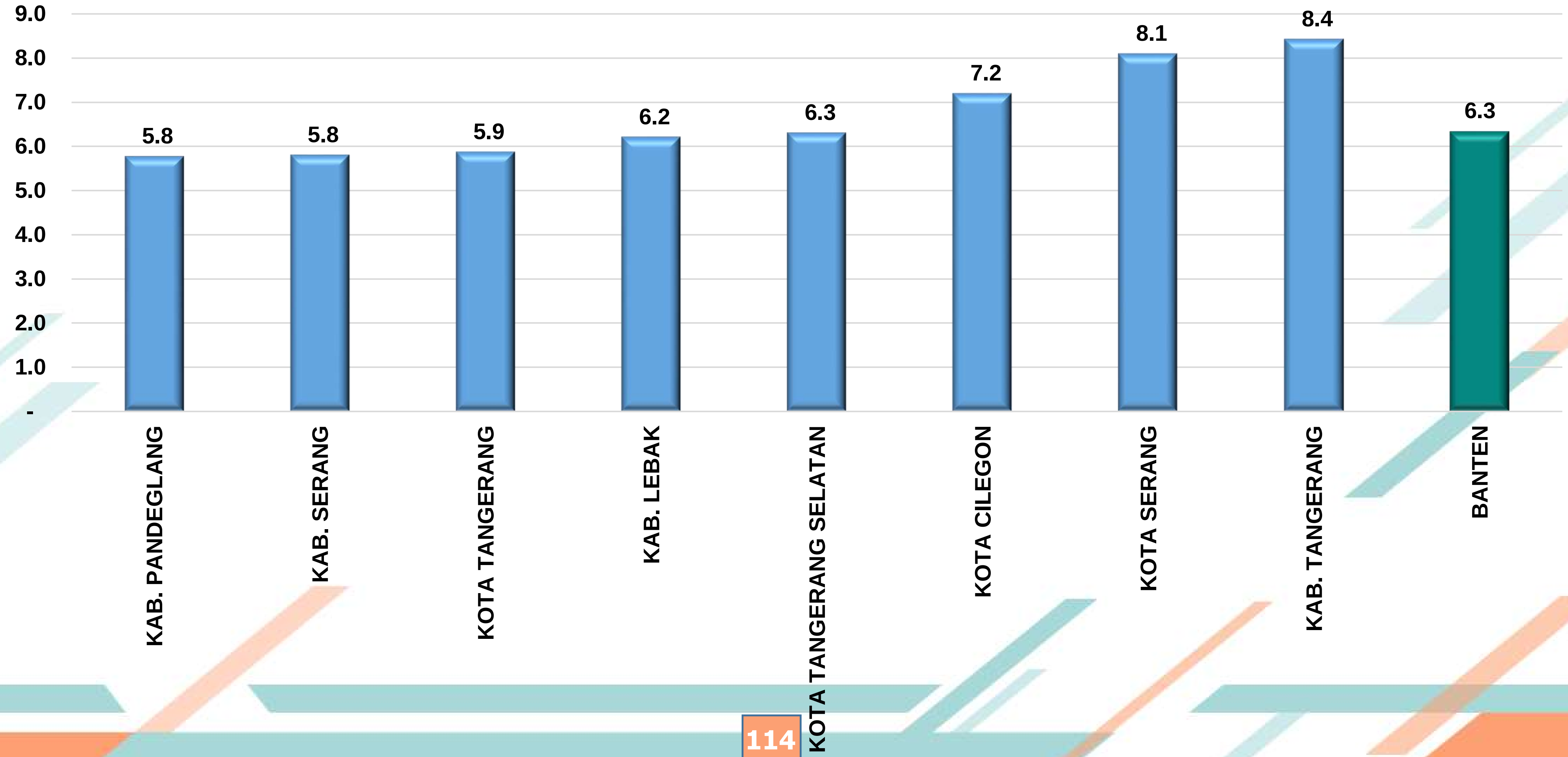

PREVALENSI BALITA *WASTED* (BERAT BADAN MENURUT TINGGI BADAN) BERDASARKAN KABUPATEN/KOTA DI PROVINSI BALI, SSGI 2021

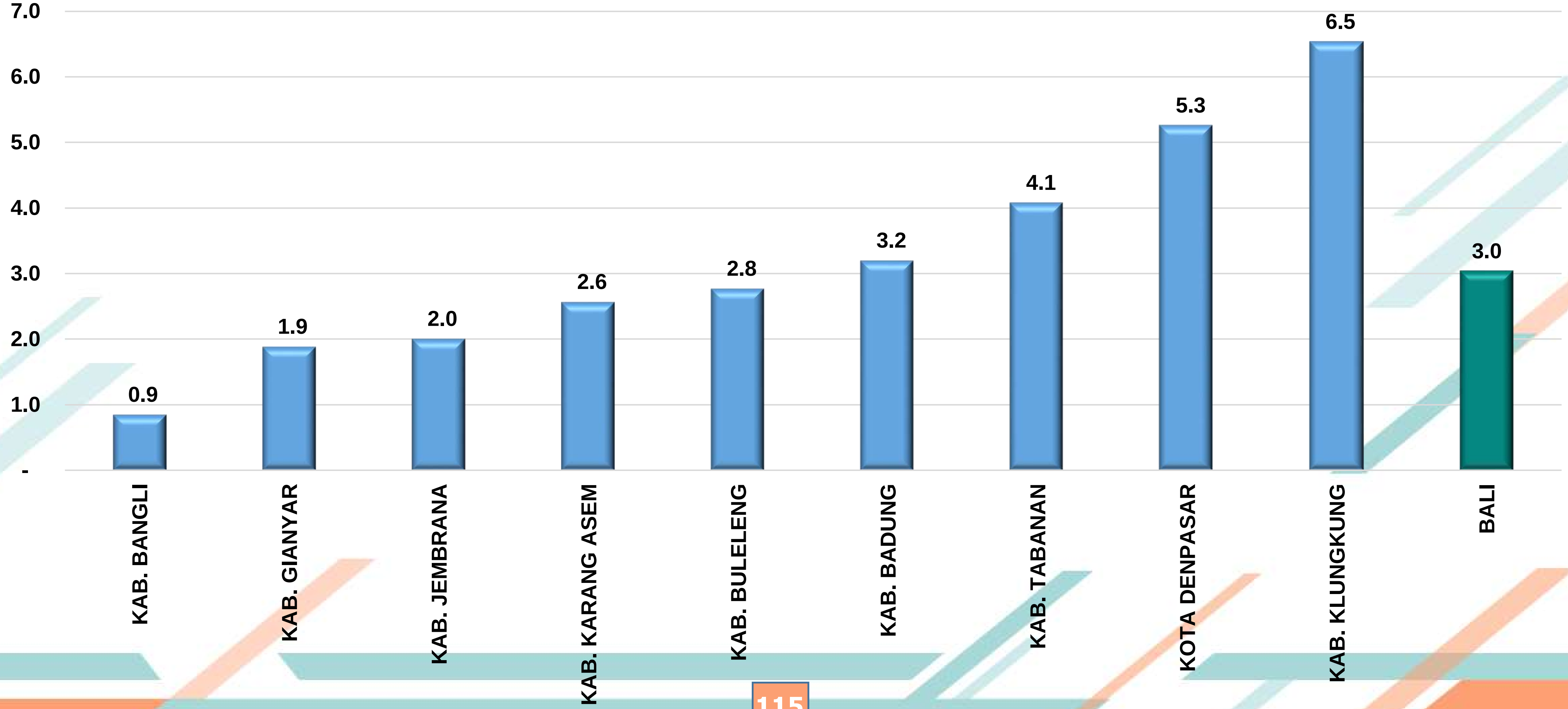

KEMENTERIAN KESEHATAN REPUBLIK INDONESIA

SSGI

PREVALENSI BALITA *WASTED* (BERAT BADAN MENURUT TINGGI BADAN) BERDASARKAN KABUPATEN/KOTA DI PROVINSI NTB, SSGI 2021

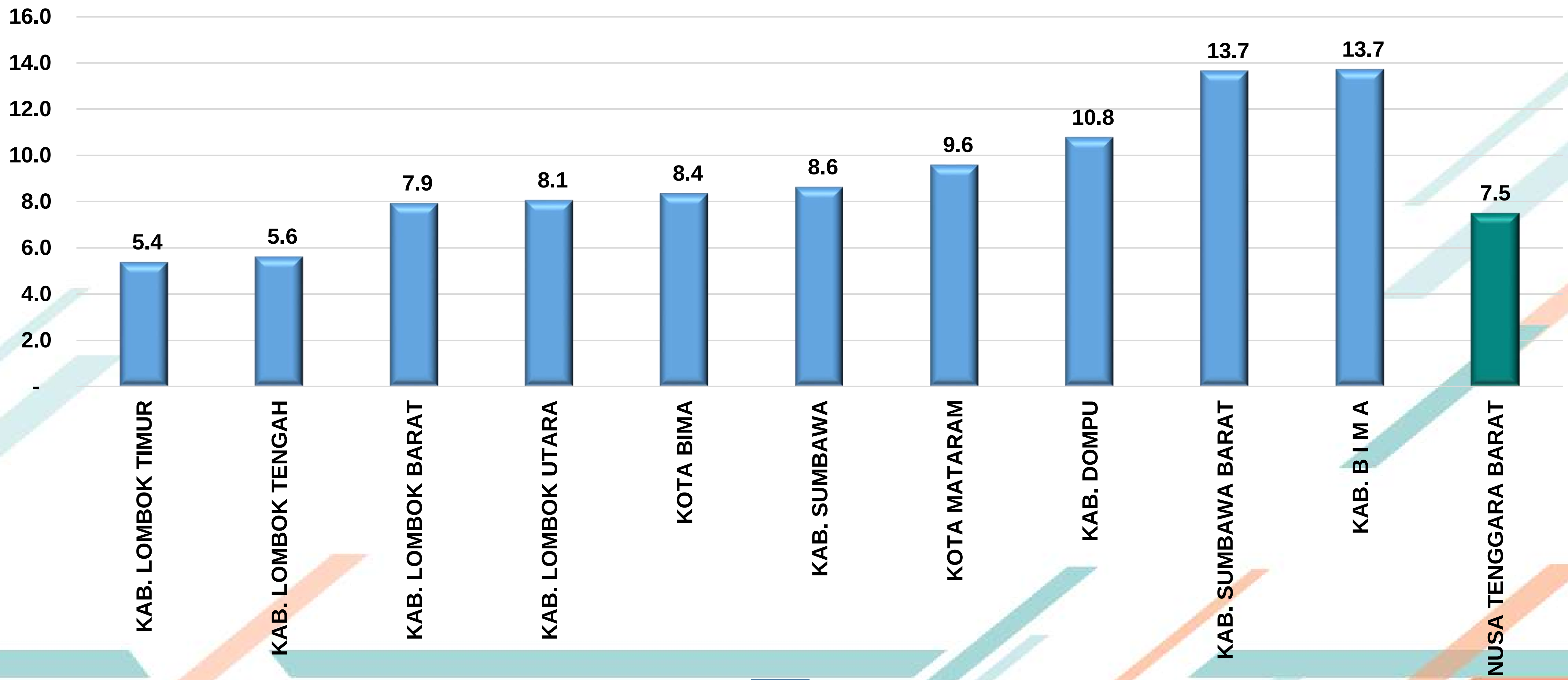

PREVALENSI BALITA *WASTED* (BERAT BADAN MENURUT TINGGI BADAN) BERDASARKAN KABUPATEN/KOTA DI PROVINSI NTT, SSGI 2021

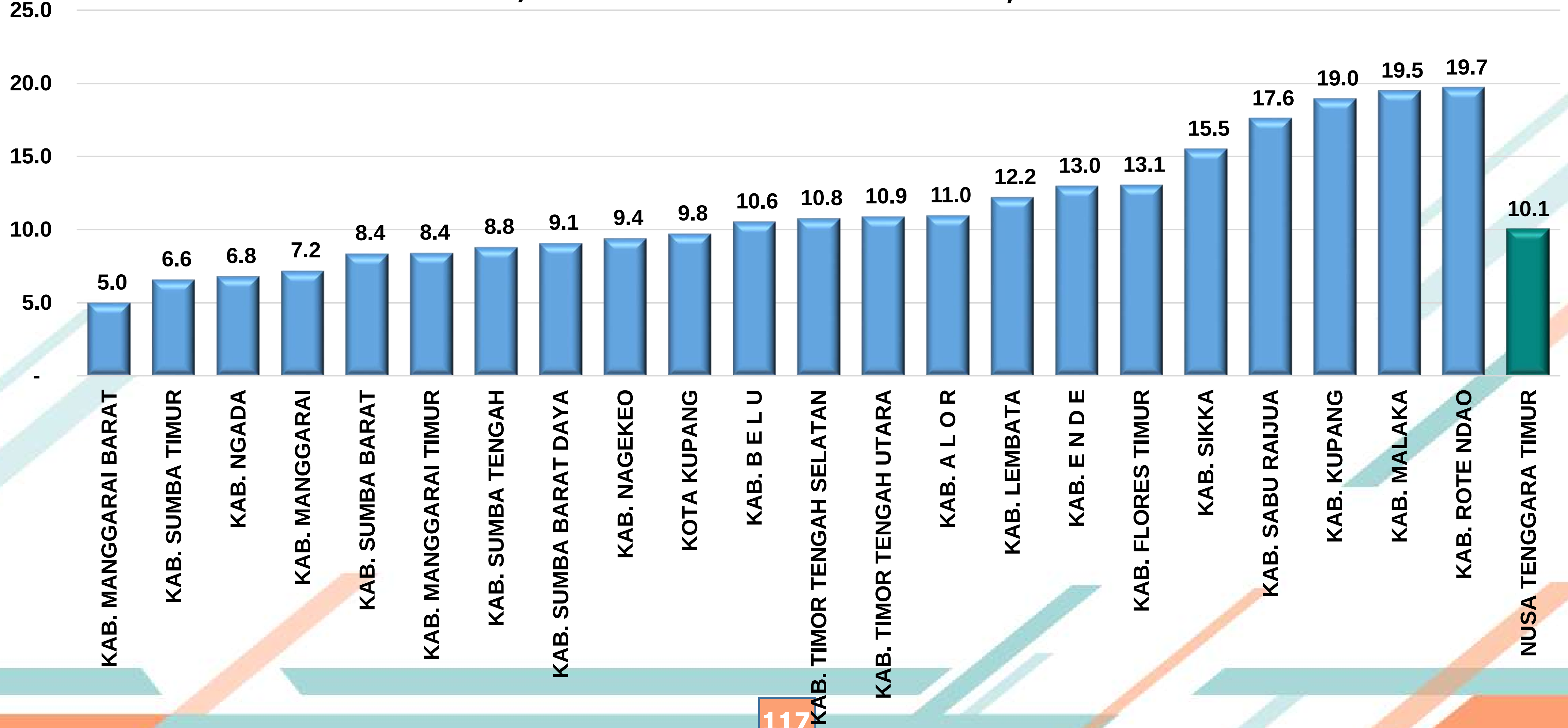

PREVALENSI BALITA *WASTED* (BERAT BADAN MENURUT TINGGI BADAN) BERDASARKAN KABUPATEN/KOTA DI PROVINSI KALIMANTAN BARAT, SSGI 2021

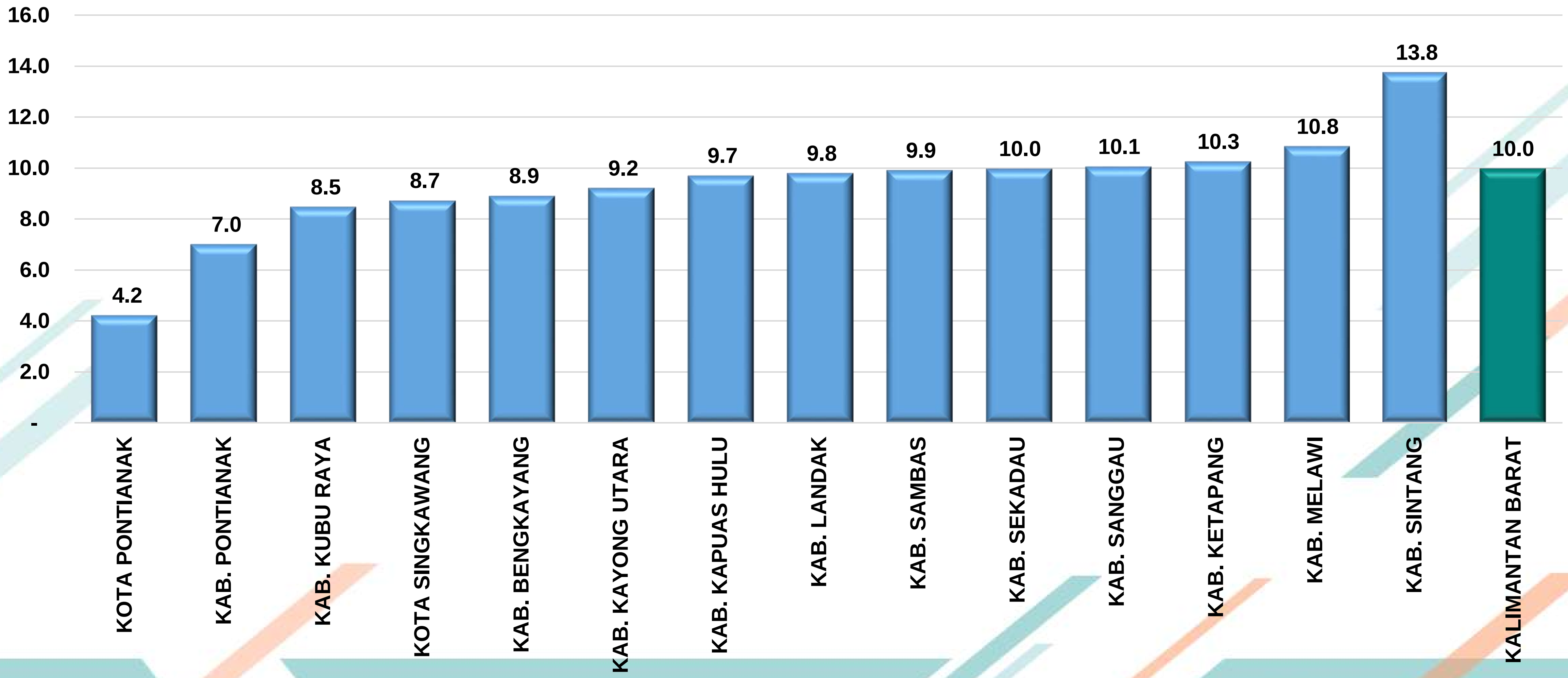

KEMENTERIAN KESEHATAN REPUBLIK INDONESIA

SSGI

PREVALENSI BALITA *WASTED* (BERAT BADAN MENURUT TINGGI BADAN) BERDASARKAN KABUPATEN/KOTA DI PROVINSI KALIMANTAN TENGAH, SSGI 2021

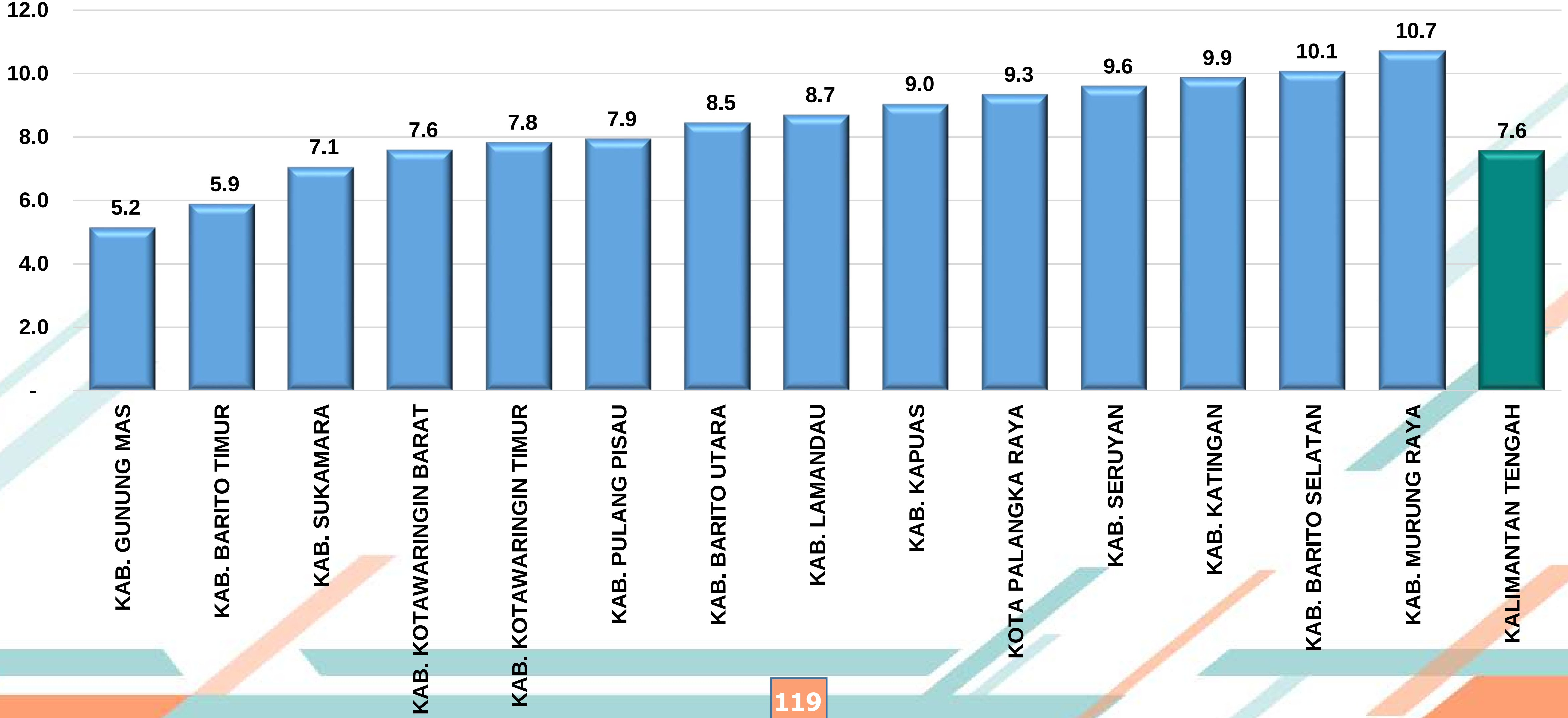

KEMENTERIAN KESEHATAN REPUBLIK INDONESIA

SSGI

PREVALENSI BALITA *WASTED* (BERAT BADAN MENURUT TINGGI BADAN) BERDASARKAN KABUPATEN/KOTA DI PROVINSI KALIMANTAN SELATAN, SSGI 2021

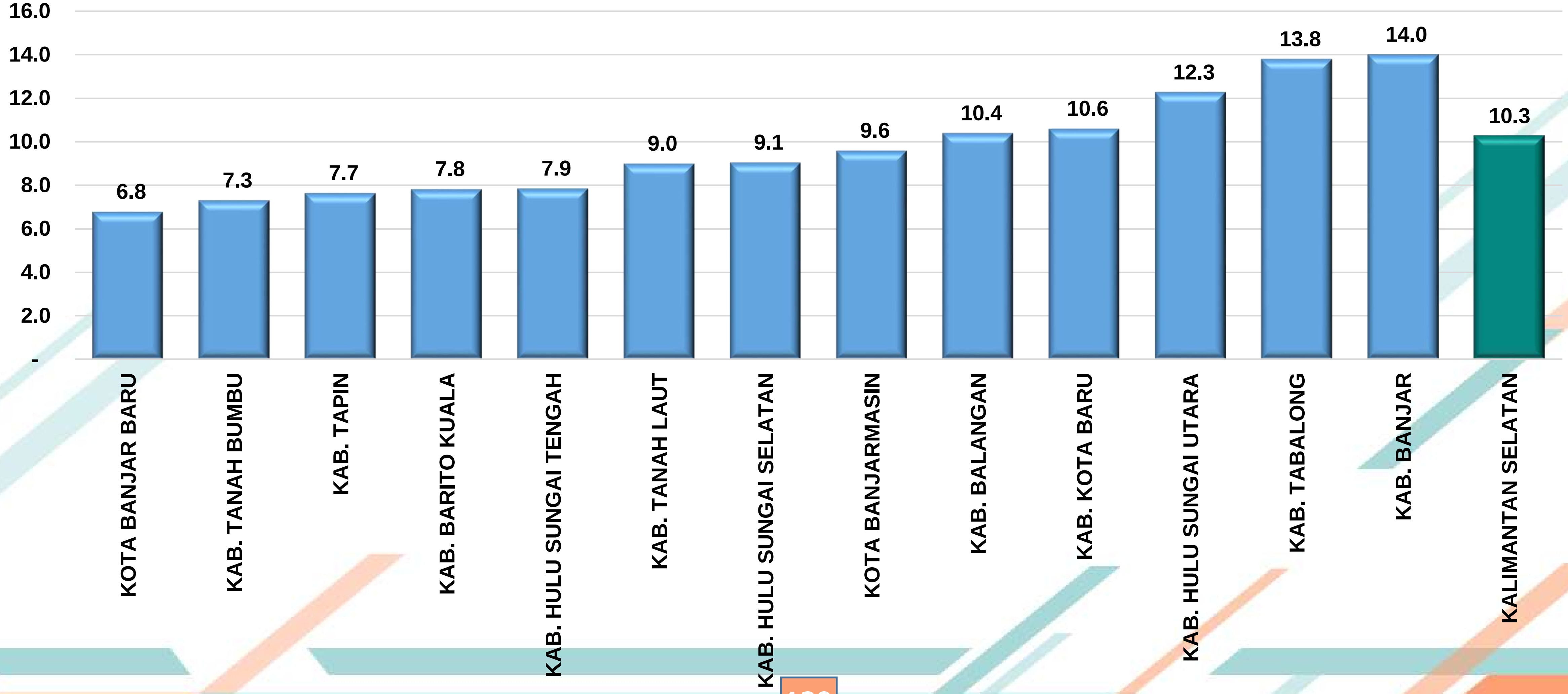

KEMENTERIAN KESEHATAN REPUBLIK INDONESIA

PREVALENSI BALITA *WASTED* (BERAT BADAN MENURUT TINGGI BADAN) BERDASARKAN KABUPATEN/KOTA DI PROVINSI KALIMANTAN TIMUR, SSGI 2021

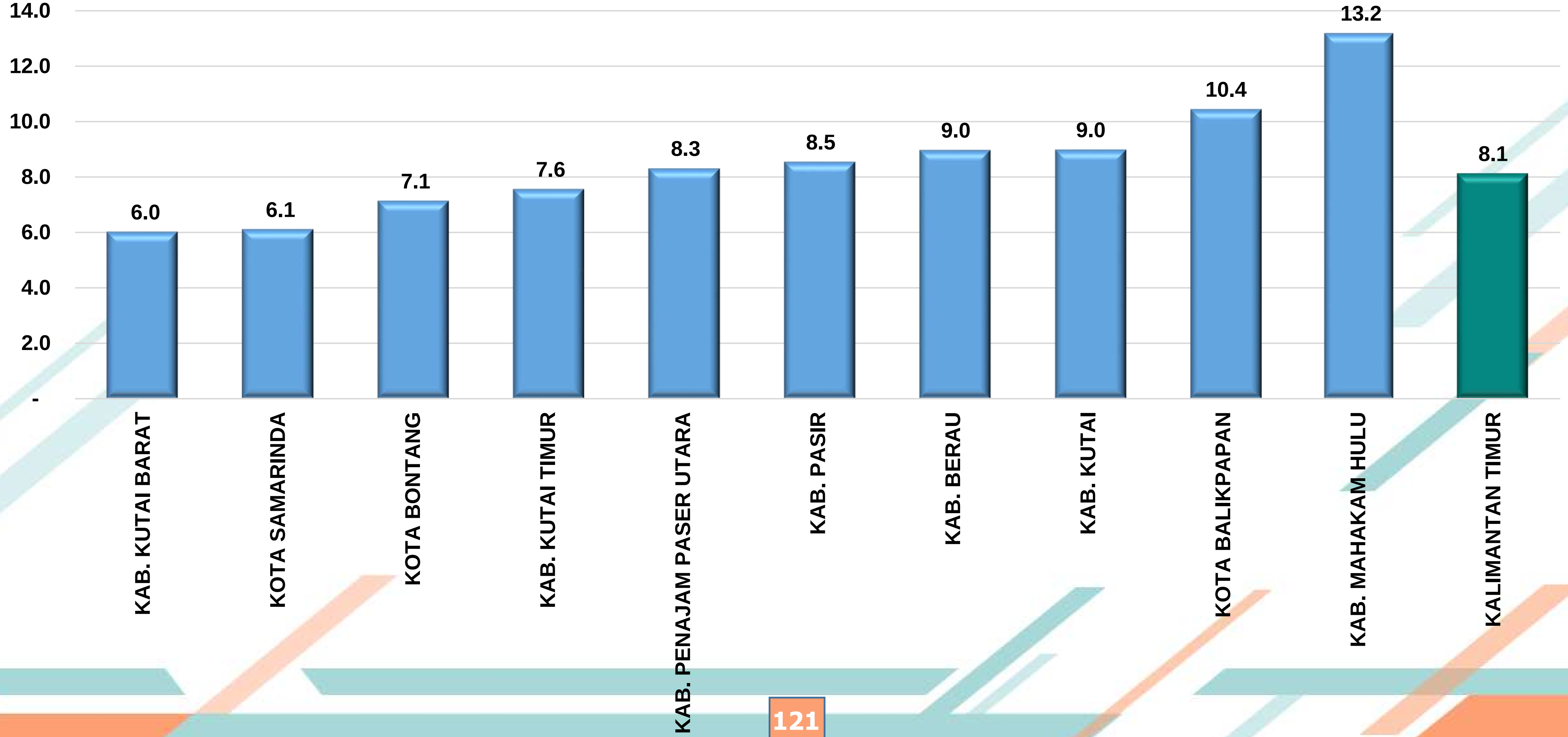

PREVALENSI BALITA *WASTED* (BERAT BADAN MENURUT TINGGI BADAN) BERDASARKAN KABUPATEN/KOTA DI PROVINSI KALIMANTAN UTARA, SSGI 2021

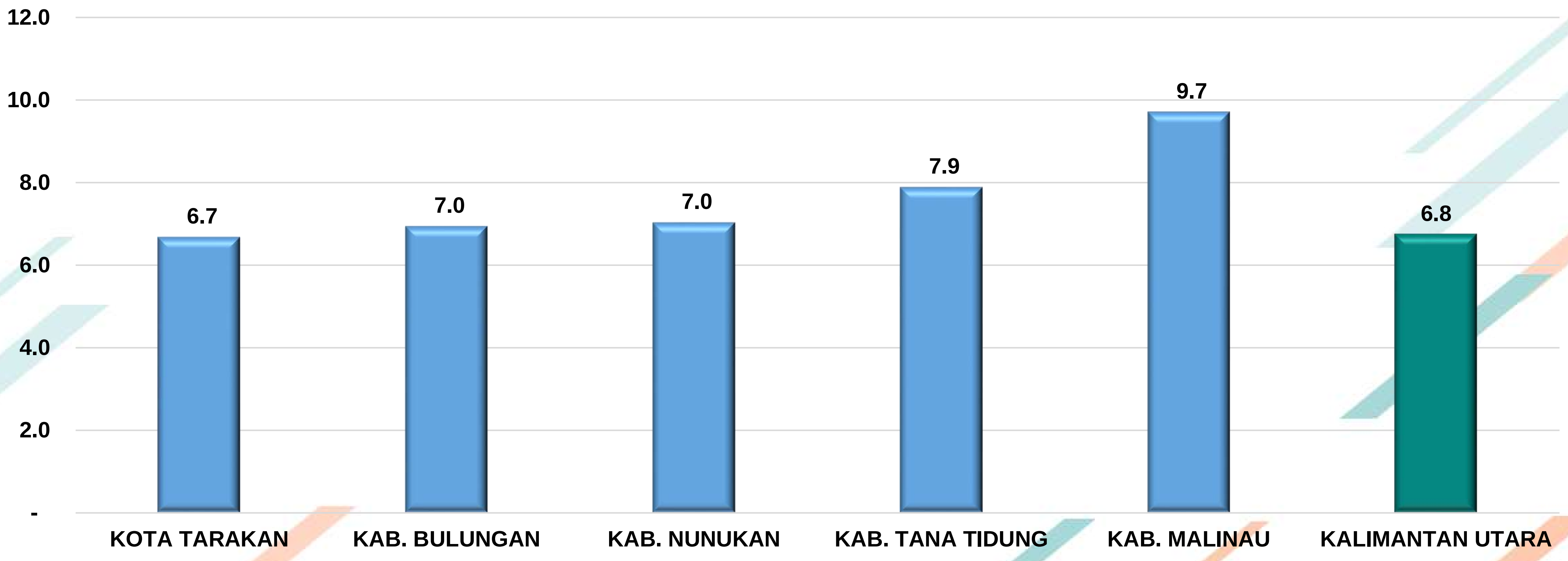

KEMENTERIAN KESEHATAN REPUBLIK INDONESIA

SSGI

PREVALENSI BALITA *WASTED* (BERAT BADAN MENURUT TINGGI BADAN) BERDASARKAN KABUPATEN/KOTA DI PROVINSI SULAWESI UTARA, SSGI 2021

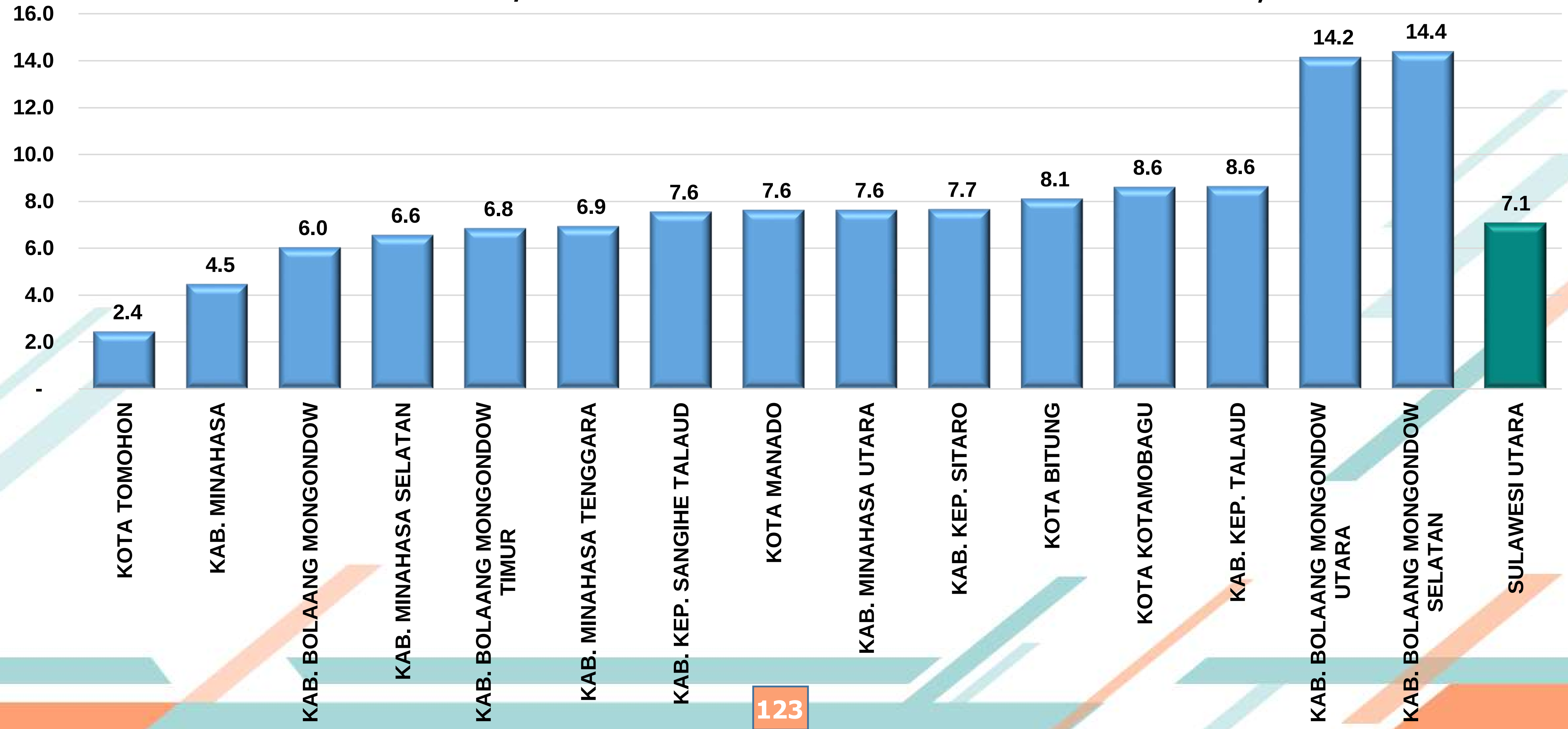

KEMENTERIAN KESEHATAN REPUBLIK INDONESIA

SSGI

PREVALENSI BALITA *WASTED* (BERAT BADAN MENURUT TINGGI BADAN) BERDASARKAN KABUPATEN/KOTA DI PROVINSI SULAWESI TENGAH, SSGI 2021

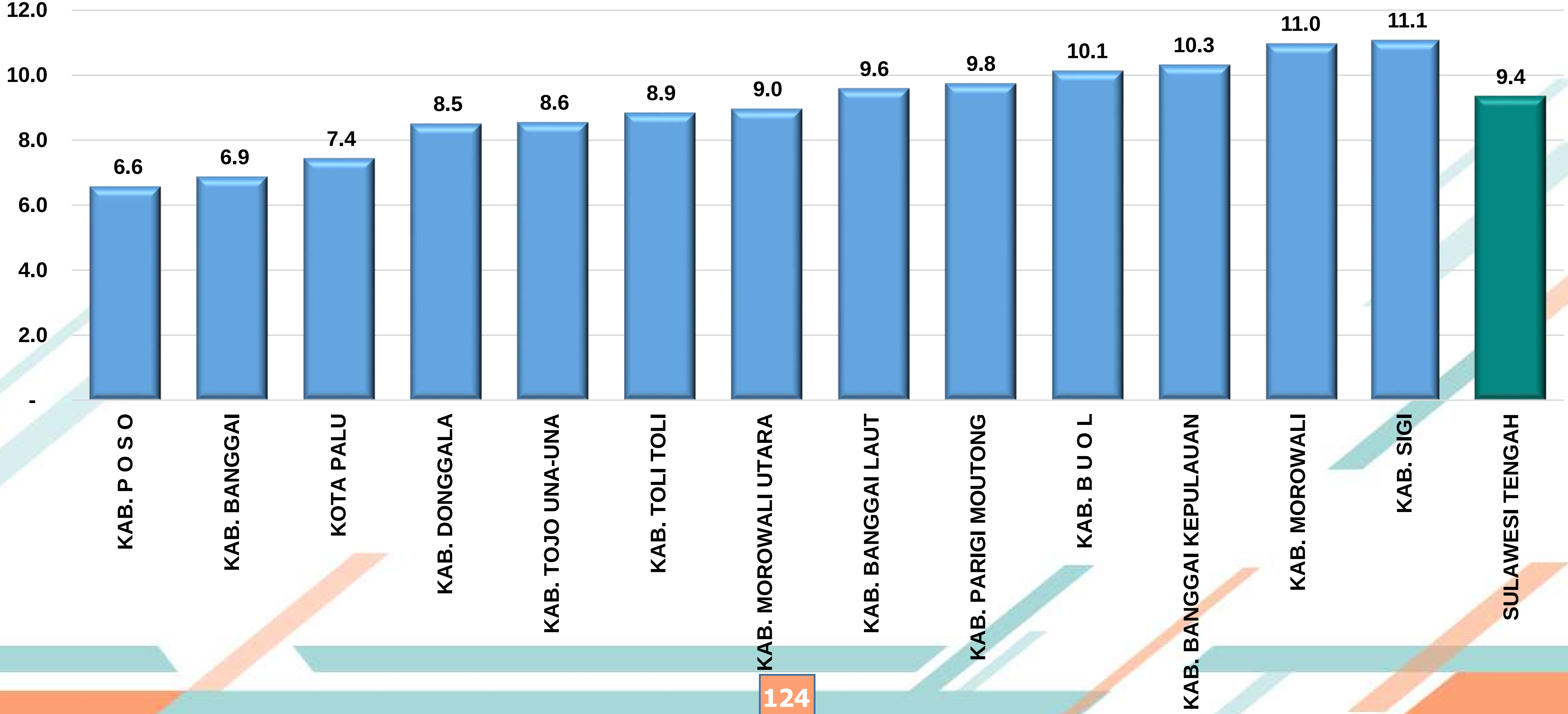

PREVALENSI BALITA *WASTED* (BERAT BADAN MENURUT TINGGI BADAN) BERDASARKAN KABUPATEN/KOTA DI PROVINSI SULAWESI SELATAN, SSGI 2021

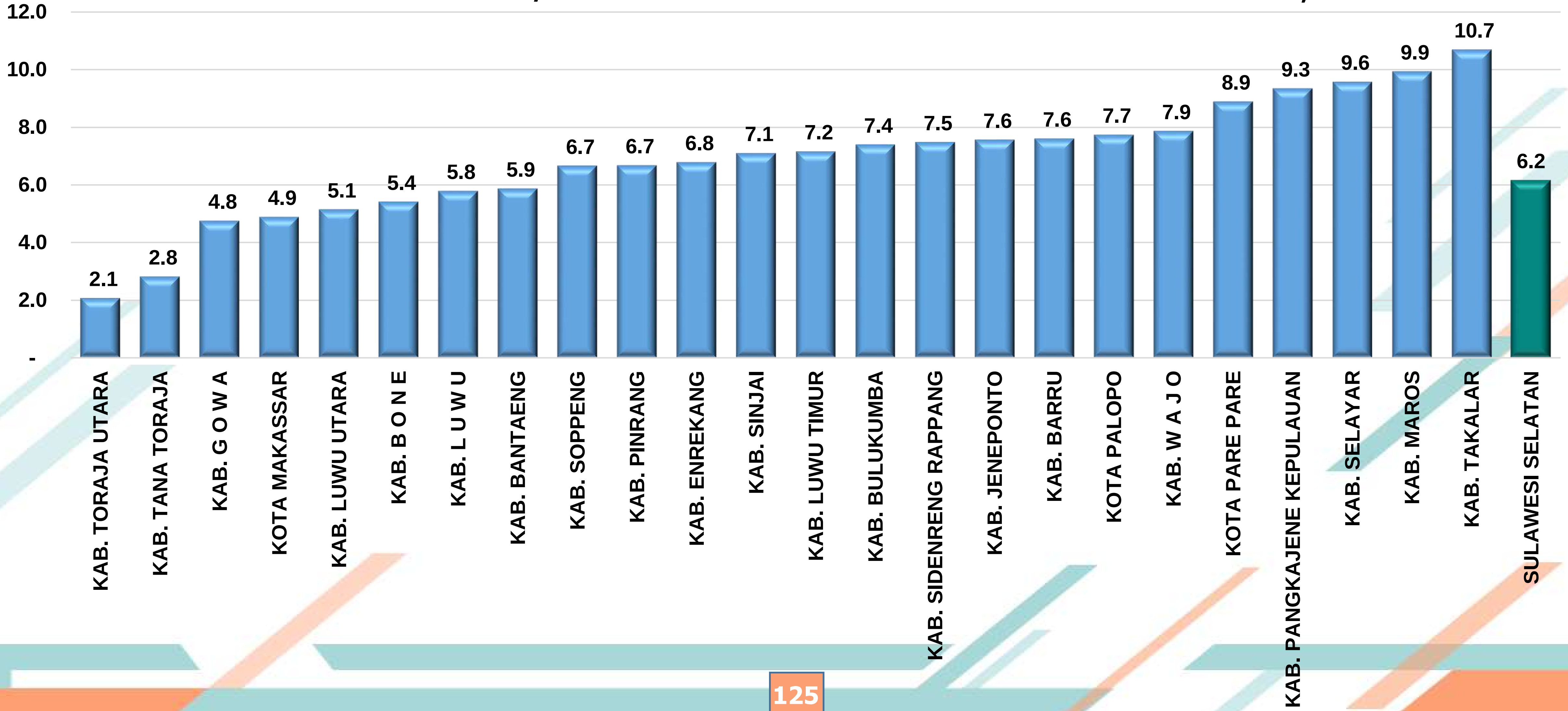

PREVALENSI BALITA *WASTED* (BERAT BADAN MENURUT TINGGI BADAN) BERDASARKAN KABUPATEN/KOTA DI PROVINSI SULAWESI TENGGARA, SSGI 2021

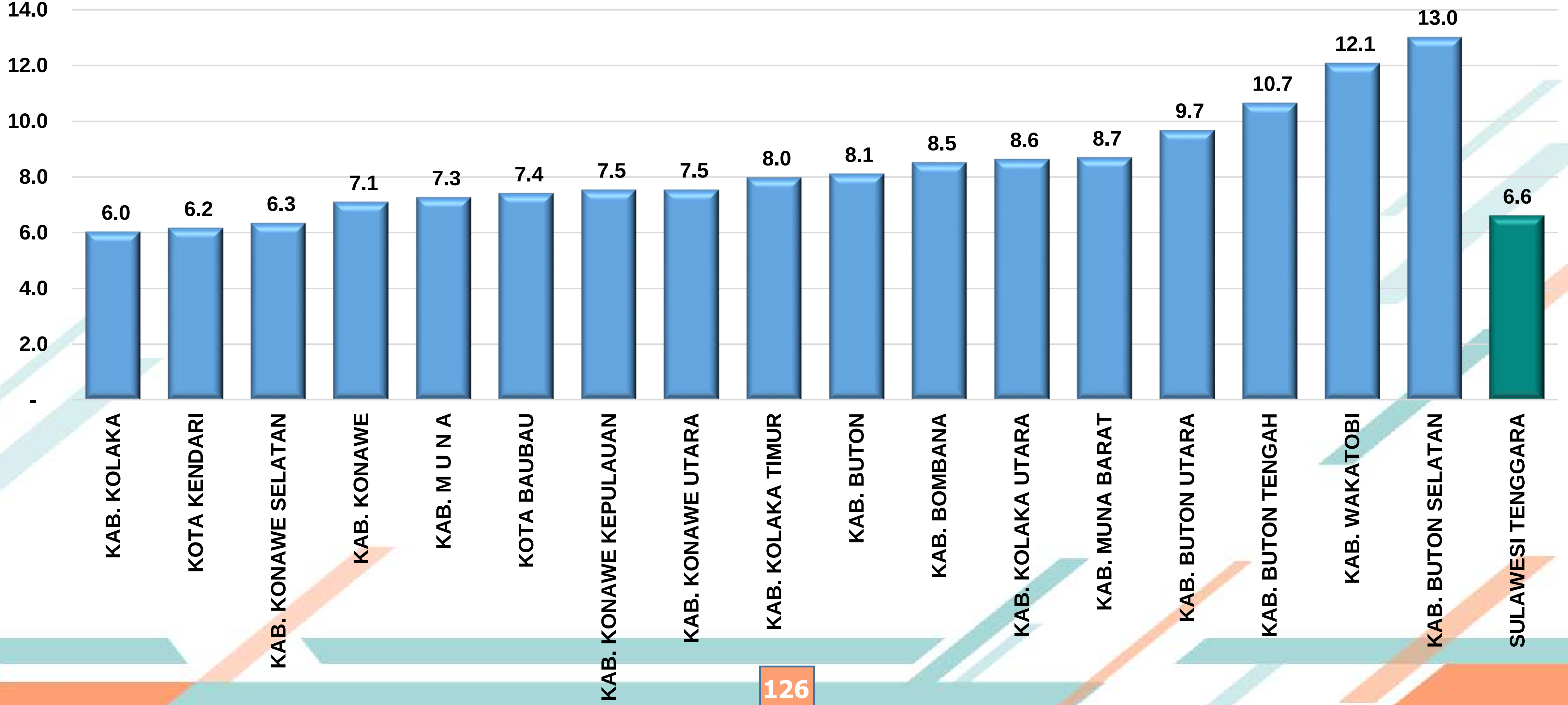

KEMENTERIAN KESEHATAN REPUBLIK INDONESIA

SSGI

PREVALENSI BALITA *WASTED* (BERAT BADAN MENURUT TINGGI BADAN) BERDASARKAN KABUPATEN/KOTA DI PROVINSI GORONTALO, SSGI 2021

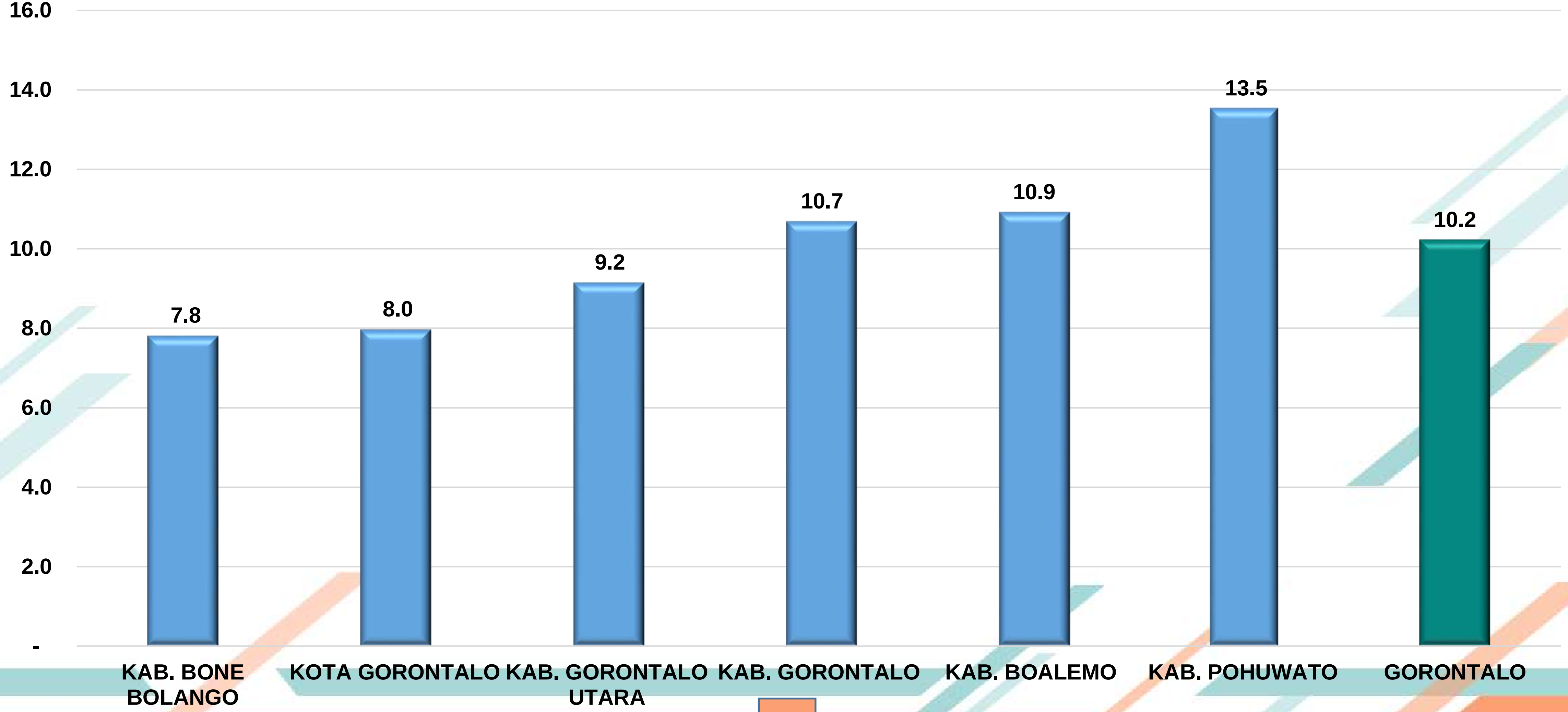

PREVALENSI BALITA *WASTED* (BERAT BADAN MENURUT TINGGI BADAN) BERDASARKAN KABUPATEN/KOTA DI PROVINSI SULAWESI BARAT, SSGI 2021

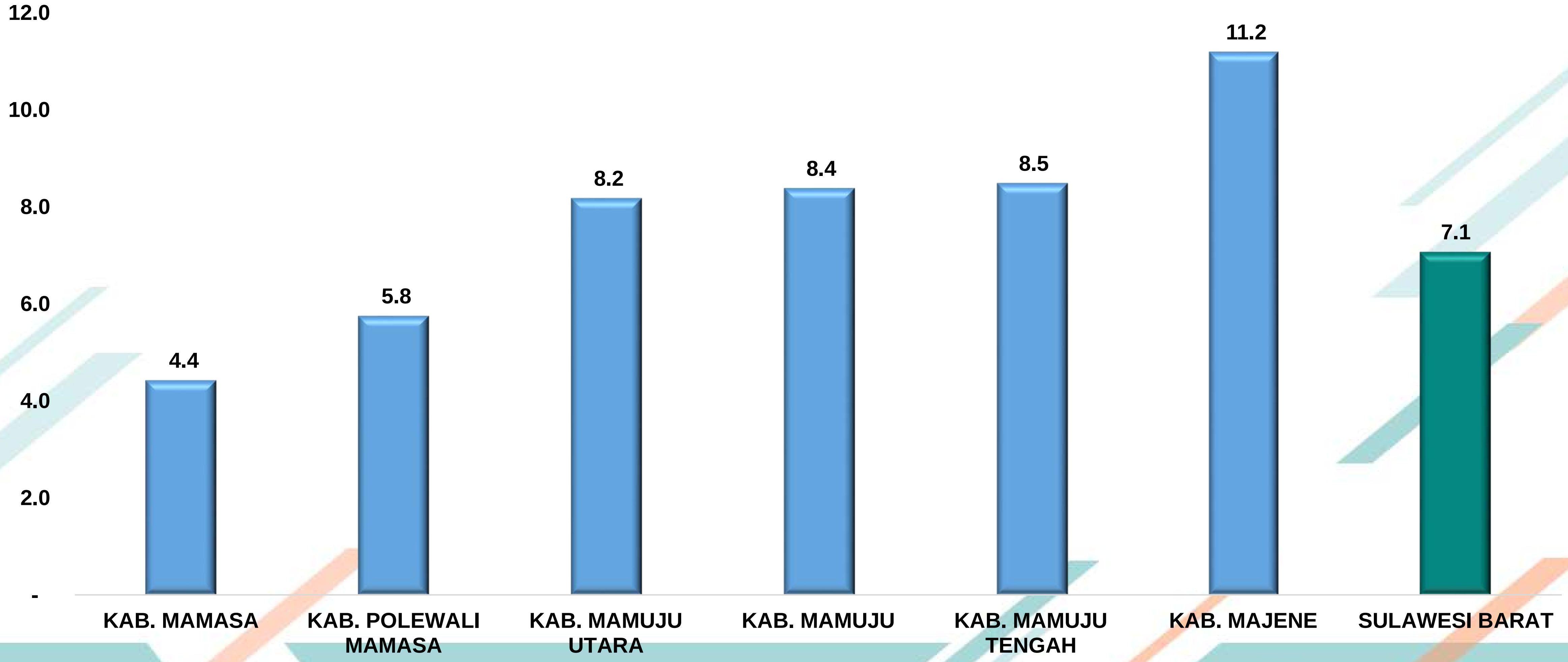

KEMENTERIAN KESEHATAN REPUBLIK INDONESIA

SSGI Studi Status Gizi Indonesia

PREVALENSI BALITA *WASTED* (BERAT BADAN MENURUT TINGGI BADAN) BERDASARKAN KABUPATEN/KOTA DI PROVINSI MALUKU, SSGI 2021

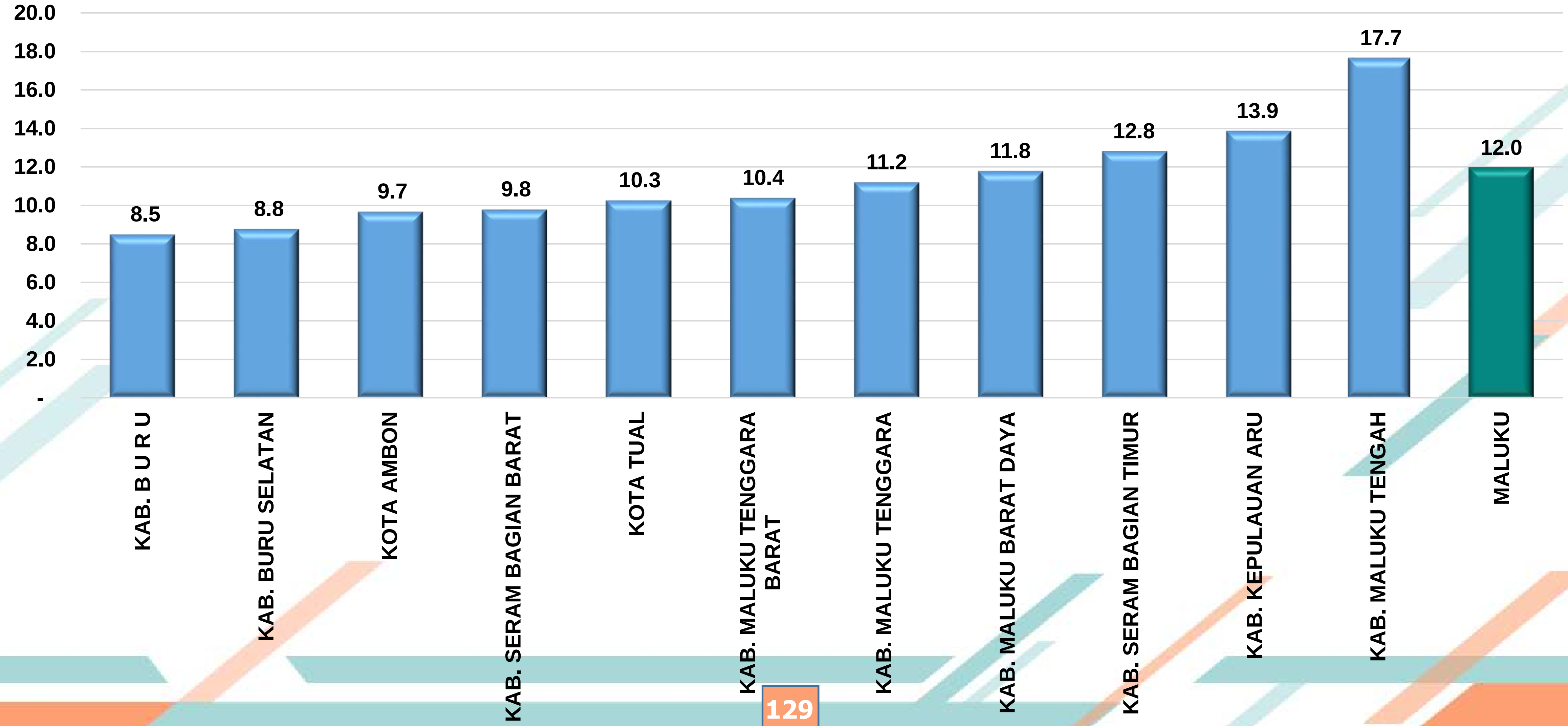

PREVALENSI BALITA *WASTED* (BERAT BADAN MENURUT TINGGI BADAN) BERDASARKAN KABUPATEN/KOTA DI PROVINSI MALUKU UTARA, SSGI 2021

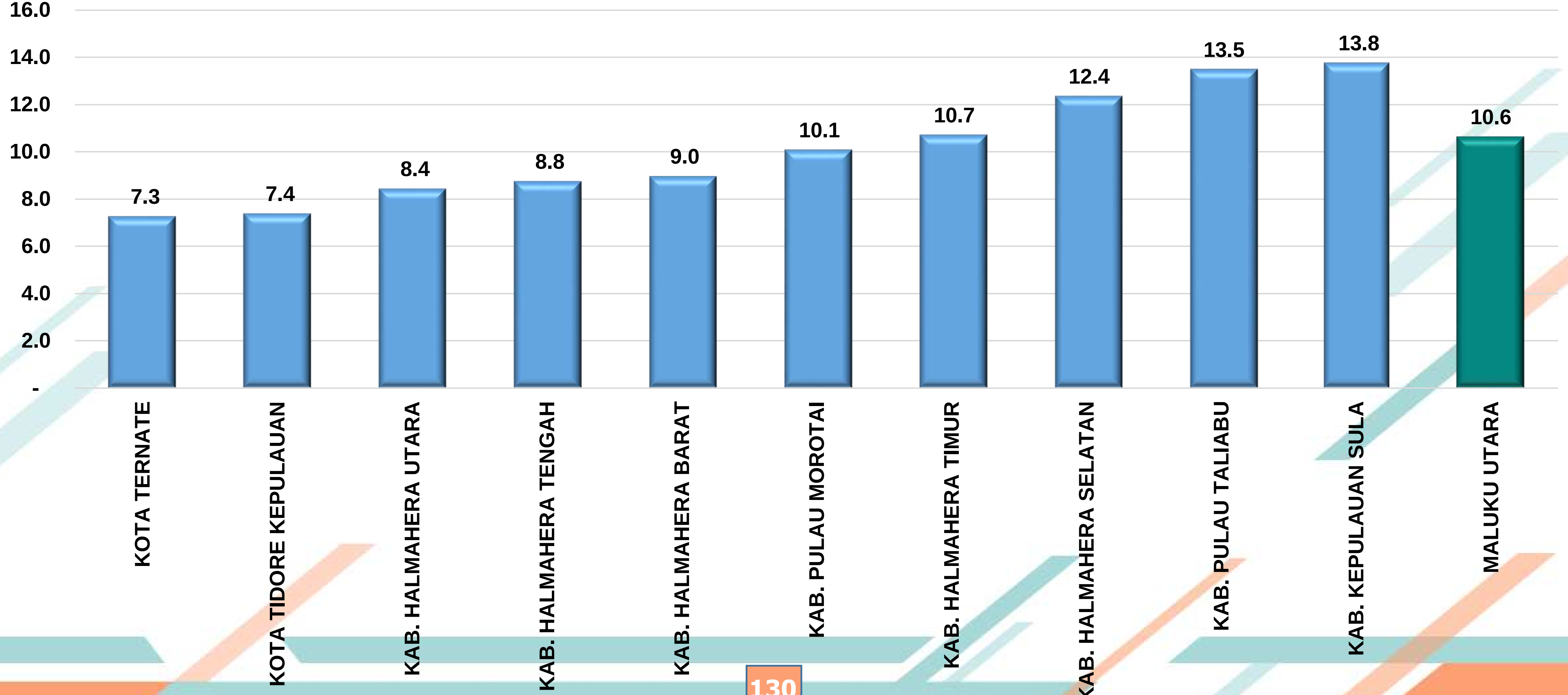

PREVALENSI BALITA *WASTED* (BERAT BADAN MENURUT TINGGI BADAN) BERDASARKAN KABUPATEN/KOTA DI PROVINSI PAPUA BARAT, SSGI 2021

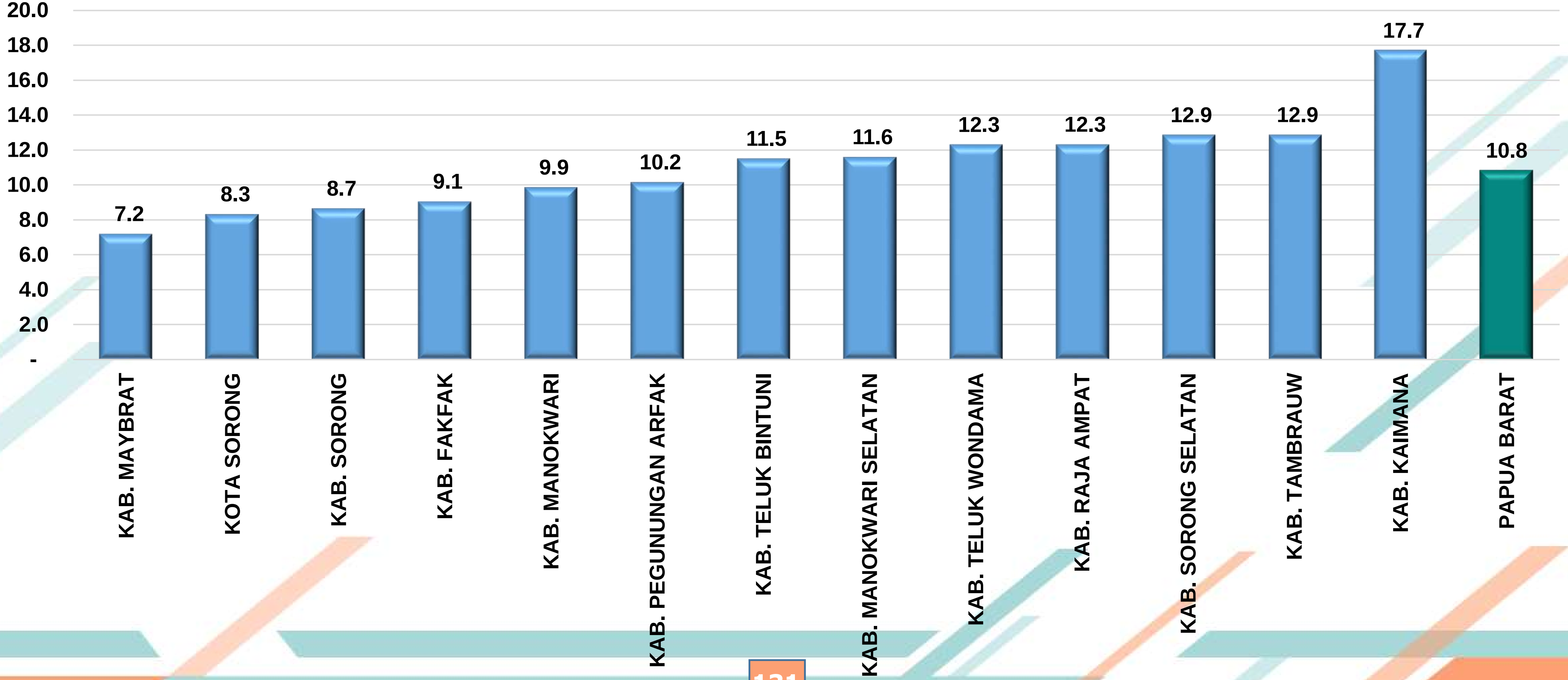

KEMENTERIAN KESEHATAN REPUBLIK INDONESIA

SSGI

PREVALENSI BALITA *WASTED* (BERAT BADAN MENURUT TINGGI BADAN) BERDASARKAN KABUPATEN/KOTA DI PROVINSI PAPUA, SSGI 2021

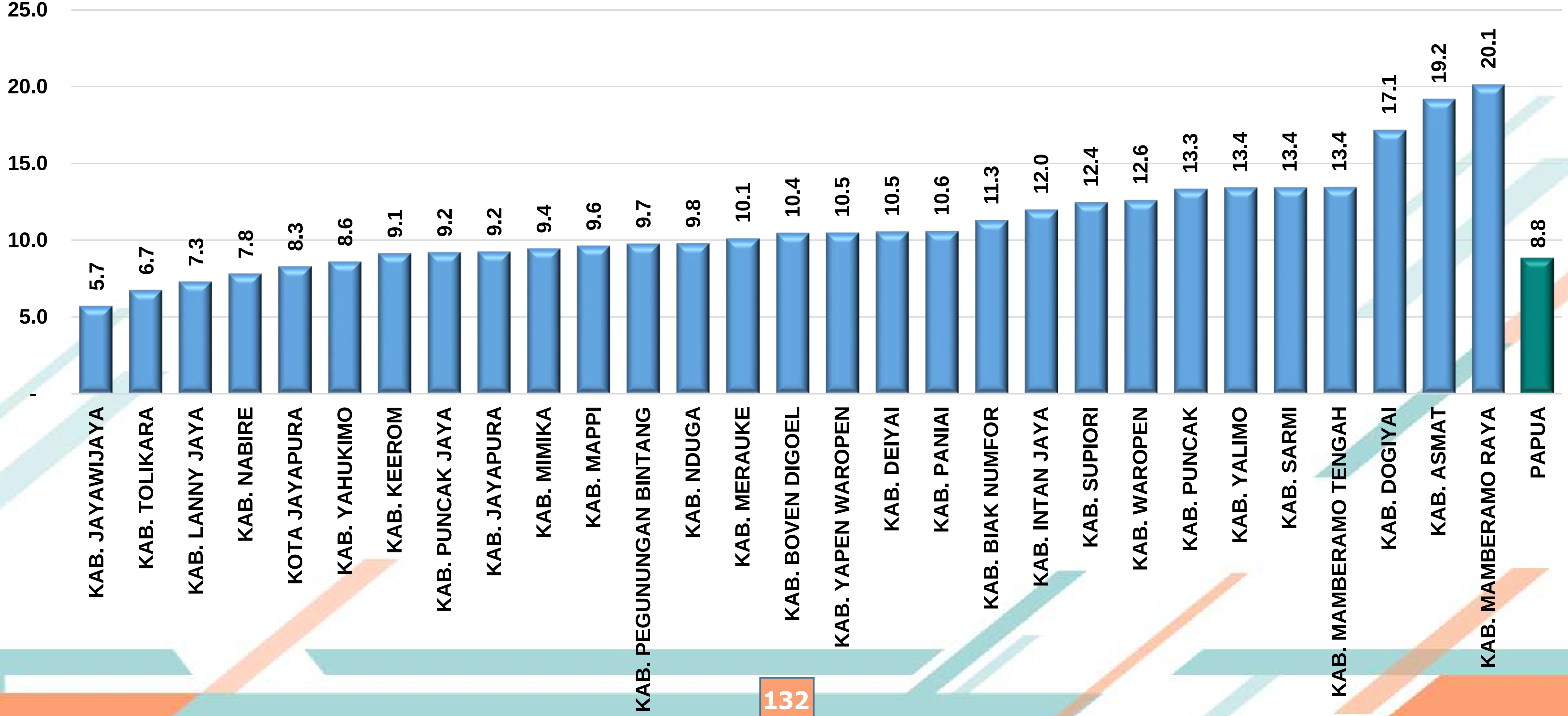

ANGKA *UNDERWEIGHT*
TINGKAT KABUPATEN DAN KOTA

KEMENTERIAN KESEHATAN REPUBLIK INDONESIA

SSGI
Studi Status Gizi Indonesia

PREVALENSI BALITA *UNDERWEIGHT* (BERAT BADAN MENURUT UMUR) BERDASARKAN KABUPATEN/KOTA DI PROVINSI ACEH, SSGI 2021

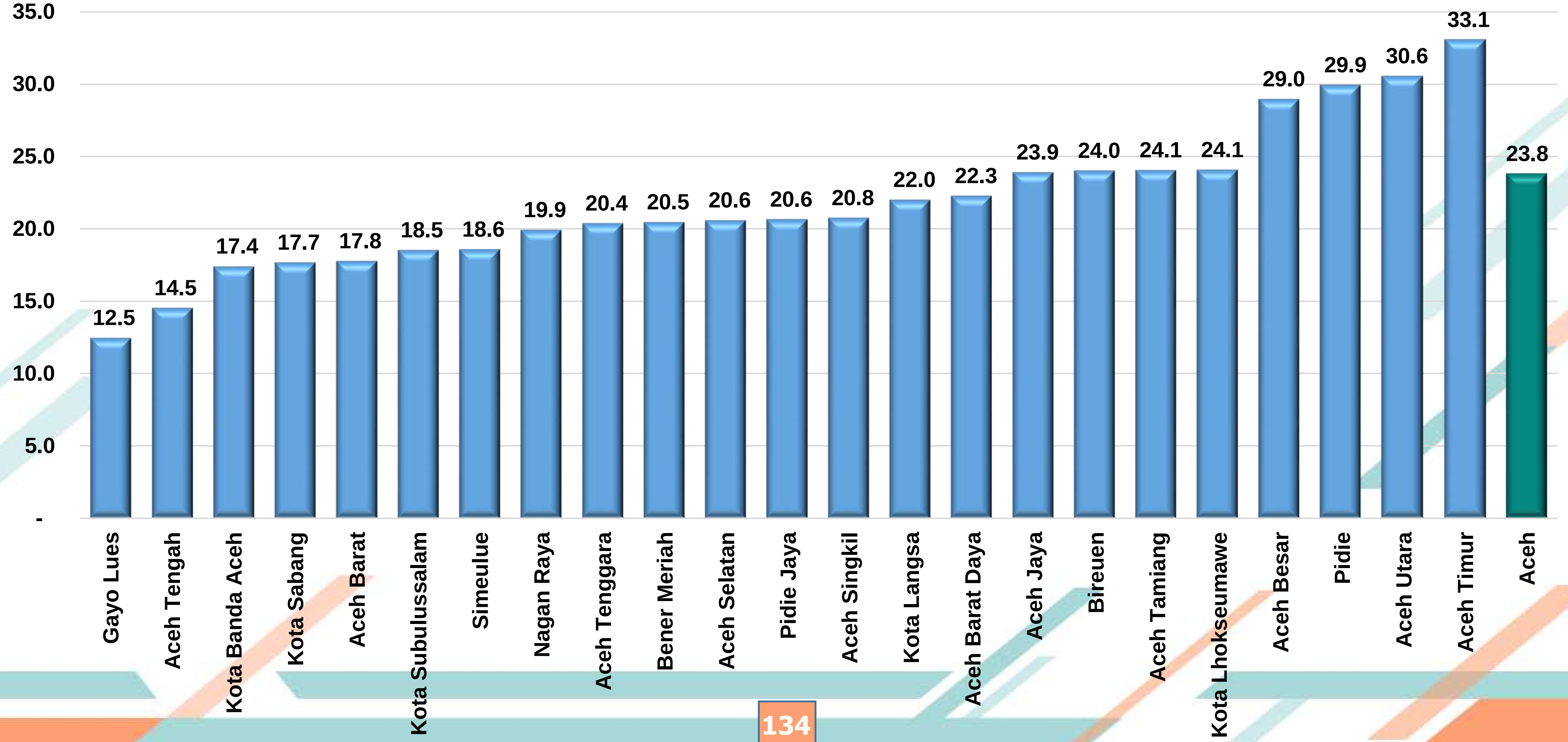

KEMENTERIAN KESEHATAN REPUBLIK INDONESIA

SSGI Studi Status Gizi Indonesia

PREVALENSI BALITA *UNDERWEIGHT* (BERAT BADAN MENURUT UMUR) BERDASARKAN KABUPATEN/KOTA DI PROVINSI SUMATERA UTARA, SSGI 2021

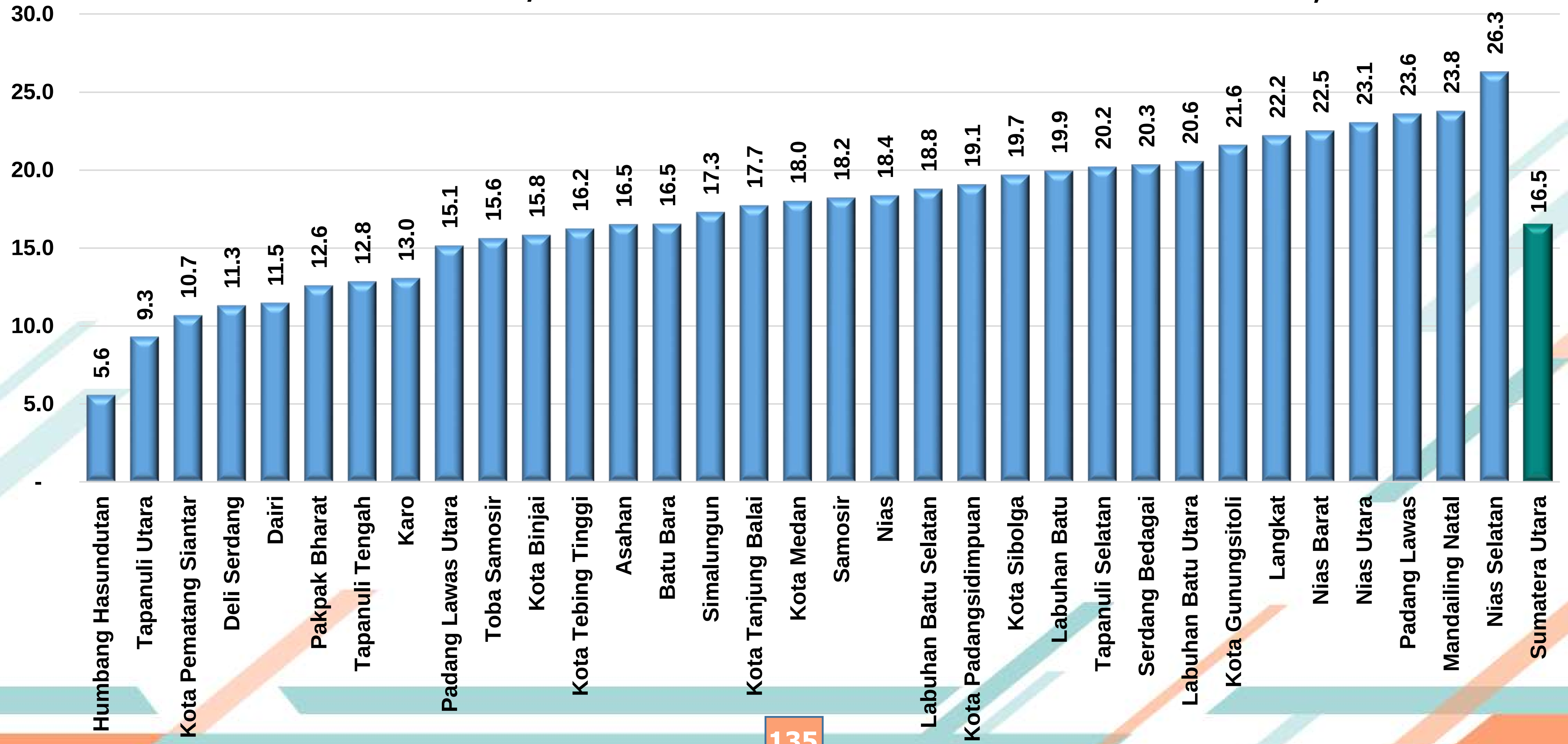

KEMENTERIAN KESEHATAN REPUBLIK INDONESIA

SSGI Studi Status Gizi Indonesia

PREVALENSI BALITA *UNDERWEIGHT* (BERAT BADAN MENURUT UMUR) BERDASARKAN KABUPATEN/KOTA DI PROVINSI SUMATERA BARAT, SSGI 2021

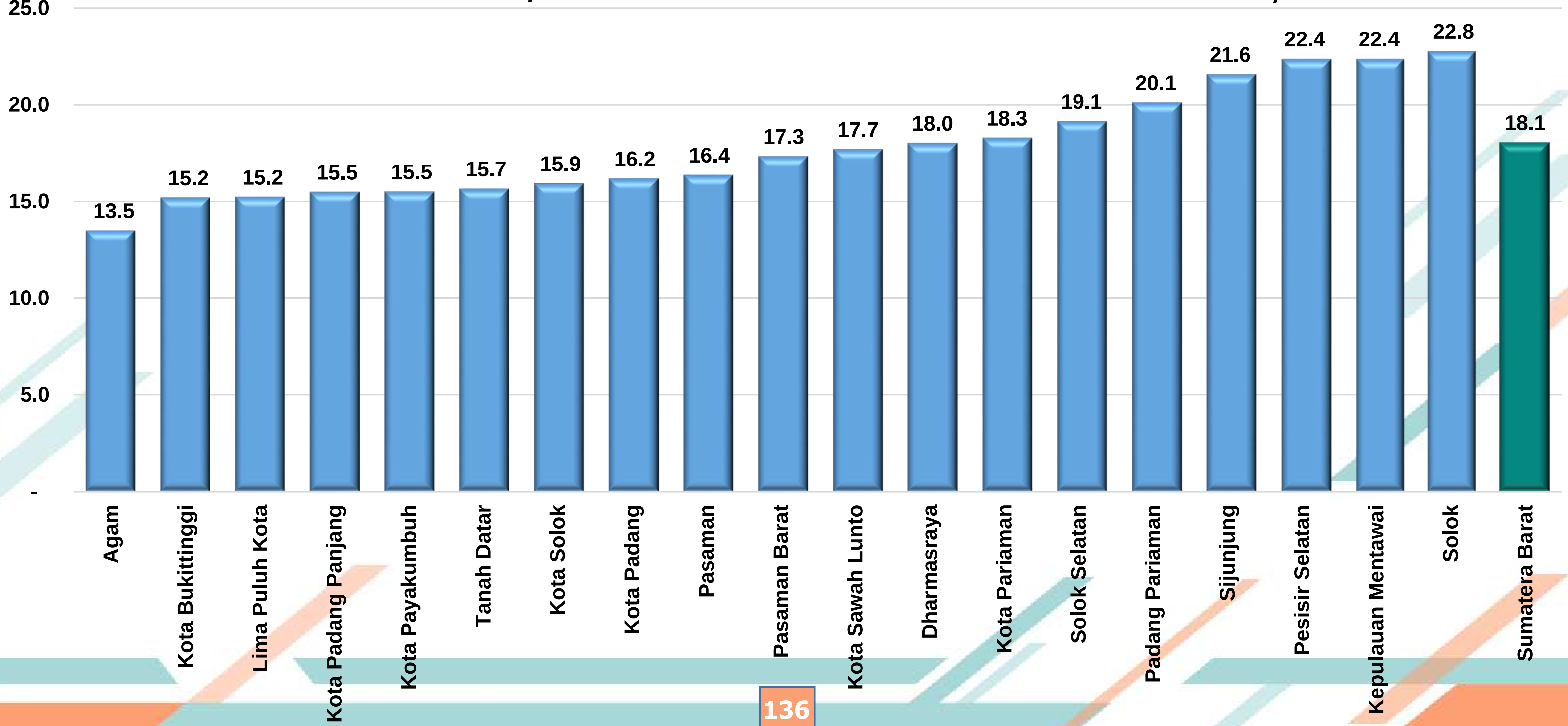

PREVALENSI BALITA *UNDERWEIGHT* (BERAT BADAN MENURUT UMUR) BERDASARKAN KABUPATEN/KOTA DI PROVINSI RIAU, SSGI 2021

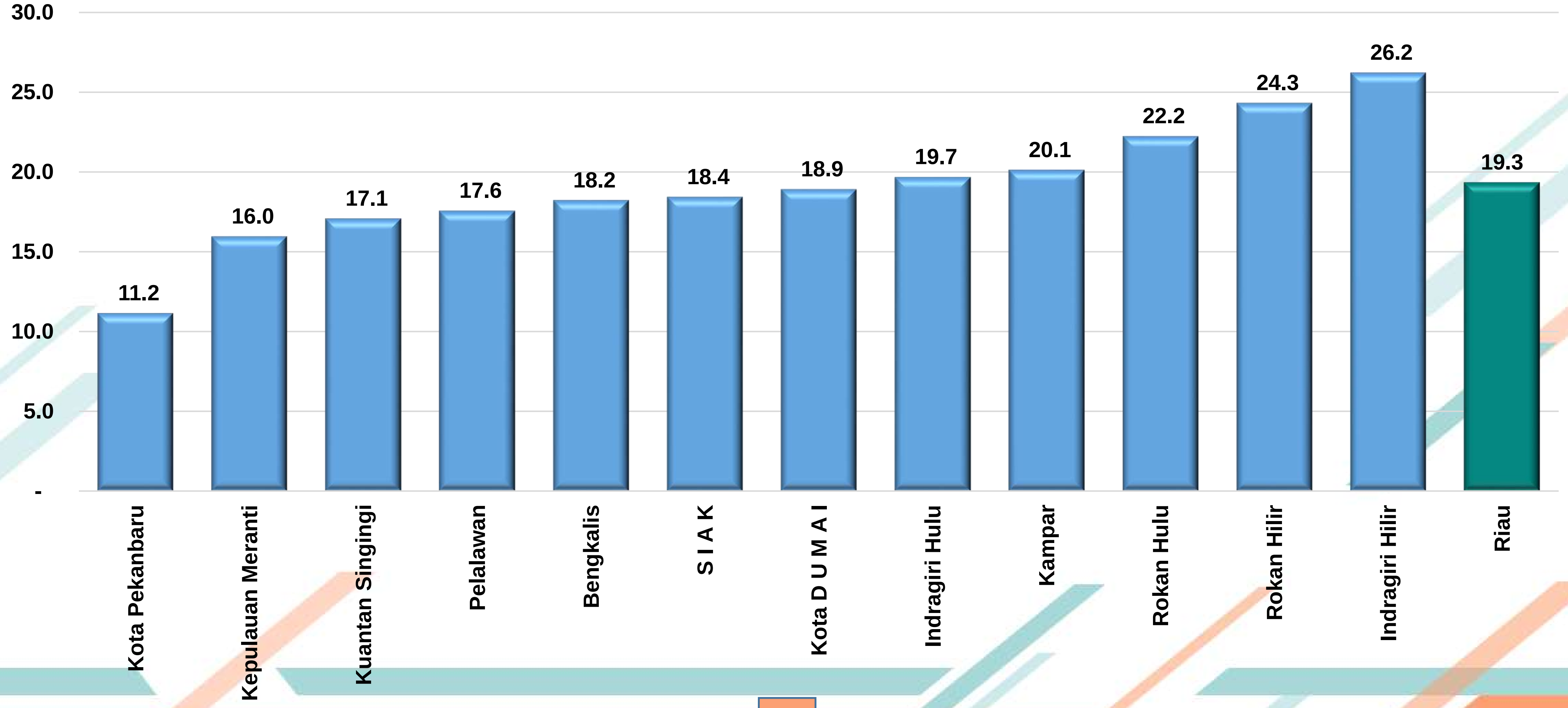

PREVALENSI BALITA *UNDERWEIGHT* (BERAT BADAN MENURUT UMUR) BERDASARKAN KABUPATEN/KOTA DI PROVINSI JAMBI, SSGI 2021

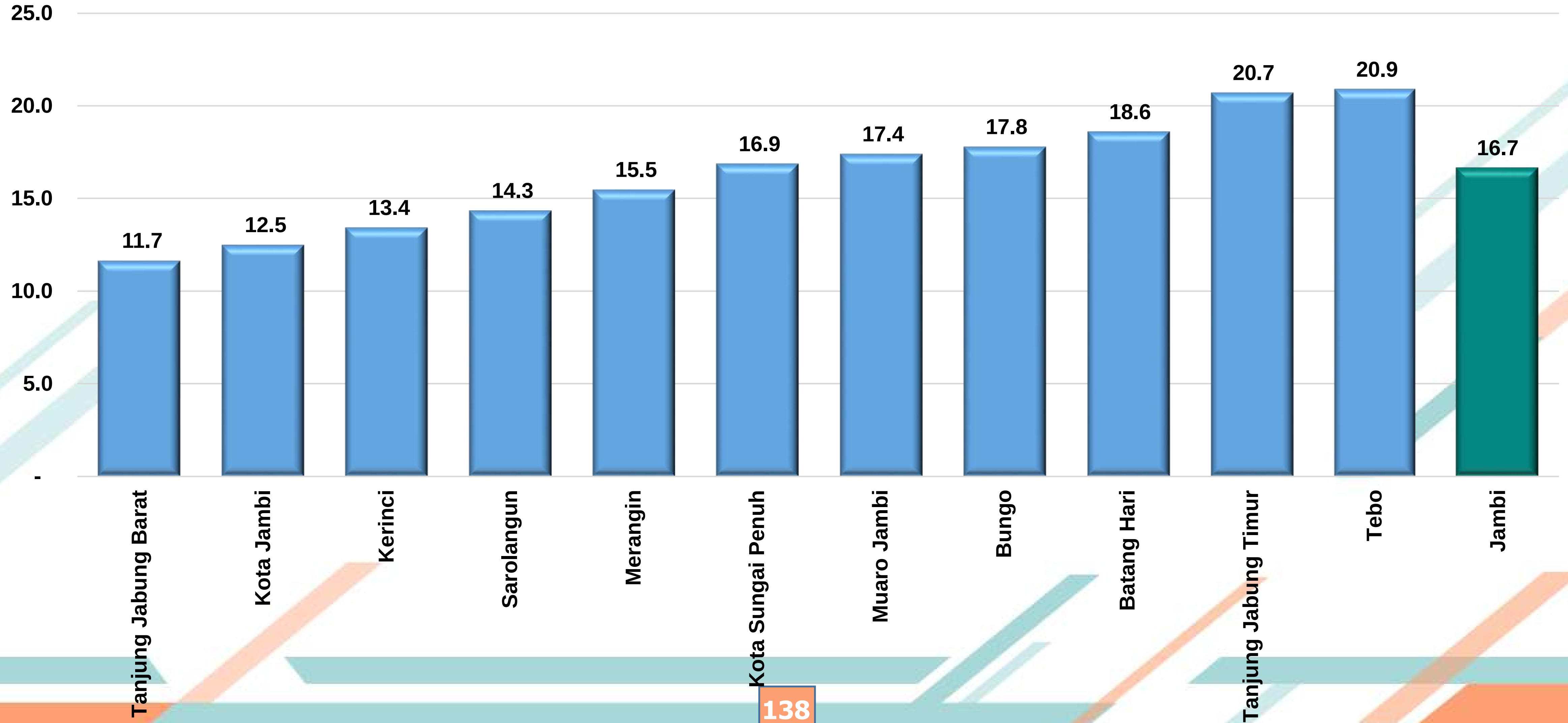

KEMENTERIAN KESEHATAN REPUBLIK INDONESIA

SSGI Studi Status Gizi Indonesia

PREVALENSI BALITA *UNDERWEIGHT* (BERAT BADAN MENURUT UMUR) BERDASARKAN KABUPATEN/KOTA DI PROVINSI SUMATERA SELATAN, SSGI 2021

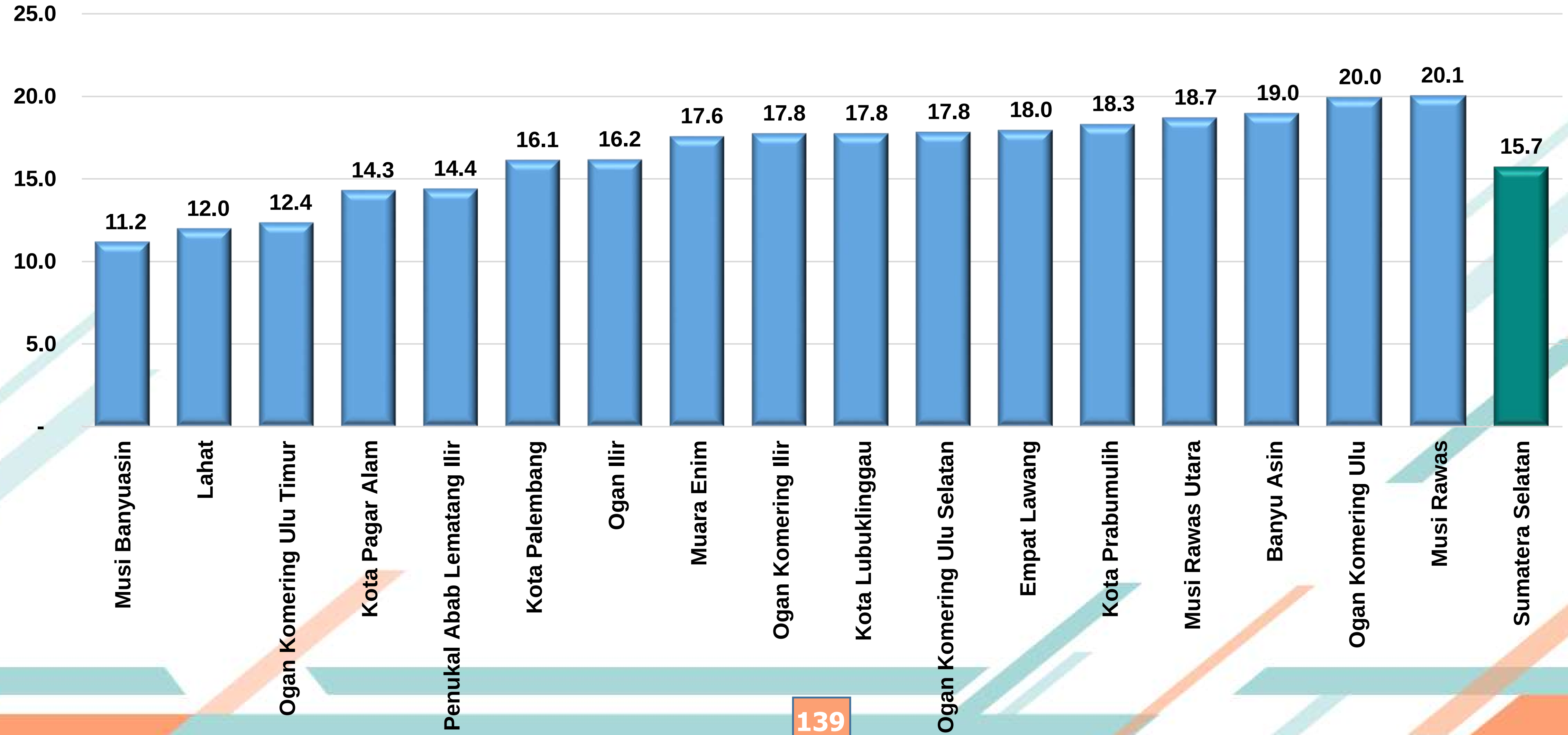

PREVALENSI BALITA *UNDERWEIGHT* (BERAT BADAN MENURUT UMUR) BERDASARKAN KABUPATEN/KOTA DI PROVINSI BENGKULU, SSGI 2021

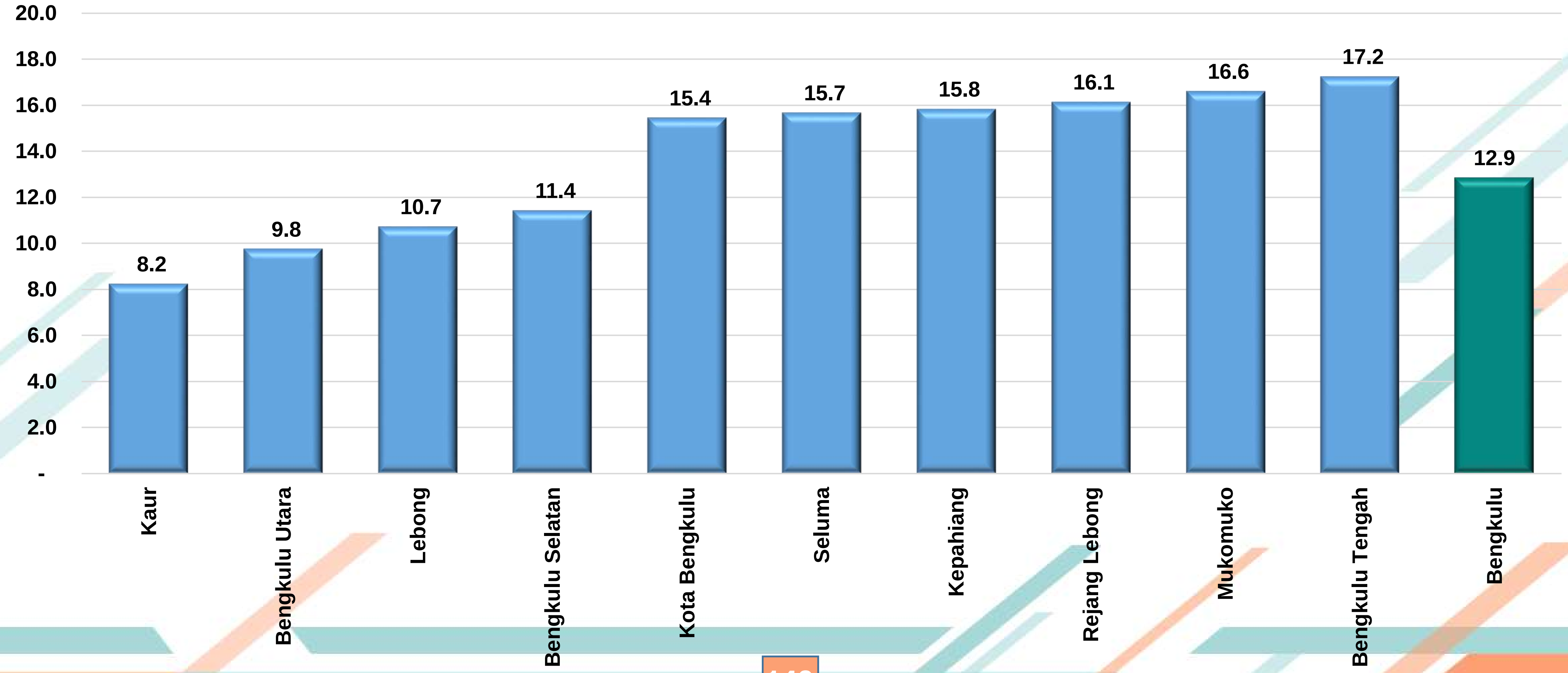

PREVALENSI BALITA *UNDERWEIGHT* (BERAT BADAN MENURUT UMUR) BERDASARKAN KABUPATEN/KOTA DI PROVINSI LAMPUNG, SSGI 2021

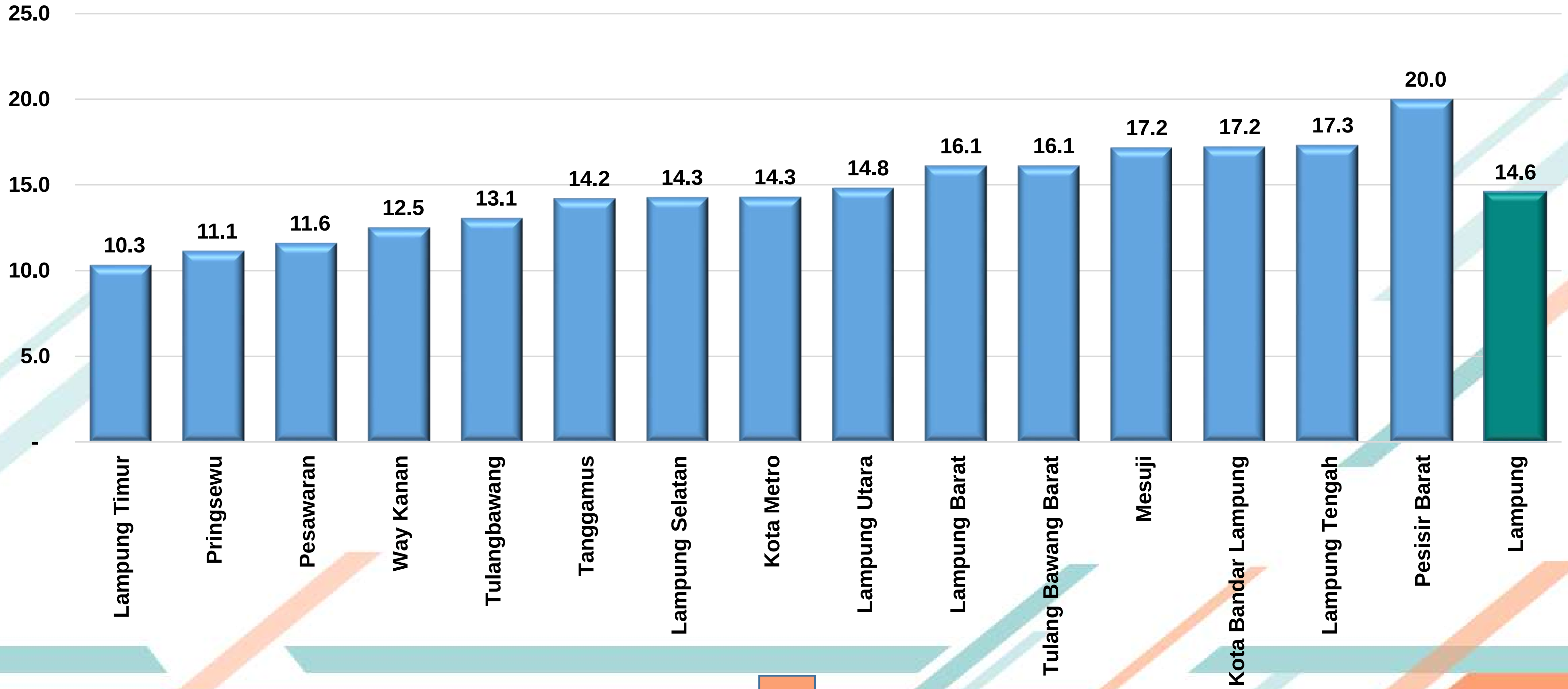

PREVALENSI BALITA *UNDERWEIGHT* (BERAT BADAN MENURUT UMUR) BERDASARKAN KABUPATEN/KOTA DI PROVINSI KEP.BABEL, SSGI 2021

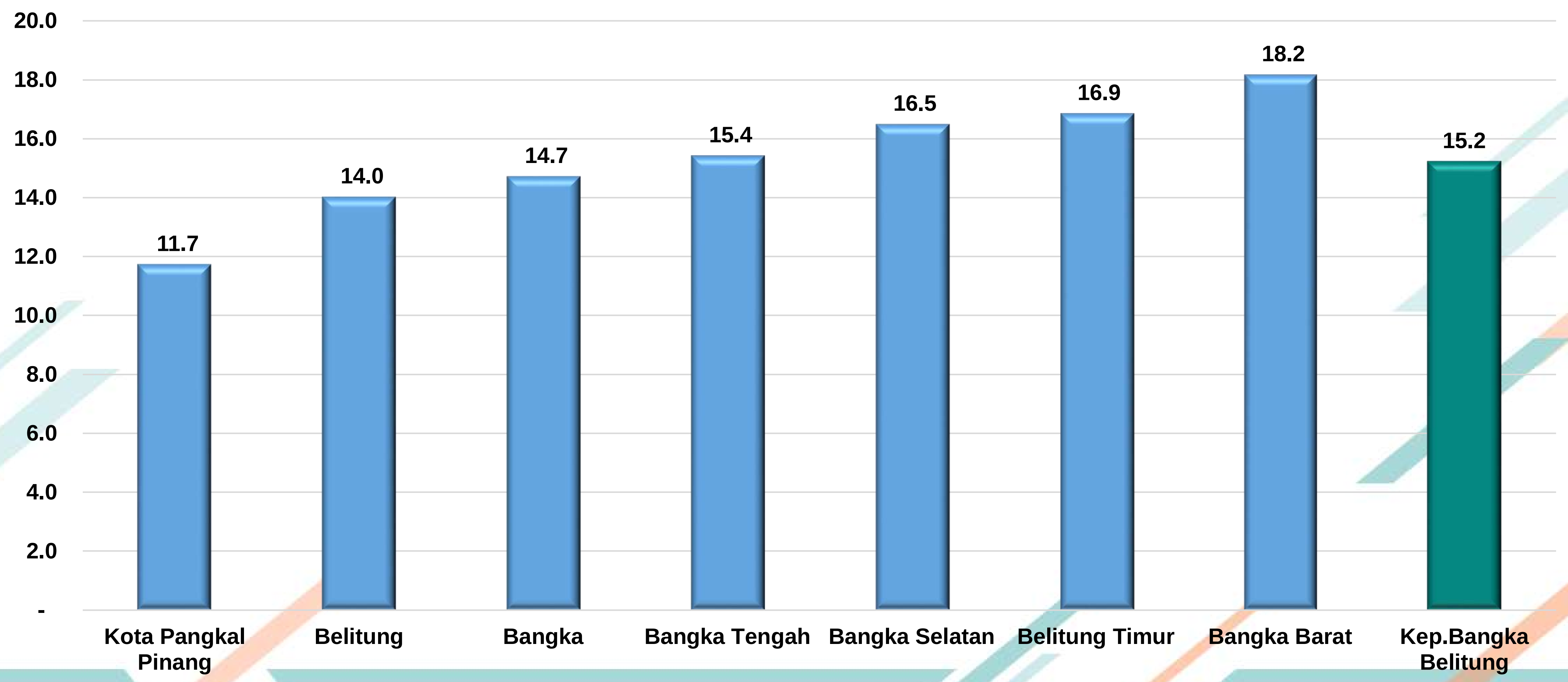

PREVALENSI BALITA *UNDERWEIGHT* (BERAT BADAN MENURUT UMUR) BERDASARKAN KABUPATEN/KOTA DI PROVINSI KEP.RIAU, SSGI 2021

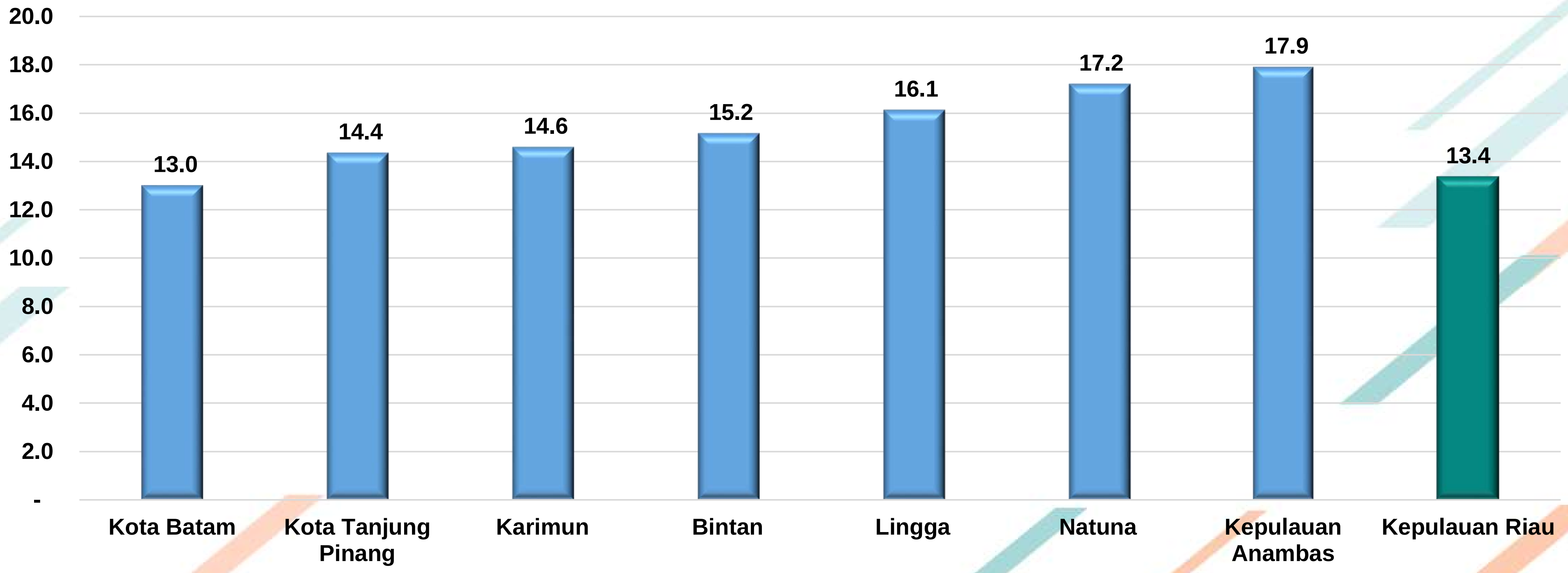

PREVALENSI BALITA *UNDERWEIGHT* (BERAT BADAN MENURUT UMUR) BERDASARKAN KABUPATEN/KOTA DI PROVINSI DKI JAKARTA, SSGI 2021

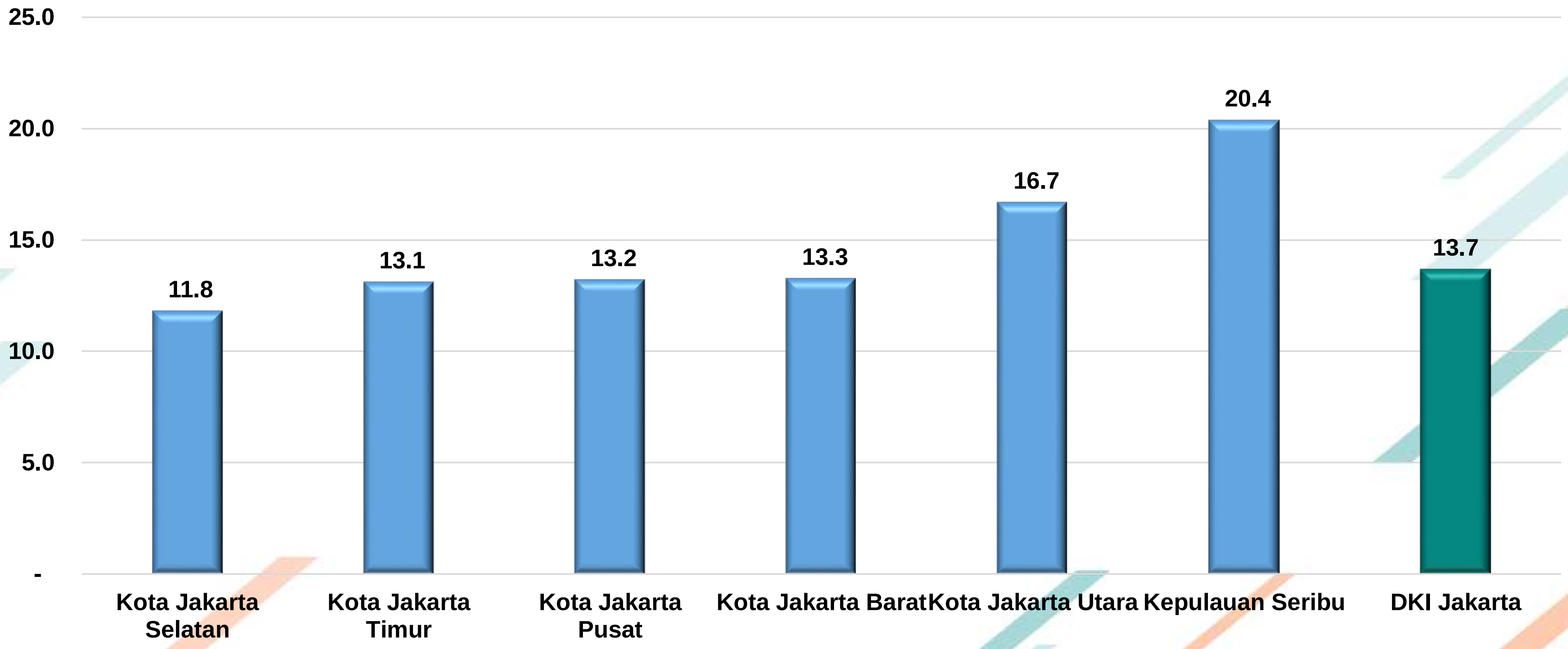

KEMENTERIAN KESEHATAN REPUBLIK INDONESIA

SSGI Studi Status Gizi Indonesia

PREVALENSI BALITA *UNDERWEIGHT* (BERAT BADAN MENURUT UMUR) BERDASARKAN KABUPATEN/KOTA DI PROVINSI JAWA BARAT, SSGI 2021

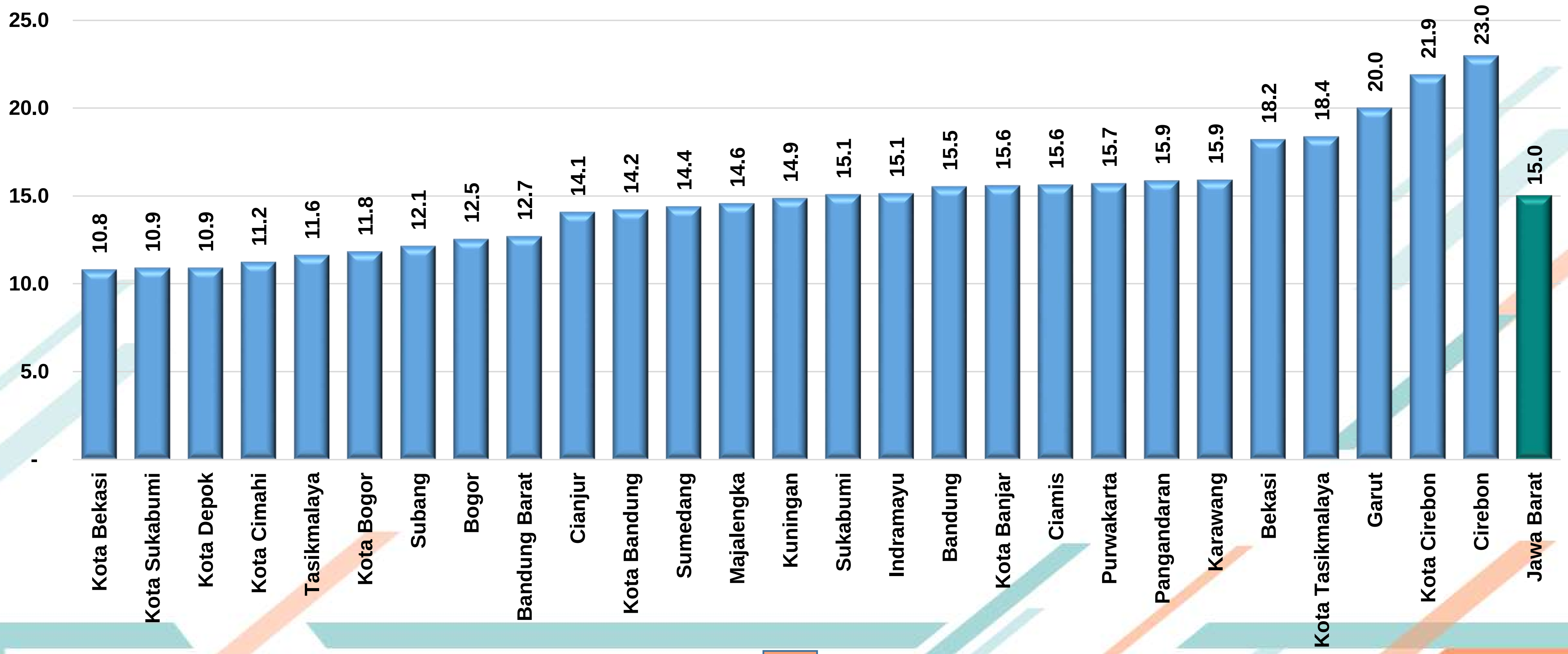

PREVALENSI BALITA *UNDERWEIGHT* (BERAT BADAN MENURUT UMUR) BERDASARKAN KABUPATEN/KOTA DI PROVINSI JAWA TENGAH, SSGI 2021

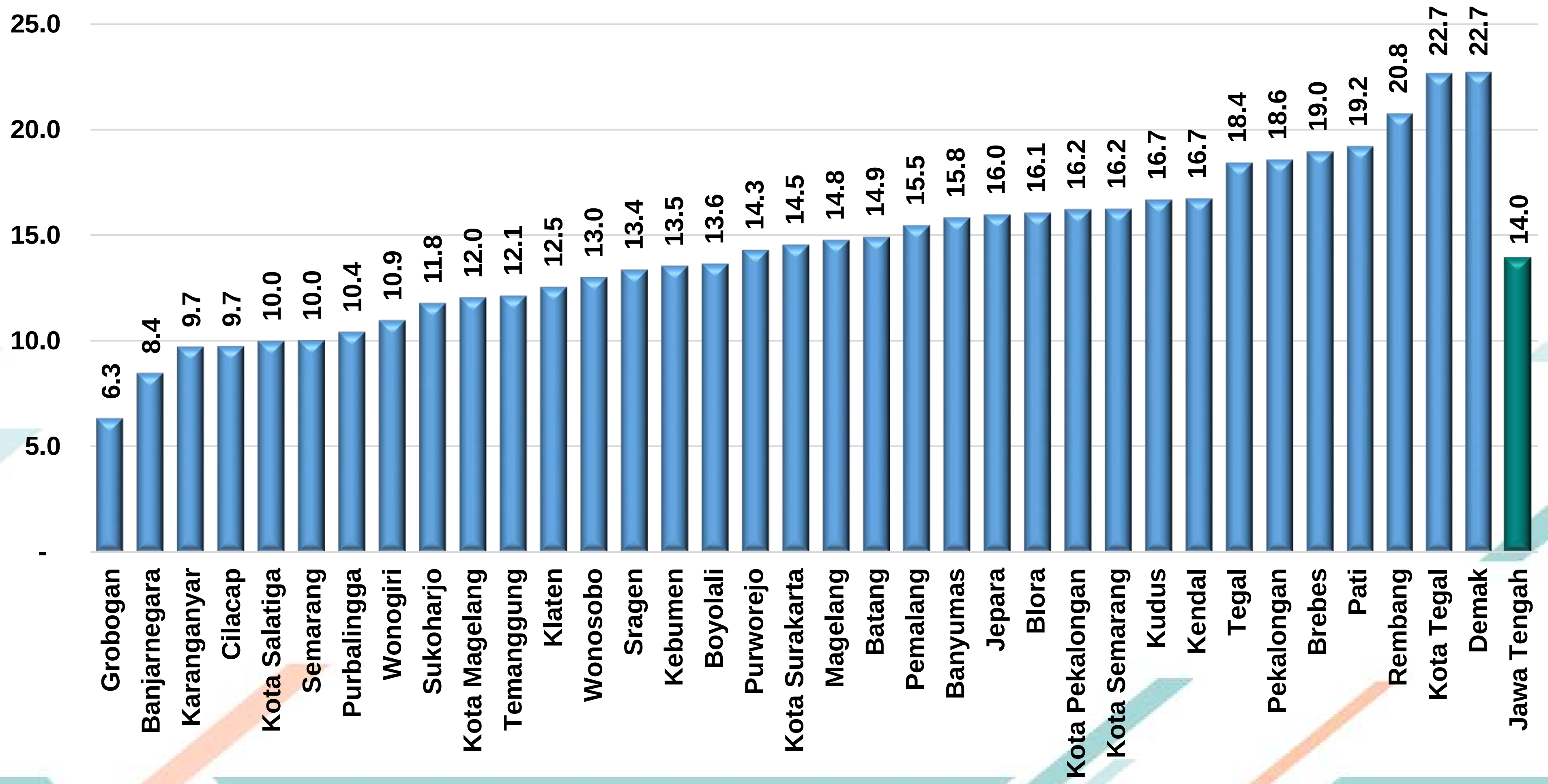

PREVALENSI BALITA *UNDERWEIGHT* (BERAT BADAN MENURUT UMUR) BERDASARKAN KABUPATEN/KOTA DI PROVINSI DIY, SSGI 2021

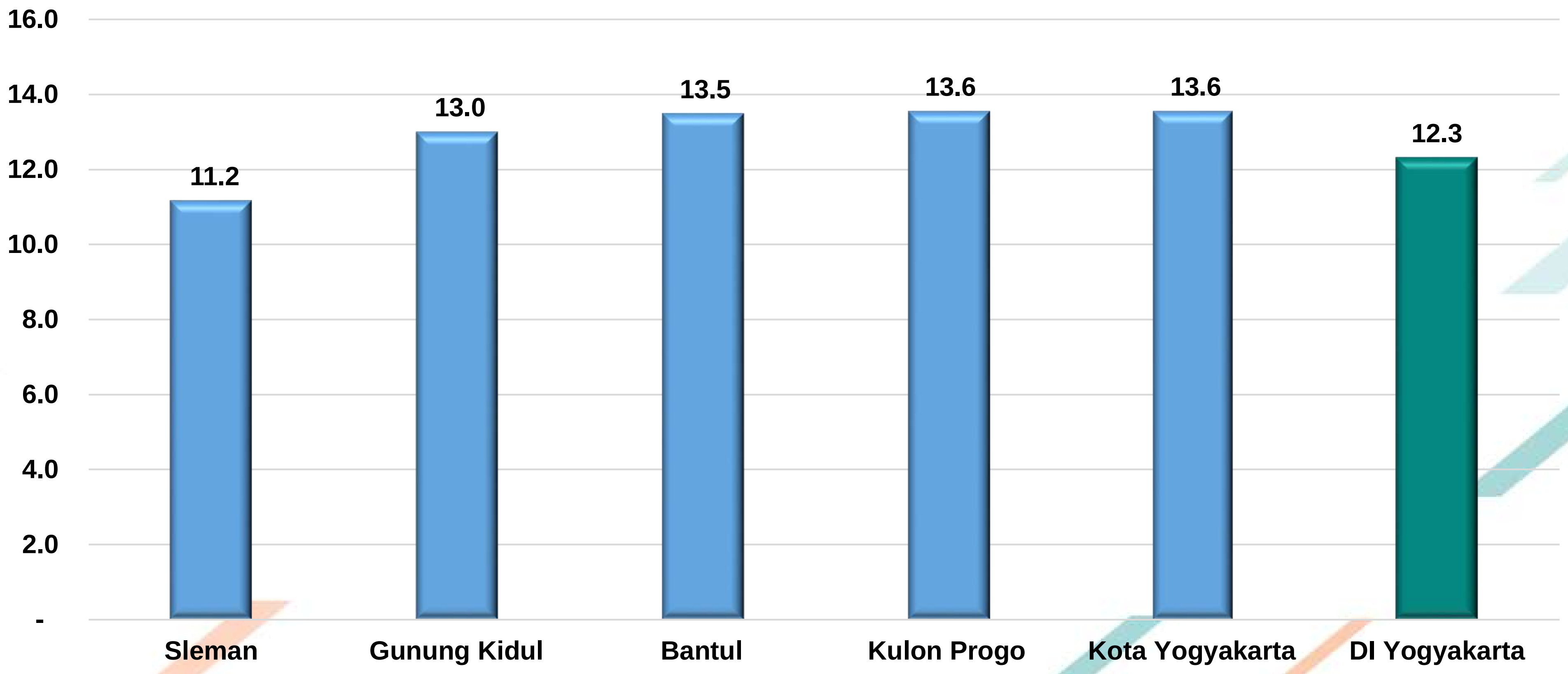

KEMENTERIAN KESEHATAN REPUBLIK INDONESIA

SSGI
Studi Status Gizi Indonesia

PREVALENSI BALITA *UNDERWEIGHT* (BERAT BADAN MENURUT UMUR) BERDASARKAN KABUPATEN/KOTA DI PROVINSI JAWA TIMUR, SSGI 2021

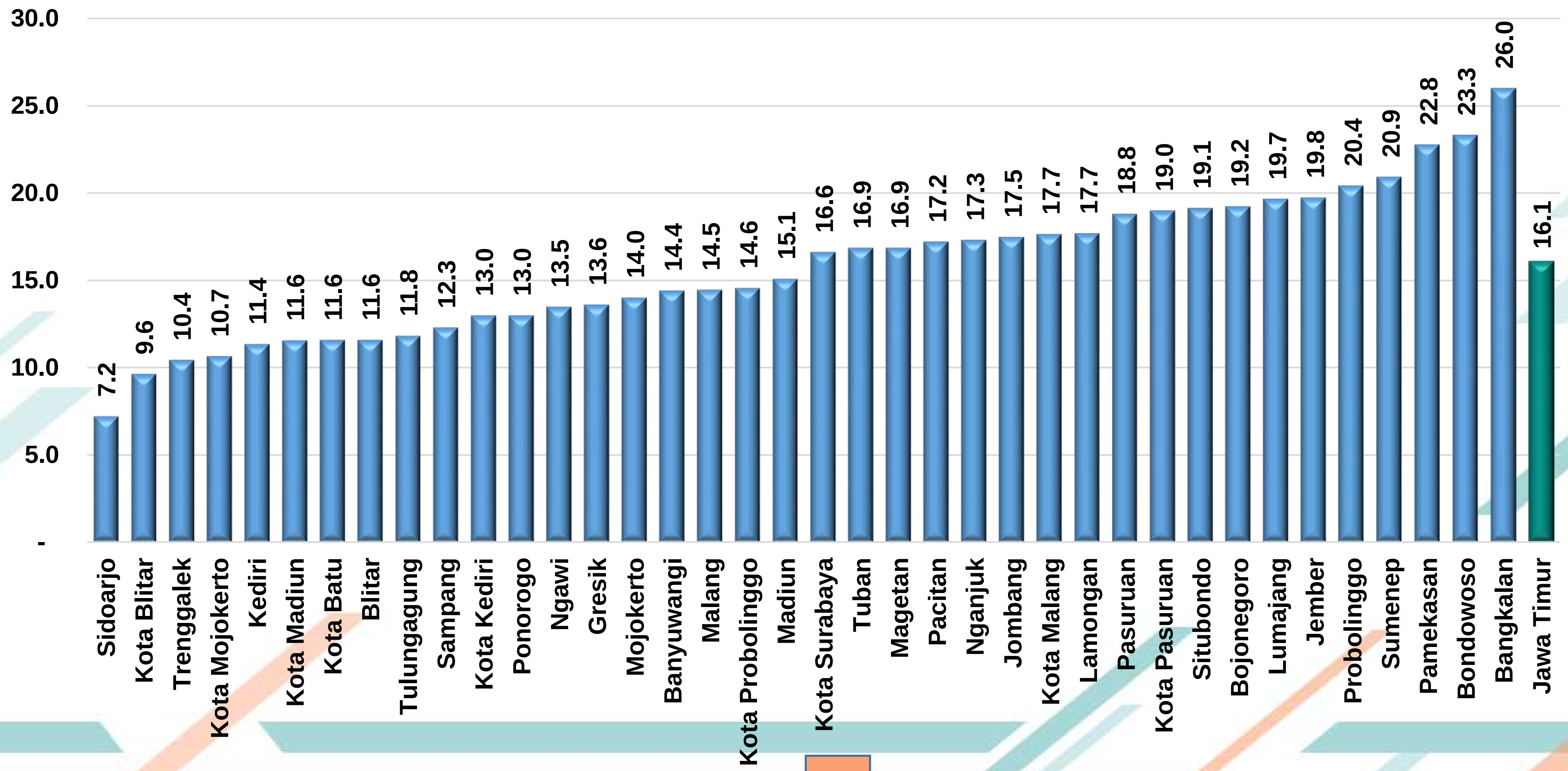

PREVALENSI BALITA *UNDERWEIGHT* (BERAT BADAN MENURUT UMUR) BERDASARKAN KABUPATEN/KOTA DI PROVINSI BANTEN, SSGI 2021

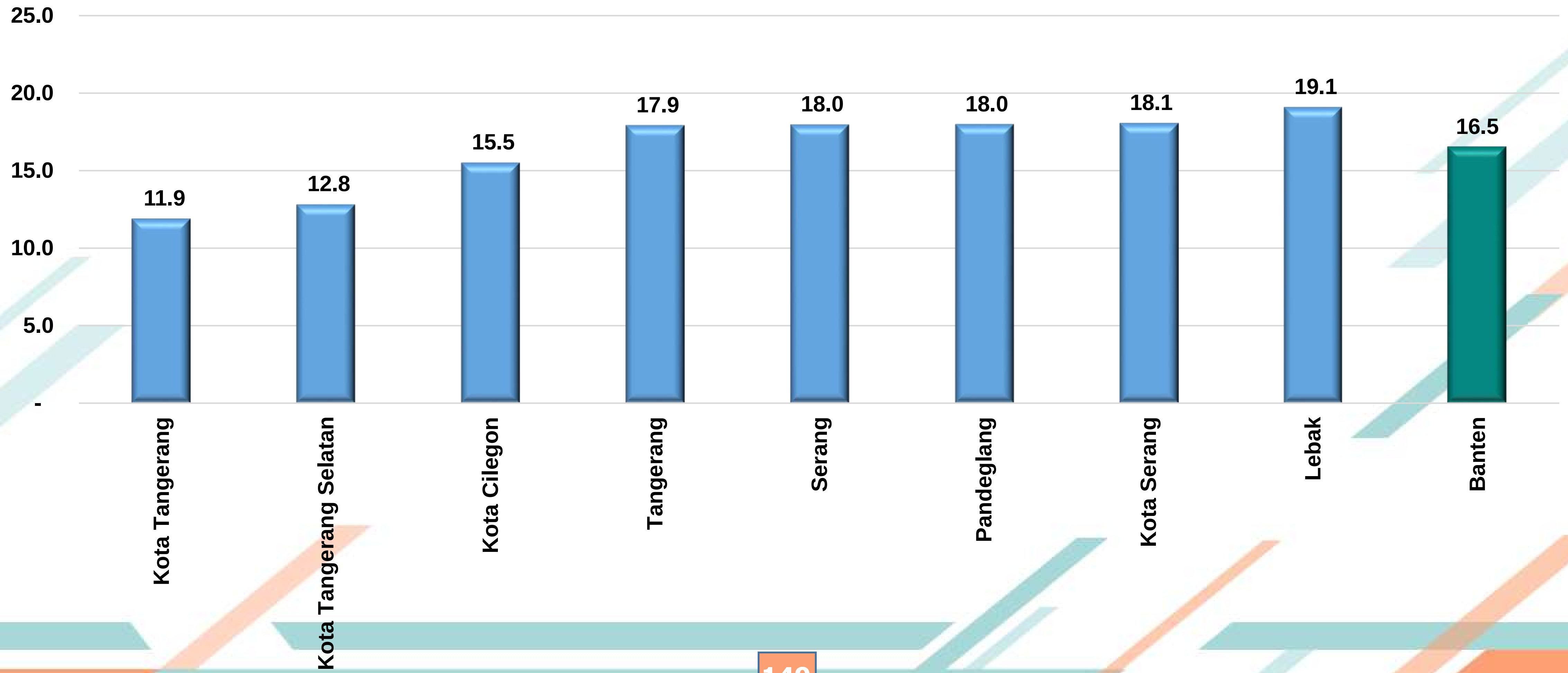

KEMENTERIAN KESEHATAN REPUBLIK INDONESIA

SSGI Studi Status Gizi Indonesia

PREVALENSI BALITA *UNDERWEIGHT* (BERAT BADAN MENURUT UMUR) BERDASARKAN KABUPATEN/KOTA DI PROVINSI BALI, SSGI 2021

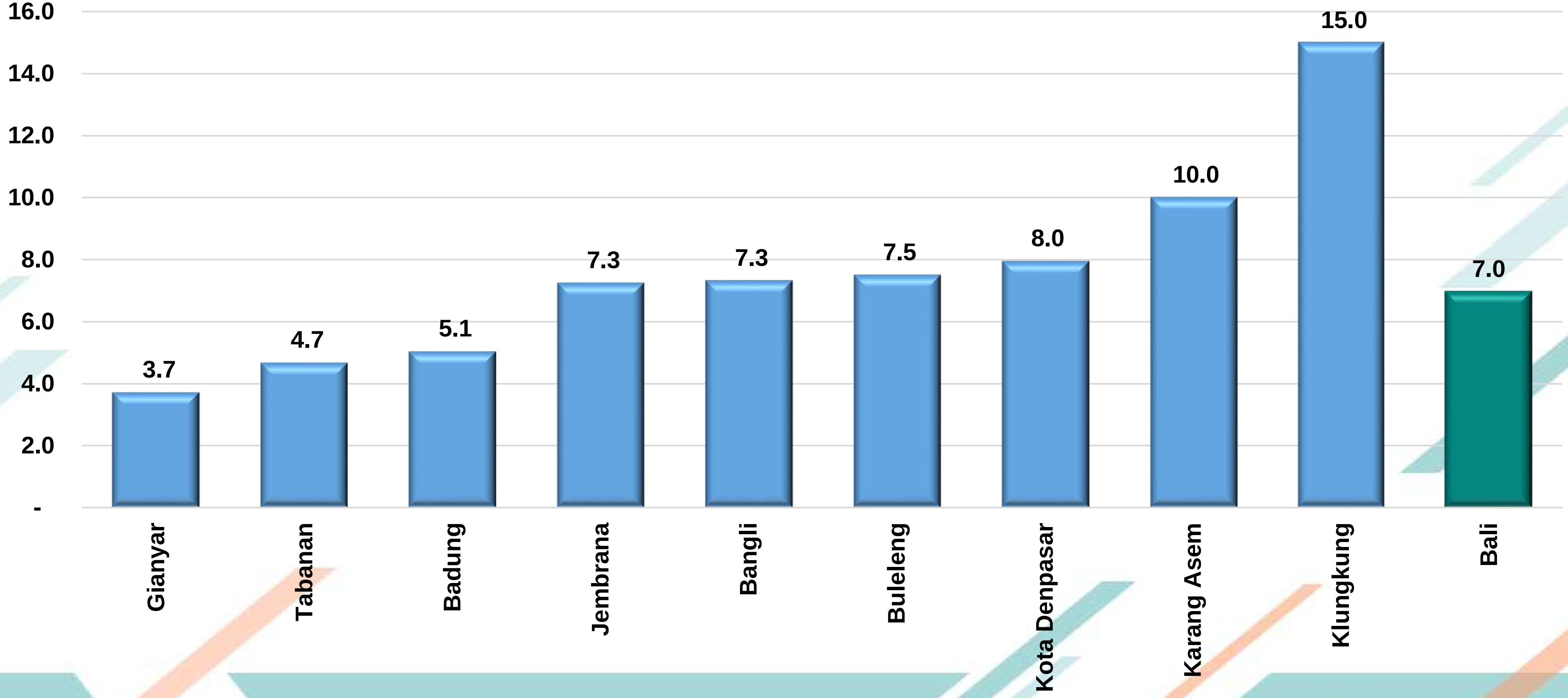

PREVALENSI BALITA *UNDERWEIGHT* (BERAT BADAN MENURUT UMUR) BERDASARKAN KABUPATEN/KOTA DI PROVINSI NTB, SSGI 2021

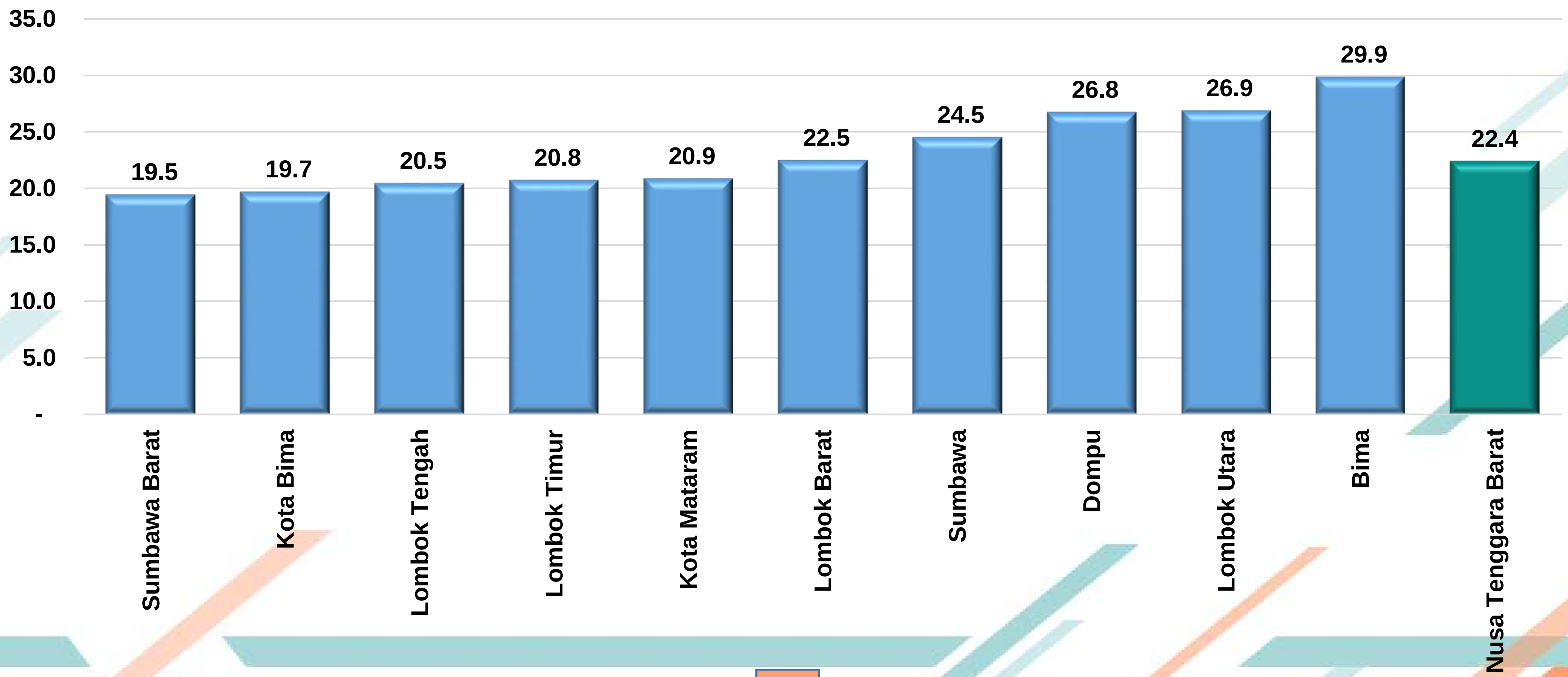

PREVALENSI BALITA *UNDERWEIGHT* (BERAT BADAN MENURUT UMUR) BERDASARKAN KABUPATEN/KOTA DI PROVINSI NTT, SSGI 2021

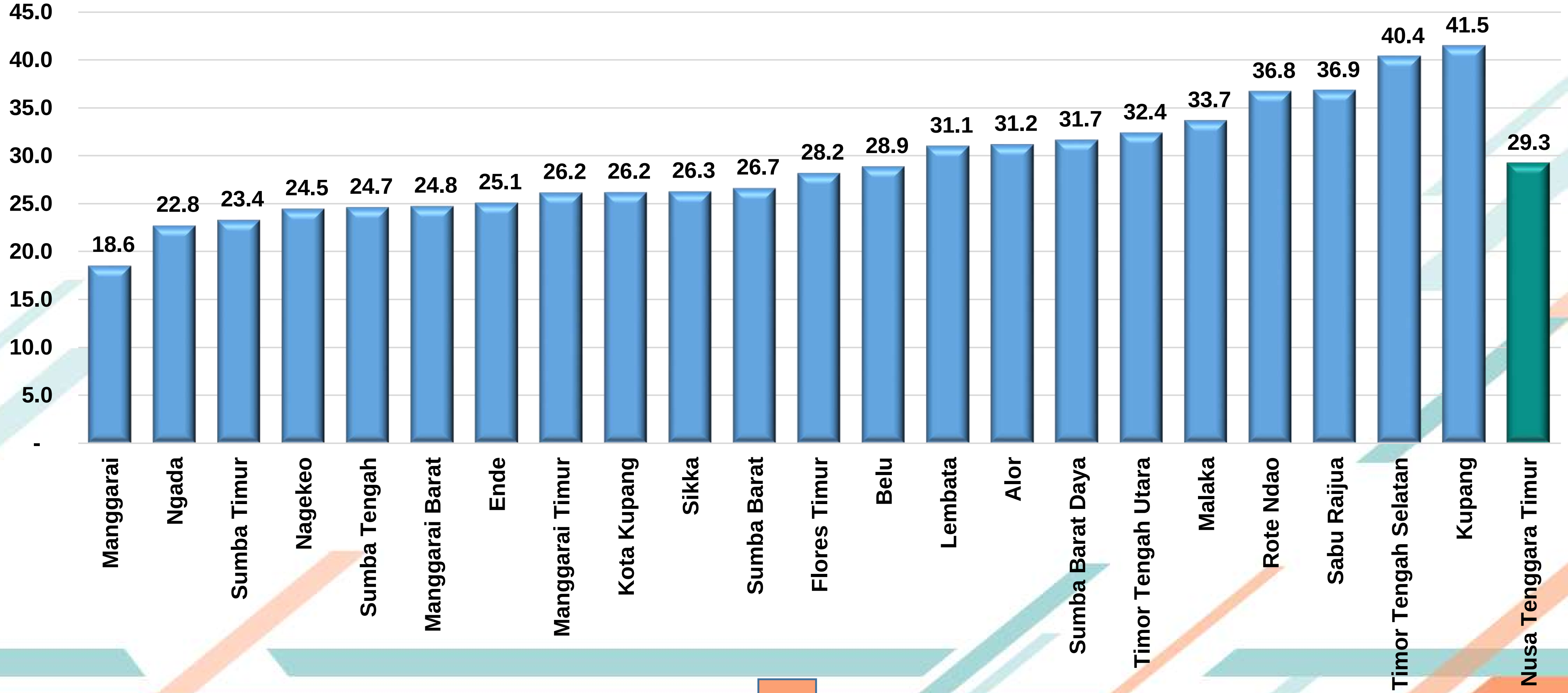

PREVALENSI BALITA *UNDERWEIGHT* (BERAT BADAN MENURUT UMUR) BERDASARKAN KABUPATEN/KOTA DI PROVINSI KALIMANTAN BARAT, SSGI 2021

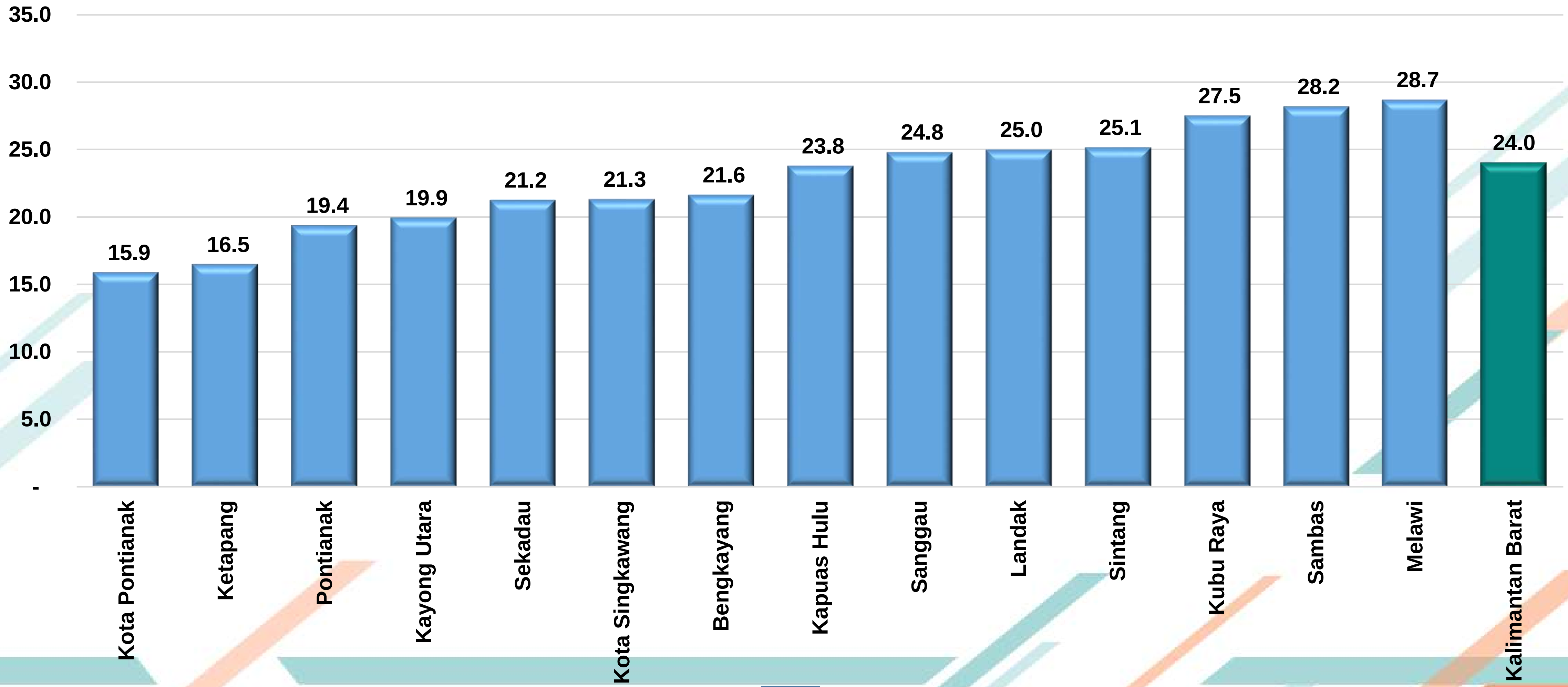

KEMENTERIAN KESEHATAN REPUBLIK INDONESIA

SSGI Studi Status Gizi Indonesia

PREVALENSI BALITA *UNDERWEIGHT* (BERAT BADAN MENURUT UMUR) BERDASARKAN KABUPATEN/KOTA DI PROVINSI KALIMANTAN TENGAH, SSGI 2021

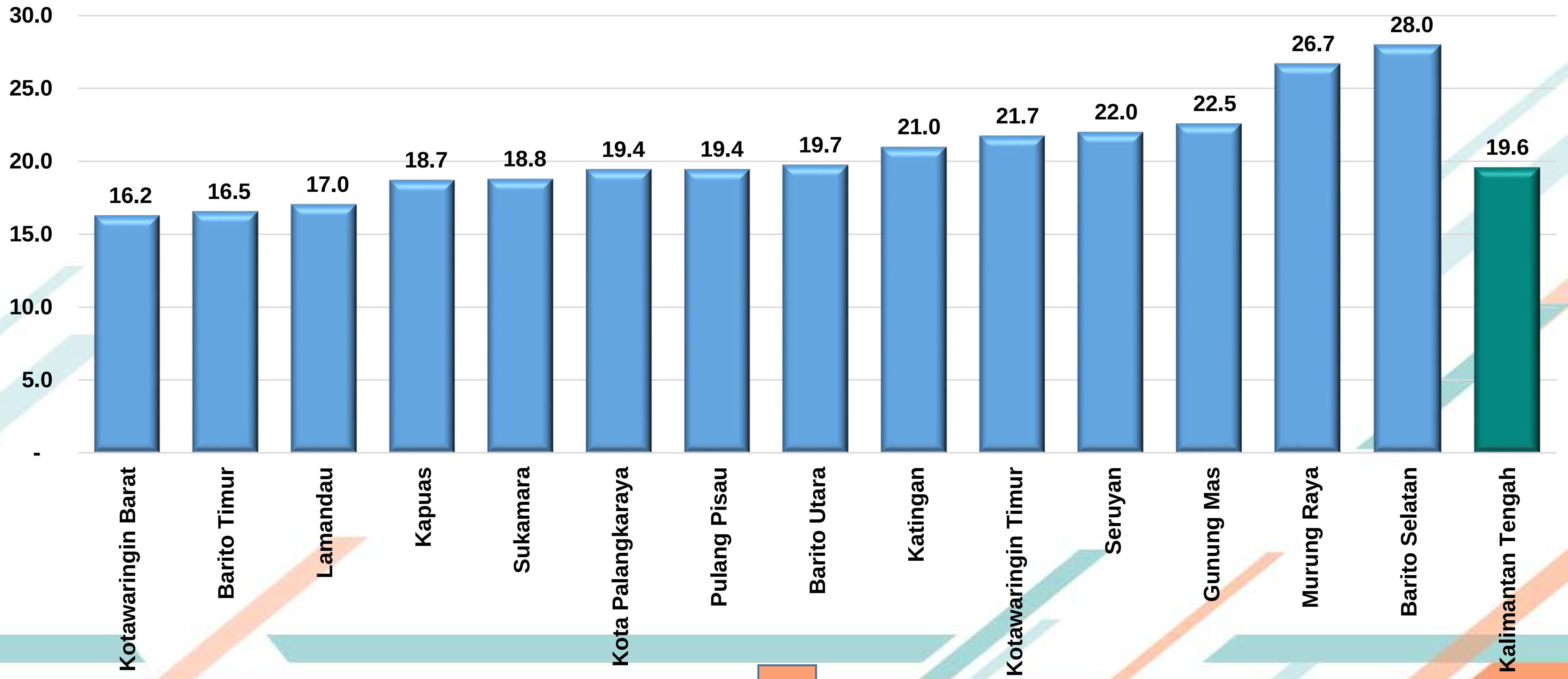

KEMENTERIAN KESEHATAN REPUBLIK INDONESIA

SSGI
Studi Status Gizi Indonesia

PREVALENSI BALITA *UNDERWEIGHT* (BERAT BADAN MENURUT UMUR) BERDASARKAN KABUPATEN/KOTA DI PROVINSI KALIMANTAN SELATAN, SSGI 2021

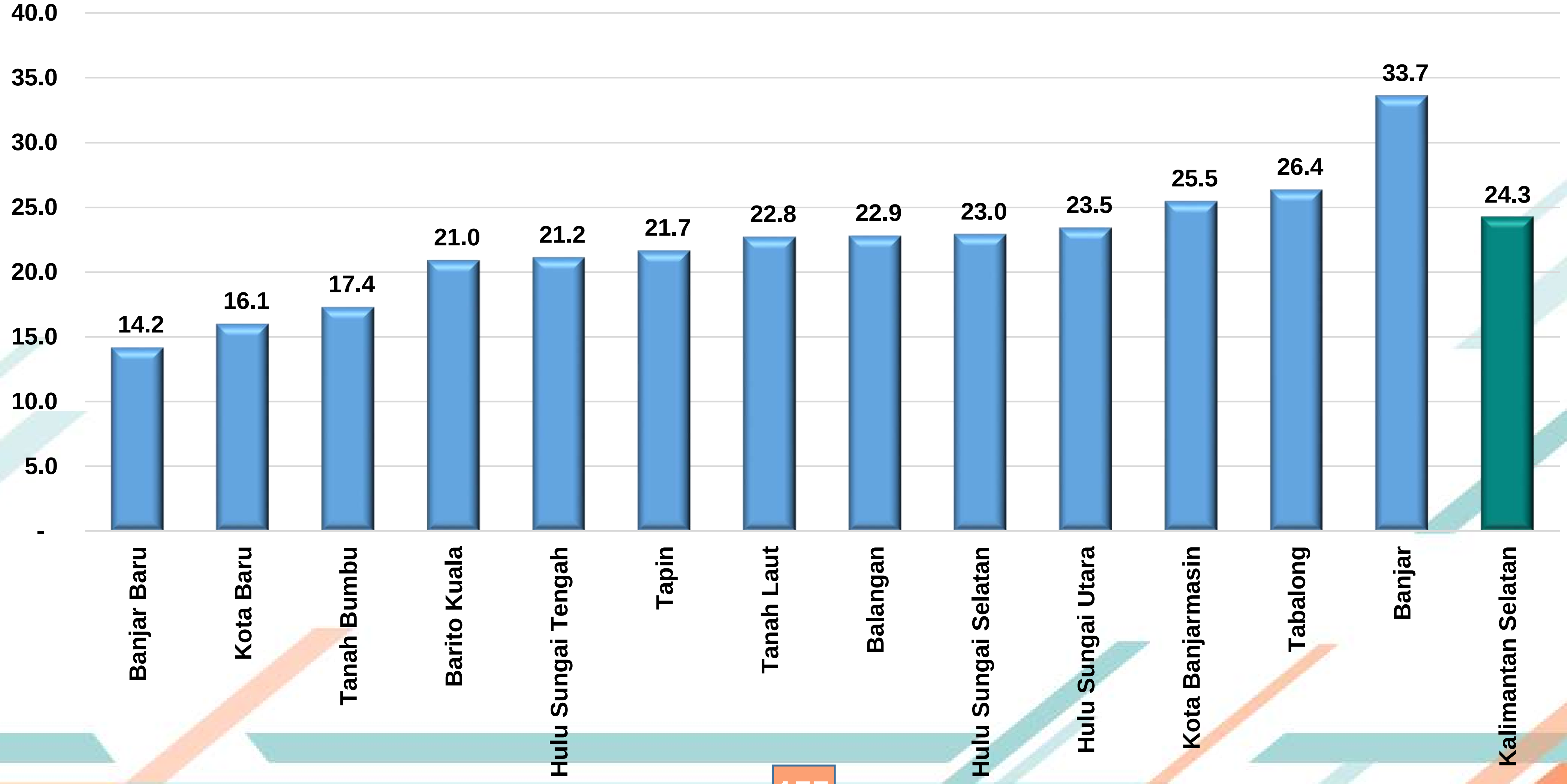

PREVALENSI BALITA *UNDERWEIGHT* (BERAT BADAN MENURUT UMUR) BERDASARKAN KABUPATEN/KOTA DI PROVINSI KALIMANTAN TIMUR, SSGI 2021

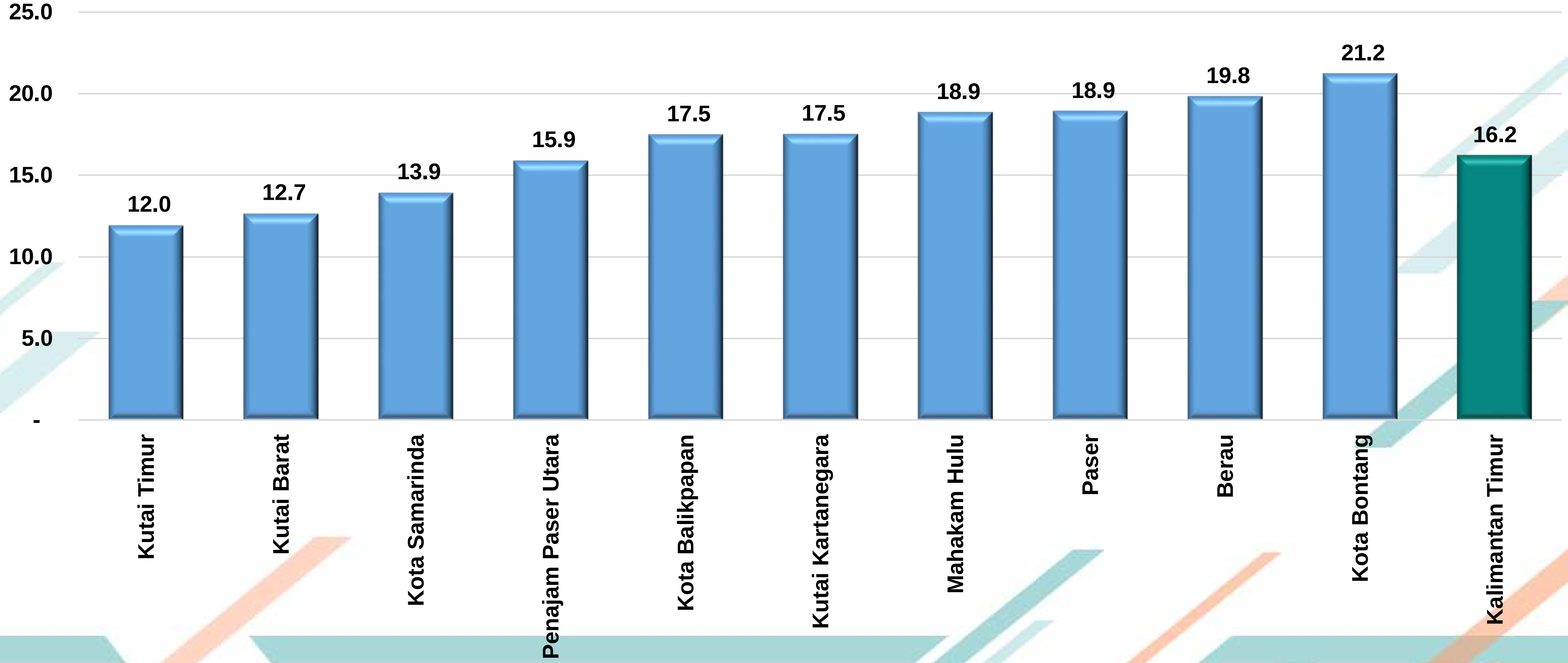

KEMENTERIAN KESEHATAN REPUBLIK INDONESIA

SSGI Studi Status Gizi Indonesia

PREVALENSI BALITA *UNDERWEIGHT* (BERAT BADAN MENURUT UMUR) BERDASARKAN KABUPATEN/KOTA DI PROVINSI KALIMANTAN UTARA, SSGI 2021

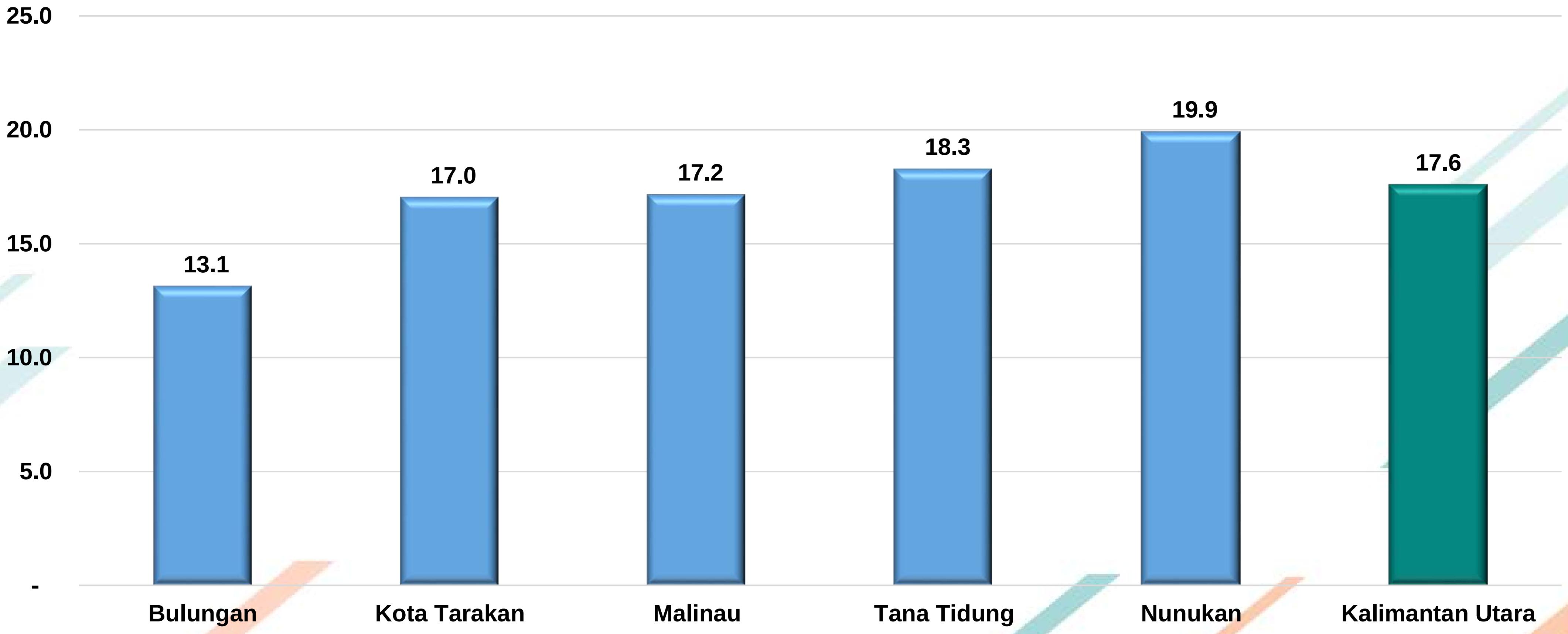

KEMENTERIAN KESEHATAN REPUBLIK INDONESIA

SSGI Studi Status Gizi Indonesia

PREVALENSI BALITA *UNDERWEIGHT* (BERAT BADAN MENURUT UMUR) BERDASARKAN KABUPATEN/KOTA DI PROVINSI SULAWESI UTARA, SSGI 2021

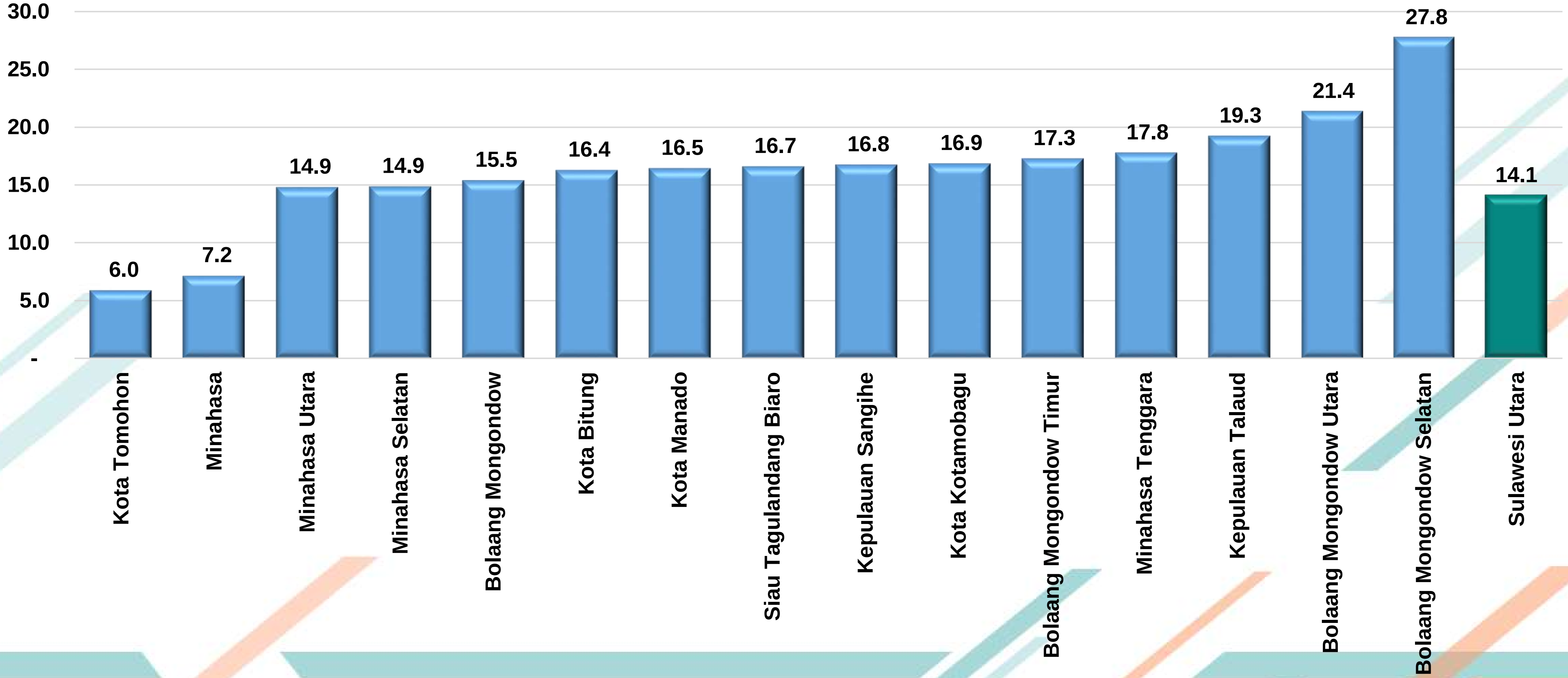

KEMENTERIAN KESEHATAN REPUBLIK INDONESIA

SSGI
Studi Status Gizi Indonesia

PREVALENSI BALITA *UNDERWEIGHT* (BERAT BADAN MENURUT UMUR) BERDASARKAN KABUPATEN/KOTA DI PROVINSI SULAWESI TENGAH, SSGI 2021

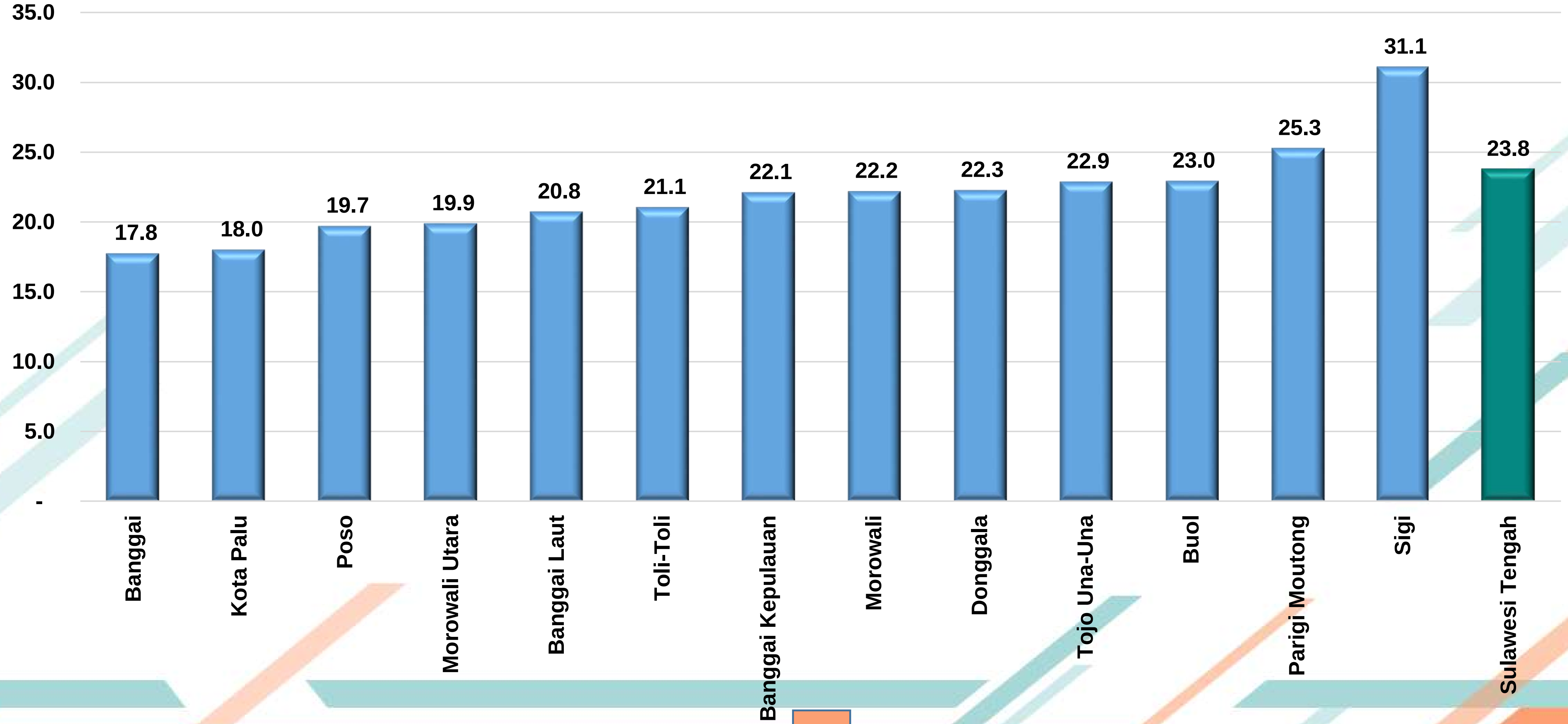

PREVALENSI BALITA *UNDERWEIGHT* (BERAT BADAN MENURUT UMUR) BERDASARKAN KABUPATEN/KOTA DI PROVINSI SULAWESI SELATAN, SSGI 2021

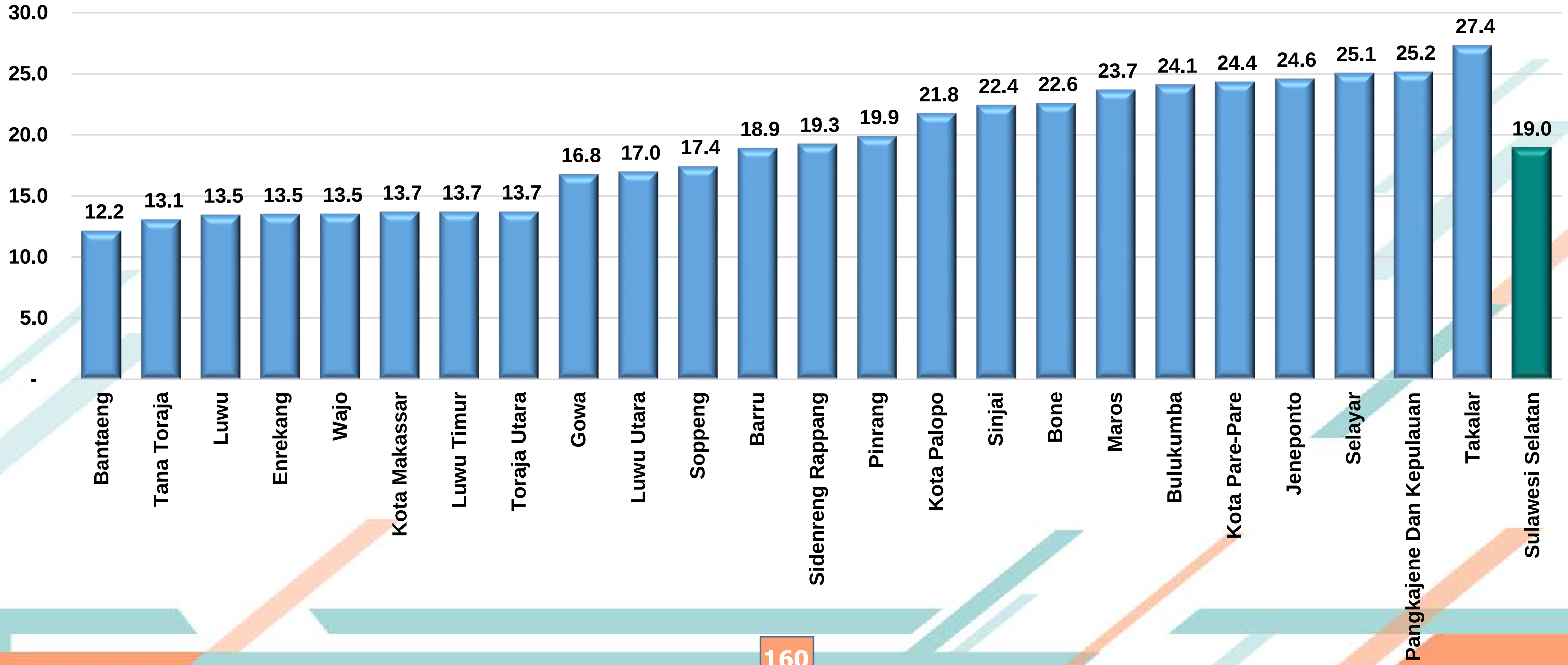

PREVALENSI BALITA *UNDERWEIGHT* (BERAT BADAN MENURUT UMUR) BERDASARKAN KABUPATEN/KOTA DI PROVINSI SULAWESI TENGGARA, SSGI 2021

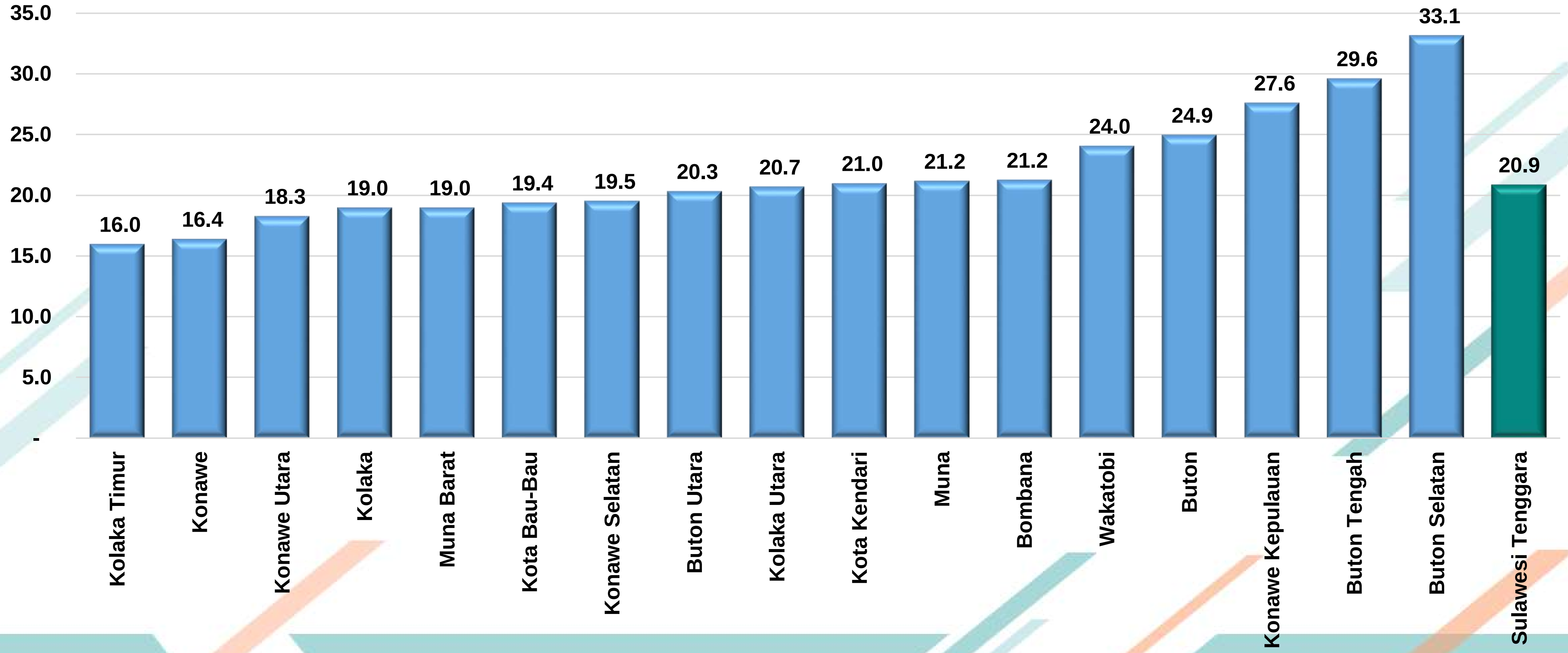

PREVALENSI BALITA *UNDERWEIGHT* (BERAT BADAN MENURUT UMUR) BERDASARKAN KABUPATEN/KOTA DI PROVINSI GORONTALO, SSGI 2021

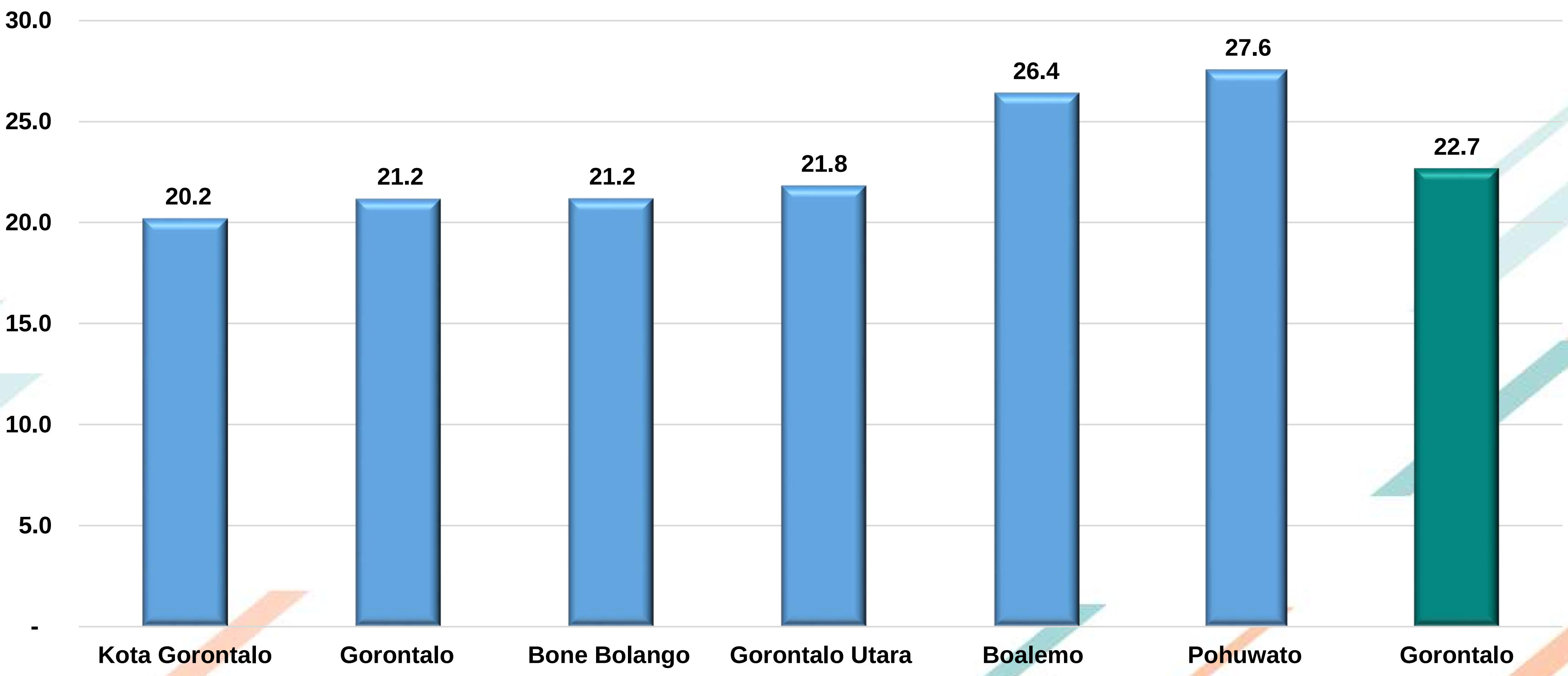

KEMENTERIAN KESEHATAN REPUBLIK INDONESIA

SSGI Studi Status Gizi Indonesia

PREVALENSI BALITA *UNDERWEIGHT* (BERAT BADAN MENURUT UMUR) BERDASARKAN KABUPATEN/KOTA DI PROVINSI SULAWESI BARAT, SSGI 2021

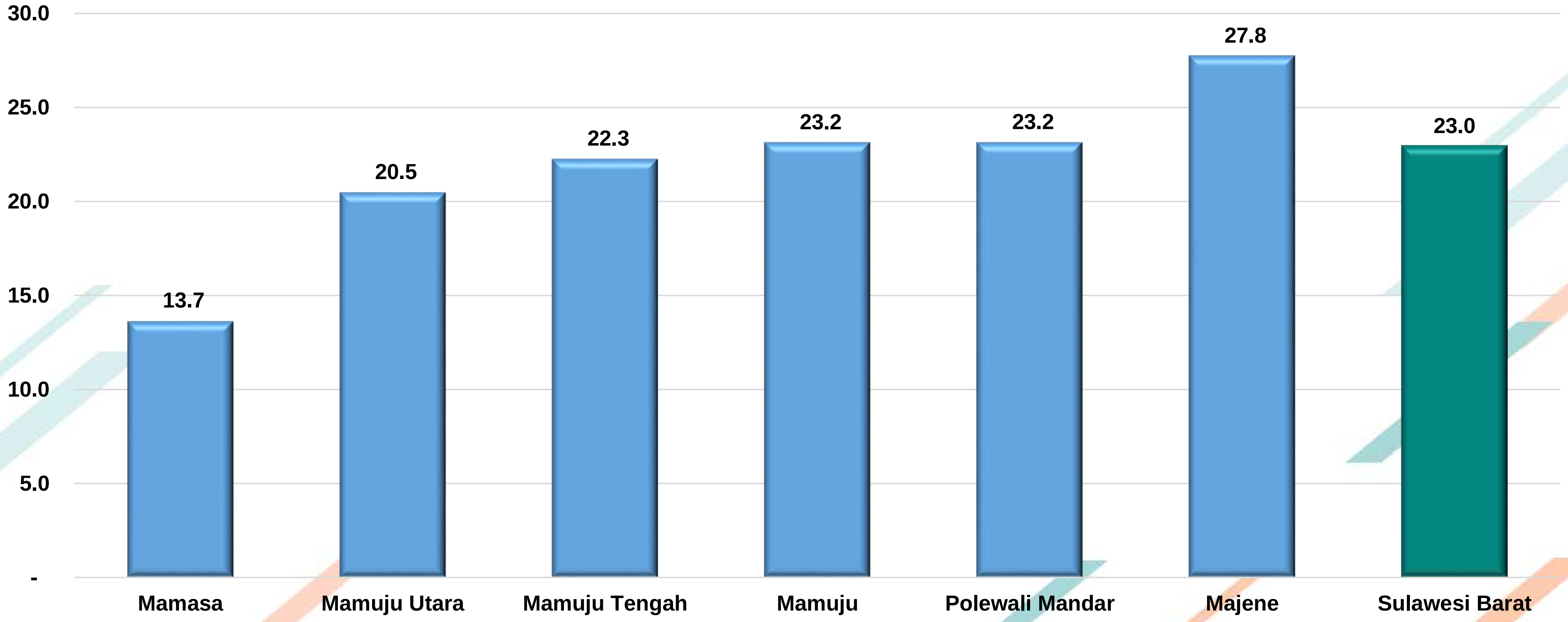

PREVALENSI BALITA *UNDERWEIGHT* (BERAT BADAN MENURUT UMUR) BERDASARKAN KABUPATEN/KOTA DI PROVINSI MALUKU, SSGI 2021

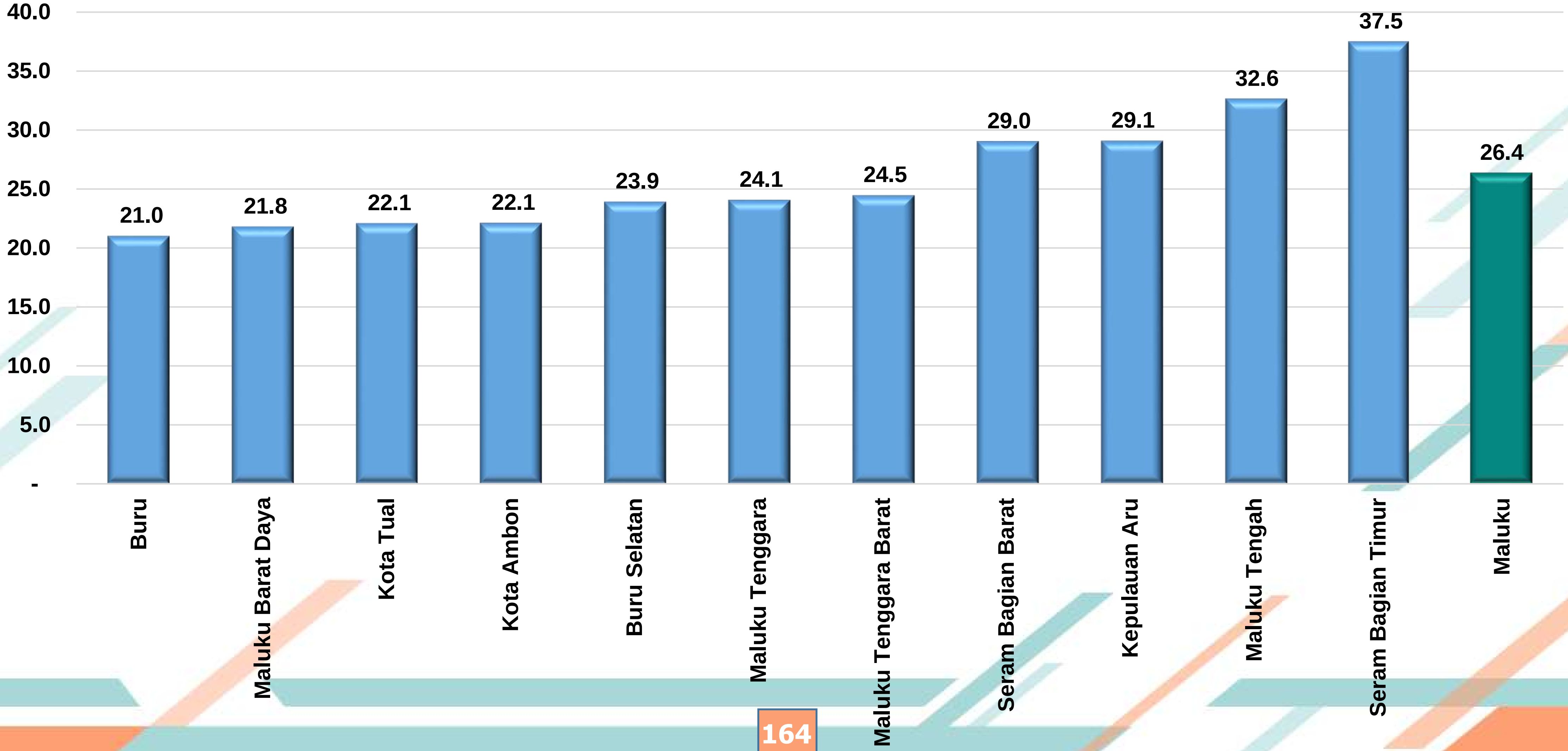

PREVALENSI BALITA *UNDERWEIGHT* (BERAT BADAN MENURUT UMUR) BERDASARKAN KABUPATEN/KOTA DI PROVINSI MALUKU UTARA, SSGI 2021

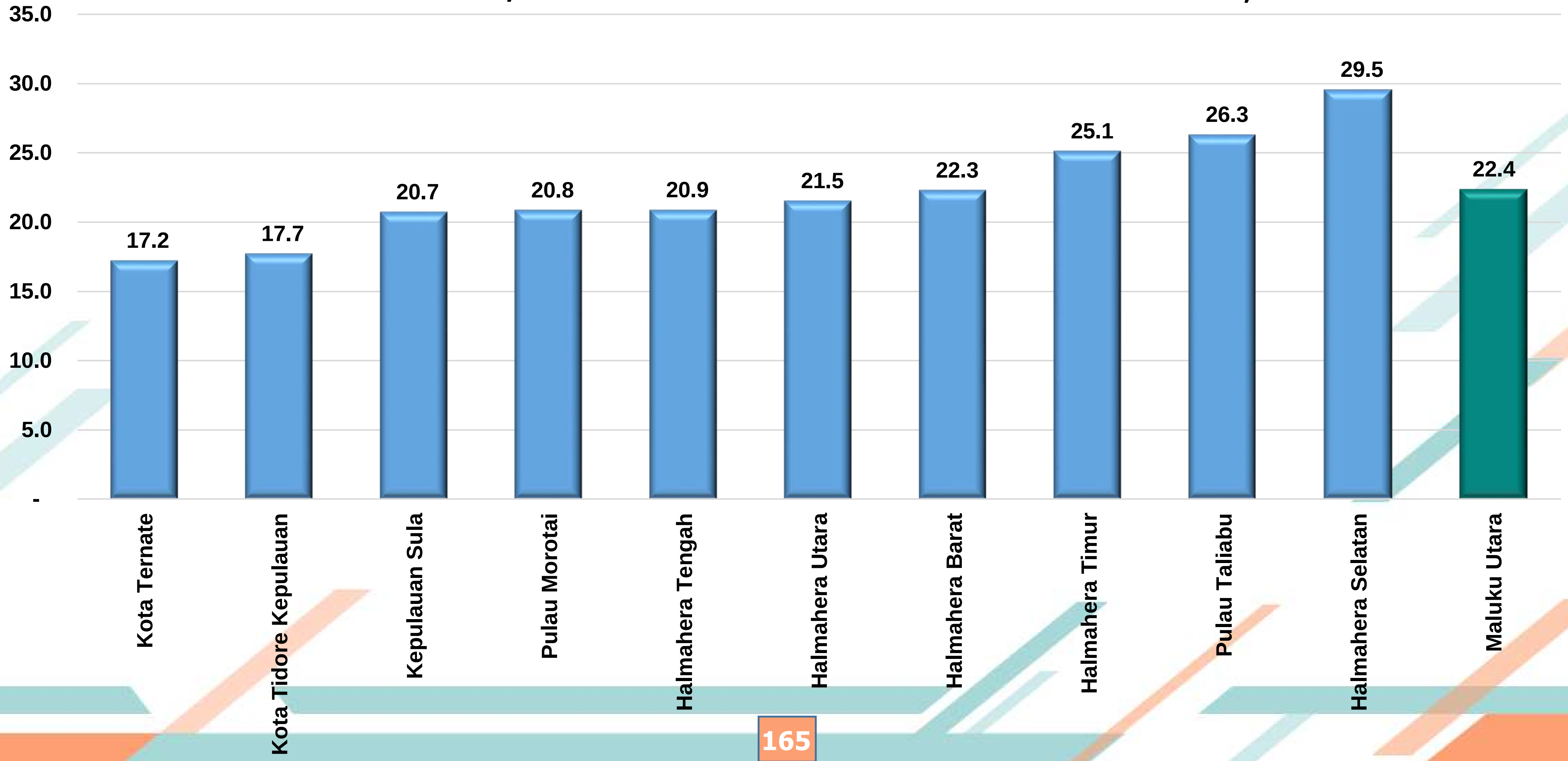

PREVALENSI BALITA *UNDERWEIGHT* (BERAT BADAN MENURUT UMUR) BERDASARKAN KABUPATEN/KOTA DI PROVINSI PAPUA BARAT, SSGI 2021

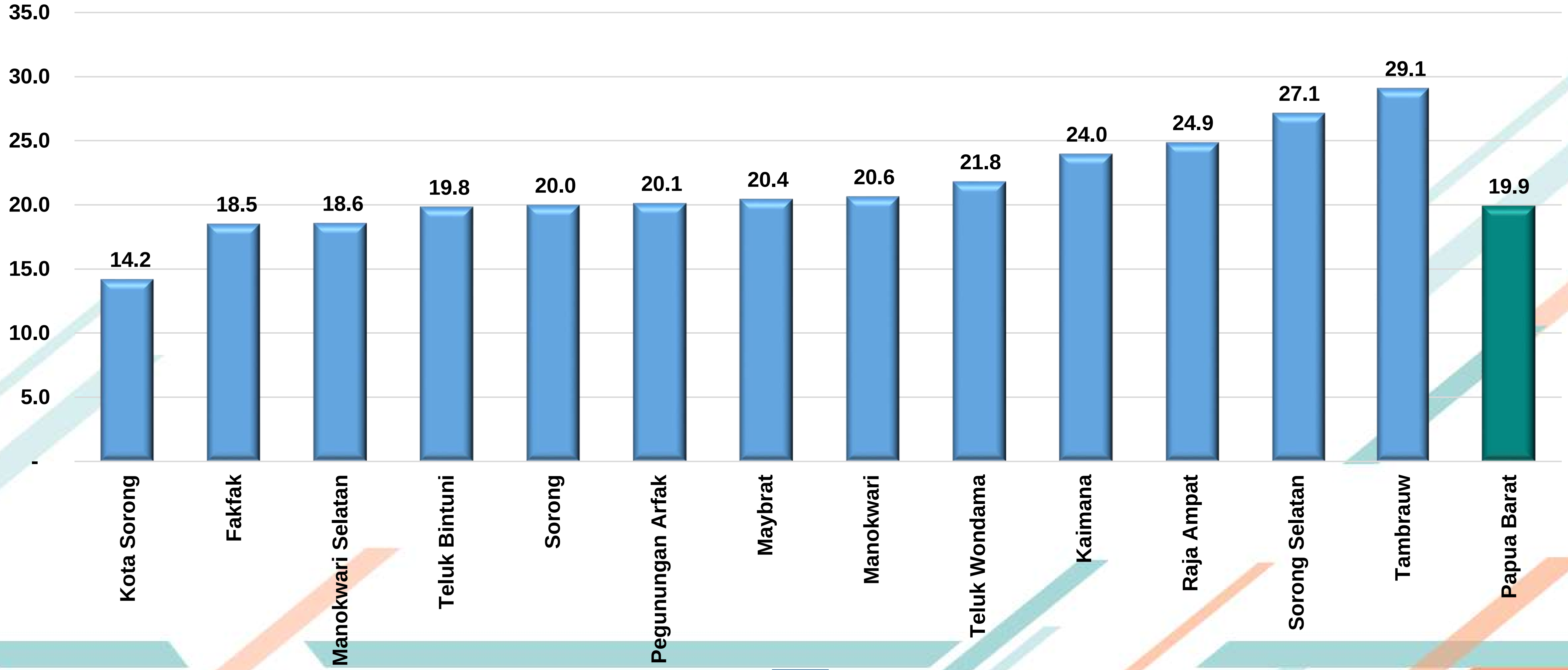

KEMENTERIAN KESEHATAN REPUBLIK INDONESIA

SSGI

PREVALENSI BALITA *UNDERWEIGHT* (BERAT BADAN MENURUT UMUR) BERDASARKAN KABUPATEN/KOTA DI PROVINSI PAPUA, SSGI 2021

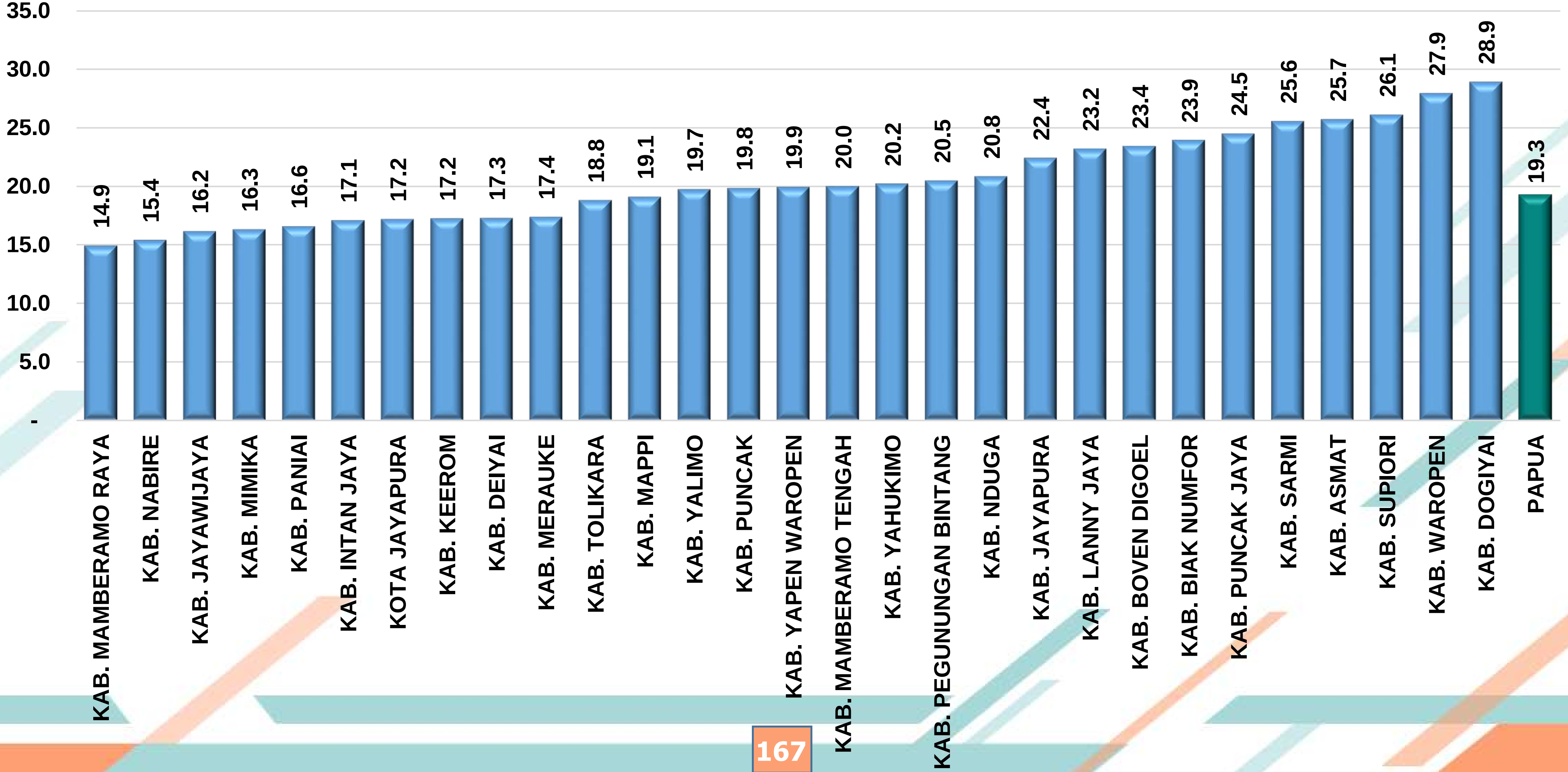

KEMENTERIAN KESEHATAN REPUBLIK INDONESIA

SSGI
Studi Status Gizi Indonesia

Terima kasih
